# Selective Late‐Stage Sulfonyl Chloride Formation from Sulfonamides Enabled by Pyry‐BF_4_

**DOI:** 10.1002/anie.201910895

**Published:** 2019-10-28

**Authors:** Alejandro Gómez‐Palomino, Josep Cornella

**Affiliations:** ^1^ Max-Planck-Institut für Kohlenforschung Kaiser-Wilhelm-Platz 1 45470 Mülheim an der Ruhr Germany

**Keywords:** amines, heterocycles, nucleophilic addition, sulfonamides, synthetic methods

## Abstract

Reported here is a simple and practical functionalization of primary sulfonamides, by means of a pyrylium salt (Pyry‐BF_4_), with nucleophiles. This simple reagent activates the poorly nucleophilic NH_2_ group in a sulfonamide, enabling the formation of one of the best electrophiles in organic synthesis: a sulfonyl chloride. Because of the variety of primary sulfonamides in pharmaceutical contexts, special attention has been focused on the direct conversion of densely functionalized primary sulfonamides by a late‐stage formation of the corresponding sulfonyl chloride. A variety of nucleophiles could be engaged in this transformation, thus permitting the synthesis of complex sulfonamides, sulfonates, sulfides, sulfonyl fluorides, and sulfonic acids. The mild reaction conditions and the high selectivity of Pyry‐BF_4_ towards NH_2_ groups permit the formation of sulfonyl chlorides in a late‐stage fashion, tolerating a preponderance of sensitive functionalities.

## Graphical abstract

**It's never too late**: The conversion of primary sulfonamides into sulfonyl chlorides has been achieved by capitalizing on the use of Pyry‐BF_4_ as activating reagent. The mild reaction conditions and high selectivity of the protocol permit the presence of a preponderance of functional groups and the conversion of poorly nucleophilic and unreactive sulfonamides for late‐stage functionalizations.

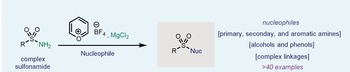

Since the seminal report of the antibacterial activity of sulfonamides from the collaborative effort by Domagk, Mietzsch, and Klarer in 1932 at I. G. Farbenindustrie,^1^ sulfonamide‐based drugs represented a turning point in the history of medicine and became of capital importance for the treatment of a great variety of diseases.^2^ With Prontosil as the flagship compound, the sulfonamide linkage is still an ever‐present motif in a large variety of biologically relevant compounds (Figure 1).^3^ For this reason, intense efforts have been focused on developing efficient strategies for their synthesis, and a plethora of methods have appeared in the literature.^4^ However, a closer analysis of these precedents reveals that primary sulfonamide moiety has always been conceived as an endgame point, rather than a modifiable handle for derivatization. Based on the importance of sulfur‐containing motifs for drug discovery and their large presence in many medicinal chemistry libraries,^2^ we envisaged that primary sulfonamides could be repurposed as powerful coupling partners in drug discovery if methods to convert the NH_2_ into other functionalities would exist. Indeed, the traditional and most common strategy for the formation of complex sulfonamides has been the reaction between a sulfonyl chloride and the corresponding amine.^5^ Over many years, sulfonyl chlorides have become extremely powerful electrophiles, permitting a great variety of nucleophiles to be engaged in fast and selective couplings.^6^ Although alternative methodologies have been reported,^7^ the synthesis of sulfonyl chlorides has been largely dominated by the conversion of sulfonic acids through dehydration by the use of highly oxidizing and unselective reagents namely POCl_3_, SO_2_Cl_2_, and derivatives thereof (Figure 2 A).^8^ Yet, the low chemoselectivity and the low functional‐group compatibility of these reagents have restricted its widespread use when more complex targets are required. As a result, the powerful reactivity of sulfonyl chlorides has only been harvested in earlier stages of medicinal chemistry campaigns, resulting in virtually inexistent methods for late‐stage functionalization contexts.

**Figure 1:**
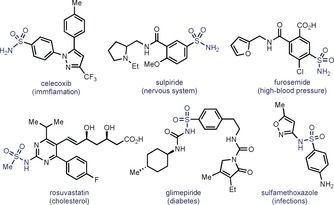
The sulfonamide bond in medicinally relevant compounds.

**Figure 2:**
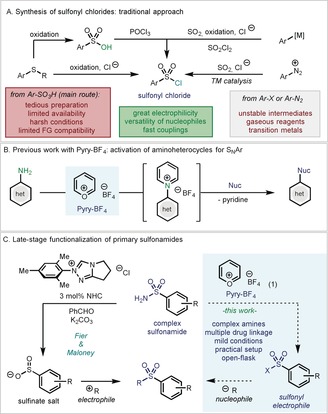
A) Synthetic approaches to sulfonyl chlorides. B) Prior work on the application of Pyry‐BF_4_ (**1**) in S_*N*_Ar with aminoheterocycles. C) State‐of‐the‐art of primary sulfonamide modification: electrophiles versus nucleophiles as coupling partners.

Recently, our group started a program to selectively activate amino groups by means of pyrylium salts.^9^ Along these lines, we have reported on the synthesis of a simple and practical reagent (Pyry‐BF_4_; **1**) and its high reactivity towards NH_2_ groups in heteroaromatic amines, thus priming them for S_N_Ar reaction to forge a plethora of new carbon–heteroatom bonds (Figure 2 B). Based on this precedent, we envisaged that this simple reagent could engage with primary sulfonamides in a similar fashion and hence, prime the amino group for nucleophilic substitution. Recently, Fier and Maloney reported an elegant method to convert primary sulfonamides into the corresponding sulfinate salt under N‐heterocyclic carbene catalysis (Figure 2 C, left).^10^ This work permits the late‐stage modification of complex primary sulfonamides in a practical and straightforward manner. Despite the tremendous power of this transformation, the subsequent modification of the sulfinate is restricted to the use of electrophiles, which could potentially be a hurdle when either complex amines or alcohols are the desired coupling moiety. Therefore, we envisaged that a complementary strategy based on an umpolung reactivity, where the sulfonamide becomes the electrophile and reacts selectively with a nucleophile, would be highly versatile and desirable. Herein we report on the ability of Pyry‐BF_4_ in combination with simple and cheap MgCl_2_ towards the activation of poorly nucleophilic primary sulfonamides and the subsequent formation of the corresponding sulfonyl chloride (Figure 2 C, right). The mild reaction conditions and the high chemoselectivity permit the formation of sulfonyl chlorides in densely functionalized molecules which contain a wide variety of sensible functionalities. With this method, a plethora of nucleophiles including drugs and agrochemicals could efficiently be coupled in a late‐stage functionalization fashion, thus and expanding the palette of opportunities for drug discovery.

To explore the reactivity of **1** with primary sulfonamides, tosylsulfonamide (**2**) was chosen as a model substrate (Table 1 A). An initial reaction between **1** and **2** in ^*t*^BuOH revealed the formation of the corresponding sulfonic acid **3** in quantitative yields. We envisaged that such interesting hydration could proceed through an initial condensation of **1** and the amino group of **2**, with concomitant formation of one molecule of H_2_O. The activated sulfonyl group is hence reactive enough to permit the attack of H_2_O to finally deliver the sulfonic acid. At this point, we speculated that the presence of a mildly nucleophilic chloride source could engage with the sulfonyl intermediate and override the formation of the sulfonic acid. Indeed, if the deaminative reaction is performed in the presence of MgCl_2_, quantitative yields of *p*‐toluensulfonyl chloride (**4**) were obtained (Table 1 A). The simplicity of the reaction setup is exemplified by carrying the reaction without any precautions for air‐exclusion or water sensitivity. Having a practical protocol in hand, we explored the effect of the electronics and sterics on the sulfonamide moiety both in the hydration reaction and in the formation of sulfonyl chlorides (Table 1 B). As shown, *ortho*, *meta*, and *para* substitution did not affect the reactivity and delivered the sulfonyl chloride and the acid in almost quantitative yields in all cases (**5**–**12**). The presence of electron‐deficient groups in the aryl ring of the sulfonamide, afforded lower yields both of sulfonic acid and sulfonyl chloride (**5** and **6**), suggesting that the lone pair of the NH_2_ group in these cases is even less nucleophilic and condensation with **1** is slower. We next turned our attention to the use of simple alkyl sulfonamides. In this case, smooth conversion into the corresponding acid or sulfonyl chloride was obtained independent of the sterics and electronics on the alkyl groups (**13**–**18**). In an attempt to trap the intermediate of the condensation, *tert*‐butylsulfonamide was reacted with **1** in the absence of MgCl_2_ in CD_3_OD. Indeed, a 65 % of the sulfonate product **19** was obtained after 3 hours of reaction, thus highlighting the ability of mildly nucleophilic oxygenated compounds to outcompete sulfonic acid formation. Although the use of a primary alcohol as solvent affords the corresponding sulfonate, attempts to reduce the amount of ROH in solution resulted in almost exclusive formation of sulfonic acid.^11^

**Table 1:** A) Initial discovery. B) Scope of the conversion of primary sulfonamides into sulfonyl chlorides, sulfonic acids, and sulfonates.

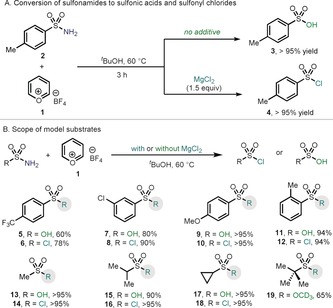

Reaction conditions: sulfonamide (1.00 equiv), Pyry‐BF_4_ (1.10–1.30 equiv), with or without MgCl_2_ (2.05 equiv) in ^*t*^BuOH at 60 °C for 3 h. [a] Reaction carried out in CD_3_OD without MgCl_2_.

The use of simple reagents and mild reaction conditions to form sulfonyl chlorides led us to hypothesize that a wide variety of functional groups could potentially be tolerated. In this sense, we speculated that this protocol could serve as an excellent tool for providing sulfonyl chlorides in late‐stage functionalization contexts were functional‐group compatibility is essential. To this end, we explored a variety of complex druglike derivatives bearing primary sulfonamides in their structures (Table 2). Gratifyingly, the pyrazole derivative celecoxib was smoothly converted into the corresponding sulfonyl chloride in 77 % yield upon isolation after 3 hours at 60 °C (**20**). A sulfonamide bearing a trifluoromethyl ketone and an amide in their structure smoothly reacted to afford 77 % of sulfonyl chloride (**21**). Despite the presence of a secondary amine, an ester and furan, furosemide could successfully be modified and high yields of the sulfonyl chloride were obtained (**22**). The highly chelating precursor to glibenclamide (diabetes type‐II) also afforded good yields (**23**). A sulfonamido‐urea derivative bearing an aryl bromide smoothly reacted under the optimized conditions (76 %, **24**). Compounds bearing the medicinally relevant trifluoromethoxy group (OCF_3_) were also amenable for sulfonyl chloride formation (**25**). The protocol boded well with the presence of unprotected alcohols as exemplified by the good yields obtained of compound **26** (83 %). Gratifyingly, free carboxylic acids were also compatible with these reaction conditions, affording 69 % of the sulfonyl chloride **27**. N‐Boc‐protected anilines also posed no difficulties in forging the sulfonyl chloride (62 %, **28**). Oryzalin, a microtubule disruptor herbicide bearing a tertiary amine, was smoothly converted from the sulfamino group into the corresponding sulfonyl chloride (85 %, **29**). Alkyl sulfonamides were also amenable to sulfonyl chloride formation as exemplified by (1*S*)‐10‐camphor sulfonamide, which could successfully be converted into the corresponding sulfonyl chloride (**30**). Not surprisingly, when the sulfonamide compound bears an unprotected amine in their structure, competition for condensation with **1** occurs, thus preventing sulfonyl chloride formation (**31**). This result highlights one of the main limitations of this protocol. It is important to mention that the mild reaction conditions of this method for the synthesis of sulfonyl chlorides from sulfonamides are in stark contrast to the methods reported utilizing POCl_3_ and sulfonic acids as starting materials. As a result, the reactions are plagued with side reactions resulting in low‐selectivities and limited functional‐group tolerance. For example, the use of POCl_3_ in the presence of simple amides, lactames or ureas leads to Vilsmeyer acyl chloride derivatives and subsequent side‐reactions occur.^12^

**Table 2:** Late‐stage conversion of druglike compounds bearing primary sulfonamides into sulfonyl chlorides. 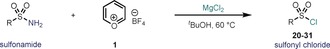

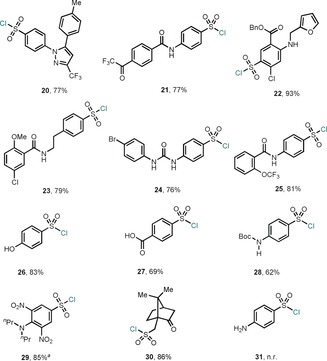

Reaction conditions: Sulfonamide (1.00 equiv), **1** (2.00 equiv), MgCl_2_ (2.55 equiv), and ^*t*^BuOH (0.1 m) at 60 °C for 3–5 h. Yields are those of isolated products. [a] Additional 2.00 equiv of **1** were added after 3 h.

Having established a protocol for synthesizing highly versatile and electrophilic sulfonyl chlorides from various complex molecules, selective diversification of these entities is within reach in a facile and straightforward fashion. With the importance of complex sulfonamide linkages in drug discovery,^2^, ^3^ we initially investigated the possibility of forging sulfonamide bonds, capitalizing on the high reactivity and facile coupling of sulfonyl chlorides with amines (Table 3). The biologically active furosemide could be rapidly converted into the corresponding sulfonyl chloride, and further modified with complex nucleophiles in a simple one‐pot operation (**32**–**35**). To provide medicinally relevant examples, we decided to explore even more complex nucleophiles as potential coupling partners in sulfonamide or sulfonate bond formation. To this end, we turned our attention to biologically active compounds bearing various functional groups embedded in their structure. In this sense, furosemide could be coupled with the polycyclic antidepressant amoxapine in excellent yields (**36**). Celecoxib was also successfully coupled with a variety of primary and secondary amines, affording excellent yields of the corresponding sulfonamides over two steps (**37**–**42**). Pharmacologically relevant and highly functionalized molecules such as paroxetine bearing a benzodioxole (86 %, **43**) or sitagliptin bearing two Lewis‐basic nitrogen atoms (70 %, **44**) were both amenable for sulfonamide linkage with celecoxib. When the glibenclamide precursor was subjected to coupling with warfarin, an anticoagulant drug, a 67 % yield of the sulfonate linkage was obtained (**45**). Similarly, the sulfonamide linkage between the glibenclamide precursor and duloxetine formed in a 75 % yield (**46**). Other complex primary sulfonamides were also compatible in the sulfonamide bond formation by this simple two‐step process and through the parent sulfonyl chloride as exemplified by **47**–**49**. It is important to mention that the yields reported in Table 3 are over two‐steps, starting from the corresponding primary sulfonamide. As mentioned in Table 1, when the reaction of a primary sulfonamide and **1** was carried out in the presence of a primary or secondary alcohol, a sulfonate linkage was obtained. Capitalizing on this reactivity, the complex antipsychotic (±)‐sulpiride, bearing a tertiary amine, reacted similarly and afforded excellent yields of methyl sulfonate (90 %, **50**). In a similar manner, the diuretic and anti‐edema hydrochlorothiazide bearing a cyclic secondary sulfonamide and a secondary amine in their core posed no difficulties for obtaining the ethyl sulfonate when the reaction was carried in EtOH (73 %, **51**).

**Table 3:** Scope of the pyrylium‐medaited sulfonamide coupling. 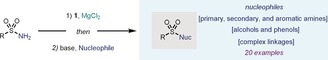

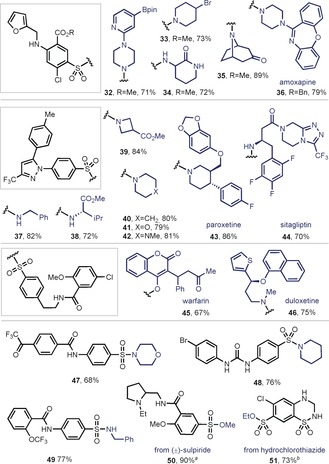

Reaction conditions for step 1: Sulfonamide (1.00 equiv), **1** (2.00 equiv), MgCl_2_ (2.55 equiv), and ^*t*^BuOH (0.1 m) at 60 °C for 3–5 h. Reaction conditions for step 2. Nucleophile (1.5 equiv), and Et_3_N (2 equiv) in either MeCN or CH_2_Cl_2_. See the Supporting Information for specific details. Yields of isolated product are provided. [a] Yield after step 1 without MgCl_2_ in MeOH as solvent. [b] Yield after step 1 without MgCl_2_ in EtOH as solvent.

Having established the facile formation of SO_2_−NR_2_ and SO_2_−OR bonds, we intended to extend the protocol to other desirable linkages derived from complex primary sulfonamides, namely S−P bonds and S−F bonds. In the former, we capitalized on the report by Li and Zhang,^13^ where sulfonyl chlorides were engaged in a reductive phosphorylation. Indeed, when benzylated furosemide was subjected to our sulfonyl chloride synthesis followed by Zhang's protocol, excellent yields of S−P bond formation were obtained (Figure 3 A, **52**). In a similar way, the conversion of sulfonyl chlorides into more robust sulfonyl fluorides has recently attracted a lot of attention for its tremendous properties as click reagents.^14^ When the glibenclamide precursor was subjected to the two‐step operation with potassium hydrogen difluoride,4d, 15 smooth conversion into the corresponding sulfonyl fluoride was obtained (Figure 3 A, **53**). To further illustrate the robustness of the protocol, we scaled‐up our sulfonyl chloride synthesis using benzyl furosemide as starting material. Without modification of the original protocol, 1 gram of benzyl furosemide could successfully be converted into **22** without noticeable erosion in yield (85 %, Figure 3 B).

**Figure 3:**
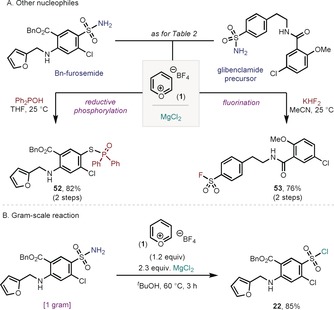
A) Derivatization of complex sulfonamides through the parent sulfonyl chloride. See the Supporting Information for conditions in each particular case. B) Formation of the sulfonyl chloride on gram scale.

In conclusion, we report a simple, practical, robust, and efficient method for the synthesis of sulfonyl chlorides from primary sulfonamides. The reaction capitalizes on the use of a simple pyryilium reagent (Pyry‐BF_4_, **1**) as an activating agent and MgCl_2_ as the chloride source. The mild reaction conditions and the tolerance to a wide variety of functional groups permit the derivatization of complex sulfonamides, whose diversification would be troublesome with other methods available. This report also highlights the simplicity of stitching together molecules through the venerable and “old‐school” sulfonamide coupling without requiring complicated setups and tedious work‐arounds. The method reported herein intends to provide a platform for rapid derivatization of libraries of complex primary sulfonamides which are currently conceived as an unreactive end‐point. With this method, those compounds could be revisited and repurposed to implement further complexity. We strongly believe that the combined practicality and robustness of this method will find direct applications in the chemical community.

## Conflict of interest

The authors declare no conflict of interest.

## Supporting information

As a service to our authors and readers, this journal provides supporting information supplied by the authors. Such materials are peer reviewed and may be re‐organized for online delivery, but are not copy‐edited or typeset. Technical support issues arising from supporting information (other than missing files) should be addressed to the authors.

Supplementary
