# Supplementary material for: Selective Late‐Stage Sulfonyl Chloride Formation from Sulfonamides Enabled by Pyry‐BF4
Source: Angew Chem Int Ed Engl. 2019 Oct 28;58(50):18235–9. doi: 10.1002/anie.201910895 (PMC6916363; doi:10.1002/anie.201910895)
Supplement: Supplementary file 1 — Supplementary [file ANIE-58-18235-s001.pdf]

Angewandte Chemie
Eine Zeitschrift der Gesellschaft Deutscher Chemiker

Supporting Information

Selective Late-Stage Sulfonyl Chloride Formation from Sulfonamides Enabled by Pyry-BF_4

*Alejandro Gómez-Palomino and Josep Cornella**

anie_201910895_sm_miscellaneous_information.pdf

Contents

1. General Experimental Considerations S1
2. Preparation of Pyrylium tetrafluoroborate (Pyry-BF_4) Reagent (**1**) S1
3. General Procedures S3
   3.1. General Procedure A: activation of model substrates S3
   3.2. General Procedure B: activation of complex substrates S4
   3.3. General Procedure C: pyrylium-mediated sulfonamide coupling S4
4. Characterization of Products S4
5. Spectra for Described Compounds S24

1. General Experimental Considerations

Unless otherwise stated, all manipulations were performed using standard experimental techniques without any specific precautions. Anhydrous solvents were distilled from appropriate drying agents and were transferred under Argon: ^{t}BuOH, CH_3CN, EtOH, MeOH (CaH_2, MS).

Pyridine sulfur trioxide complex was purchased from ©TCI Deutschland GmbH, transferred into a Schlenk tube and stored in the fridge under Ar for not longer than 1 week. Prior to use the Pyridine Sulfur Trioxide complex was finely grounded with a pestle and used immediately.

Thin layer chromatography (TLC): Macherey-Nagel precoated plates (POLYGRAM®SIL/UV254). Flash chromatography: Merck silica gel 60 (40-63 μm).

NMR spectra were recorded using a Bruker Avance VIII-300 or Bruker Avance III HD 400 MHz spectrometer in the solvents indicated, chemical shifts (δ) are given in ppm and coupling constants (*J*) provided in Hz. ^{1}H NMR spectra (400.1 MHz) were referenced to the residual protons of the deuterated solvent used. ^{13}C NMR spectra (101 MHz) were referenced internally to the D-coupled 13C resonances of the NMR solvent. ^{19}F NMR spectra (225 MHz) were referenced externally to the 19F resonances of $CFCl_3$.

MS (EI): Finnigan MAT 8200 (70 eV), ESI-MS: ESQ 3000 (Bruker). Accurate mass determinations: Bruker APEX III FT-MS (7 T magnet) or MAT 95 (Finnigan).

IR: Spectrum One (Perkin-Elmer) spectrometer, wavenumbers ($\tilde{\nu}$) in cm-1.

Melting points were recorded on a Büchi® melting point apparatus, Model B-540 (Büchi, Switzerland) and are uncorrected.

2. Preparation of Pyrylium tetrafluoroborate (Pyry-BF$_4$) Reagent (1)

2.1. Preparation of Glutacon Dialdehyde Potassium Salt

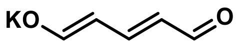

Sulfur trioxide pyridine complex (25.00 g, 157.1 mmol, 1 equiv.) was slowly added in portions to a rigorously stirred solution of KOH (50.2 g, 895.3 mmol, 5.7 equiv.) in water (93 mL) at -20 °C, forming a thick orange slurry. After 1 hour, the temperature was slowly raised to 20 °C over 4 hours, heated at 40 °C for 30 minutes and cooled down to 5°C again. The insoluble crude product was filtered, washed with ice-cold acetone (2 × 50 mL) and MTBE (2 × 50 mL), and dried under vacuum to give dark orange-green crystals.

This material was heated to reflux in methanol (800-1000 mL) and the insoluble solid was filtered out in hot. The clear filtrate was concentrated under reduced pressure to a volume of approximately 50-100 mL and the appearing yellow crystals are collected by filtration, washed with ice-cold acetone (2 × 50 mL) and MTBE (2 × 50 mL), and dried under vacuum to yield the title compound as a yellow solid (13.91 g, 102.1 mmol, 65%, Purity by ISTD-NMR: >99%).

CAS No. 40418-44-8

^{1}H NMR (400 MHz, DMSO-d_6) δ 8.67 (d, *J* = 9.1 Hz, 2H), 7.04 (t, *J* = 13.0 Hz, 1H), 5.10 (dd, *J* = 13.1, 9.2 Hz, 2H).
^{13}C NMR (101 MHz, DMSO-d_6) δ 184.4, 159.8, 106.2.
HRMS (ESI): calcd. for $C_5H_5O_2$ $[M–K^+]^-$: 97.029505; found: 97.029550.

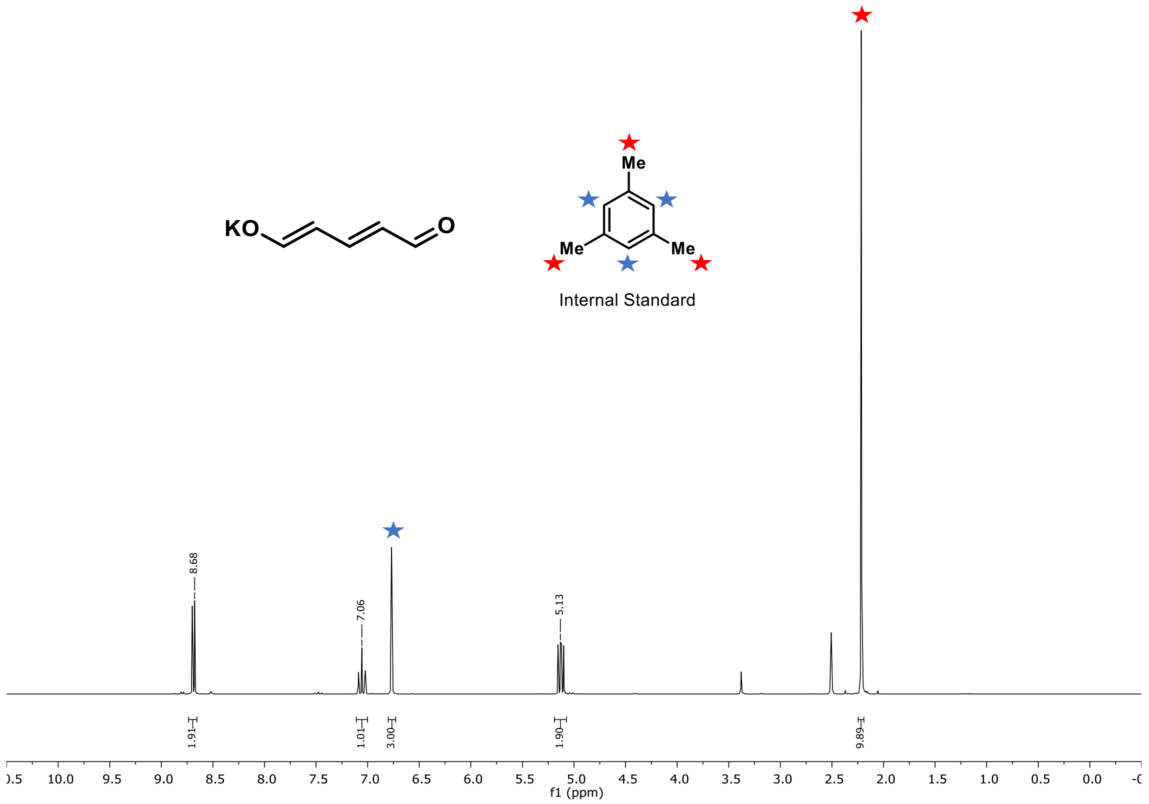

2.2. Preparation of Pyrylium tetrafluoroborate (Pyry-BF4) (1)

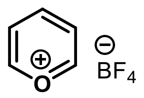

1

***Note**: The reaction was carried out in a flame-dried three-necked round-bottomed flask carefully covered with aluminium foil. The reaction was performed in the dark to avoid polymerization.*

A vigorously stirred suspension of glutacon dialdehyde potassium salt (3.00 g, 22 mmol, 1.0 equiv.) in Et_2O (50 mL) was cooled down to -20 °C under argon atmosphere in the dark. To this mixture, precooled $HBF_4·OEt_2$ (at -20°C, 20 mL, 147 mmol, 6.7 eq) was added in one portion. The mixture was stirred for 16 hours, allowing it to warm up to room temperature slowly. Additional Et_2O (100 mL) was added and stirred for 1 additional hour at room temperature. Then, the mixture was cooled to -20 °C to afford a brown bottom layer of thick slurry and the supernatant solvent was removed. Then, the brown thick slurry was vigorously stirred with more dry Et_2O (50 mL) at room temperature for 5 min, cooled to -20 °C and the supernatant solvent was removed again. This process was repeated a total of three times.

Finally, CH_3CN (Total volume: 120 mL) was added to the remaining fraction, stirred vigorously (ultrasound if needed to dissolve), and filtered under argon obtaining a dark red-brown solution. To this solution, dry Et_2O (300 mL) was added dropwise under argon with an addition funnel (2-3 drops per second, gently stirring of the mixture) appearing a fine precipitate. The mixture was then allowed to stir for additional 10 minutes and the solid was then filtered under argon, washed with Et_2O (2 × 20 mL) and dried under vacuum

to yield the title compound as an analytically pure, off-white to clear-brown solid (2.77 g, 16.5 mmol, 75%, Purity by ISTD-NMR: >99%).

^{1}H NMR (300 MHz, CD_3CN) δ 9.58 (dt, *J* = 3.6, 1.8 Hz, 2H), 9.19 (tt, *J* = 8.1, 1.8 Hz, 1H), 8.37 (ddd, *J* = 8.1, 3.6, 1.5 Hz, 2H).
^{13}C NMR (101 MHz, CD_3CN) δ 169.3, 161.2, 127.7.
^{19}F NMR (282 MHz, CD_3CN) δ -151.7 (s, 1F), -151.8 (s, 3F).
^{11}B NMR (128 MHz, CD_3CN) δ -1.17.
HRMS (ESI): calcd. for $C_5H_5O_1$ $[M–BF_4]^+$ 81.033490; found 81.033520.

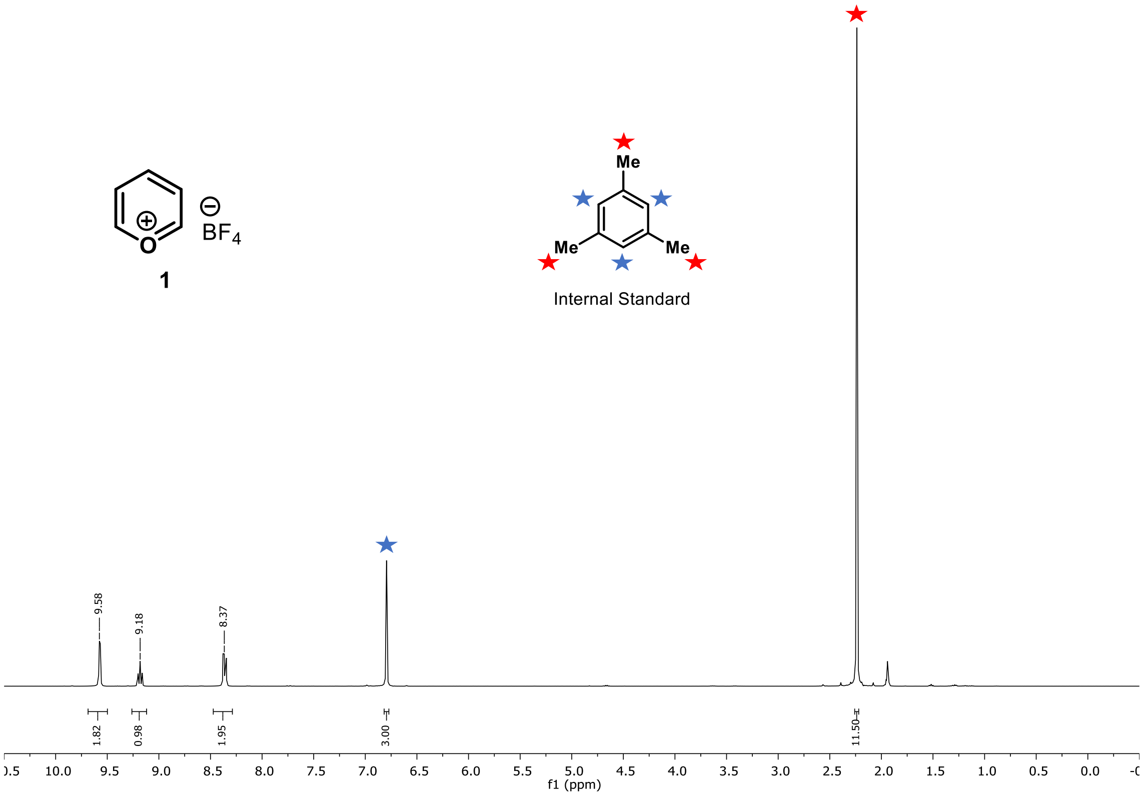

3. General Procedures

3.1. General Procedure A: activation of model substrates

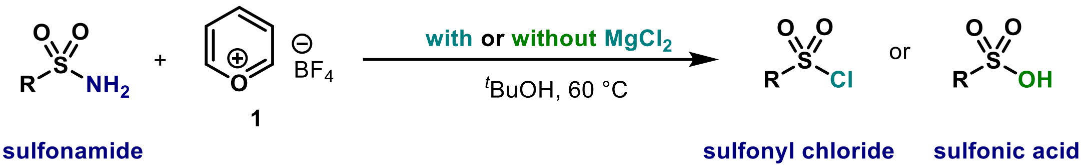

Method A1: without $MgCl_2$

A suspension of sulfonamide (1.0 equiv.) and Pyry-BF_4 (1.1) in *^t^*BuOH (0.1 M) was stirred for 5 min before it was subjected to a preheated oil-bath and stirred for 3 hours at 60 °C. The reaction mixture was then allowed to cool down while being stirred. NMR yields were calculated by using mesitylene (1.0 equiv) as Internal Standard.

Method A2: with $MgCl_2$

A suspension of sulfonamide (1.00 equiv.), Pyry-BF_4 (1.30 equiv.) and $MgCl_2$ (2.05 equiv.) in ^{t}BuOH (0.1 M) was stirred for 5 min before it was subjected to a preheated oil-bath and stirred for 3 hours at 60 °C. The

reaction mixture was then allowed to cool down while being stirred. NMR yields were calculated by using mesitylene (1.00 equiv) as Internal Standard.

3.2. General Procedure B: activation of complex substrates

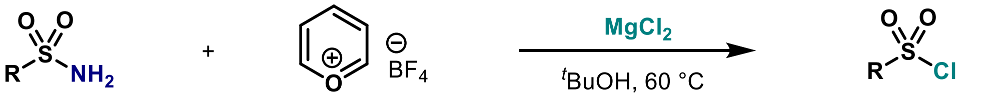

A suspension of sulfonamide (1.00 equiv.), Pyry-BF_4 (2.00 equiv.) and $MgCl_2$ (2.55 equiv.) in ^{t}BuOH (0.1 M) was stirred for 5 min before it was subjected to a preheated oil-bath and stirred for 3-5 hours at 60 °C. The reaction mixture was then allowed to cool down while being stirred. Then, the mixture was filtered through a short plug of silica and washed with EtOAc and evaporated. Finally, the residue was purified by column chromatography (hexanes/EtOAc) to afford the corresponding sulfonyl chlorides.

3.3. General Procedure C: pyrylium-mediated sulfonamide coupling

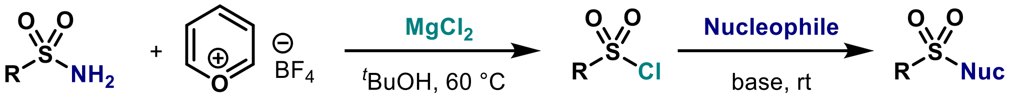

Step 1: a suspension of sulfonamide (1.00 equiv.), Pyry-BF_4 (2.00 equiv.) and $MgCl_2$ (2.55 equiv.) in ^{t}BuOH (0.1 M) was stirred for 5 min before it was subjected to a preheated oil-bath and stirred for 3-5 hours at 60 °C. The reaction mixture was then allowed to cool down while being stirred. Then, the mixture was filtered through a short plug of silica and washed with EtOAc, evaporated.
Step 2: the former residue was dissolved in CH_3CN or CH_2Cl_2 (0.1 M), treated with Et_3N (2.00 equiv.) and the desired nucleophile (1.50 equiv.) at rt for 1 h. Then, the solvent was removed, and the residue was purified by column chromatography (hexanes/EtOAc) to afford the corresponding sulfonamides and sulfonates.

4. Characterization of Products

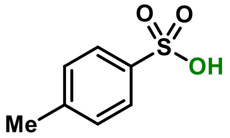

***p*-Toluenesulfonic acid** (**3**)

Following the general procedure A1, **3** was prepared from *p*-toluenesulfonamide (**2**) (17.1 mg, 0.10 mmol, 1.0 equiv.) and Pyry-BF_4 (18.4 mg, 0.11 mmol, 1.1 equiv.). NMR data are in accordance with the literature report.
CAS No. 6192-52-5
^{1}H NMR (400 MHz, $CDCl_3$) δ 7.76 (d, *J* = 8.2 Hz, 2H), 7.21 (d, *J* = 8.2 Hz, 2H), 2.37 (s, 3H).

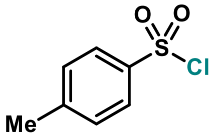

***p*-Toluenesulfonyl chloride** (**4**)

Following the general procedure A2, **4** was prepared from *p*-toluenesulfonamide (**2**) (17.1 mg, 0.100 mmol, 1.00 equiv.), Pyry-BF_4 (21.8 mg, 0.130 mmol, 1.30 equiv.) and $MgCl_2$ (19.5 mg, 0.205 mmol, 2.05 equiv.). NMR data are in accordance with the literature report.
CAS No. 98-59-9
^{1}H NMR (400 MHz, $CDCl_3$) δ 7.93 (d, *J* = 8.5 Hz, 2H), 7.41 (d, *J* = 8.5 Hz, 2H), 2.49 (s, 3H).

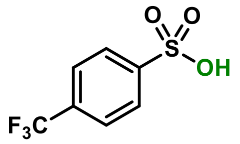

***p*-(Trifluoromethyl)benzenesulfonic acid** (**5**)

Following the general procedure A1, **5** was prepared from *p*-(trifluoromethyl)benzenesulfonamide (22.5 mg, 0.10 mmol, 1.0 equiv.) and Pyry-BF_4 (18.4 mg, 0.11 mmol, 1.1 equiv.). NMR data are in accordance with the literature report.
CAS No. 455-24-3
^{1}H NMR (400 MHz, $CDCl_3$) δ 7.98 (d, *J* = 8.1 Hz, 2H), 7.65 (d, *J* = 8.1 Hz, 2H).
^{19}F NMR (225 MHz, $CDCl_3$) δ -63.2 (s, 3F).

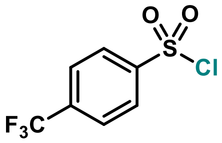

***p*-(Trifluoromethyl)benzenesulfonyl chloride** (**6**)

Following the general procedure A2, **6** was prepared from *p*-(trifluoromethyl)benzenesulfonamide (22.5 mg, 0.100 mmol, 1.00 equiv.), Pyry-BF_4 (21.8 mg, 0.130 mmol, 1.30 equiv.) and $MgCl_2$ (19.5 mg, 0.205 mmol, 2.05 equiv.). NMR data are in accordance with the literature report.
CAS No. 2991-42-6
^{1}H NMR (400 MHz, $CDCl_3$) δ 8.19 (d, *J* = 8.3 Hz, 2H), 7.90 (d, *J* = 8.3 Hz, 2H).
^{19}F NMR (225 MHz, $CDCl_3$) δ -63.4 (s, 3F).

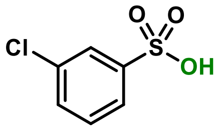

***m*-Chlorobenzenesulfonic acid** (**7**)

Following the general procedure A1, **7** was prepared from *m*-chlorobenzenesulfonamide (19.2 mg, 0.10 mmol, 1.0 equiv.) and Pyry-BF_4 (18.4 mg, 0.11 mmol, 1.1 equiv.). NMR data are in accordance with the literature report.
CAS No. 20677-52-5
^{1}H NMR (400 MHz, $CDCl_3$) δ 7.85 (t, *J* = 1.8 Hz, 1H), 7.75 (dt, *J* = 7.6, 1.5 Hz, 1H), 7.39–7.36 (m, 1H), 7.33 (t, *J* = 7.6 Hz, 1H).

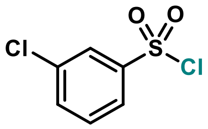

***m*-Chlorobenzenesulfonyl chloride** (**8**)

Following the general procedure A2, **8** was prepared from *m*-chlorobenzenesulfonamide (19.2 mg, 0.100 mmol, 1.00 equiv.), Pyry-BF_4 (21.8 mg, 0.130 mmol, 1.30 equiv.) and $MgCl_2$ (19.5 mg, 0.205 mmol, 2.05 equiv.). NMR data are in accordance with the literature report.
CAS No. 2888-06-4

^{1}H NMR (400 MHz, $CDCl_3$) δ 8.02 (t, J = 1.9 Hz, 1H), 7.93 (ddd, J = 8.0, 1.9, 1.0 Hz, 1H), 7.71 (ddd, J = 8.0, 1.9, 1.0 Hz, 1H), 7.57 (t, J = 8.0 Hz, 1H).

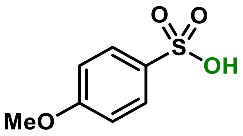

***p*-Methoxybenzenesulfonic acid** (**9**)

Following the general procedure A1, **9** was prepared from *p*-methoxybenzenesulfonamide (18.7 mg, 0.10 mmol, 1.0 equiv.) and Pyry-BF_4 (18.4 mg, 0.11 mmol, 1.1 equiv.). NMR data are in accordance with the literature report.
CAS No. 5857-42-1
^{1}H NMR (400 MHz, $CDCl_3$) δ 7.81 (d, J = 8.8 Hz, 2H), 6.90 (d, J = 8.8 Hz, 2H), 3.82 (s, 3H).

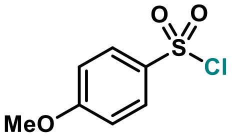

***p*-Methoxybenzenesulfonyl chloride** (**10**)

Following the general procedure A2, **10** was prepared from *p*-methoxybenzenesulfonamide (18.7 mg, 0.100 mmol, 1.00 equiv.), Pyry-BF_4 (21.8 mg, 0.130 mmol, 1.30 equiv.) and $MgCl_2$ (19.5 mg, 0.205 mmol, 2.05 equiv.). NMR data are in accordance with the literature report.
CAS No. 98-68-0
^{1}H NMR (400 MHz, $CDCl_3$) δ 7.97 (d, J = 9.2 Hz, 2H), 7.04 (d, J = 9.2 Hz, 2H), 3.92 (s, 3H).

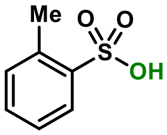

***o*-Toluenesulfonic acid** (**11**)

Following the general procedure A1, **11** was prepared from *o*-toluenesulfonamide (17.1 mg, 0.10 mmol, 1.0 equiv.) and Pyry-BF_4 (18.4 mg, 0.11 mmol, 1.1 equiv.). NMR data are in accordance with the literature report.
CAS No. 88-20-0
^{1}H NMR (400 MHz, $CDCl_3$) δ 7.94 (d, J = 7.7 Hz, 1H), 7.33–7.18 (m, 3H), 2.68 (s, 3H).

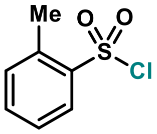

***o*-Toluenesulfonyl chloride** (**12**)

Following the general procedure A2, **12** was prepared from *o*-toluenesulfonamide (17.1 mg, 0.100 mmol, 1.00 equiv.), Pyry-BF_4 (21.8 mg, 0.130 mmol, 1.30 equiv.) and $MgCl_2$ (19.5 mg, 0.205 mmol, 2.05 equiv.). NMR data are in accordance with the literature report.
CAS No. 133-59-5
^{1}H NMR (400 MHz, $CDCl_3$) δ 8.06 (dd, J = 8.6, 1.2 Hz, 1H), 7.99 (dd, J = 7.6, 6.5 Hz,), 7.60 (dt, J = 7.6, 1.2 Hz, 1H), 7.42–7-38 (m, 2H), 2.78 (s, 3H).

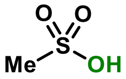

Methanesulfonic acid (**13**)

Following the general procedure A1, **13** was prepared from methanesulfonamide (9.5 mg, 0.10 mmol, 1.0 equiv.) and Pyry-BF_4 (18.4 mg, 0.11 mmol, 1.1 equiv.). NMR data are in accordance with the literature report.
CAS No. 75-75-2
^{1}H NMR (400 MHz, $CDCl_3$) δ 2.87 (s, 3H).

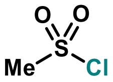

Methanesulfonyl chloride (**14**)

Following the general procedure A2, **14** was prepared from methanesulfonamide (9.5 mg, 0.100 mmol, 1.00 equiv.), Pyry-BF_4 (21.8 mg, 0.130 mmol, 1.30 equiv.) and $MgCl_2$ (19.5 mg, 0.205 mmol, 2.05 equiv.). NMR data are in accordance with the literature report.
CAS No. 124-63-0
^{1}H NMR (400 MHz, $CDCl_3$) δ 3.67 (s, 3H).

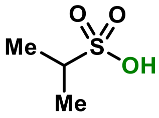

Propane-2-sulfonic acid (**15**)

Following the general procedure A1, **15** was prepared from propane-2-sulfonamide (12.3 mg, 0.10 mmol, 1.0 equiv.) and Pyry-BF_4 (18.4 mg, 0.11 mmol, 1.1 equiv.). NMR data are in accordance with the literature report.
CAS No. 14159-48-9
^{1}H NMR (400 MHz, $CDCl_3$) δ 3.09 (hept, J = 7.1 Hz, 1H), 1.36 (d, J = 7.1 Hz, 6H).

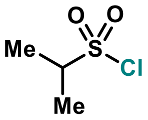

Propane-2-sulfonyl chloride (**16**)

Following the general procedure A2, **16** was prepared from propane-2-sulfonamide (12.3 mg, 0.100 mmol, 1.00 equiv.), Pyry-BF_4 (21.8 mg, 0.130 mmol, 1.30 equiv.) and $MgCl_2$ (19.5 mg, 0.205 mmol, 2.05 equiv.). NMR data are in accordance with the literature report.
CAS No. 10147-37-2
^{1}H NMR (400 MHz, $CDCl_3$) δ 3.76 (hept, J = 6.8 Hz, 1H), 1.61 (d, J = 6.8 Hz, 6H).

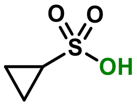

Cyclopropanesulfonic acid (**17**)

Following the general procedure A1, **17** was prepared from propane-2-sulfonamide (12.1 mg, 0.10 mmol, 1.0 equiv.) and Pyry-BF_4 (18.4 mg, 0.11 mmol, 1.1 equiv.). NMR data are in accordance with the literature report.
CAS No. 21297-68-7
^{1}H NMR (400 MHz, $CDCl_3$) δ 2.44–2.36 (m, 1H), 1.14–1.11 (m, 2H), 0.93–0.89 (m, 2H).

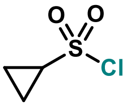

Cyclopropanesulfonyl chloride (**18**)

Following the general procedure A2, **18** was prepared from propane-2-sulfonamide (12.1 mg, 0.100 mmol, 1.00 equiv.), Pyry-BF_4 (21.8 mg, 0.130 mmol, 1.30 equiv.) and $MgCl_2$ (19.5 mg, 0.205 mmol, 2.05 equiv.). NMR data are in accordance with the literature report.
CAS No. 139631-62-2
^{1}H NMR (400 MHz, $CDCl_3$) δ 3.27 (tt, *J* = 7.9, 4.6 Hz, 1H), 1.37–1.32 (m, 4H).

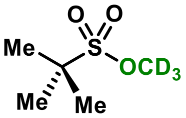

Methyl-*d*$_3$ 2-methylpropane-2-sulfonate (**19**)

Following the general procedure A1, **18** was prepared from 2-methylpropane-2-sulfonamide (13.7 mg, 0.100 mmol, 1.00 equiv.) and Pyry-BF_4 (21.8 mg, 0.130 mmol, 1.30 equiv.) in CD_3OD.
^{1}H NMR (400 MHz, CD_3OD-*d*$_4$) δ 1.20 (s, 9H).

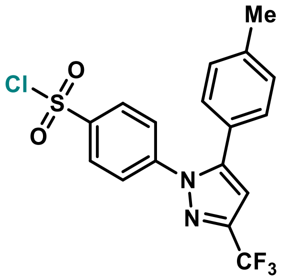

Celecoxib chloride (**20**)

Following the general procedure B, **20** was prepared from celecoxib (38.1 mg, 0.100 mmol, 1.00 equiv.), Pyry-BF_4 (33.6 mg, 0.200 mmol, 2.00 equiv.) and $MgCl_2$ (24.3 mg, 0.255 mmol, 2.55 equiv.) for 3 h. The product was purified by column chromatography on silica gel (hexanes/EtOAc 8:2) to yield the title compound (30.7 mg, 0.077 mmol, 77% yield) as a yellowish wax.
^{1}H NMR (400 MHz, $CDCl_3$) δ 8.02 (d, *J* = 8.9 Hz, 2H), 7.59 (d, *J* = 8.9 Hz, 2H), 7.22 (d, *J* = 7.9 Hz, 2H), 7.14 (d, *J* = 7.9 Hz, 2H), 6.76 (s, 1H), 2.41 (s, 3H).
^{13}C NMR (101 MHz, $CDCl_3$) δ 145.6, 144,8 (q, *J* = 38.76 Hz), 144.6, 143.1, 140.3, 130.1, 128.9, 128.3, 125.7, 125.6, 121.0 (q, *J* = 269.4 Hz), 107.1, 21.5.
^{19}F NMR (225 MHz, $CDCl_3$) δ -62.6 (s, 3F).
HRMS (ESI): calcd. for $C_{17}H_{13}{}^{35}ClF_3N_2O_2S$ $[M+H]^+$: 401.033288; found: 401.033160; calcd. for $C_{17}H_{13}{}^{37}ClF_3N_2O_2S$ $[M+H]^+$: 403.030332; found: 403.030080.

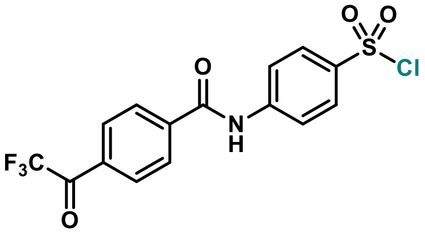

4-(4-(Trifluoroacetyl)benzamido)benzenesulfonyl chloride (**21**)

Following the general procedure B, **21** was prepared from *N*-(4-sulfamoylphenyl)-4-(2,2,2-trifluoroacetyl)benzamide (37.2 mg, 0.100 mmol, 1.00 equiv.), Pyry-BF_4 (33.6 mg, 0.200 mmol, 2.00 equiv.) and $MgCl_2$ (24.3 mg, 0.255 mmol, 2.55 equiv.) for 5 h. The product was purified by column chromatography on silica gel (hexanes/EtOAc 6:4) to yield the title compound (30.4 mg, 0.077 mmol, 77% yield) as a yellowish wax.
^{1}H NMR (400 MHz, $CDCl_3$) δ 8.23 (d, *J* = 7.7 Hz, 2H), 8.14 (br s, 1H), 8.08 (d, *J* = 7.7 Hz, 2H), 8.06 (d, *J* = 7.3 Hz, 2H), 7.95 (d, *J* = 7.3 Hz, 2H).

^{13}C NMR (101 MHz, $CDCl_3$) δ 164.4, 143.7, 139.9, 139.8, 133.0, 130.9 (q, *J* = 2.0 Hz), 129.0, 128.0, 120.2.
^{19}F NMR (225 MHz, $CDCl_3$) δ -71.9 (s, 3F).
HRMS (ESI): calcd. for $C_{15}H_8{}^{35}ClF_3NO_4S$ [M–H]$^-$: 389.982020; found: 389.981940; calcd. for $C_{15}H_8{}^{37}ClF_3NO_4S$ [M–H]$^-$: 391.979063; found: 391.978950.

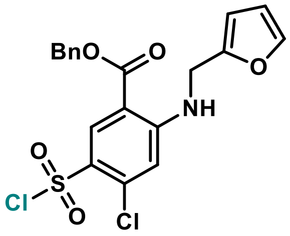

Benzyl furosemide chloride (**22**)

Following the general procedure B, **22** was prepared from benzyl furosemide (42.1 mg, 0.100 mmol, 1.00 equiv.), Pyry-BF_4 (33.6 mg, 0.200 mmol, 2.00 equiv.) and $MgCl_2$ (24.3 mg, 0.255 mmol, 2.55 equiv.) for 5 h. The product was purified by column chromatography on silica gel (hexanes/EtOAc 8:2) to yield the title compound (40.8 mg, 0.093 mmol, 93% yield) as a yellow wax.
^{1}H NMR (400 MHz, $CDCl_3$) δ 8.96 (br s, 1H), 8.70 (s, 1H), 7.44–7.34 (m, 6H), 6.95 (s, 1H), 6.37 (dd, *J* = 3.3, 1.9 Hz, 1H), 6.31 (dd, *J* = 3.3, 0.9 Hz, 1H), 5.34 (s, 2H), 4.48 (d, *J* = 5.6 Hz, 2H).
^{13}C NMR (101 MHz, $CDCl_3$) δ 166.6, 154.4, 149.6, 143.0, 138.8, 135.9, 135.3, 128.9, 128.8, 128.5, 127.5, 114.3, 110.7, 108.4, 108.3, 67.3, 40.4.
HRMS (ESI): calcd. for $C_{19}H_{14}{}^{35}Cl_2NO_5S$ [M–H]$^-$: 437.997528; found: 437.998300; calcd. for $C_{19}H_{14}{}^{37}Cl_2NO_5S$ [M–H]$^-$: 439.994571; found: 439.995340.

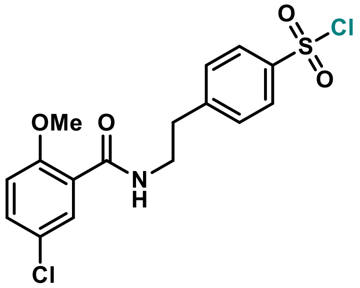

4-(2-(5-Chloro-2-methoxybenzamido)ethyl)benzenesulfonyl chloride (**23**)

Following the general procedure B, **23** was prepared from 5-chloro-2-methoxy-N-(4-sulfamoylphenethyl)benzamide (36.9 mg, 0.100 mmol, 1.00 equiv.), Pyry-BF_4 (33.6 mg, 0.200 mmol, 2.00 equiv.) and $MgCl_2$ (24.3 mg, 0.255 mmol, 2.55 equiv.) for 3 h. The product was purified by column chromatography on silica gel (hexanes/EtOAc 4:6) to yield the title compound (30.6 mg, 0.079 mmol, 79% yield) as a yellowish wax.
^{1}H NMR (400 MHz, $CDCl_3$) δ 8.17 (d, *J* = 2.8 Hz, 1H), 8.00 (d, *J* = 8.5 Hz, 2H), 7.81 (br s, 1H), 7.5 (d, *J* = 8.5 Hz, 2H), 7.39 (dd, *J* = 8.8, 2.8 Hz, 1H), 6.89 (d, *J* = 8.8 Hz, 1H), 3.81 (s, 3H), 3.77 (q, *J* = 6.9 Hz, 2H), 3.08 (t, *J* = 6.9 Hz, 2H).
^{13}C NMR (101 MHz, $CDCl_3$) δ 164.3, 156.0, 148.1, 142.8, 132.7, 132.2, 130.3, 127.4, 127.1, 122.7, 113.0, 56.4, 40.7, 35.9.
HRMS (ESI): calcd. for $C_{16}H_{15}{}^{35}Cl_2NO_4SNa$ [M+H]$^+$: 409.999106; found: 409.999090; calcd. for $C_{16}H_{15}{}^{35}Cl^{37}ClNO_4SNa$ [M+H]$^+$: 411.996150; found: 411.996170; calcd. for $C_{16}H_{15}{}^{37}Cl_2NO_4SNa$ [M+H]$^+$: 413.993194; found: 413.993360.

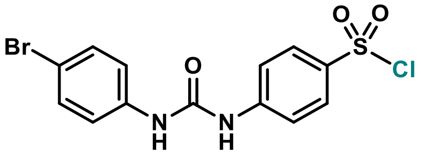

4-(3-(4-Bromophenyl)ureido)benzenesulfonyl chloride (**24**)

Following the general procedure B, **24** was prepared from 4-(3-(4-bromophenyl)ureido)benzenesulfonamide (37.0 mg, 0.100 mmol, 1.00 equiv.), Pyry-BF_4 (33.6 mg, 0.200 mmol, 2.00 equiv.) and $MgCl_2$ (24.3 mg, 0.255 mmol, 2.55 equiv.) for 5 h. The product was purified by column chromatography on silica gel (hexanes/EtOAc 4:6) to yield the title compound (29.8 mg, 0.076 mmol, 76% yield) as a yellowish wax.
^{1}H NMR (400 MHz, acetone-*d_6*) δ 8.91 (br s, 1H), 8.50 (br s, 1H), 8.02 (d, *J* = 9.1 Hz, 2H), 7.89 (d, *J* = 9.1 Hz, 2H), 7.53 (d, *J* = 8.9 Hz, 2H), 7.46 (d, *J* = 8.9 Hz, 2H).
^{13}C NMR (101 MHz, acetone-*d_6*) δ 152.6, 147.8, 139.4, 137.2, 132.6, 129.6, 121.7, 119.2, 115.6.
HRMS (ESI): calcd. for $C_{13}H_9{}^{79}Br^{35}ClN_2O_3S$ $[M+Na]^+$: 410.917637; found: 410.917470; calcd. for $C_{13}H_9{}^{79}Br^{37}ClN_2O_3S$ $[M+Na]^+$: 412.914681; found: 412.914490; calcd. for $C_{13}H_9{}^{81}Br^{35}ClN_2O_3S$ $[M+Na]^+$: 412.915631; found: 412.915430; calcd. for $C_{13}H_9{}^{81}Br^{37}ClN_2O_3S$ $[M+Na]^+$: 414.912674; found: 414.912470.

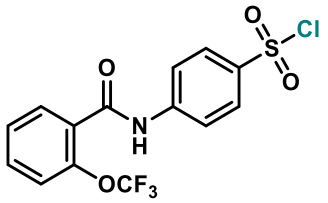

4-(2-(Trifluoromethoxy)benzamido)benzenesulfonyl chloride (**25**)

Following the general procedure B, **25** was prepared from *N*-(4-sulfamoylphenyl)-2-(trifluoromethoxy)benzamide (36.0 mg, 0.100 mmol, 1.00 equiv.), Pyry-BF_4 (33.6 mg, 0.200 mmol, 2.00 equiv.) and $MgCl_2$ (24.3 mg, 0.255 mmol, 2.55 equiv.) for 5 h. The product was purified by column chromatography on silica gel (hexanes/EtOAc 6:4) to yield the title compound (30.6 mg, 0.081 mmol, 81% yield) as a yellow wax.
^{1}H NMR (400 MHz, $CDCl_3$) δ 8.69 (br s, 1H), 8.10 (dd, *J* = 7.8, 1.8 Hz, 1H), 8.04 (d, *J* = 9.0 Hz, 2H), 7.90 (d, *J* = 9.0 Hz, 2H), 7.63 (ddd, *J* = 8.3, 7.5, 1.8 Hz), 7.5 (td, *J* = 7.5, 1.5 Hz, 1H), 7.39 (dt, *J* = 8.3, 1.5 Hz).
^{13}C NMR (101 MHz, $CDCl_3$) δ 162.7, 146.1 (q, *J* = 1.5 Hz), 143.9, 139.5, 133.9, 132.4, 128.9, 128.1, 127.0, 121.4, 120.4 (q, *J* = 261.0 Hz), 120.1.
^{19}F NMR (225 MHz, $CDCl_3$) δ -57.5 (s, 3F).
HRMS (ESI): calcd. for $C_{14}H_8{}^{35}ClF_3NO_4S$ $[M–H]^-$: 377.982020; found: 377.981780; calcd. for $C_{14}H_8{}^{37}ClF_3NO_4S$ $[M–H]^-$: 379.979063; found: 379.978710

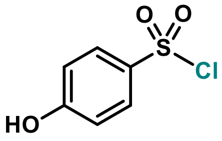

4-Hydroxybenzenesulfonyl chloride (**26**)

Following the general procedure B, **26** was prepared from 4-hydroxybenzenesulfonamide (17.3 mg, 0.100 mmol, 1.00 equiv.), Pyry-BF_4 (33.6 mg, 0.200 mmol, 2.00 equiv.) and $MgCl_2$ (24.3 mg, 0.255 mmol, 2.55 equiv.) for 3 h. The product was purified by column chromatography on silica gel (hexanes/EtOAc 7:3) to yield the title compound (15.9 mg, 0.082 mmol, 82% yield) as a yellowish wax. NMR data are in accordance with the literature report.
CAS No. 4025-67-6
^{1}H NMR (400 MHz, $CDCl_3$) δ 7.98 (d, *J* = 9.0 Hz, 2H), 7.15 (d, *J* = 9.0 Hz, 2H).
^{13}C NMR (101 MHz, $CDCl_3$) δ 164.0, 134.7, 129.9, 116.5.

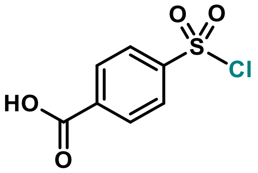

4-(Chlorosulfonyl)benzoic acid (**27**)

Following the general procedure B, **27** was prepared from 4-carboxybenzenesulfonamide (40.2 mg, 0.20 mmol, 2.00 equiv.), Pyry-BF_4 (43.7 mg, 0.26 mmol, 1.30 equiv.) and $MgCl_2$ (48.9 mg, 0.51 mmol, 2.55 equiv.) for 3 h. The product was purified by column chromatography on silica gel (hexanes/EtOAc 7:3 + 1% AcOH) to yield the title compound (30.8 mg, 0.14 mmol, 69% yield) as a yellowish wax. NMR data are in accordance with the literature report.
CAS No. 10130-89-9
^{1}H NMR (400 MHz, acetone-d_6) δ 8.38 (d, J = 8.8 Hz, 2H), 8.28 (d, J = 8.8 Hz, 2H).
^{13}C NMR (101 MHz, acetone-d_6) δ 166.7, 147.9, 132.1, 128.1.
HRMS (ESI): calcd. for $C_7H_4{}^{35}ClO_4S$ $[M–H]^–$: 218.952436; found: 218.952400; calcd. for $C_7H_4{}^{37}ClO_4S$ $[M–H]^–$: 220.949479; found: 220.949450.

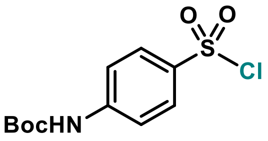

^{t}Butyl (4-(chlorosulfonyl)phenyl)carbamate (**28**)

Following the general procedure B, **28** was prepared from ^{t}butyl (4-(chlorosulfonyl)phenyl)carbamate (54.5 mg, 0.20 mmol, 2.00 equiv.), Pyry-BF_4 (43.7 mg, 0.26 mmol, 1.30 equiv.) and $MgCl_2$ (48.9 mg, 0.51 mmol, 2.55 equiv.) for 3 h. The product was purified by column chromatography on silica gel (hexanes/EtOAc 7:3) to yield the title compound (36.4 mg, 0.13 mmol, 62% yield) as a yellowish wax. NMR data are in accordance with the literature report.
CAS No. 10130-89-9
^{1}H NMR (400 MHz, $CDCl_3$) δ 9.06 (br s, 1H), 7.9 (d, J = 9.1 Hz, 2H), 7.78 (d, J = 9.1 Hz, 2H), 1.38 (s, 9H).
^{13}C NMR (101 MHz, $CDCl_3$) δ 153.2, 147.7, 137.4, 129.5, 119.0, 81.6, 28.4.
HRMS (ESI): calcd. for $C_{11}H_{13}{}^{35}ClNO_4S$ $[M–H]^–$: 290.025934; found: 290.026150; calcd. for $C_{11}H_{13}{}^{37}ClNO_4S$ $[M–H]^–$: 292.022978; found: 292.023080.

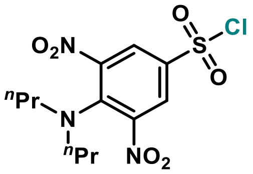

4-(Dipropylamino)-3,5-dinitrobenzenesulfonyl chloride (**29**)

Following the general procedure B, **29** was prepared from 4-(dipropylamino)-3,5-dinitrobenzenesulfonamide (34.6 mg, 0.100 mmol, 1.00 equiv.), Pyry-BF_4 (33.6 mg, 0.200 mmol, 2.00 equiv.) and $MgCl_2$ (24.3 mg, 0.255 mmol, 2.55 equiv.) for 3 h, followed by another addition of Pyry-BF_4 (33.6 mg, 0.200 mmol, 2.00 equiv.) for 2 h. The product was purified by column chromatography on silica gel (hexanes/EtOAc 8:2) to yield the title compound (31.2 mg, 0.085 mmol, 85% yield) as an orange solid.
^{1}H NMR (400 MHz, $CDCl_3$) δ 8.41 (s, 2H), 3.04–3.01 (m, 4H), 1.66 (h, J = 7.4 Hz, 4H), 0.91 (t, J = 7.4 Hz, 6H).
^{13}C NMR (101 MHz, $CDCl_3$) δ 144.0, 143.2, 132.6, 129.0, 54.4, 20.9, 11.3.
HRMS (ESI): calcd. for $C_{12}H_{16}{}^{35}ClN_3O_6S$ $[M]^+$: 365.044287; found: 365.043860; calcd. for $C_{12}H_{16}{}^{37}ClN_3O_6S$ $[M]^+$: 367.041330; found: 367.040720.
M.P.: 110.3-111.5 °C.

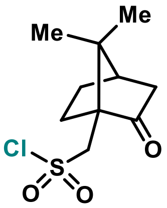

(*S*)-10-Camphorsulfonyl chloride (30)

Following the general procedure B, **30** was prepared from (*S*)-10-camphorsulfonamide (23.1 mg, 0.100 mmol, 1.00 equiv.), Pyry-BF_4 (33.6 mg, 0.200 mmol, 2.00 equiv.) and $MgCl_2$ (24.3 mg, 0.255 mmol, 2.55 equiv.) for 5 h. The product was purified by column chromatography on silica gel (hexanes/EtOAc 8:2) to yield the title compound (21.5 mg, 0.086 mmol, 86% yield) as a yellowish wax. NMR data are in accordance with the literature report.
CAS No. 21286-54-4
^{1}H NMR (400 MHz, $CDCl_3$) δ 4.31 (d, *J* = 14.6 Hz, 1H), 3.72 (d, *J* = 14.6 Hz, 1H), 2.52–2.39 (m, 2H), 2.18–1.96 (m, 3H), 1.77 (ddd, *J* = 13.5, 9.2, 4.5 Hz, 1H), 1.48 (ddd, *J* = 13.5, 9.2, 3.9 Hz, 1H), 1.14 (s, 3H), 0.93 (s, 3H).

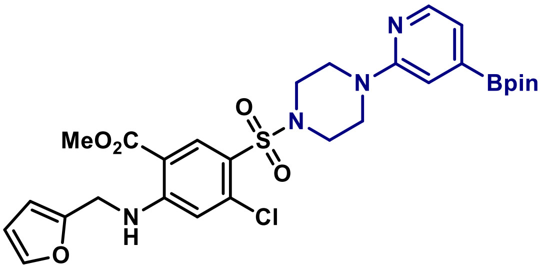

Methyl 4-chloro-2-((furan-2-ylmethyl)amino)-5-((4-(4-(4,4,5,5-tetramethyl-1,3,2-dioxaborolan-2-yl)pyridin-2-yl)piperazin-1-yl)sulfonyl)benzoate (32)

Following the general procedure C, **32** was prepared from methyl furosemide (34.5 mg, 0.100 mmol, 1.00 equiv.), Pyry-BF_4 (33.6 mg, 0.200 mmol, 2.00 equiv.) and $MgCl_2$ (24.3 mg, 0.255 mmol, 2.55 equiv.) for 5 h, followed by Et_3N (28 µL, 0.20 mmol, 2.00 equiv.) and 2-(1-piperazinyl)pyridine-4-boronic acid pinacol ester (43.3 mg, 0.15 mmol, 1.50 equiv.). The product was purified by column chromatography on silica gel (EtOAc) to yield the title compound (43.9 mg, 0.071 mmol, 71% yield) as an off-white solid.
^{1}H NMR (400 MHz, $CDCl_3$) δ 8.63 (br t, *J* = 5.6 Hz, 1H), 8.58 (s, 1H), 8.18 (d, *J* = 4.8 Hz, 1H), 7.39 (s, 1H), 7.01 (s, 1H), 6.97 (d, *J* = 4.8 Hz, 1H), 6.85 (s, 1H), 6.34 (dd, *J* = 3.1, 1.9 Hz, 1H), 6.28 (d, *J* = 3.1 Hz, 1H), 4.43 (d, *J* = 5.6 Hz, 2H), 3.88 (s, 3H), 3.66–3.64 (m, 4H), 3.35–3.33 (m, 4H), 1.33 (s, 12H).
^{13}C NMR (101 MHz, $CDCl_3$) δ 167.7, 158.5, 153.1, 150.3, 147.4, 142.8, 138.4, 136.9, 121.2, 118.7, 114.2, 113.0, 110.7, 108.6, 108.0, 84.5, 52.3, 45.5, 45.3, 40.4, 25.0.
HRMS (ESI): calcd. for $C_{28}H_{35}B^{35}ClN_4O_7S$ $[M]^+$: 617.200255; found: 617.200570; calcd. for $C_{28}H_{35}B^{37}ClN_4O_7S$ $[M]^+$: 619.197299; found: 619.197170.
IR (ATR): 3340, 2984, 2949, 916, 2850, 1693, 1592, 1444, 1348, 1164 cm^{-1}.
M.P.: 183.4–184.6 °C (decomposition possible).

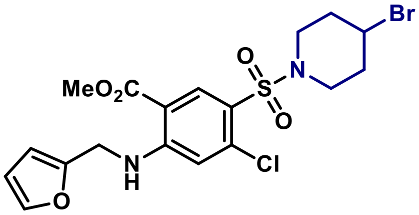

Methyl 5-((4-bromopiperidin-1-yl)sulfonyl)-4-chloro-2-((furan-2-ylmethyl)amino)benzoate (33)
Following the general procedure C, **33** was prepared from methyl furosemide (34.5 mg, 0.100 mmol, 1.00 equiv.), Pyry-BF_4 (33.6 mg, 0.200 mmol, 2.00 equiv.) and $MgCl_2$ (24.3 mg, 0.255 mmol, 2.55 equiv.) for 5 h, followed by Et_3N (28 µL, 0.20 mmol, 2.00 equiv.) and 4-bromo-1l2-piperidine (24.5 mg, 0.15 mmol, 1.50

equiv.). The product was purified by column chromatography on silica gel (hexanes/EtOAc 8:2) to yield the title compound (35.8 mg, 0.073 mmol, 73% yield) as an off-white solid.
^{1}H NMR (400 MHz, $CDCl_3$) δ 8.64 (br t, *J* = 5.5 Hz, 1H), 8.58 (s, 1H), 7.40 (s, 1H), 6.87 (s, 1H), 6.36 (dd, *J* = 3.2, 1.9 Hz, 1H), 6.29 (d, *J* = 3.2 Hz, 1H), 4.44 (d, *J* = 5.5 Hz, 2H), 4.35 (tt, J = 7.1, 3.7 Hz, 1H), 3.88 (s, 3H), 3.50–3.44 (m, 2H), 3.33–3.27 (m, 2H), 2.21–2.15 (m, 2H), 2.07–1.99 (m, 2H).
^{13}C NMR (101 MHz, $CDCl_3$) δ 167.7, 153.1, 150.3, 142.8, 138.4, 136.7, 121.8, 114.1, 110.7, 108.5, 108.0, 52.3, 48.6, 43.5, 40.4, 35.2.
HRMS (ESI): calcd. for $C_{18}H_{20}{}^{79}Br^{35}ClN_2O_5SNa$ $[M+Na]^+$: 512.985717; found: 512.985470; calcd. for $C_{18}H_{20}{}^{79}Br^{37}ClN_2O_5SNa$ $[M+Na]^+$: 514.982761; found: 514.982460; calcd. for $C_{18}H_{20}{}^{91}Br^{35}ClN_2O_5SNa$ $[M+Na]^+$: 514.983711; found: 514.983430; calcd. for $C_{18}H_{20}{}^{81}Br^{37}ClN_2O_5SNa$ $[M+Na]^+$: 516.980754; found: 516.980450.
IR (ATR): 3341, 3102, 2946, 2863, 1686, 1594, 1570, 1320, 1237, 1160, 1096 cm^{-1}.
M.P.: 165.8–166.3 °C (decomposition possible).

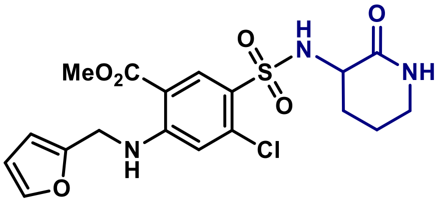

Methyl 4-chloro-2-((furan-2-ylmethyl)amino)-5-(N-(2-oxopiperidin-3-yl)sulfamoyl)benzoate (**34**)

Following the general procedure C, **34** was prepared from methyl furosemide (34.5 mg, 0.100 mmol, 1.00 equiv.), Pyry-BF_4 (33.6 mg, 0.200 mmol, 2.00 equiv.) and $MgCl_2$ (24.3 mg, 0.255 mmol, 2.55 equiv.) for 5 h, followed by Et_3N (28 µL, 0.20 mmol, 2.00 equiv.) and 3-aminopiperidine-2-one (17.1 mg, 0.15 mmol, 1.50 equiv.). The product was purified by column chromatography on silica gel (hexanes/EtOAc 7:3) to yield the title compound (31.7 mg, 0.089 mmol, 89% yield) as an off-white solid.
^{1}H NMR (400 MHz, $CDCl_3$) δ 8.63 (br t, *J* = 5.5 Hz, 1H), 8.57 (s, 1H), 7.39 (s, 1H), 6.89 (s, 1H), 6.34 (dd, *J* = 3.2, 1.9 Hz, 1H), 6.28 (d, *J* = 3.2,Hz, 1H), 6.22 (s, 1H), 5.78 (br s, 1H), 4.43 (d, *J* = 5.5 Hz, 2H), 3.88 (s, 3H), 3.48–3.44 (m, 21), 3.34–3.22 (m, 2H), 2.51–2.47 (m, 1H), 1.96–1.88 (m, 1H), 1.83–1.75 (m, 2H).
^{13}C NMR (101 MHz, $CDCl_3$) δ 169.9, 167.8, 153.3, 150.2, 142.8, 138.3, 135.9, 121.8, 113.9, 110.7, 108.3, 108.0, 53.7, 52.2, 42.1, 40.4, 28.9, 21.0.
HRMS (ESI): calcd. for $C_{18}H_{20}{}^{35}ClN_3O_6SNa$ $[M+Na]^+$: 464.065356; found: 464.065100; calcd. for $C_{18}H_{20}{}^{37}ClN_3O_6SNa$ $[M+Na]^+$: 466.062399; found: 466.062030.
IR (ATR): 3365, 3306, 3247, 3118, 2961, 2868, 1676, 1593, 1568, 1328, 1260, 1166, 1065 cm^{-1}.
M.P.: 195.4–196.2 °C (decomposition possible).

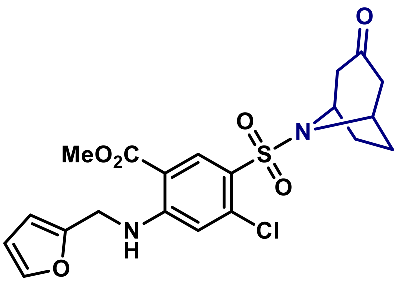

Methyl 4-chloro-2-((furan-2-ylmethyl)amino)-5-((3-oxo-8-azabicyclo[3.2.1]octan-8-yl)sulfonyl)benzoate (**35**)
Following the general procedure C, **35** was prepared from methyl furosemide (34.5 mg, 0.100 mmol, 1.00 equiv.), Pyry-BF_4 (33.6 mg, 0.200 mmol, 2.00 equiv.) and $MgCl_2$ (24.3 mg, 0.255 mmol, 2.55 equiv.) for 3 h, followed by Et_3N (49 µL, 0.15 mmol, 3.50 equiv.) and nortropinone hydrochloride (24.2 mg, 0.15 mmol, 1.50 equiv.). The product was purified by column chromatography on silica gel (hexanes/EtOAc 7:3) to yield the title compound (40.2 mg, 0.089 mmol, 89% yield) as an off-white solid.

^{1}H NMR (400 MHz, $CDCl_3$) δ 8.67 (br t, *J* = 5.5 Hz, 1H), 8.66 (s, 1H), 7.40 (dd, J = 1.9, 0.9 Hz, 1H), 6.89 (s, 1H), 6.35 (dd, *J* = 3.2, 1.9 Hz, 1H), 6.29 (dd, *J* = 3.2, 0.9 Hz, 1H), 4.49 (br s, 2H), 4.45 (d, *J* = 5.5 Hz, 2H), 3.89 (s, 3H), 2.86–2.81 (m, 2H), 2.39–2.35 (m, 2H), 2.14–2.11 (m, 2H), 1.74–1.68 (m, 2H).
^{13}C NMR (101 MHz, $CDCl_3$) δ 207.5, 167.6, 153.3, 150.2, 142.8, 138.5, 136.1, 123.0, 114.2, 110.7, 108.4, 108.0, 56.0, 52.3, 50.0, 40.4, 30.1.
HRMS (ESI): calcd. for $C_{20}H_{21}{}^{35}ClN_2O_6SNa$ $[M+Na]^+$: 475.070107; found: 475.070110; calcd. for $C_{20}H_{21}{}^{37}ClN_2O_6SNa$ $[M+Na]^+$: 477.067151; found: 477.066970.
IR (ATR): 3328, 2951, 2914, 2852, 1699, 1593, 1572, 1222, 1159, 1125 cm^{-1}.
M.P.: 148.3–149.7 °C (decomposition possible).

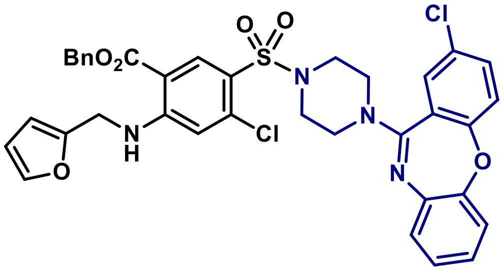

Benzyl 4-chloro-5-((4-(2-chlorodibenzo[b,f][1,4]oxazepin-11-yl)piperazin-1-yl)sulfonyl)-2-((furan-2-ylmethyl)amino)benzoate (**36**)

Following the general procedure C, **36** was prepared from benzyl furosemide (42.1 mg, 0.100 mmol, 1.00 equiv.), Pyry-BF_4 (33.6 mg, 0.200 mmol, 2.00 equiv.) and $MgCl_2$ (24.3 mg, 0.255 mmol, 2.55 equiv.) for 5 h, followed by Et_3N (49 µL, 0.15 mmol, 3.50 equiv.) and amoxapine (47.1 mg, 0.15 mmol, 1.50 equiv.). The product was purified by column chromatography on silica gel (hexanes/EtOAc 7:3) to yield the title compound (57.0 mg, 0.079 mmol, 79% yield) as an off-white solid.
^{1}H NMR (400 MHz, $CDCl_3$) δ 8.65 (t, *J* = 5.5 Hz, 1H) 8.64 (s, 1H), 7.43–7.31 (m, 7H), 7.18 (d, *J* = 8.6 Hz, 1H), 7.13–7.06 (m, 3H), 7.02–6.98 (m, 1H), 6.88 (s, 1H), 6.34 (dd, *J* = 3.3, 1.9 Hz, 1H), 6.28 (d, *J* = 3.3 Hz, 1H), 5.33 (s, 2H), 4.43 (d, *J* = 5.5 Hz, 2H), 3.58 (br s, 4H), 3.37 (br s, 4H).
^{13}C NMR (101 MHz, $CDCl_3$) δ 167.0, 159.4, 158.6, 153.3, 151.8, 150.2, 142.8, 139.8, 138.5, 136.8, 135.6, 133.0, 130.6, 128.9, 128.8, 128.6, 128.3, 127.2, 126.0, 125.1, 124.8, 123.0, 121.4, 120.3, 114.3, 110.7, 108.6, 108.0, 67.0, 47.4, 45.4, 40.4.
HRMS (ESI): calcd. for $C_{36}H_{30}{}^{35}Cl_2N_4O_6S$ Na $[M+Na]^+$: 739.115532; found: 739.115980; calcd. for $C_{36}H_{30}{}^{37}Cl_2N_4O_6S$ Na $[M+Na]^+$: 741.112576; found: 741.113010.
IR (ATR): 3331, 3065, 2917, 2853, 1685, 1588, 1561, 1254, 1217, 1160, 1098 cm^{-1}.
M.P.: 95.5–96.7 °C.

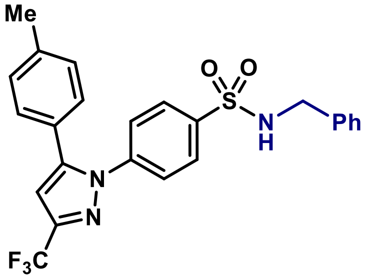

***N*-Benzyl-4-(5-(*p*-tolyl)-3-(trifluoromethyl)-1H-pyrazol-1-yl)benzenesulfonamide** (**37**)

Following the general procedure C, **37** was prepared from celecoxib (38.1 mg, 0.100 mmol, 1.00 equiv.), Pyry-BF_4 (33.6 mg, 0.200 mmol, 2.00 equiv.) and $MgCl_2$ (24.3 mg, 0.255 mmol, 2.55 equiv.) for 3 h, followed by Et_3N (28 µL, 0.20 mmol, 2.00 equiv.) and benzylamine (17 µL, 0.15 mmol, 1.50 equiv.). The product was purified by column chromatography on silica gel (hexanes/EtOAc 4:1) to yield the title compound (38.5 mg, 0.082 mmol, 82% yield) as an off-white solid.

^{1}H NMR (400 MHz, $CDCl_3$) δ 7.83 (d, *J* = 8.7 Hz, 2H), 7.45 (d, *J* = 8.7 Hz, 2H), 7.28–7.26 (m, 3H), 7.19–7.17 (m, 4H), 7.11 (d, *J* = 8.2 Hz, 2H), 6.75 (s, 1H), 4.87 (br t, *J* = 6.0 Hz, 1H), 4.13 (d, *J* = 6.0 Hz, 2H), 2.38 (s, 3H).
^{13}C NMR (101 MHz, $CDCl_3$) δ 145.4, 144.2 (q, *J* = 38.5 Hz), 142.7, 139.9, 139.5, 136.0, 129.9, 128.9, 128.8, 128.3, 128.0, 125.8, 125.7, 121.2 (q, *J* = 269.3 Hz), 106.5, 106.4, 47.5, 21.5.
^{19}F NMR (225 MHz, $CDCl_3$) δ -62.5 (s, 3F).
HRMS (ESI): calcd. for $C_{24}H_{20}F_3N_3O_2SNa$ $[M+Na]^+$: 494.112053; found: 494.111850.
IR (ATR): 3282, 3031, 2918, 1865, 1471, 1374, 1326, 1175, 1130, 1092, 1050, 967 cm^{-1}.
M.P.: 139.6–140.3 °C.

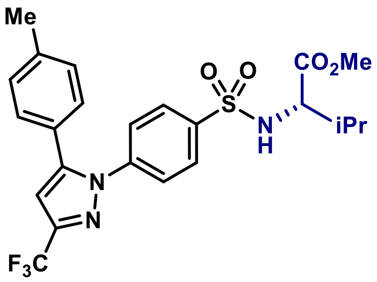

Methyl ((4-(5-(p-tolyl)-3-(trifluoromethyl)-1H-pyrazol-1-yl)phenyl)sulfonyl)-L-valinate (**38**)

Following the general procedure C, **38** was prepared from celecoxib (38.1 mg, 0.100 mmol, 1.00 equiv.), Pyry-BF_4 (33.6 mg, 0.200 mmol, 2.00 equiv.) and $MgCl_2$ (24.3 mg, 0.255 mmol, 2.55 equiv.) for 3 h, followed by Et_3N (49 µL, 0.15 mmol, 3.50 equiv.) and L-valine methyl ester hydrochloride (25.1 mg, 0.15 mmol, 1.50 equiv.). The product was purified by column chromatography on silica gel (hexanes/EtOAc 2:1) to yield the title compound (35.7 mg, 0.072 mmol, 72% yield) as an off-white solid.
^{1}H NMR (400 MHz, $CDCl_3$) δ 7.81 (d, *J* = 8.7 Hz, 2H), 7.45 (d, *J* = 8.7 Hz, 2H), 7.17 (d, *J* = 7.8 Hz, 2H), 7.08 (d, *J* = 7.8 Hz, 2H), 6.72 (s, 1H), 5.20 (d, *J* = 9.9 Hz, 1H), 3.76 (dd, *J* = 9.9, 5.0, 1H), 3.52 (s, 1H), 2.38 (s, 3H), 2.10–2.02 (m, 1H), 0.95 (d, *J* =6.9 Hz, 3H), 0.86 (d, *J* =6.9 Hz, 3H).
^{13}C NMR (101 MHz, $CDCl_3$) δ 171.7, 145.4, 144.2 (q, *J* = 38.5 Hz), 142.7, 139.9, 139.26, 129.9, 128.8, 128.4, 125.8, 125.5, 121.2 (q, *J* = 269.0 Hz), 106.5, 61.2, 52.6, 31.8, 21.5, 19.1, 17.5.
^{19}F NMR (225 MHz, $CDCl_3$) δ -62.5 (s, 3F).
HRMS (ESI): calcd. for $C_{23}H_{24}F_3N_3O_4SNa$ $[M+Na]^+$: 518.133183; found: 518.133310.
IR (ATR): 3312, 2969, 2924, 2880, 1736, 1470, 1348, 1336, 1236, 1059, 1130, 1090, 970 cm^{-1}.
M.P.: 130.6–132.8 °C.

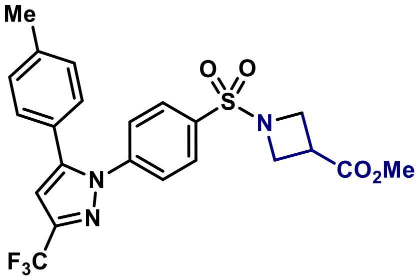

Methyl 1-((4-(5-(p-tolyl)-3-(trifluoromethyl)-1H-pyrazol-1-yl)phenyl)sulfonyl)azetidine-3-carboxylate (**39**)

Following the general procedure C, **39** was prepared from celecoxib (38.1 mg, 0.100 mmol, 1.00 equiv.), Pyry-BF_4 (33.6 mg, 0.200 mmol, 2.00 equiv.) and $MgCl_2$ (24.3 mg, 0.255 mmol, 2.55 equiv.) for 3 h, followed by Et_3N (28 µL, 0.20 mmol, 2.00 equiv.) and methyl azetidine-3-carboxylate hydrochloride (22.7 mg, 0.15 mmol, 1.50 equiv.). The product was purified by column chromatography on silica gel (hexanes/EtOAc 2:1) to yield the title compound (40.5 mg, 0.084 mmol, 84% yield) as an off-white solid.

^{1}H NMR (400 MHz, $CDCl_3$) δ 7.83 (d, *J* = 8.7 Hz, 2H), 7.55 (d, *J* = 8.7 Hz, 2H), 7.19 (d, *J* = 8.0 Hz, 2H), 7.12 (d, *J* = 8.0 Hz, 2H), 6.75 (s, 1H), 4.02–3.98 (m, 2H), 3.94–3.90 (m, 2H), 3.66 (s, 3H), 3.28 (tt, *J* = 8.9, 6.7 Hz, 1H 4H), 2.38 (s, 3H).
^{13}C NMR (101 MHz, $CDCl_3$) δ 171.5, 145.5, 144.3 (q, *J* = 38.6 Hz), 143.2, 140.0, 134.1, 129.9, 129.4, 128.9, 125.7, 125.7, 121.2 (q, *J* = 269.2 Hz), 106.5, 106.5, 53.1, 52.6, 31.6, 21.5.
^{19}F NMR (225 MHz, $CDCl_3$) δ -62.5 (s, 3F).
HRMS (ESI): calcd. for $C_{22}H_{20}F_3N_3O_4SNa$ $[M+Na]^+$: 502.101883; found: 502.101130.
IR (ATR): 3150, 3002, 2955, 2931, 2882, 1738, 1347, 1232, 1160, 1117, 1090, 972 cm^{-1}.
M.P.: 126.0–127.4 °C.

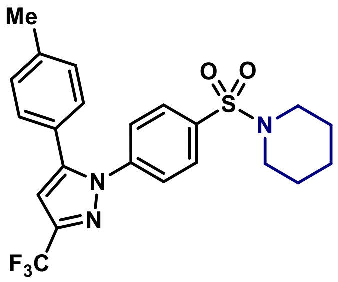

1-((4-(5-(*p*-Tolyl)-3-(trifluoromethyl)-1H-pyrazol-1-yl)phenyl)sulfonyl)piperidine (**40**)

Following the general procedure C, **40** was prepared from celecoxib (38.1 mg, 0.100 mmol, 1.00 equiv.), Pyry-BF_4 (33.6 mg, 0.200 mmol, 2.00 equiv.) and $MgCl_2$ (24.3 mg, 0.255 mmol, 2.55 equiv.) for 3 h, followed by Et_3N (28 µL, 0.20 mmol, 2.00 equiv.) and piperidine (15 µL, 0.15 mmol, 1.50 equiv.). The product was purified by column chromatography on silica gel (hexanes/EtOAc 9:1) to yield the title compound (35.9 mg, 0.080 mmol, 80% yield) as an off-white solid.
^{1}H NMR (400 MHz, $CDCl_3$) δ 7.74 (d, *J* = 8.7 Hz, 2H), 7.48 (d, *J* = 8.7 Hz, 2H), 7.17 (d, *J* = 7.9 Hz, 2H), 7.08 (d, *J* = 7.9 Hz, 2H), 6.75 (s, 1H), 2.99–2.96 (m, 4H), 2.38 (s, 3H), 1.66–1.60 (m, 4H), 1.45–1.39 (m, 2H).
^{13}C NMR (101 MHz, $CDCl_3$) δ 145.4, 144.2 (q, *J* = 38.4 Hz), 142.6, 139.9, 136.2, 129.9, 128.8, 128.7, 125.8, 125.7, 121.2 (q, *J* = 269.1 Hz), 106.3, 106.3, 47.1, 25.3, 23.6, 21.5.
^{19}F NMR (225 MHz, $CDCl_3$) δ -62.5 (s, 3F).
HRMS (ESI): calcd. for $C_{22}H_{22}F_3N_3O_2SNa$ $[M+Na]^+$: 472.127703; found: 472.126980.
IR (ATR): 3145, 3104, 3079, 2995, 2940, 2921, 2861, 1595, 1469, 1337, 1235, 1159, 1125, 1100, 972 cm^{-1}.
M.P.: 165.1–166.7 °C.

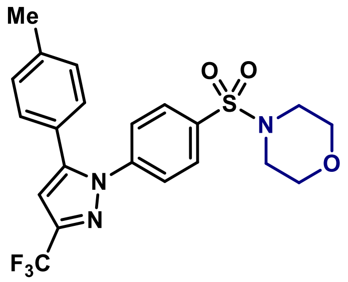

1-((4-(5-(*p*-Tolyl)-3)-3-(trifluoromethyl)-1H-pyrazol-1-yl)phenyl)sulfonyl)morpholine (**41**)

Following the general procedure C, **41** was prepared from celecoxib (38.1 mg, 0.100 mmol, 1.00 equiv.), Pyry-BF_4 (33.6 mg, 0.200 mmol, 2.00 equiv.) and $MgCl_2$ (24.3 mg, 0.255 mmol, 2.55 equiv.) for 3 h, followed by Et_3N (28 µL, 0.20 mmol, 2.00 equiv.) and morpholine (14 µL, 0.15 mmol, 1.50 equiv.). The product was purified by column chromatography on silica gel (hexanes/EtOAc 2:1) to yield the title compound (35.8 mg, 0.079 mmol, 79% yield) as an off-white solid.

^{1}H NMR (400 MHz, $CDCl_3$) δ 7.74 (d, *J* = 8.6 Hz, 2H), 7.52 (d, *J* = 8.6 Hz, 2H), 7.18 (d, *J* = 7.9 Hz, 2H), 7.10 (d, *J* = 7.9 Hz, 2H), 6.75 (s, 1H), 3.75–3.72 (m, 4H), 3.00–2.98 (m, 4H), 2.38 (s, 3H).
^{13}C NMR (101 MHz, $CDCl_3$) δ 145.4, 144.4 (q, *J* = 38.6 Hz), 143.0, 140.0, 134.9, 129.9, 128.9, 128.8, 125.8, 125.7, 121.1 (q, *J* = 269.2 Hz), 106.5, 106.5, 66.2, 46.1, 21.5.
^{19}F NMR (225 MHz, $CDCl_3$) δ -62.5 (s, 3F).
HRMS (ESI): calcd. for $C_{21}H_{21}F_3N_3O_3S$ $[M+H]^+$: 452.125024; found: 452.125070.
IR (ATR): 3141, 2990, 2928, 2868, 1598, 1499, 1471, 1453, 1346, 1260, 1234, 1155, 1130, 1097, 972 cm^{-1}.
M.P.: 162.4–163.8 °C.

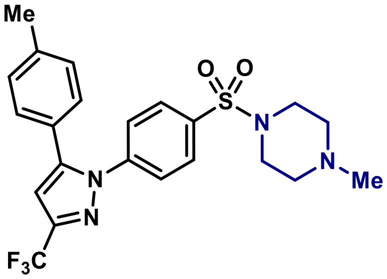

1-Methyl-4-((4-(5-(p-tolyl)-3-(trifluoromethyl)-1H-pyrazol-1-yl)phenyl)sulfonyl)piperazine (**42**)

Following the general procedure C, **42** was prepared from celecoxib (38.1 mg, 0.100 mmol, 1.00 equiv.), Pyry-BF_4 (33.6 mg, 0.200 mmol, 2.00 equiv.) and $MgCl_2$ (24.3 mg, 0.255 mmol, 2.55 equiv.) for 3 h, followed by Et_3N (28 µL, 0.20 mmol, 2.00 equiv.) and methylpiperazine (17 µL, 0.15 mmol, 1.50 equiv.). The product was purified by column chromatography on silica gel (EtOAc) to yield the title compound (37.6 mg, 0.081 mmol, 81% yield) as an off-white solid.
^{1}H NMR (400 MHz, $CDCl_3$) δ 7.73 (d, *J* = 8.7 Hz, 2H), 7.49 (d, *J* = 8.7 Hz, 2H), 7.18 (d, *J* = 8.0 Hz, 2H), 7.10 (d, *J* = 8.0 Hz, 2H), 6.73 (s, 1H), 3.05 (br s, 4H), 2.48–2.45 (m, 4H), 2.38 (s, 3H), 2.27 (s, 3H).
^{13}C NMR (101 MHz, $CDCl_3$) δ 145.3, 144.3 (q, *J* = 38.5 Hz), 142.8, 139.9, 135.4, 129.9, 128.8, 128.8, 125.8, 125.5, 121.2 (q, *J* = 269.2 Hz), 106.5, 54.1, 46.0, 45.8, 21.5.
^{19}F NMR (225 MHz, $CDCl_3$) δ -62.5 (s, 3F).
HRMS (ESI): calcd. for $C_{22}H_{24}F_3N_4O_2S$ $[M+H]^+$: 465.156658; found: 465.155890.
IR (ATR): 3137, 3106, 2952, 2931, 2795, 1596, 1495, 1472, 1447, 1345, 1236, 1155, 1132, 1099, 973 cm^{-1}.
M.P.: 149.8–150.9 °C.

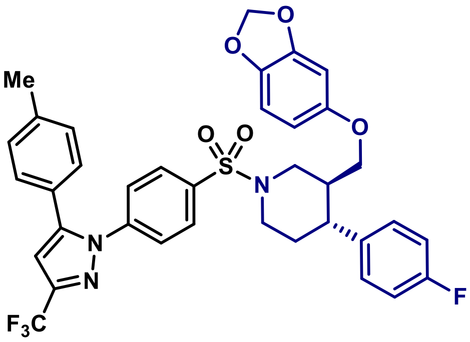

(3*S*,4*R*)-3-((Benzo[d][1,3]dioxol-5-yloxy)methyl)-4-(4-fluorophenyl)-1-((4-(5-(p-tolyl)-3-(trifluoromethyl)-1H-pyrazol-1-yl)phenyl)sulfonyl)piperidine (**43**)

Following the general procedure C, **43** was prepared from celecoxib (38.1 mg, 0.100 mmol, 1.00 equiv.), Pyry-BF_4 (33.6 mg, 0.200 mmol, 2.00 equiv.) and $MgCl_2$ (24.3 mg, 0.255 mmol, 2.55 equiv.) for 3 h, followed by Et_3N (49 µL, 0.15 mmol, 3.50 equiv.) and paroxetine hydrochloride (54.9 mg, 0.15 mmol, 1.50 equiv.). The product was purified by column chromatography on silica gel (hexanes/EtOAc 2:1) to yield the title compound (59.8 mg, 0.086 mmol, 86% yield) as an off-white solid.
^{1}H NMR (400 MHz, $CDCl_3$) δ 7.79 (d, *J* = 8.7 Hz, 2H), 7.52 (d, *J* = 8.7 Hz, 2H), 7.18 (d, *J* = 8.0 Hz, 2H), 7.13–7.07 (m, 4H), 6.97 (, *J* = 8.7 Hz, 2H), 6.76 (s, 1H), 6.61 (d, *J* = 8.5 Hz, 1H), 6.32 (d, *J* = 2.5 Hz, 1H), 6.10 (dd, *J* = 8.5, 2.5 Hz, 1H), 5.87 (s, 2H), 4.13–4.08 (m, 1H), 3.94 (dd, *J* = 11.2, 2.1 Hz, 1H), 3.57 (dd, *J* =

9.6, 2.7 Hz, 1H), 3.38 (dd, J = 9.6, 6.3 Hz, 1H), 2.47 (td, J = 11.2, 5.2 Hz, 1H), 2.39–2.32 (m, 2H), 2.37 (s, 3H), 2.25–2.16 (m, 1H), 1.97–1.86 (m, 1H).
^{13}C NMR (101 MHz, $CDCl_3$) δ 163.1, 160.6, 154.0, 148.3, 145.5, 144.3 (q, J = 38.7 Hz), 142.8, 142.0, 140.0, 138.2, 138.2, 135.8, 129.9, 128.9, 128.8, 125.8, 125.7, 121.2 (q, J = 269.4 Hz), 108.0, 106.4, 105.6, 101.3, 98.0, 68.4, 49.6, 46.8, 43.2, 41.8, 33.3, 21.4.
^{19}F NMR (225 MHz, $CDCl_3$) δ -62.5 (s, 3F), -115.6 (s, 1F).
HRMS (ESI): calcd. for $C_{36}H_{31}F_4N_3O_5SNa$ $[M+Na]^+$: 716.181277; found: 716.182020.
IR (ATR): 2922, 2853, 1597, 1488, 1469, 1343, 1235, 1160, 1131, 1093, 1035, 974 cm^{-1}.
M.P.: 88.5–90.3 °C (decomposition possible).

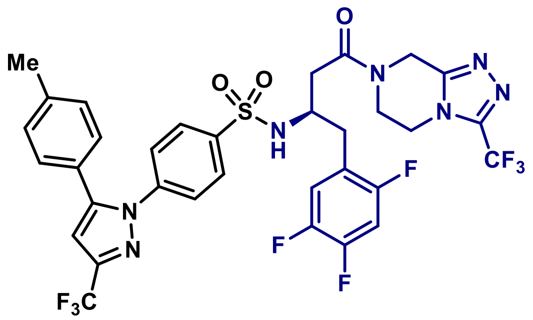

(*R*)-*N*-(4-Oxo-4-(3-(trifluoromethyl)-5,6-dihydro-[1,2,4]triazolo[4,3-a]pyrazin-7(8H)-yl)-1-(2,4,5-trifluorophenyl)butan-2-yl)-4-(5-(p-tolyl)-3-(trifluoromethyl)-1H-pyrazol-1-yl)benzenesulfonamide (**44**)

Following the general procedure C, **44** was prepared from celecoxib (38.1 mg, 0.100 mmol, 1.00 equiv.), Pyry-BF_4 (33.6 mg, 0.200 mmol, 2.00 equiv.) and $MgCl_2$ (24.3 mg, 0.255 mmol, 2.55 equiv.) for 3 h, followed by Et_3N (49 µL, 0.15 mmol, 3.50 equiv.) and sitagliptin phosphate (75.8 mg, 0.15 mmol, 1.50 equiv.). The product was purified by column chromatography on silica gel (EtOAc) to yield the title compound (53.9 mg, 0.070 mmol, 70% yield) as an off-white solid.
^{1}H NMR major rotamer (400 MHz, $CDCl_3$) δ 7.62 (d, J = 8.7 Hz, 2H), 7.32 (d, J = 8.7 Hz, 2H), 7.11 (d, J = 7.9 Hz, 2H), 7.06 (d, J = 8.1 Hz, 2H), 6.93 (ddd, J = 10.4, 9.1, 7.2 Hz, 1H), 6.71 (s, 1H), 6.69 (td, J = 9.5, 6.5 Hz, 1H), 6.58 (d, J = 9.1 Hz, 1H), 5.21 (d, J = 17.1 Hz, 1H), 4.89 (d, J = 17.1 Hz, 1H), 4.35–4.25 (m, 1H), 4.29–4.25 (m, 1H), 4.21–4.14 (m, 1H), 3.91–3.82 (m, 2H), 2.89–2.85 (m, 2H), 2.76 (dd, J = 14.1, 7.5 Hz, 1H), 2.61 (dd, J = 15.4, 4.6 Hz), 2.33 (s, 3H); **^{1}H NMR minor rotamer** (400 MHz, $CDCl_3$) δ 7.67 (d, J = 8.5 Hz, 1H), 7.37 (d, J = 8.4 Hz, 1H), 7.14 (d, J = 7.9 Hz, 1H), 7.09 (d, J = 8.1 Hz, 1H), 6.93 (ddd, J = 10.4, 9.0, 7.2 Hz, 1H), 6.73 (s, 1H), 6.32 (d, J = 8.9 Hz, 1H), 5.06 (d, J = 17.6 Hz, 1H), 4.95 (d, J = 17.6 Hz, 1H), 4.29–4.25 (m, 1H), 4.21–4.14 (m, 1H), 3.98–3.94 (m, 1H), 3.91–3.82 (m, 2H), 2.89–2.85 (m, 3H), 2.82 (dd, J = 14.1, 7.5 Hz, 2H), 2.76 (dd, J = 14.1, 7.5 Hz, 1H), 2.63 (dd, J = 11.8, 4.0 Hz, 1H), 2.35 (s, 3H).
^{13}C NMR major rotamer (101 MHz, $CDCl_3$) δ 169.73 , 155.92 (dd, J = 243.5, 8.7 Hz), 149.97, 149.00 (dt, J = 251.3, 13.3 Hz), 146.50 (dd, J = 243.0, 12.6 Hz), 145.16, 144.02 (q, J = 38.6 Hz), 143.88 (q, J = 40.3 Hz), 142.43, 139.77, 139.73, 129.69, 128.63, 127.79, 125.61, 125.24, 121.04 (q, J = 269.1 Hz), 120.29 (dt, J = 18.4, 4.8 Hz), 119.26 (dd, J = 19.0, 5.8 Hz), 118.14 (q, J = 270.5 Hz), 106.25 (q, J = 1.8 Hz), 105.58 (dd, J = 28.4, 20.7 Hz), 51.97, 43.10, 42.87, 38.14, 37.67, 34.08, 21.22; **minor rotamer** (101 MHz, $CDCl_3$) δ 169.19, 155.92 (dd, J = 243.5, 8.7 Hz), 150.33, 149.00 (dt, J = 251.3, 13.3 Hz), 146.50 (dd, J = 243.0, 12.6 Hz), 145.19, 144.02 (q, J = 38.6 Hz), 143.88 (q, J = 40.3 Hz), 142.59, 139.81, 139.55, 129.73, 128.66, 127.77, 125.62, 125.24, 121.04 (q, J = 269.1 Hz), 120.29 (dt, J = 18.4, 4.8 Hz), 119.26 (dd, J = 19.0, 5.8 Hz), 118.14 (q, J = 270.5 Hz), 106.35 (q, J = 1.8 Hz), 105.58 (dd, J = 28.4, 20.7 Hz), 51.21, 43.49, 41.67, 39.10, 36.72, 33.36, 21.26.
^{19}F NMR major rotamer (225 MHz, $CDCl_3$) δ –62.4 (s, 3F), –63.0 (s, 3F), –119.0 (d, J = 15.3 Hz, 1F), –134.5 (d, J = 21.3 Hz, 1F), –142.4 (d, J = 21.3, 15.3 Hz, 1F); **minor rotamer** (225 MHz, $CDCl_3$) δ δ –62.4 (s, 3F), –62.9 (s, 3F), –119.1 (d, J = 15.3 Hz, 1F), –134.3 (d, J = 21.3 Hz, 1F), –142.1 (d, J = 21.3, 15.3 Hz, 1F).
HRMS (ESI): calcd. for $C_{33}H_{26}F_9N_7O_3SNa$ $[M+Na]^+$: 794.156634; found: 794.156990.
IR (ATR): 3063, 2960, 2925, 2855, 1653, 1519, 1471, 1425, 1333, 1273, 1237, 1137, 1096, 1017, 974 cm^{-1}.

M.P.: 95.3–98.5 °C (decomposition possible).

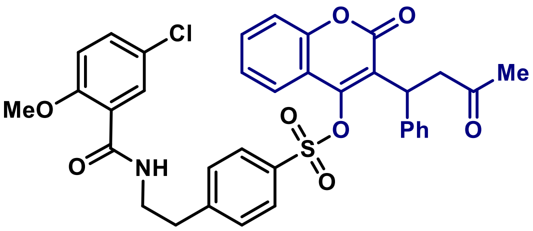

2-Oxo-3-(3-oxo-1-phenylbutyl)-2H-chromen-4-yl 4-(2-(5-chloro-2-methoxybenzamido)ethyl)benzenesulfonate (45)

Following the general procedure C, **45** was prepared from 5-chloro-2-methoxy-*N*-(4-sulfamoylphenethyl)benzamide (36.9 mg, 0.100 mmol, 1.00 equiv.), Pyry-BF_4 (33.6 mg, 0.200 mmol, 2.00 equiv.) and $MgCl_2$ (24.3 mg, 0.255 mmol, 2.55 equiv.) for 3 h, followed by Et_3N (28 µL, 0.20 mmol, 2.00 equiv.) and warfarin sodium salt (49.5 mg, 0.15 mmol, 1.50 equiv.). The product was purified by column chromatography on silica gel (hexanes/EtOAc 1:2) to yield the title compound (44.1 mg, 0.067 mmol, 67% yield) as an off-white solid.
^{1}H NMR (400 MHz, $CDCl_3$) δ 8.15 (d, *J* = 2.8 Hz, 1H), 8.11 (d, *J* =8.4 Hz, 2H), 7.92 (br s, 1H), 7.76 (dd, *J* = 8.1, 1.5 Hz, 1H), 7.53–7.49 (m, 3H), 7.36 (dd, *J* = 8.8, 2.8 Hz, 1H), 7.29–7.24 (m, 2H), 7.18–7.11 (m, 5H), 6.82 (d, *J* = 8.8 Hz, 1H), 4.80 (dd, *J* = 9.8, 4.2 Hz, 1H), 3.91–3.70 (m, 3H), 3.68 (s, 3H), 3.10–3.04 (m, 3H), 2.17 (s, 3H).
^{13}C NMR (101 MHz, $CDCl_3$) δ 206.3, 164.3, 160.9, 156.0, 153.3, 152.4, 148.2, 139.9, 133.8, 132.5, 132.4, 132.0, 130.4, 128.8, 128.5, 127.8, 127.0, 126.8, 125.1, 124.6, 123.7, 122.8, 116.7, 116.5, 112.9, 56.2, 44.8, 40.8, 37.2, 36.0, 30.4.
HRMS (ESI): calcd. for $C_{35}H_{30}{}^{35}ClNO_8SNa$ $[M+Na]^+$: 682.127288; found: 682.126240; calcd. for $C_{35}H_{30}{}^{37}ClNO_8SNa$ $[M+Na]^+$: 684.124332; found: 684.123130.
IR (ATR): 3379, 3028, 2942, 2880, 1732, 1715, 1646, 1535, 1380, 1267, 1193, 1038, 1022 cm^{-1}.
M.P.: 179.6–180.8 °C (decomposition possible).

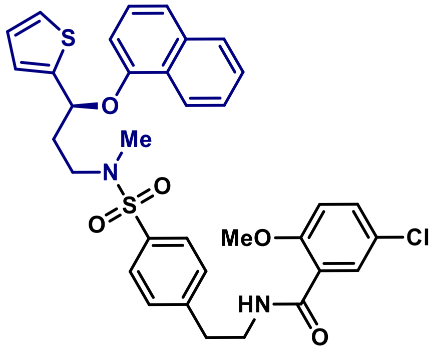

(S)-5-Chloro-2-methoxy-N-(4-(N-methyl-N-(3-(naphthalen-1-yloxy)-3-(thiophen-2-yl)propyl)sulfamoyl)phenethyl)benzamide (46)

Following the general procedure C, **46** was prepared from 5-chloro-2-methoxy-*N*-(4-sulfamoylphenethyl)benzamide (36.9 mg, 0.100 mmol, 1.00 equiv.), Pyry-BF_4 (33.6 mg, 0.200 mmol, 2.00 equiv.) and $MgCl_2$ (24.3 mg, 0.255 mmol, 2.55 equiv.) for 3 h, followed by Et_3N (49 µL, 0.15 mmol, 3.50 equiv.) and duloxetine hydrochloride (50.1 mg, 0.15 mmol, 1.50 equiv.). The product was purified by column chromatography on silica gel (hexanes/EtOAc 1:1) to yield the title compound (48.9 mg, 0.075 mmol, 75% yield) as an off-white solid.
^{1}H NMR (400 MHz, $CDCl_3$) δ 8.31–8.29 (m, 1H), 8.15 (d, *J* = 2.8 Hz, 1H), 7.80–7.75 (m, 2H), 7.69 (d, *J* = 8.3 Hz, 2H), 7.50–7.45 (m, 2H), 7.40 (d, *J* = 8.3 Hz, 1H), 7.36–7.32 (m, 3H), 7.29 (d, *J* = 7.9 Hz, 1H), 7.21 (dd, *J* = 5.1, 1.2 Hz, 1H), 7.11 (d, *J* = 3.1 Hz, 1H), 6.94 (dd, *J* = 5.1, 3.5 Hz, 1H), 6.87 (d, *J* = 7.6 Hz, 1H), 6.80 (d, *J* = 8.8 Hz, 1H), 5.78 (dd, *J* = 7.8, 4.8 Hz, 1H), 3.74–3.69 (m, 2H), 3.71 (s, 3H), 3.35–3.20 (m, 2H), 2.96 (t, *J* = 6.9 Hz, 2H), 2.77 (s, 3H), 2.56–2.47 (m, 1H), 2.37–2.29 (m, 1H).

^{13}C NMR (101 MHz, $CDCl_3$) δ 164.1, 156.0, 153.1, 144.8, 144.4, 135.6, 134.7, 132.5, 132.0, 129.7, 127.8, 127.7, 126.9, 126.8, 126.5, 126.1, 125.9, 125.4, 125.2, 125.1, 122.7, 122.0, 121.0, 112.9, 107.3, 73.7, 56.3, 47.3, 40.7, 37.7, 35.80 35.6.
HRMS (ESI): calcd. for $C_{34}H_{33}{}^{35}ClN_2O_5S_2Na$ $[M+Na]^+$: 671.141165; found: 671.142270; calcd. for $C_{34}H_{33}{}^{37}ClN_2O_5S_2Na$ $[M+Na]^+$: 673.138208; found: 673.139010.
IR (ATR): 3389, 3053, 2931, 2862, 1650, 1529, 1481, 1461, 1396, 1336, 1264, 1236, 1150, 1091, 1017 cm^{-1}.
M.P.: 68.0–70.3 °C (decomposition possible).

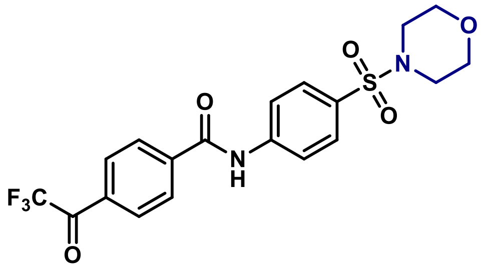

***N*-(4-(Morpholinosulfonyl)phenyl)-4-(2,2,2-trifluoroacetyl)benzamide** (**47**)

Following the general procedure C, **47** was prepared from *N*-(4-sulfamoylphenyl)-4-(2,2,2-trifluoroacetyl)benzamide (37.2 mg, 0.100 mmol, 1.00 equiv.), Pyry-BF_4 (33.6 mg, 0.200 mmol, 2.00 equiv.) and $MgCl_2$ (24.3 mg, 0.255 mmol, 2.55 equiv.) for 5 h, followed by Et_3N (28 µL, 0.20 mmol, 2.00 equiv.) morpholine (14 µL, 0.15 mmol, 1.50 equiv.). The product was purified by column chromatography on silica gel (hexanes/EtOAc 6:4) to yield the title compound (30.2 mg, 0.068 mmol, 68% yield) as an yellowish wax.
^{1}H NMR major rotamer (400 MHz, DMSO-*d*$_6$) δ 8.20 (s, 4H), 8.1 (d, *J* = 8.8 Hz, 2H), 7.77 (d, *J* = 8.8 Hz, 2H), 4.51 (s, 1H), 3.65–3.62 (m, 4H), 2.88–2.86 (m, 4H); **minor rotamer** (400 MHz, DMSO-*d*$_6$) δ 8.00 (d, *J* = 8.4 Hz, 2H), 4.47 (s, 1H).
^{13}C NMR major rotamer (101 MHz, DMSO-*d*$_6$) δ 179.5 (q, *J* = 34.8 Hz), 165.0, 143.2, 140.6, 131.7, 129.9, 128.8, 120.2, 116.5 (q, *J* = 291.5 Hz), 65.3, 45.9; **minor rotamer** (101 MHz, DMSO-*d*$_6$) δ 166.0, 143.6, 142.2 135.2, 129.0, 128.5, 127.5, 127.4, 120.0.
^{19}F NMR major rotamer (225 MHz, DMSO-*d*$_6$) δ –72.5 (s, 3F); **minor rotamer** (225 MHz, DMSO-*d*$_6$) δ –84.6 (s, 3F).
HRMS (ESI): calcd. for $C_{19}H_{16}F_3N_2O_5S$ $[M–H]^-$: 441.073756; found: 441.074130.
IR (ATR): 3320, 3258, 3093, 2963, 2917, 2856, 1647, 1587, 1532, 1487, 1328, 1301, 1218, 1160, 1094, 1070 cm^{-1}.

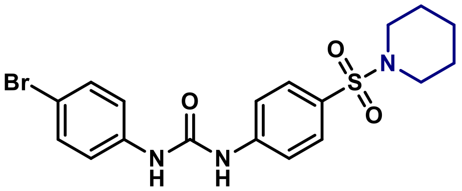

1-(4-Bromophenyl)-3-(4-(piperidin-1-ylsulfonyl)phenyl)urea (**48**)

Following the general procedure C, **48** was prepared from 4-(3-(4-bromophenyl)ureido)benzenesulfonamide (37.0 mg, 0.100 mmol, 1.00 equiv.), Pyry-BF_4 (33.6 mg, 0.200 mmol, 2.00 equiv.) and $MgCl_2$ (24.3 mg, 0.255 mmol, 2.55 equiv.) for 5 h, followed by Et_3N (28 µL, 0.20 mmol, 2.00 equiv.) and piperidine (15 µL, 0.15 mmol, 1.50 equiv.). The product was purified by column chromatography on silica gel (hexanes/EtOAc 6:4) to yield the title compound (33.3 mg, 0.076 mmol, 76% yield) as an off-white solid.
^{1}H NMR (400 MHz, DMSO-*d*$_6$) δ 9.21 (br s, 1H), 8.96 (br s, 1H), 7.69 (d, *J* = 8.9 Hz, 2H), 7.63 (d, *J* = 8.9 Hz, 2H), 7.46 (s, 4H), 2.85–2.82 (m, 4H), 1.55–1.49 (m, 4H), 1.36–1.30 (m, 2H).
^{13}C NMR (101 MHz, DMSO-*d*$_6$) δ 152.1, 143.8, 138.7, 131.6, 128.7, 127.7, 120.4, 117.8, 113.7, 46.6, 24.9, 22.9.

HRMS (ESI): calcd. for $C_{18}H_{19}{}^{79}BrN_3O_3S$ [M–H]$^-$: 436.033614; found: 438.031608; calcd. for $C_{18}H_{19}{}^{81}BrN_3O_3S$ [M–H]$^-$: 436.033614; found: 438.031990.
IR (ATR): 3324, 3266, 3099, 2952, 2916, 2848, 2681, 1678, 1648, 1587, 1536, 1488, 1328, 1306, 1214, 1156, 1092, 1070 cm^{-1}.
M.P.: 243.5–244.9 °C.

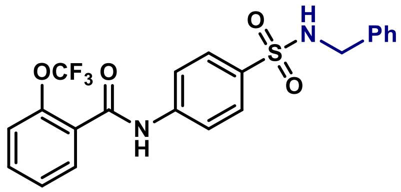

***N*-(4-(*N*-Benzylsulfamoyl)phenyl)-2-(trifluoromethoxy)benzamide** (**49**)

Following the general procedure C, **49** was prepared from *N*-(4-sulfamoylphenyl)-2-(trifluoromethoxy)benzamide (36.0 mg, 0.100 mmol, 1.00 equiv.), Pyry-BF$_4$ (33.6 mg, 0.200 mmol, 2.00 equiv.) and $MgCl_2$ (24.3 mg, 0.255 mmol, 2.55 equiv.) for 5 h, followed by Et_3N (28 µL, 0.20 mmol, 2.00 equiv.) and benzylamine (17 µL, 0.15 mmol, 1.50 equiv.). The product was purified by column chromatography on silica gel (hexanes/EtOAc 6:4) to yield the title compound (34.9 mg, 0.077 mmol, 77% yield) as an off-white solid.
^{1}H NMR (400 MHz, DMSO-*d*$_6$) δ 8.10 (br s, 1H), 7.92 (d, *J* = 8.8 Hz, 2H), 7.83 (d, *J* = 8.8 Hz, 2H), 7.77 (dd, *J* = 7.7, 1.8 Hz, 1H), 7.69 (td, *J* = 7.7, 1.8 Hz), 7.58–7.53 (m, 2H), 7.32–7.21 (m, 5H), 4.00 (s, 2H).
^{13}C NMR (101 MHz, DMSO-*d*$_6$) δ 163.8, 144.9 (q, *J* = 2.0 Hz), 142.2, 137.7, 135.3, 132.2, 130.8, 129.8, 128.2, 127.8, 127.6, 127.1, 121.8, 120.1 (q, *J* = 257.4 Hz), 119.4, 118. 46.2.
^{19}F NMR (225 MHz, $CDCl_3$) δ -56.6 (s, 3F).
HRMS (ESI): calcd. for $C_{21}H_{17}F_3N_2O_4SNa$ [M+Na]$^+$: 473.075334; found: 473.074890.
IR (ATR): 3347, 3187, 2987, 2899, 1665, 1592, 1542, 1341, 1328, 1254, 1180, 1150, 1091, 1057 cm^{-1}.

M.P.: 162.3–163.7 °C.

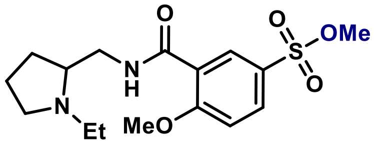

Methyl 4-methoxy-3-(((1-methylpyrrolidin-2-yl)methyl)carbamoyl)benzenesulfonate (**50**)
Following the general procedure C, **50** was prepared from (±)-sulpiride (34.1 mg, 0.100 mmol, 1.00 equiv.), Pyry-BF$_4$ (33.6 mg, 0.200 mmol, 2.00 equiv.) in MeOH (0.1 M) for 3 h. The product was purified by column chromatography on silica gel (DCM/MeOH 95:5) to yield the title compound (32.0 mg, 0.090 mmol, 90% yield) as an off-white solid.

^{1}H NMR (400 MHz, $CDCl_3$) δ 8.96 (br t, *J* = 5.1 Hz, 1H), 8.65 (d, *J* = 2.5 Hz, 1H), 8.03 (dd, *J* = 8.8, 2.5 Hz), 7.18 (d, *J* = 8.8 Hz), 4.16 (s, 3H), 4.05–3.94 (m, 2H), 3.87–3.79 (m, 1H), 3.77 (s, 3H), 3.76–3.69 (m, 1H), 3.39–3.31 (m, 1H), 3.17–3.04 (m, 2H), 2.40–2.29 (m, 1H), 2.18–2.08 (m, 2H), 2.04–1.95 (m, 1H), 1.39 (t, *J* = 7.3 Hz, 3H).
^{13}C NMR (101 MHz, CD_3OD-*d*$_4$) δ 169.3, 160.4, 132.3, 130.4, 121.6, 112.8, 69.5, 57.0, 55.0, 51.5, 41.4, 28.2, 23.5, 11.4.
HRMS (ESI): calcd. for $C_{16}H_{25}N_2O_5S$ [M+H]$^+$: 357.147870; found: 357.147500.
IR (ATR): 3366, 3027, 2992, 2712, 1638, 1597, 1536, 1486, 1171, 1094, 1025 cm^{-1}.
M.P.: 99.3–100.5 °C.

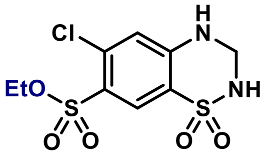

Ethyl 6-chloro-3,4-dihydro-2H-benzo[e][1,2,4]thiadiazine-7-sulfonate 1,1-dioxide (**51**)

Following the general procedure C, **51** was prepared from hydrochlorothiazide (29.8 mg, 0.100 mmol, 1.00 equiv.), Pyry-BF_4 (33.6 mg, 0.200 mmol, 2.00 equiv.) in EtOH (0.1 M) for 5 h. The product was purified by column chromatography on silica gel (hexanes/EtOAc 1:2) to yield the title compound (23.9 mg, 0.073 mmol, 73% yield) as an off-white wax.
^{1}H NMR (400 MHz, $CDCl_3$) δ 8.28 (s, 1H), 6.79 (s, 1H), 5.39 (br s, 1H), 5.11 (t, *J* = 8.7 Hz, 1H), 4.90 (dd, *J* = 8.7, 2.7 Hz), 4.19 (q, *J* = 7.1 Hz), 1.37 (t, *J* = 7.1 Hz).
^{13}C NMR (101 MHz, $CDCl_3$) δ 146.7, 137.4, 129.6, 122.8, 120.2, 118.4, 68.3, 55.5, 14.9.
HRMS (ESI): calcd. for $C_9H_{10}{}^{35}ClN_2O_5S_2$ $[M–H]^-$: 324.972521; found: 324.973150; calcd. for $C_9H_{10}{}^{37}ClN_2O_5S_2$ $[M–H]^-$: 326.969564; found: 326.969950.
IR (ATR): 3370, 3261, 3084, 2985, 2943, 1593, 1328, 1156, 925 cm^{-1}.

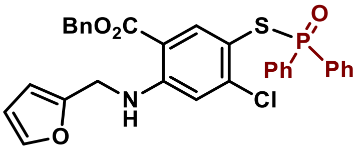

Benzyl 4-chloro-5-((diphenylphosphoryl)thio)-2-((furan-2-ylmethyl)amino)benzoate (**51**)

A solution of freshly prepared benzyl furosemide chloride (**22**) (83.4 mg, 0.19 mmol, 1.0 equiv.) in THF (0.1M) was treated with Ph_2POH (114.9 mg, 0.57 mmol, 3.0 equiv.) at rt for 2 h. Then, the solvent was evaporated and the residue was purified by column chromatography on silica gel (hexanes/EtOAc 6:4) to yield compound **51** (95.5 mg, 0.17 mmol, 88% yield) as an orange solid (82% over two steps starting from benzyl furosemide).
^{1}H NMR (400 MHz, $CDCl_3$) δ 8.23 (br t, *J* = 5.4 Hz, 1H), 8.15 (d, *J* = 1.8 Hz, 1H), 7.87–7.82 (m, 4H), 7.49–7.36 (m, 12H), 6.74 (s, 1H), 6.32 (dd, *J* = 3.2, 1.8 Hz, 1H), 6.21 (dd, *J* = 3.2, 0.91 Hz, 1H), 5.25 (s, 2H), 4.32 (d, *J* = 5.4 Hz, 2H).
^{13}C NMR (101 MHz, $CDCl_3$) δ 67.2, 151.5, 150.9, 145.9, 142.5, 141.6, 141.5, 136.1, 133.1, 132.4, 132.4, 132.0, 131.9, 131.8, 128.7, 128.6, 128.5, 128.4, 128.2, 112.9, 110.5, 110.19, 109.2, 107.6, 66.5, 40.3.
HRMS (ESI): calcd. for $C_{31}H_{26}{}^{35}ClNO_4PS$ $[M]^+$: 574.100324; found: 574.100390; calcd. for $C_{31}H_{26}{}^{37}ClNO_4PS$ $[M]^+$: 576.097367; found: 576.097210.
IR (ATR): 3340, 3059, 2953, 1685, 1587, 1564, 1486, 1438, 1216, 1093 cm^{-1}.
M.P.: 66.1-68.3 °C.

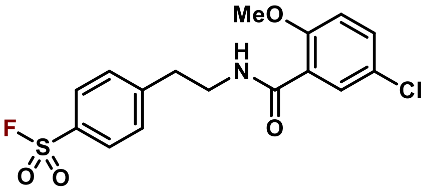

4-(2-(5-Chloro-2-methoxybenzamido)ethyl)benzenesulfonyl fluoride (**52**)

A solution of freshly prepared 4-(2-(5-chloro-2-methoxybenzamido)ethyl)benzenesulfonyl chloride (**23**) (30.6 mg, 0.079 mmol, 1.0 equiv.) in CH_3CN (0.1M) was treated with a sat. solution of KHF_2 in H_2O (15.6 mg, 0.2 mmol, 2.5 equiv.) at rt for 3 h. Then, the solvent was evaporated and the residue was dissolved in EtOAc, washed with brine and dried over $MgSO_4$ and evaporated yielding compound **52** (28.1 mg, 0.076 mmol, 97% yield) as a pale yellow solid (76% over two steps starting from 4-(2-(5-chloro-2-methoxybenzamido)ethyl)benzenesulfonamide).
^{1}H NMR (400 MHz, $CDCl_3$) δ 8.16 (d, *J* = 2.8 Hz, 1H), 8.36 (d, *J* = 8.3 Hz, 1H), 7.81 (br s, 1H), 7.51 (d, *J* = 8.3 Hz, 2H), 7.39 (dd, *J* = 8.9, 2.8 Hz, 1H), 6.88 (d, *J* = 8.9 Hz, 1H), 3.81 (s, 3H), 3.76 (q, *J* = 6.9 Hz, 2H), 3.07 (t, *J* = 6.9 Hz, 2H).
^{13}C NMR (101 MHz, $CDCl_3$) δ 164.2, 156.0, 148.4, 132.7, 132.1, 131.3 (d, *J* = 24.5 Hz), 130.3, 128.8, 127.0, 122.7, 113.0, 56.3, 40.7, 35.9.
^{19}F NMR (225 MHz, $CDCl_3$) δ -66.1 (s, 1F).

HRMS (ESI): calcd. for $C_{16}H_{16}{}^{35}ClFNO_4S$ $[M]^+$: 372.046713; found: 372.046400; calcd. for $C_{16}H_{16}{}^{37}ClFNO_4S$ $[M]^+$: 374.043756; found: 374.043310.
IR (ATR): 3385, 3103, 3045, 2941, 2852, 1646, 1532, 1397, 1272, 1210, 1181, 1016 cm^{-1}.
M.P.: 125.4-126.2 °C.

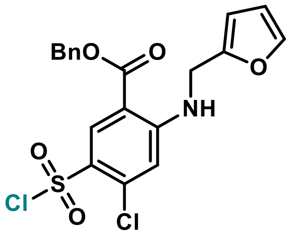

Gram Scale: Benzyl furosemide chloride (**22**)

Following the general procedure B, **22** was prepared from benzyl furosemide (968.0 mg, 2.30 mmol, 1.00 equiv.), Pyry-BF_4 (772.3 mg, 4.60 mmol, 2.00 equiv.) and $MgCl_2$ (558.4 mg, 5.87 mmol, 2.55 equiv.) for 5 h. The product was purified by column chromatography on silica gel (hexanes/EtOAc 8:2) to yield the title compound (860.3 mg, 1.95 mmol, 85% yield) as a yellow wax.

5. Spectra for Described Compounds

^{1}H Spectra

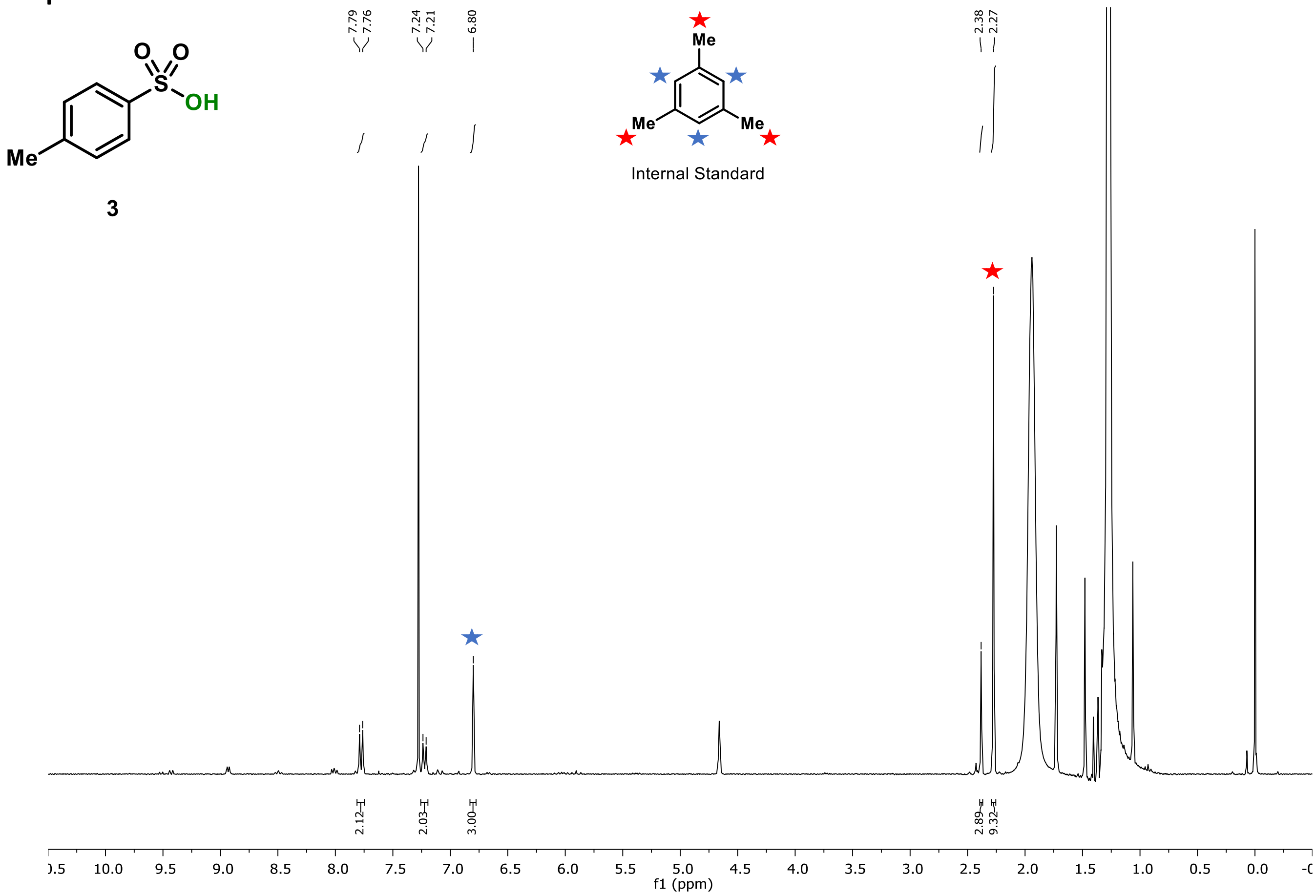

^{1}H Spectra

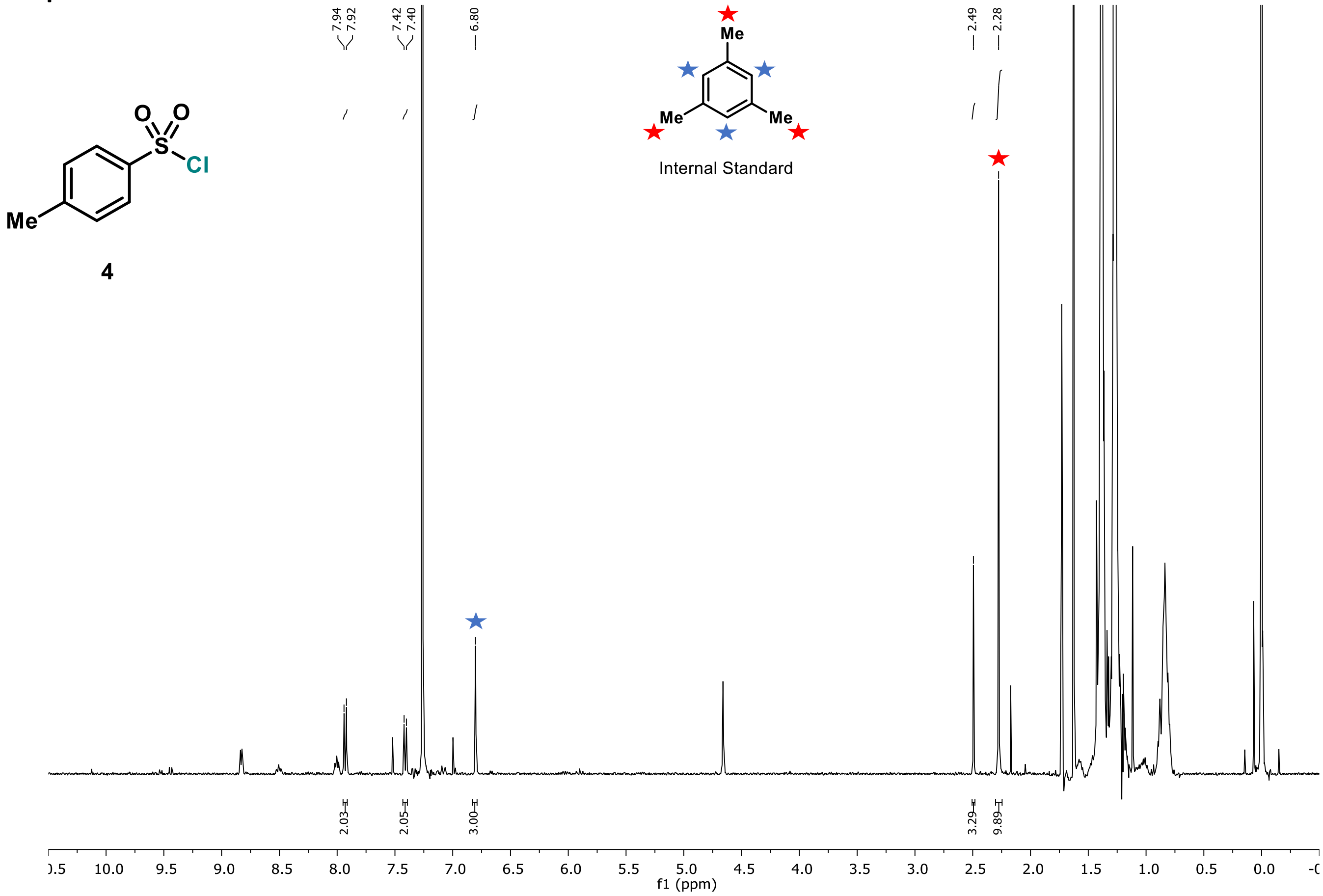

^{1}H Spectra

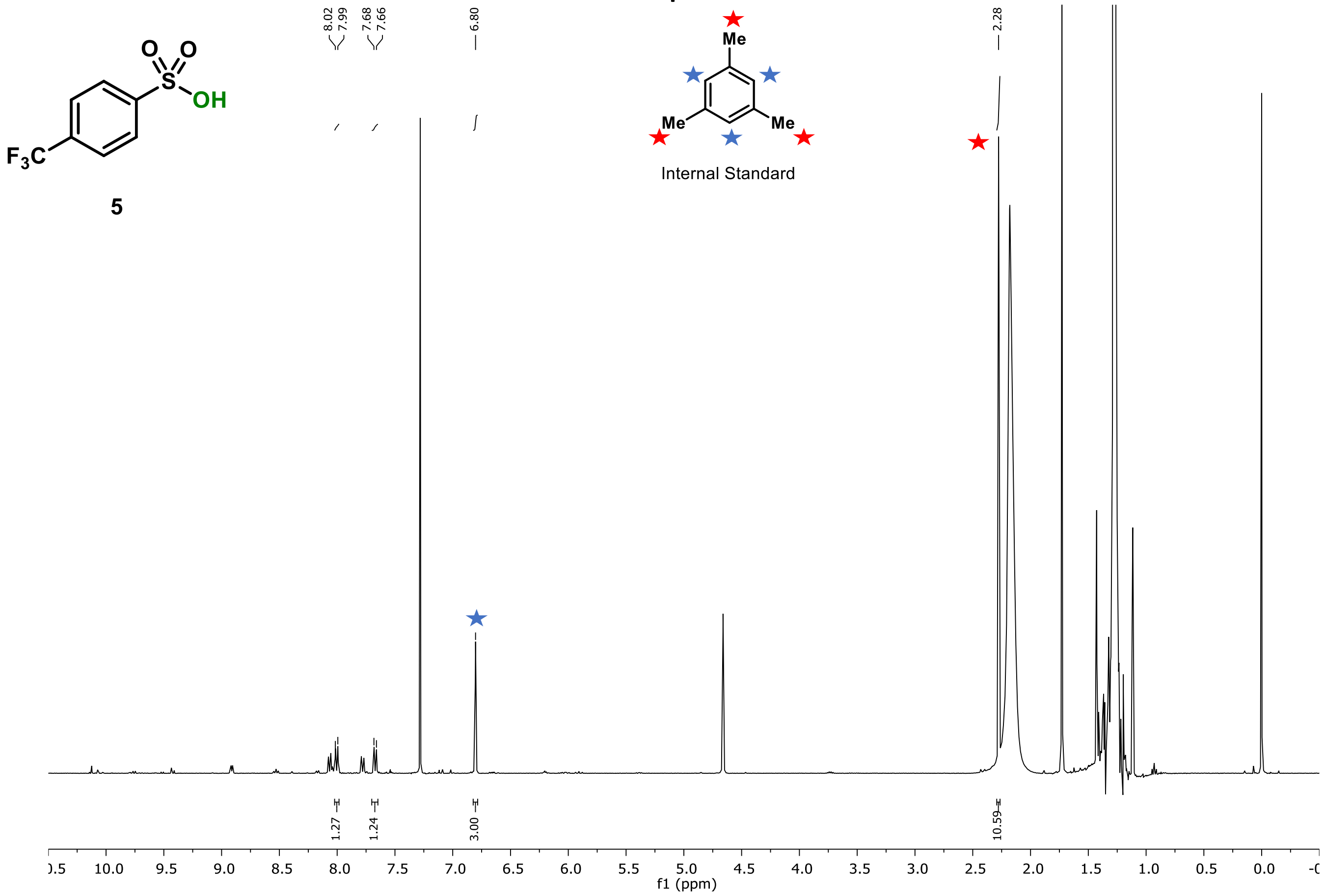

^{19}F Spectra

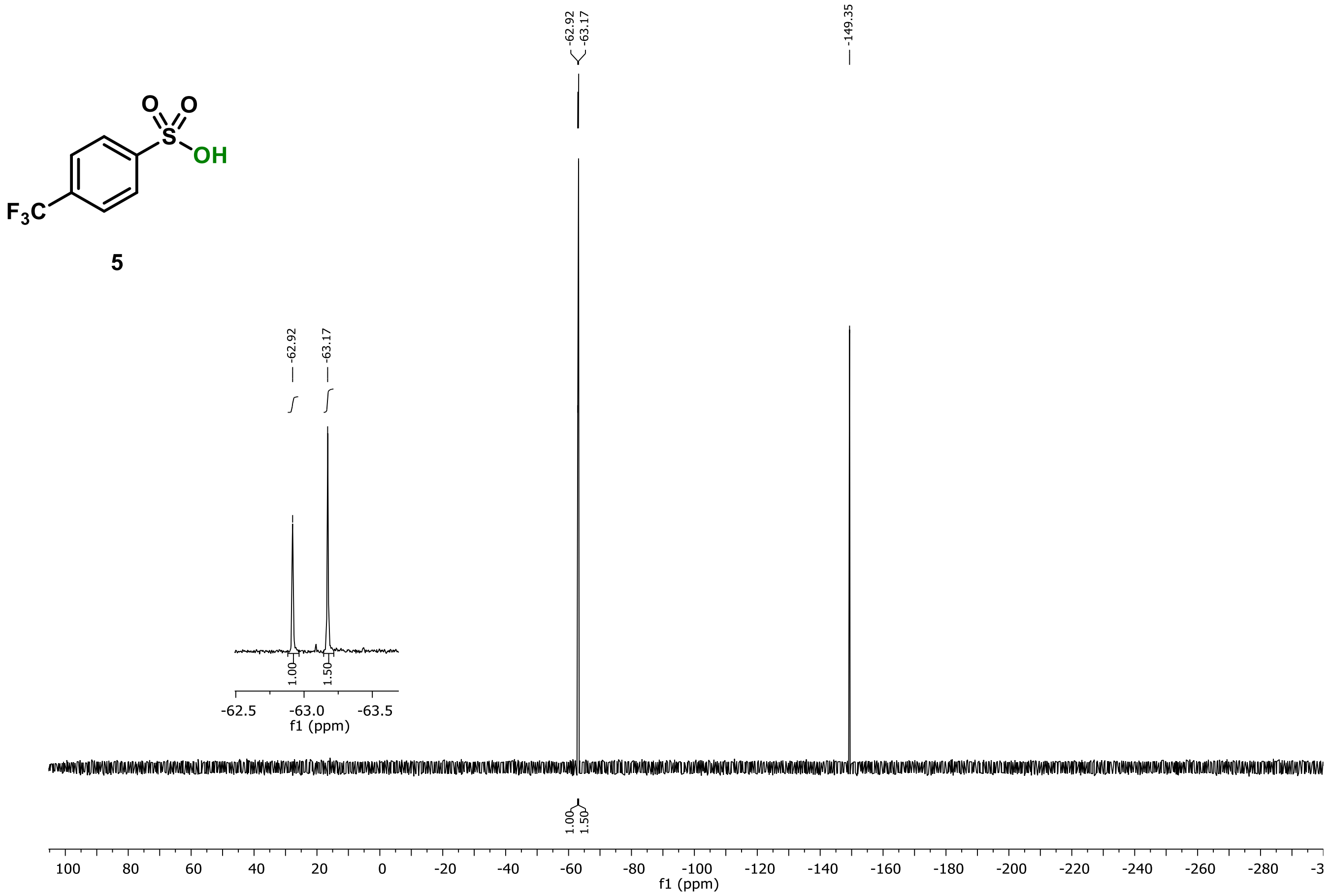

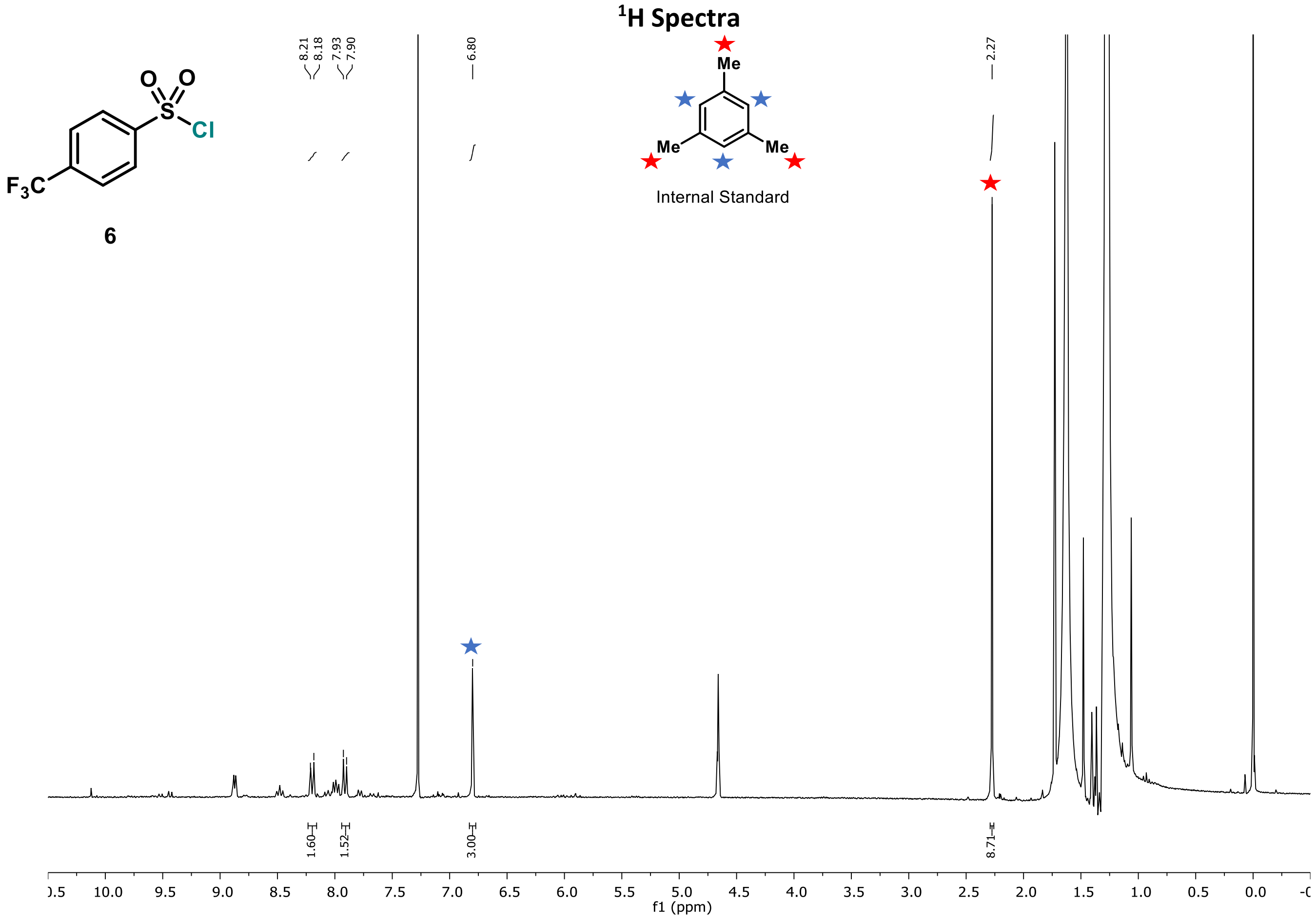

¹H Spectra
6
Me
Me
Me
Internal Standard
8.21
8.18
7.93
7.90
6.80
2.27
1.60
1.52
3.00
8.71
f1 (ppm)

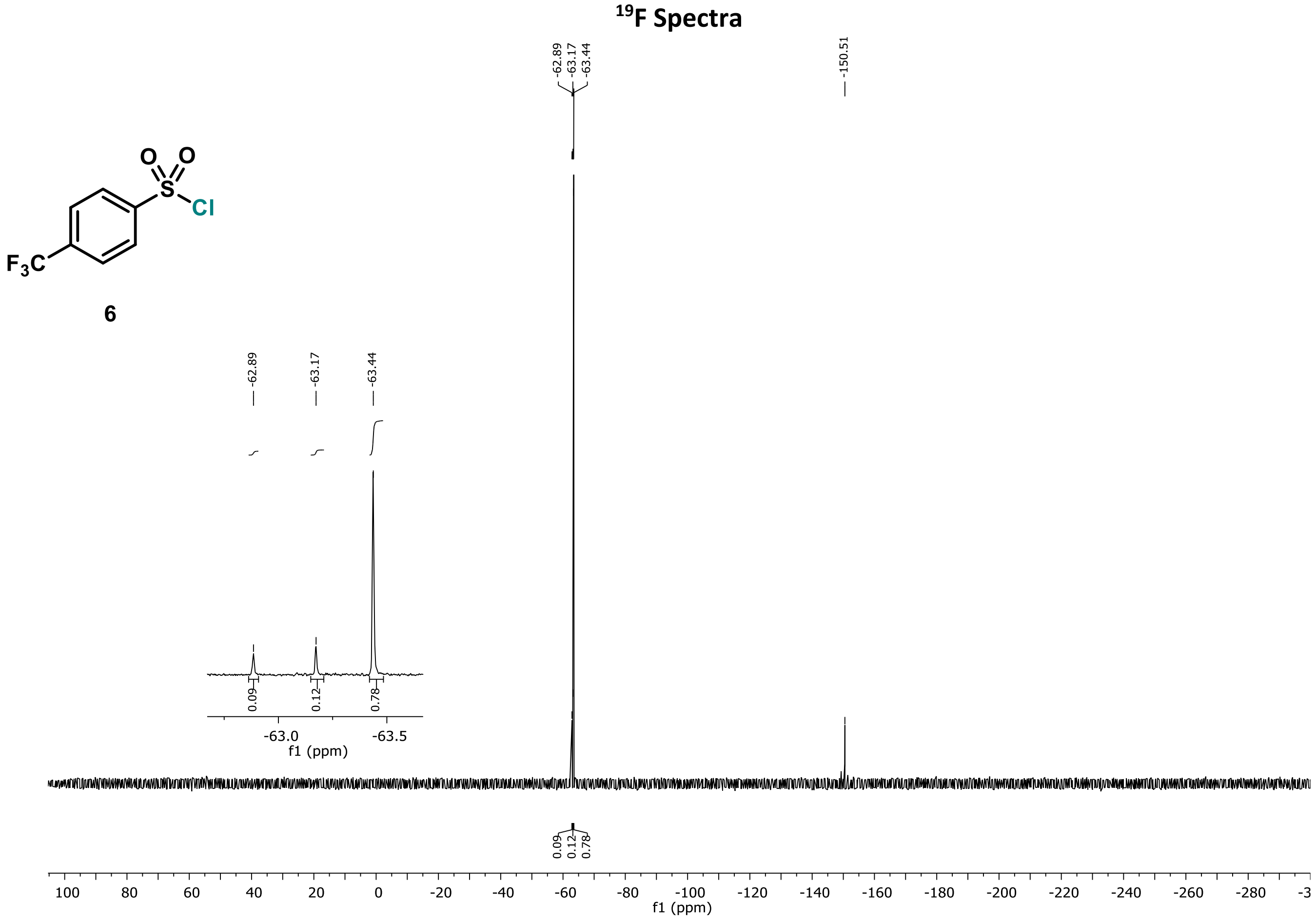

¹⁹F Spectra
6
-62.89
-63.17
-63.44
-150.51
0.09
0.12
0.78
f1 (ppm)

[1]H Spectra

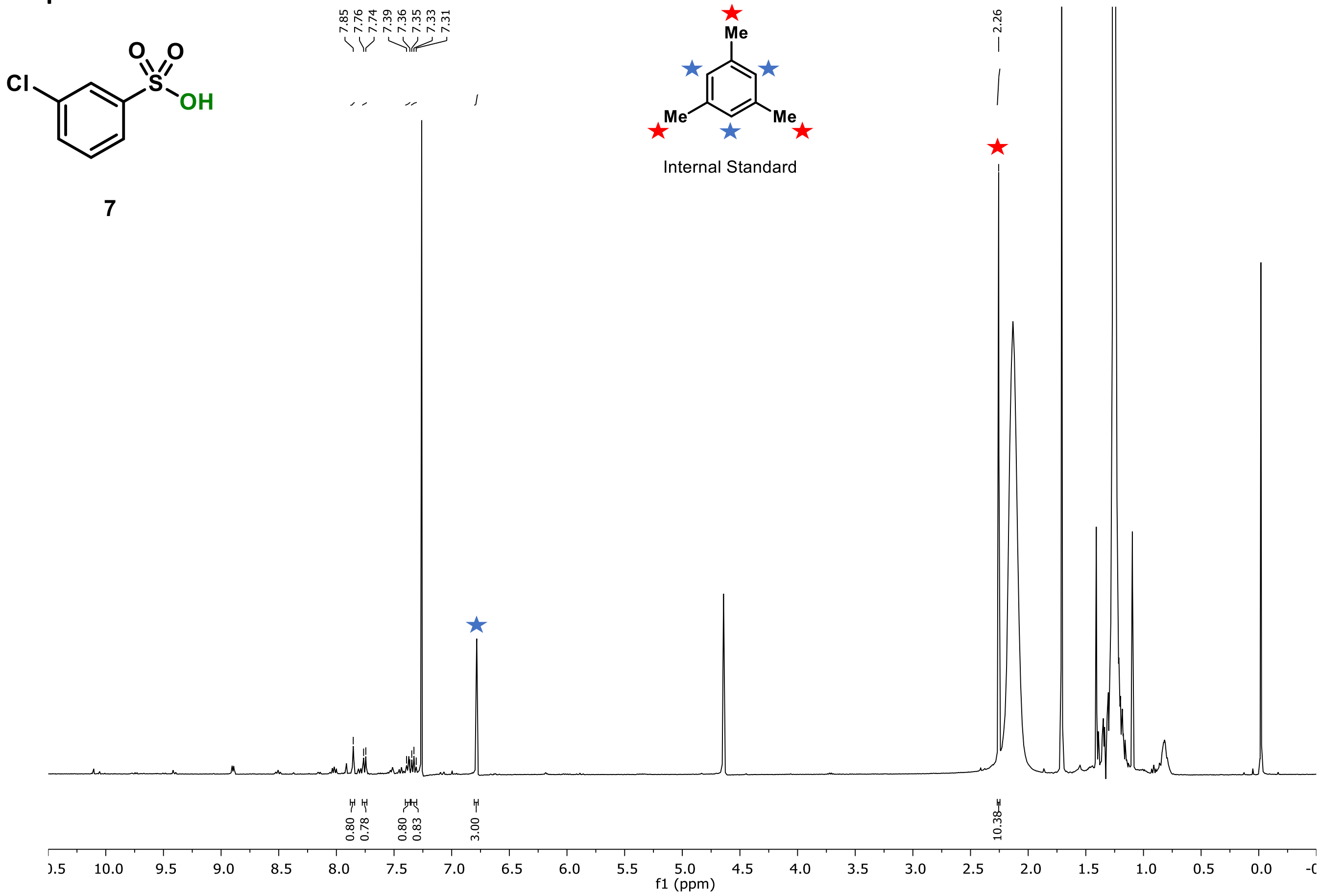

7

[1]H Spectra

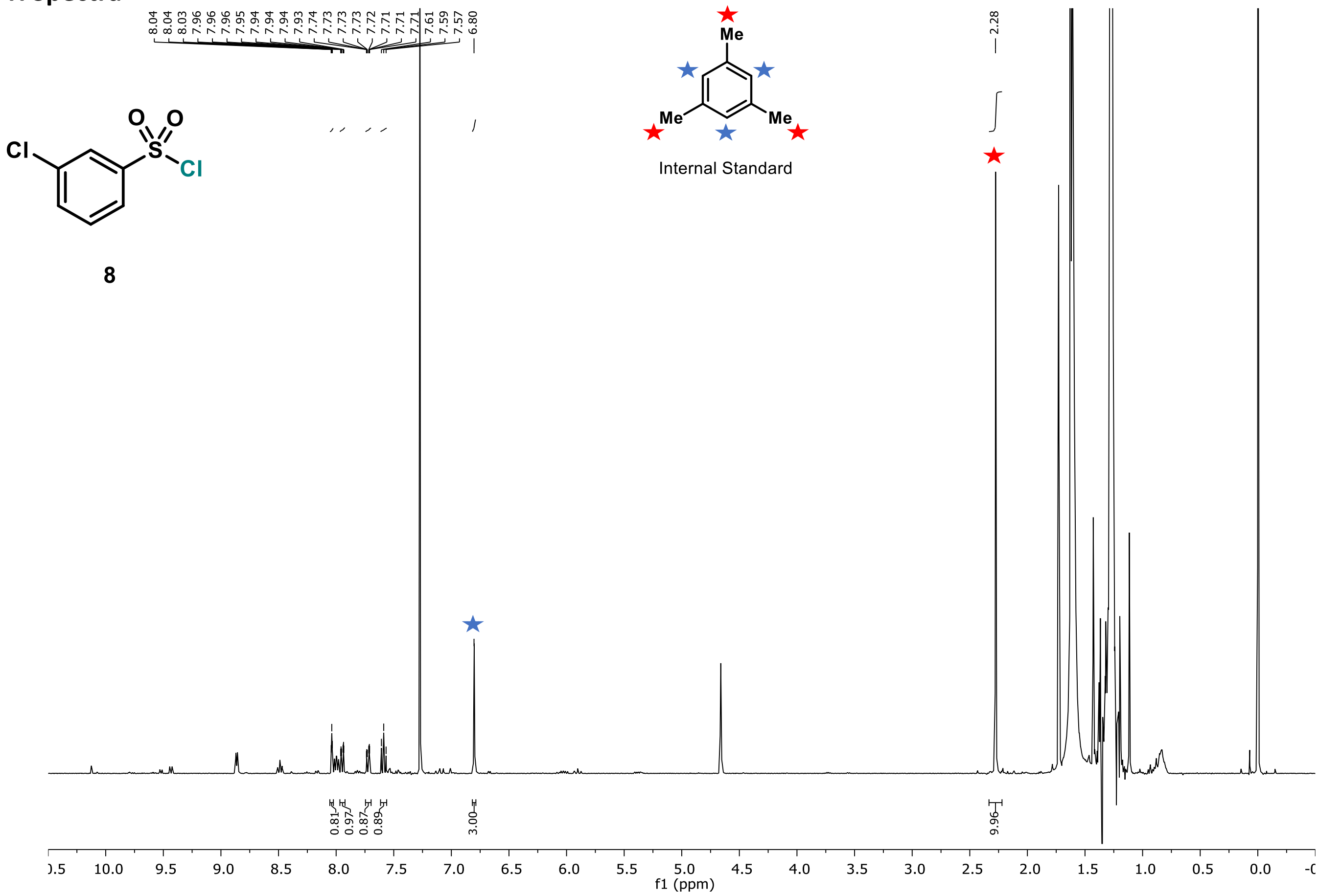

8

^{1}H Spectra

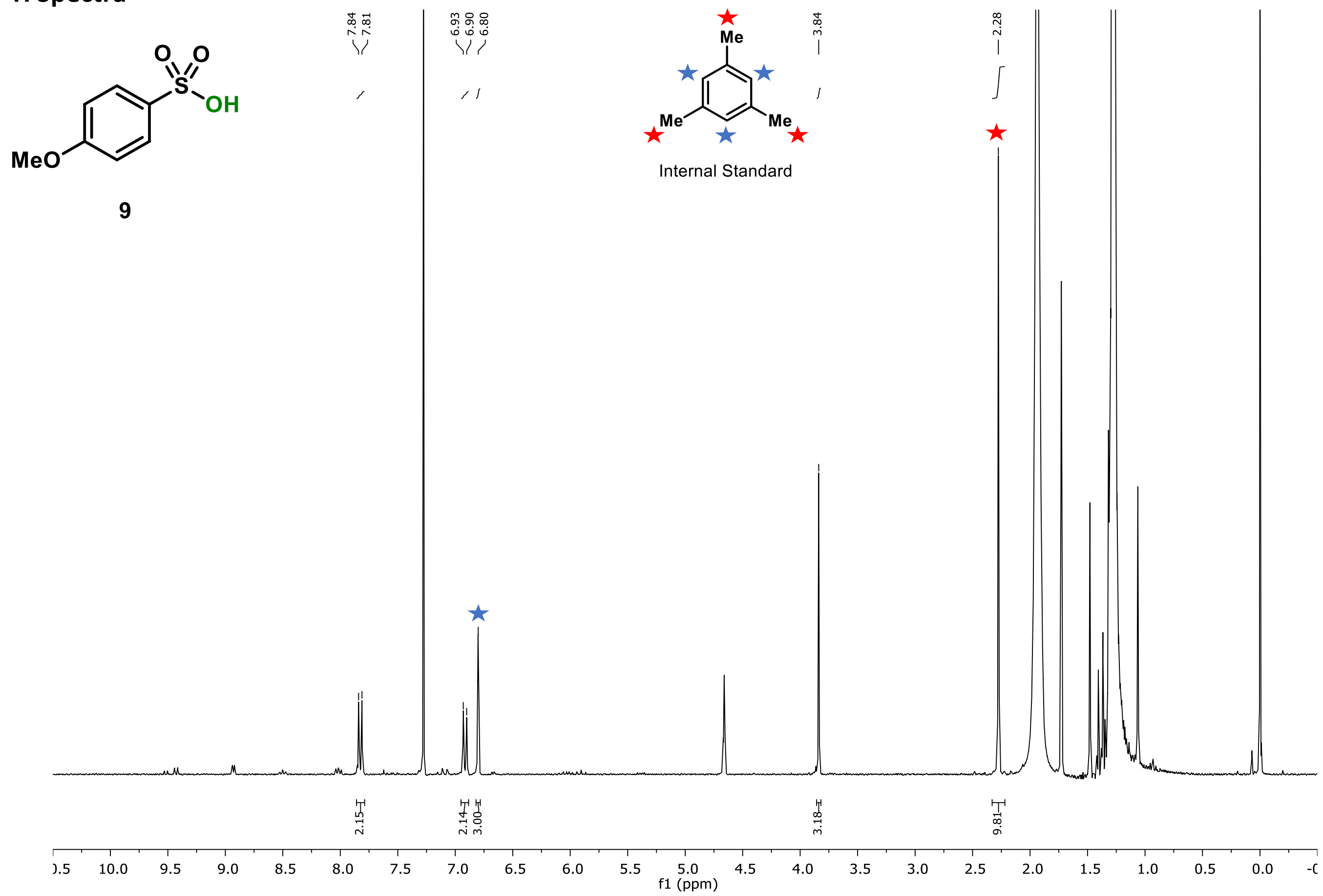

^{1}H Spectra

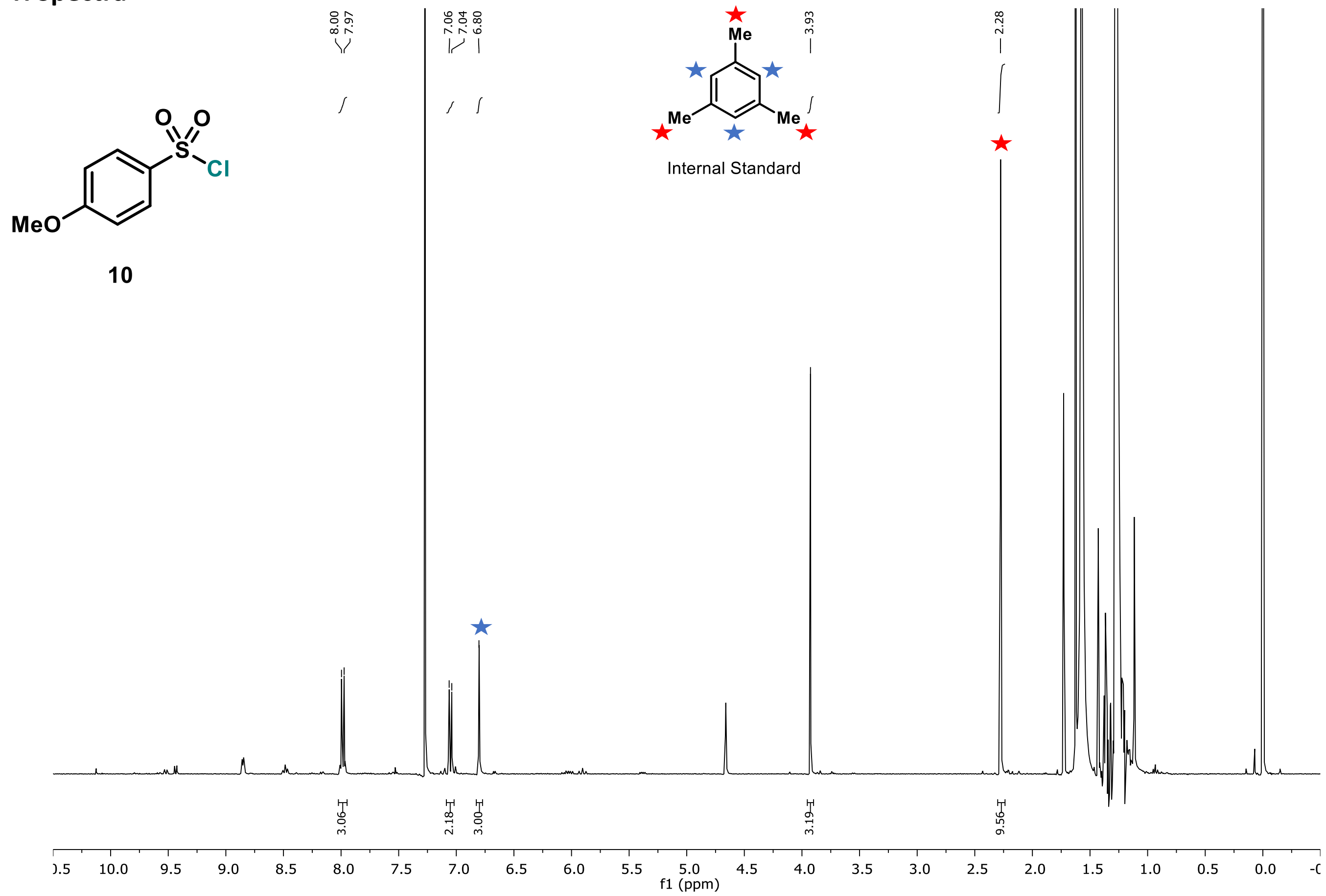

^{1}H Spectra

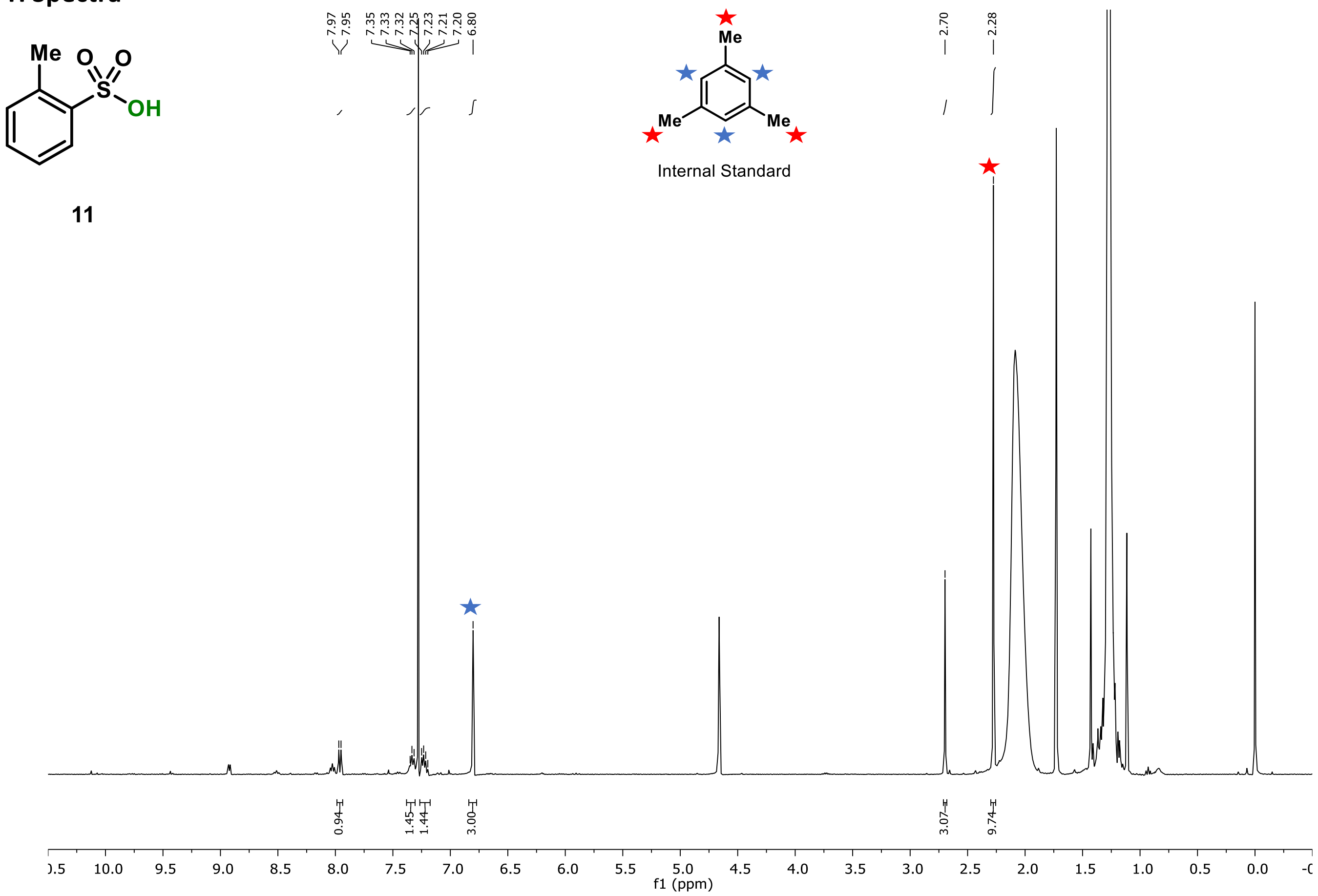

^{1}H Spectra

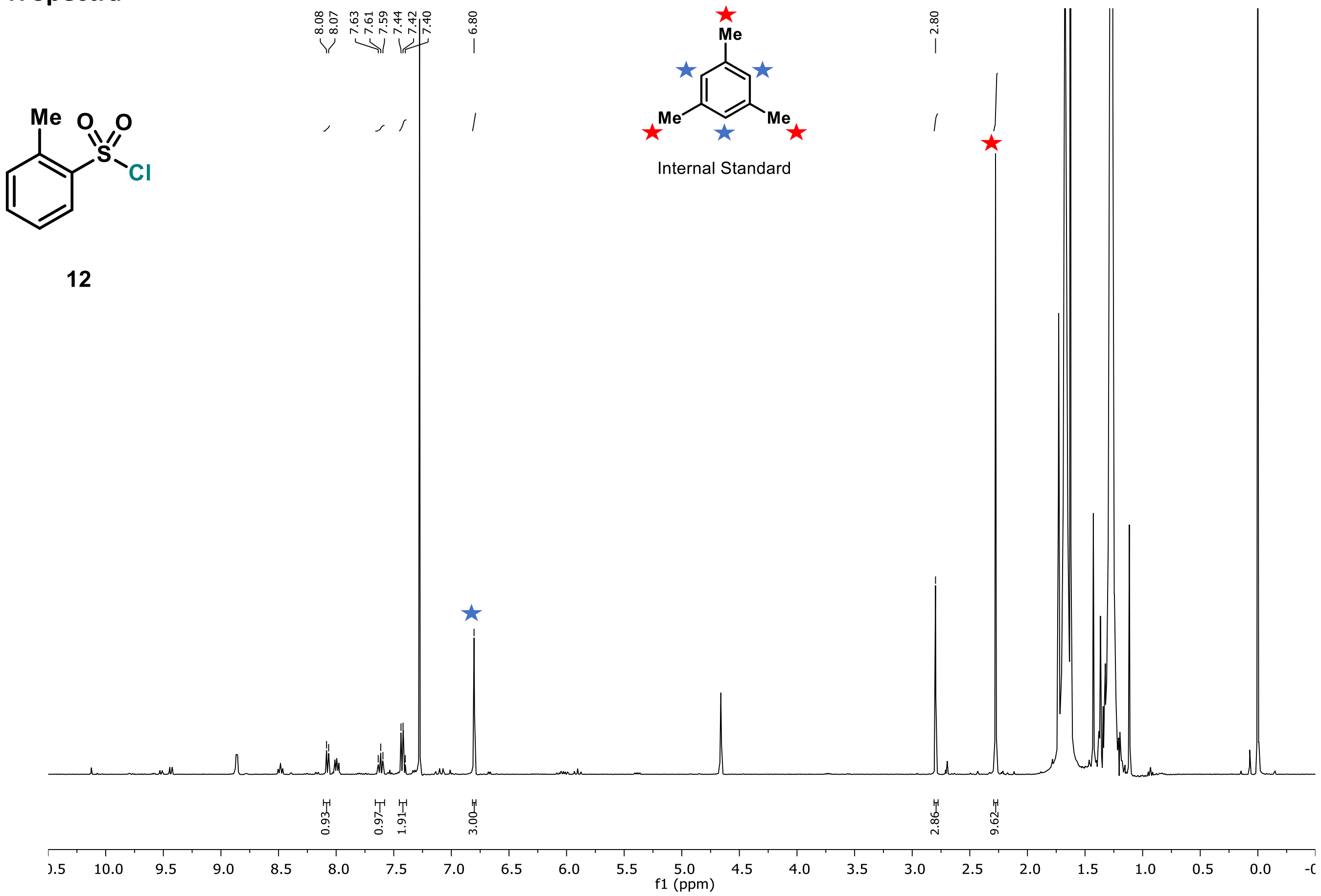

^{1}H Spectra

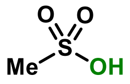

13

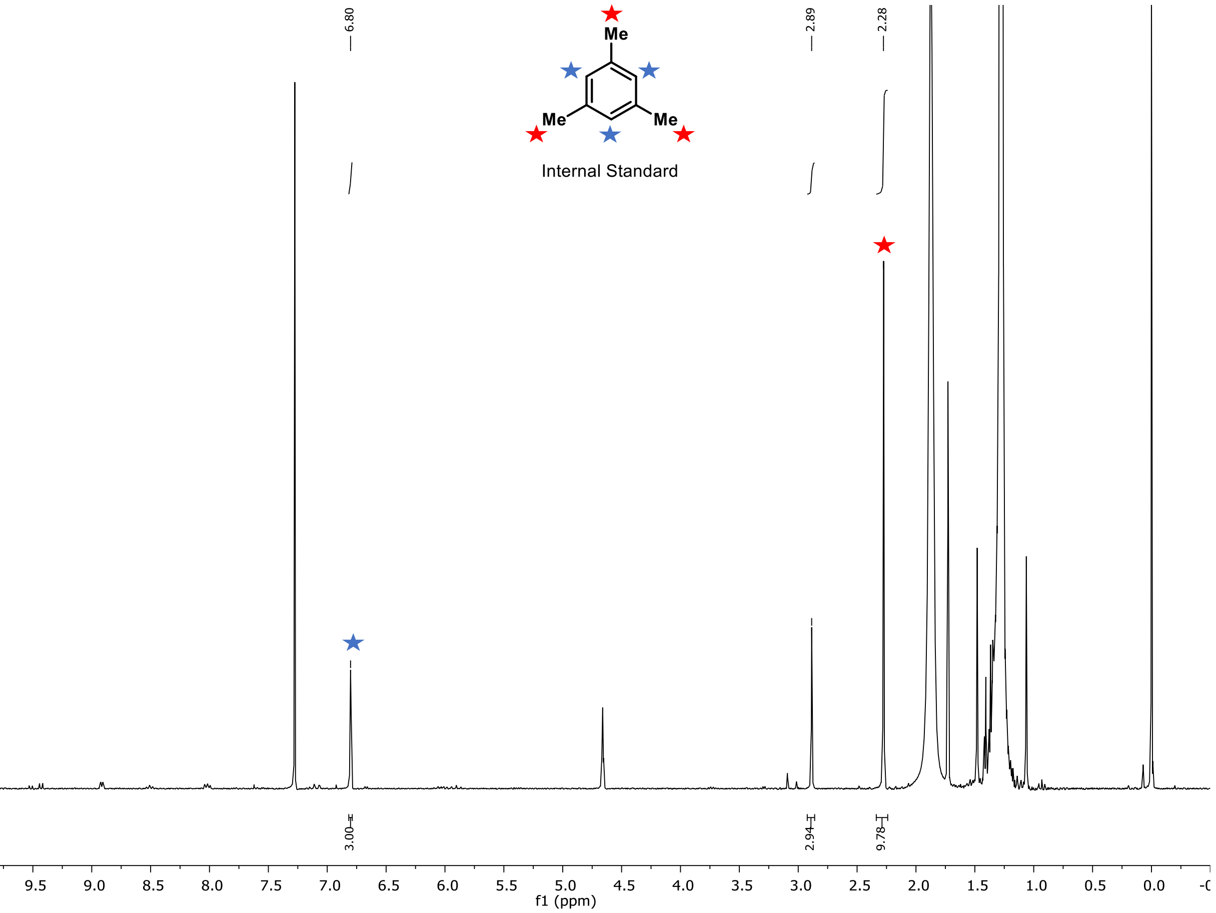

^{1}H Spectra

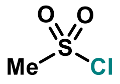

14

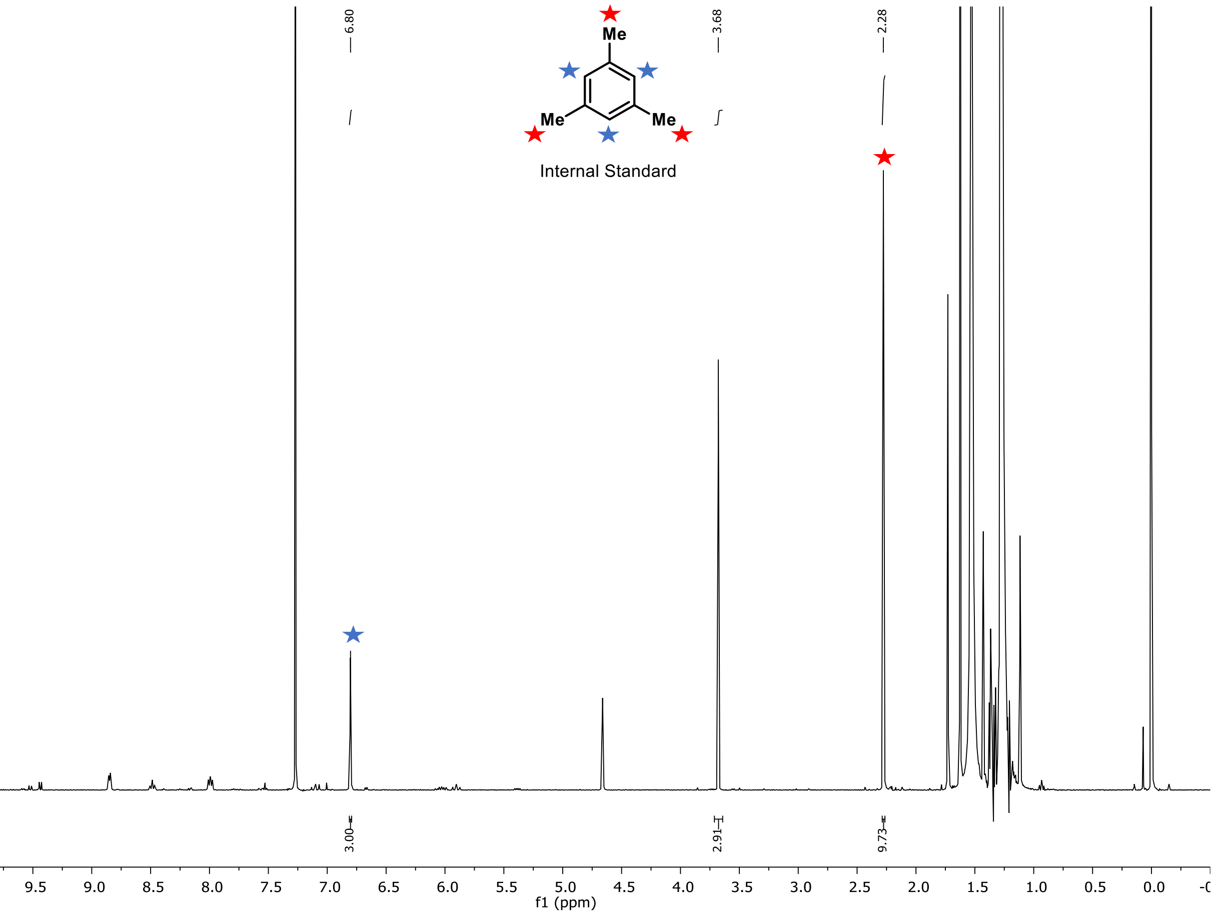

^{1}H Spectra

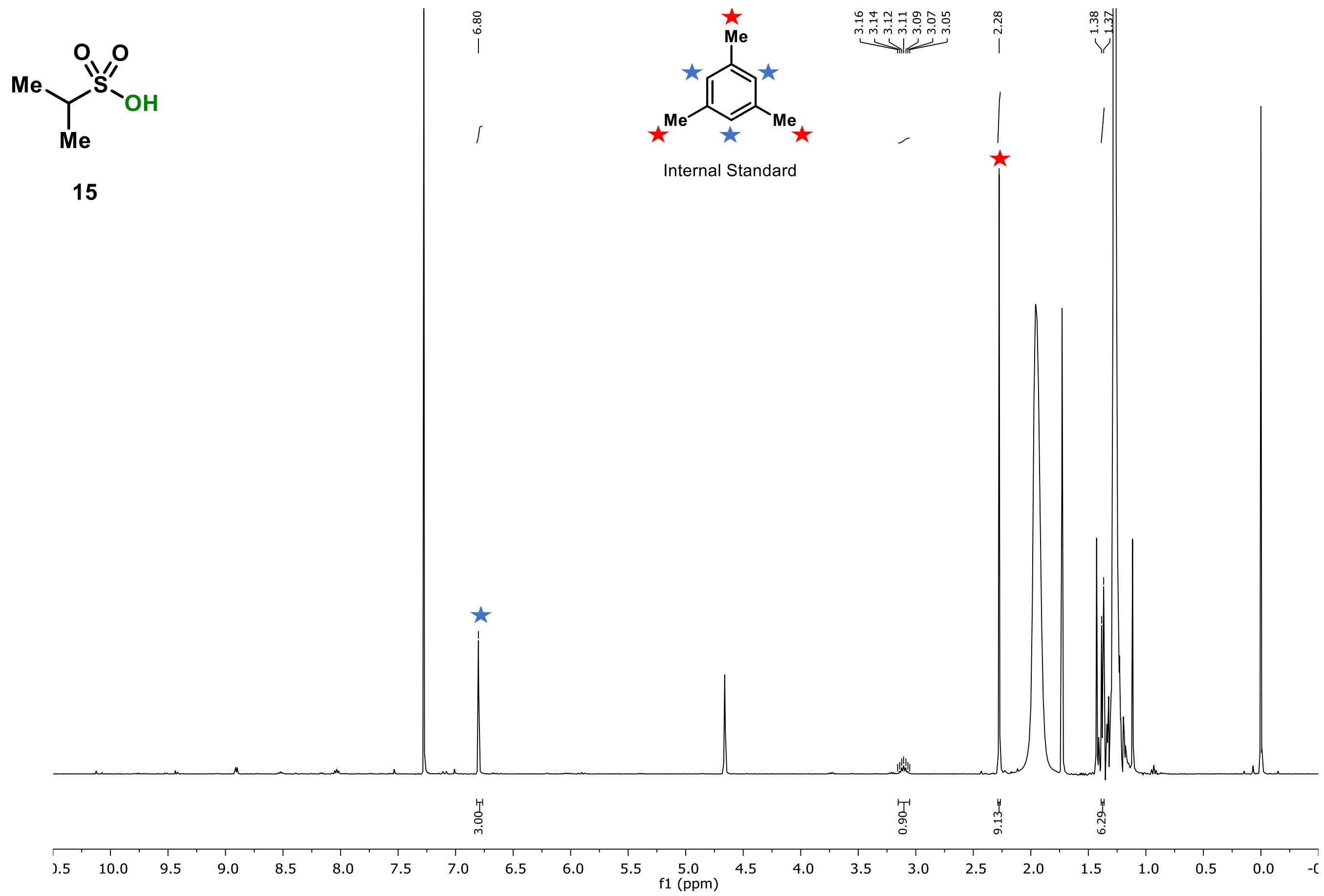

^{1}H Spectra

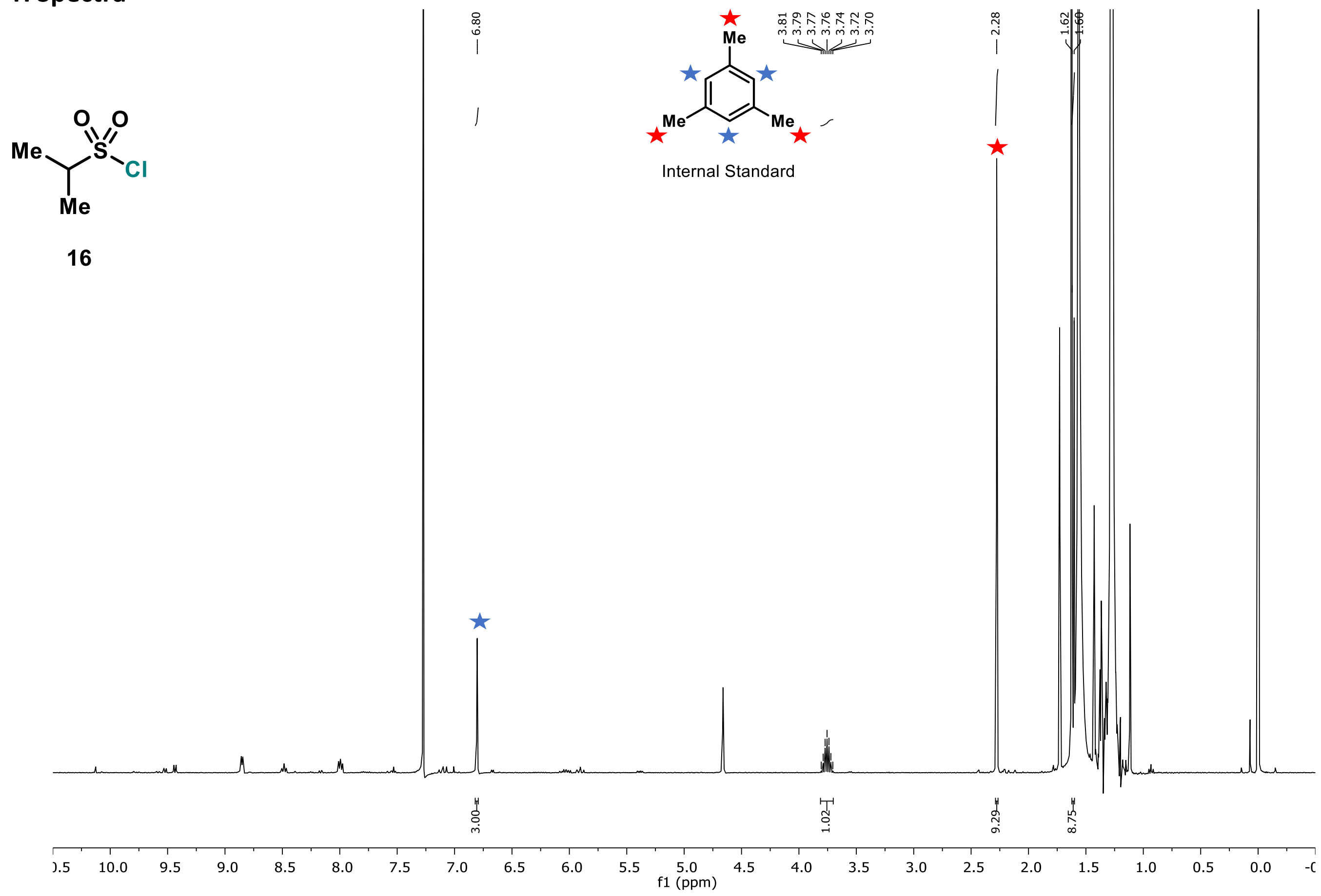

^{1}H Spectra

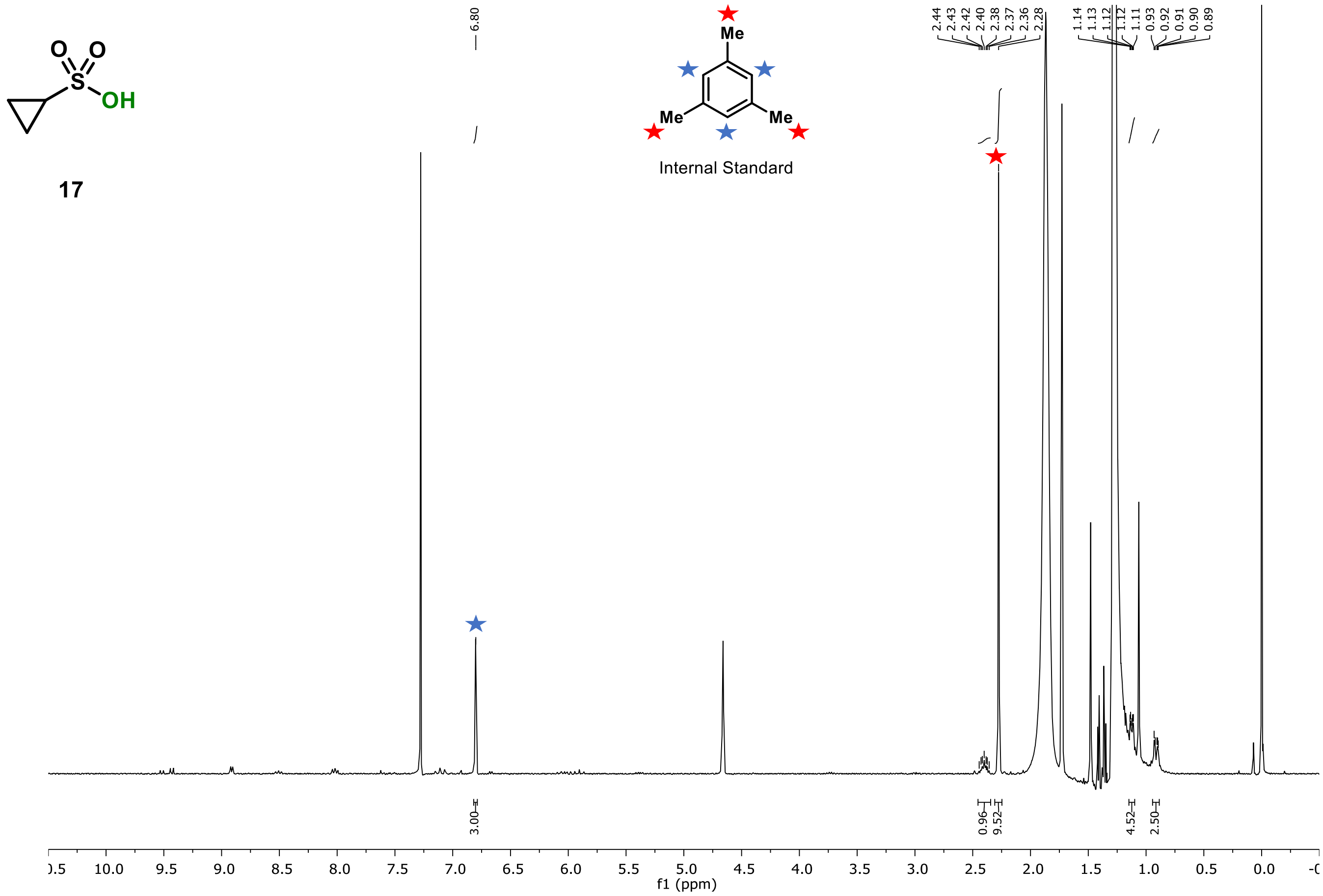

17

^{1}H Spectra

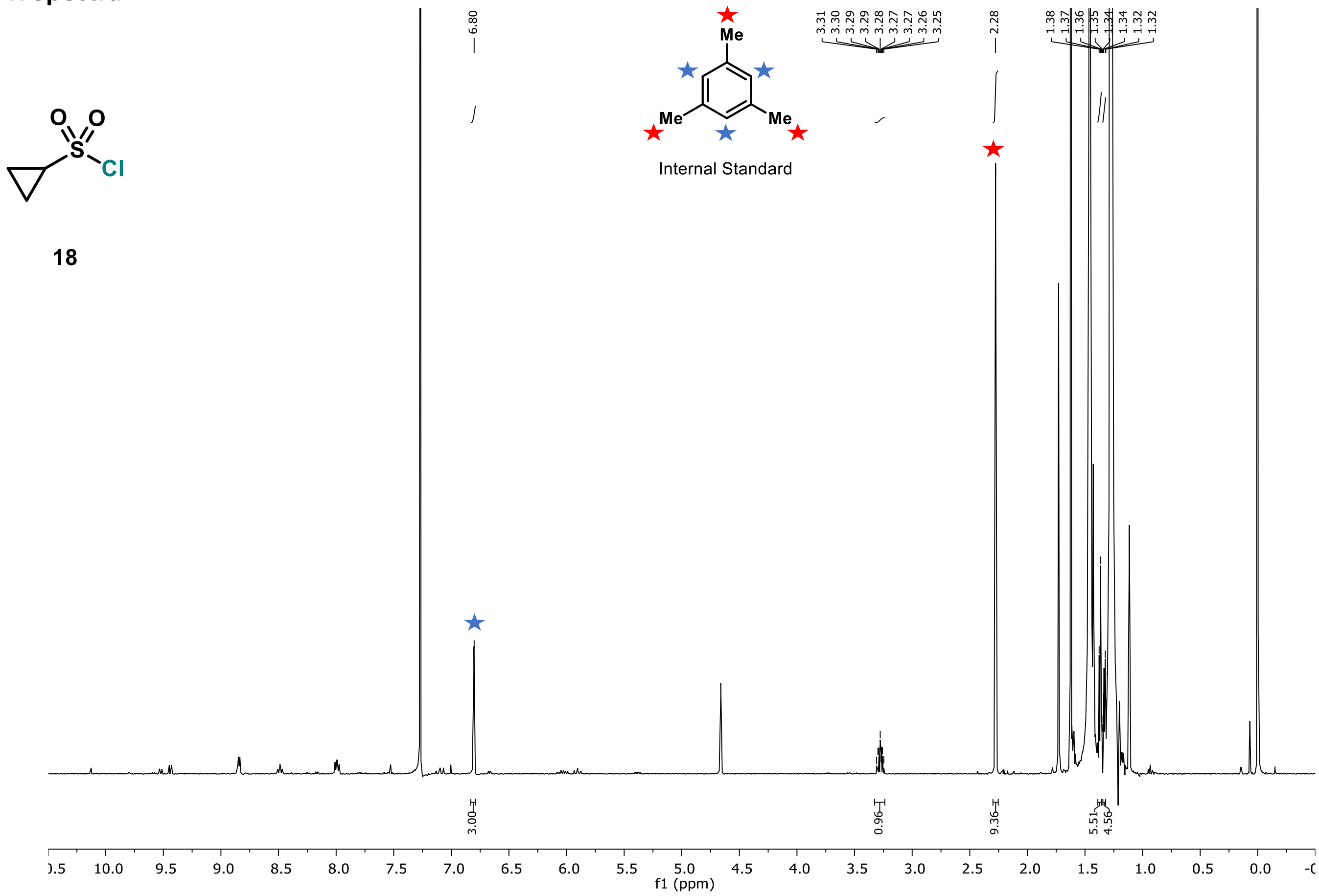

18

^{1}H Spectra

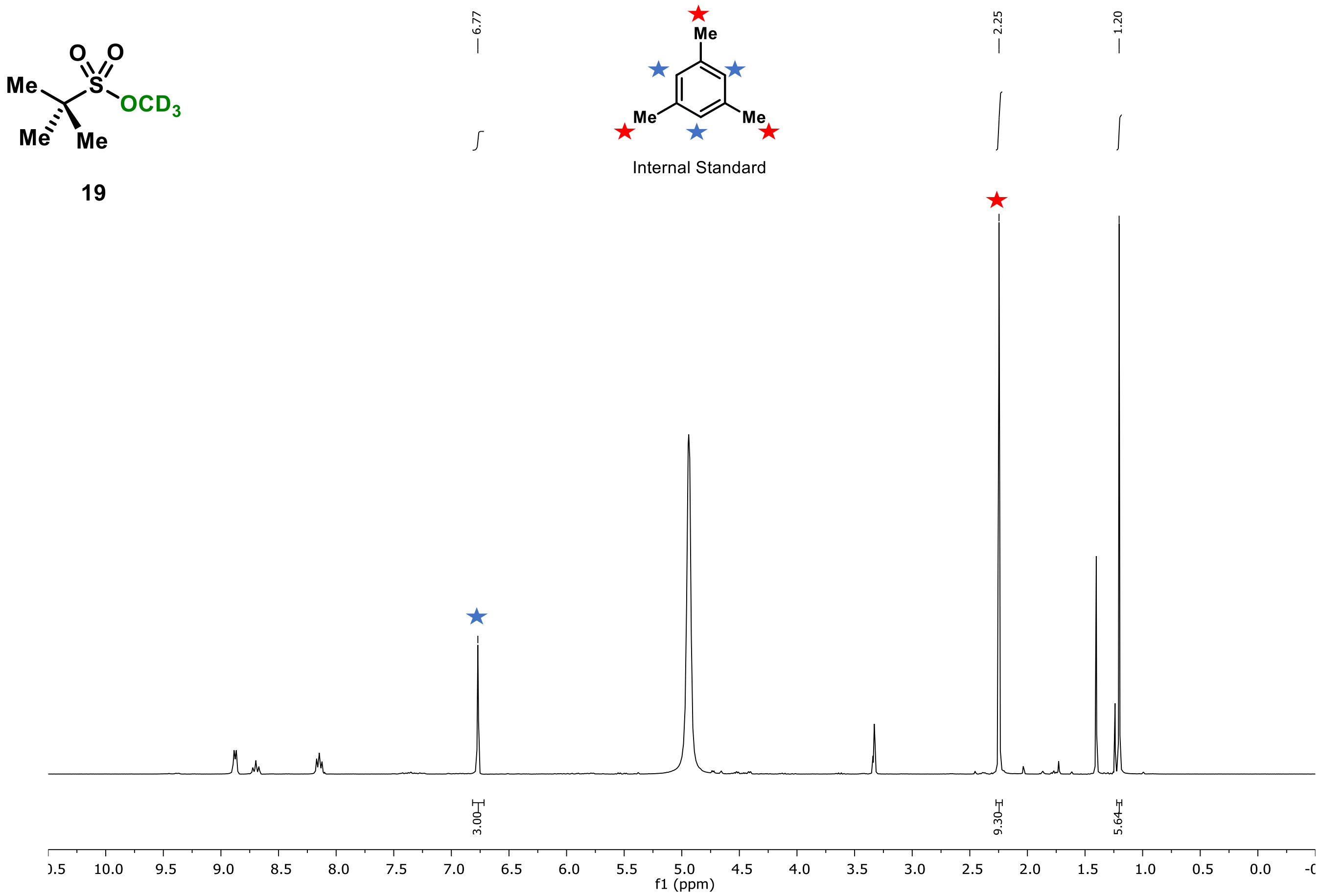

Me
Me
Me
OCD3
19
6.77
2.25
1.20
Internal Standard
3.00
9.30
5.64
f1 (ppm)

[1]H Spectra

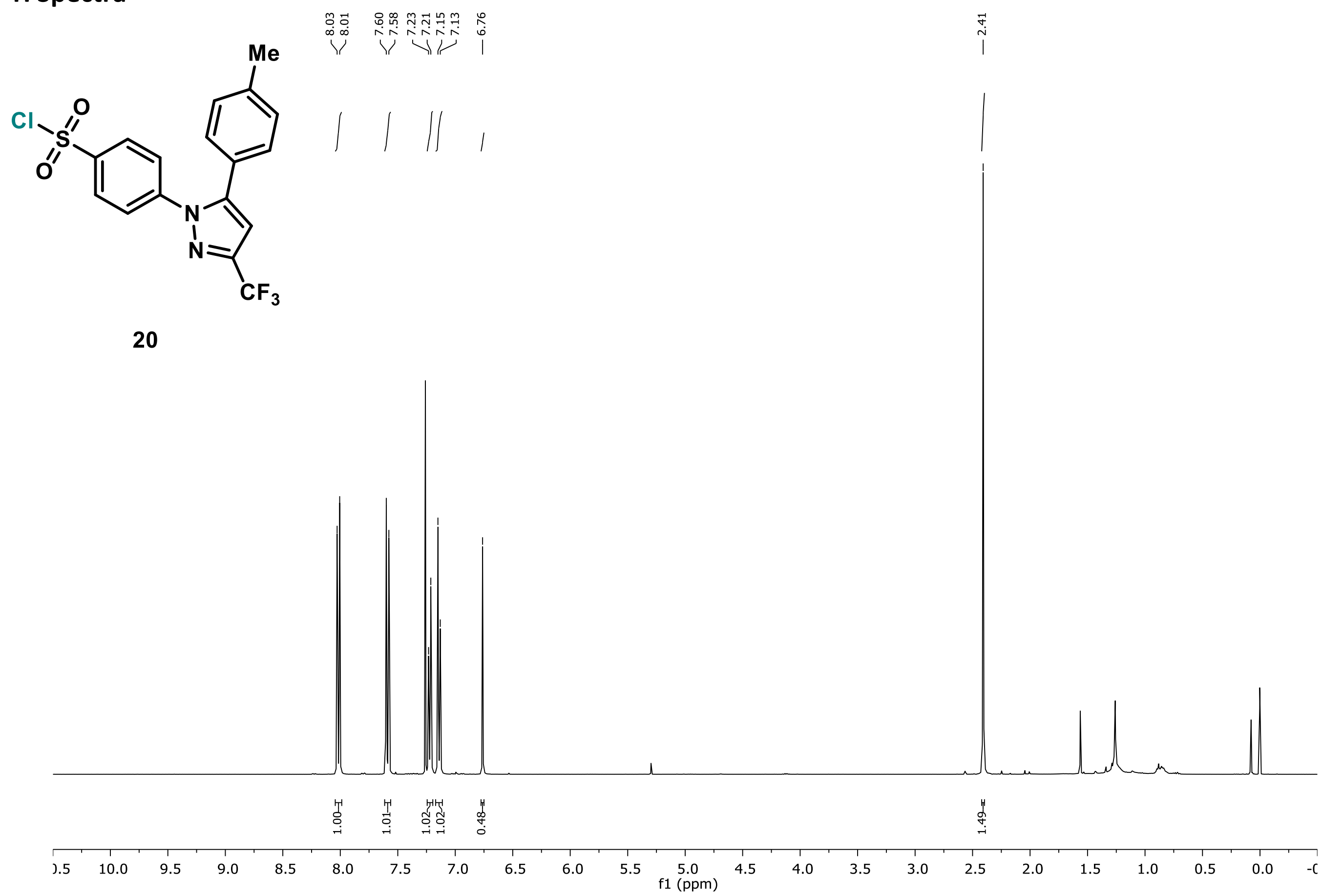

[13]C Spectra

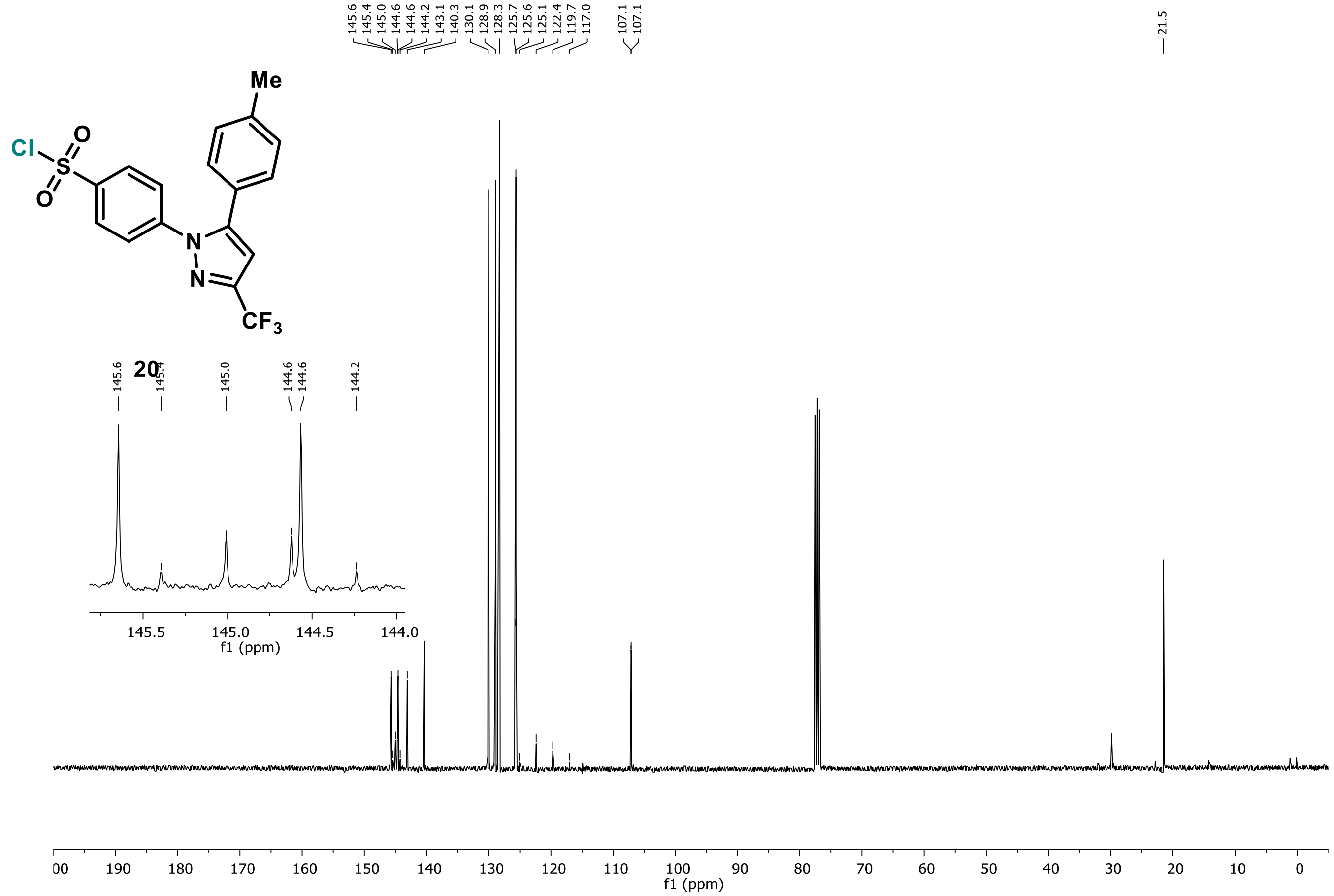

^{19}F Spectra

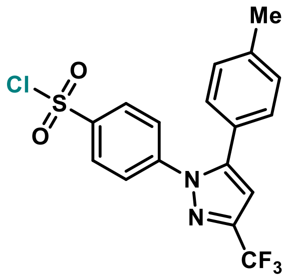

20

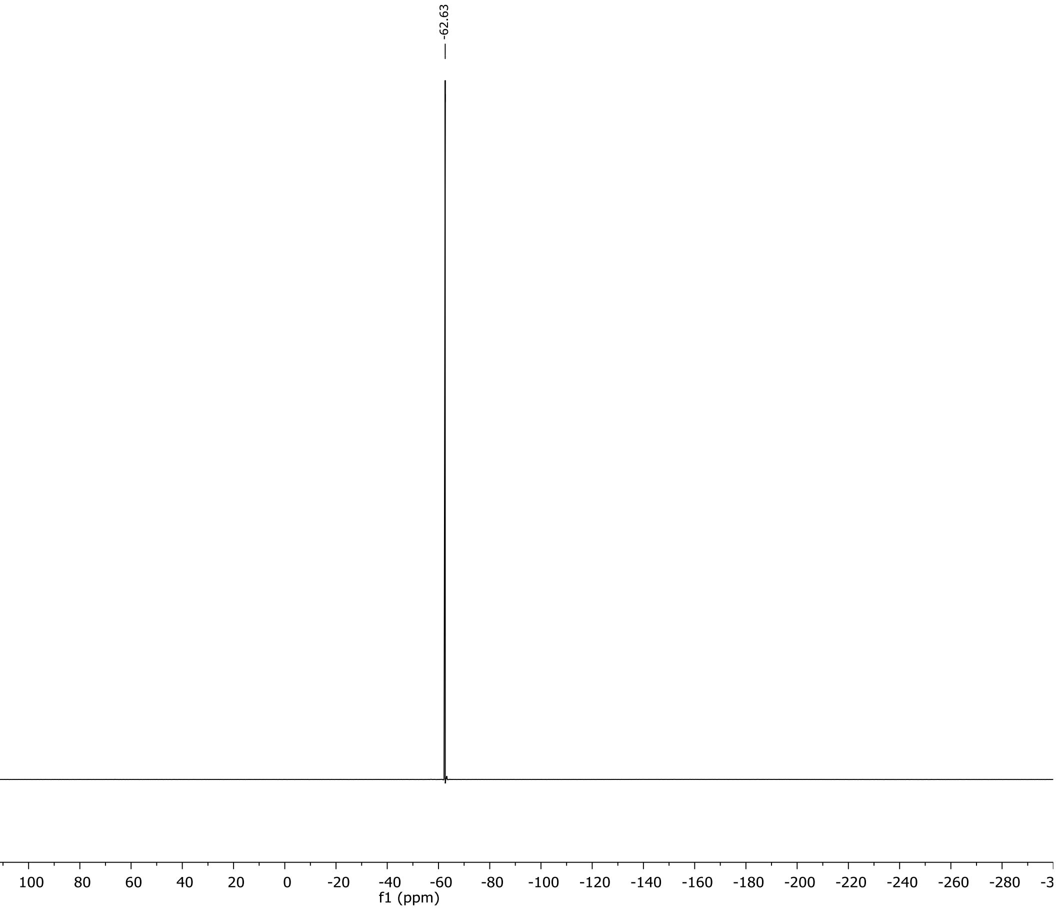

^{1}H Spectra

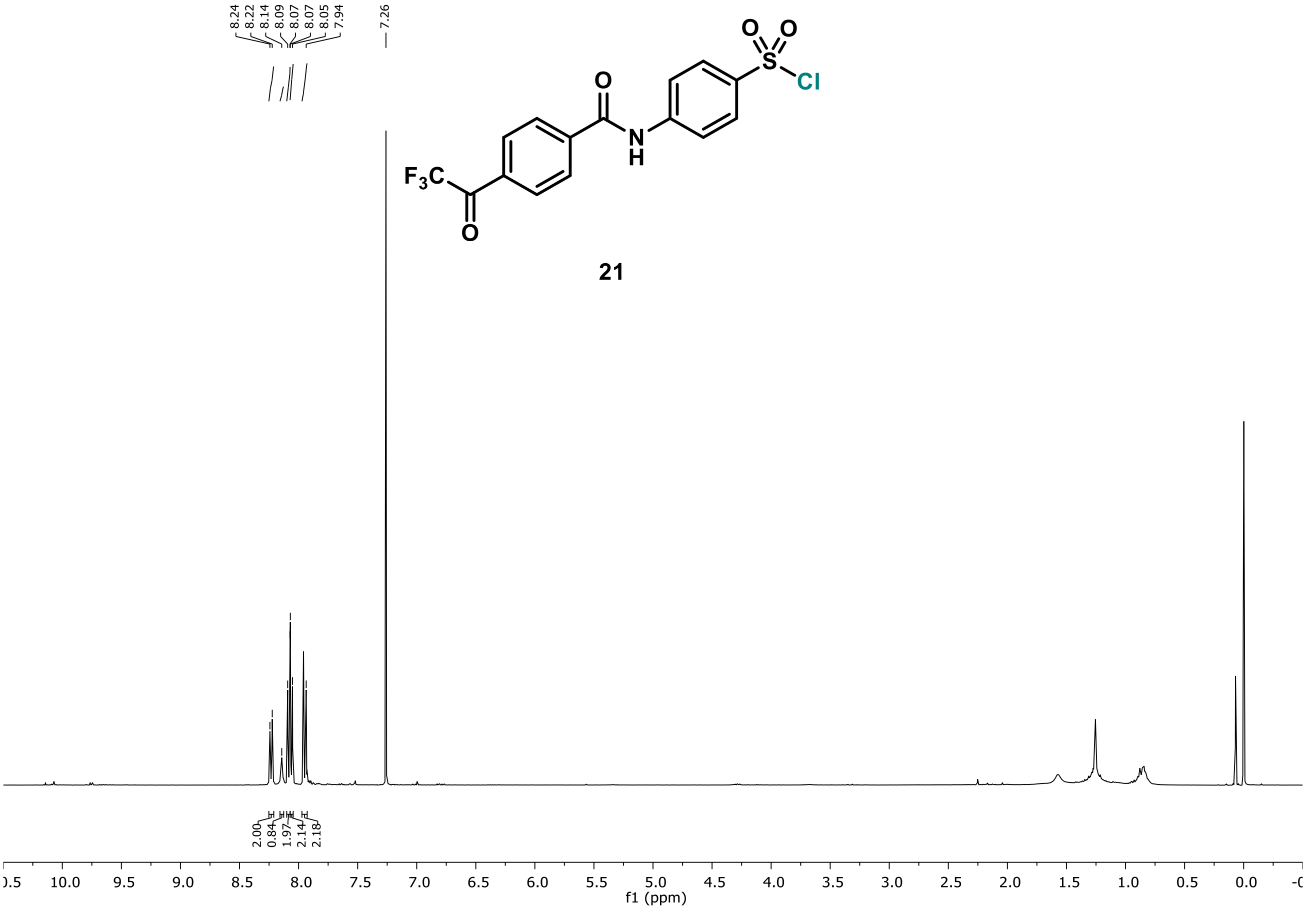

^{13}C Spectra

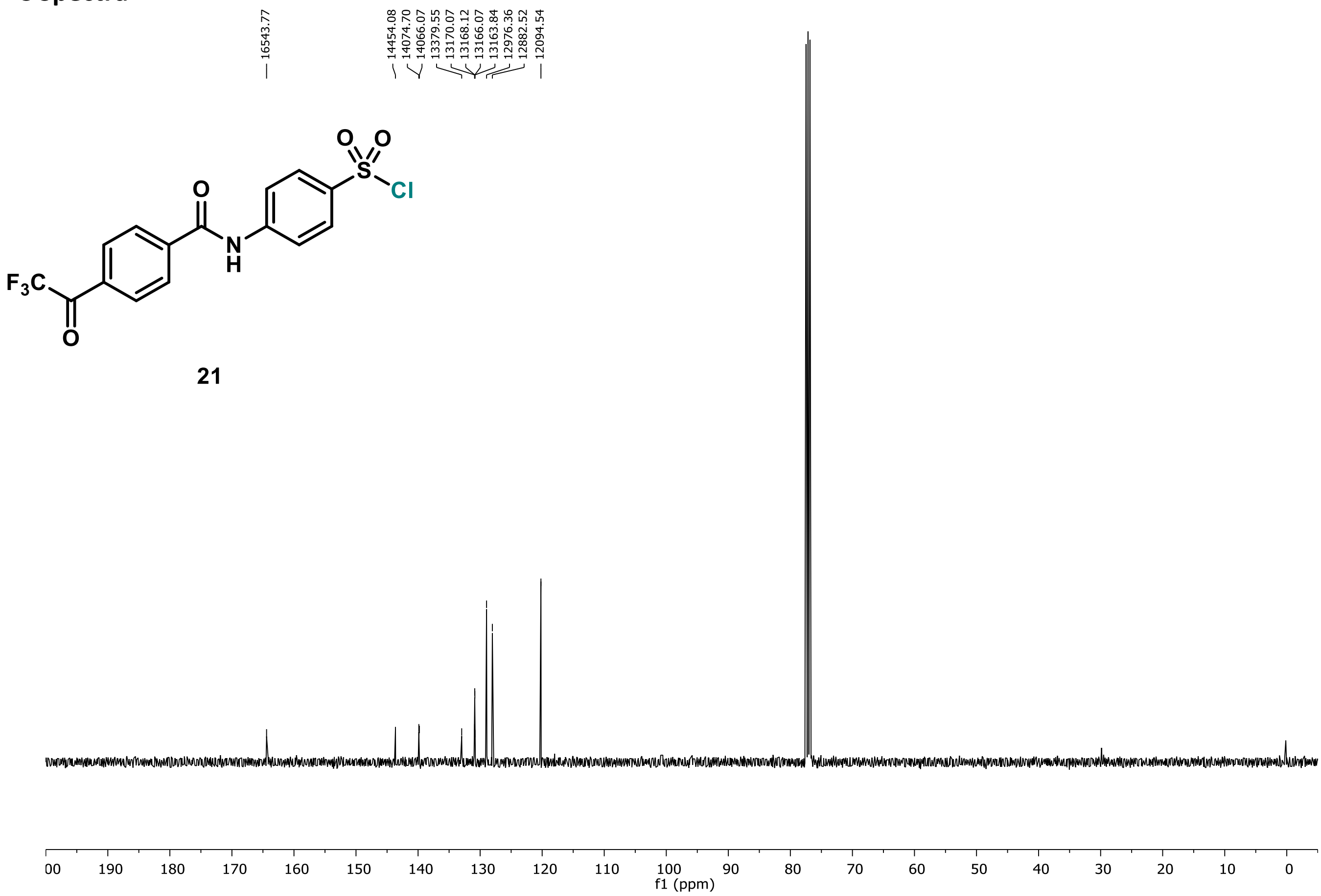

^{19}F Spectra

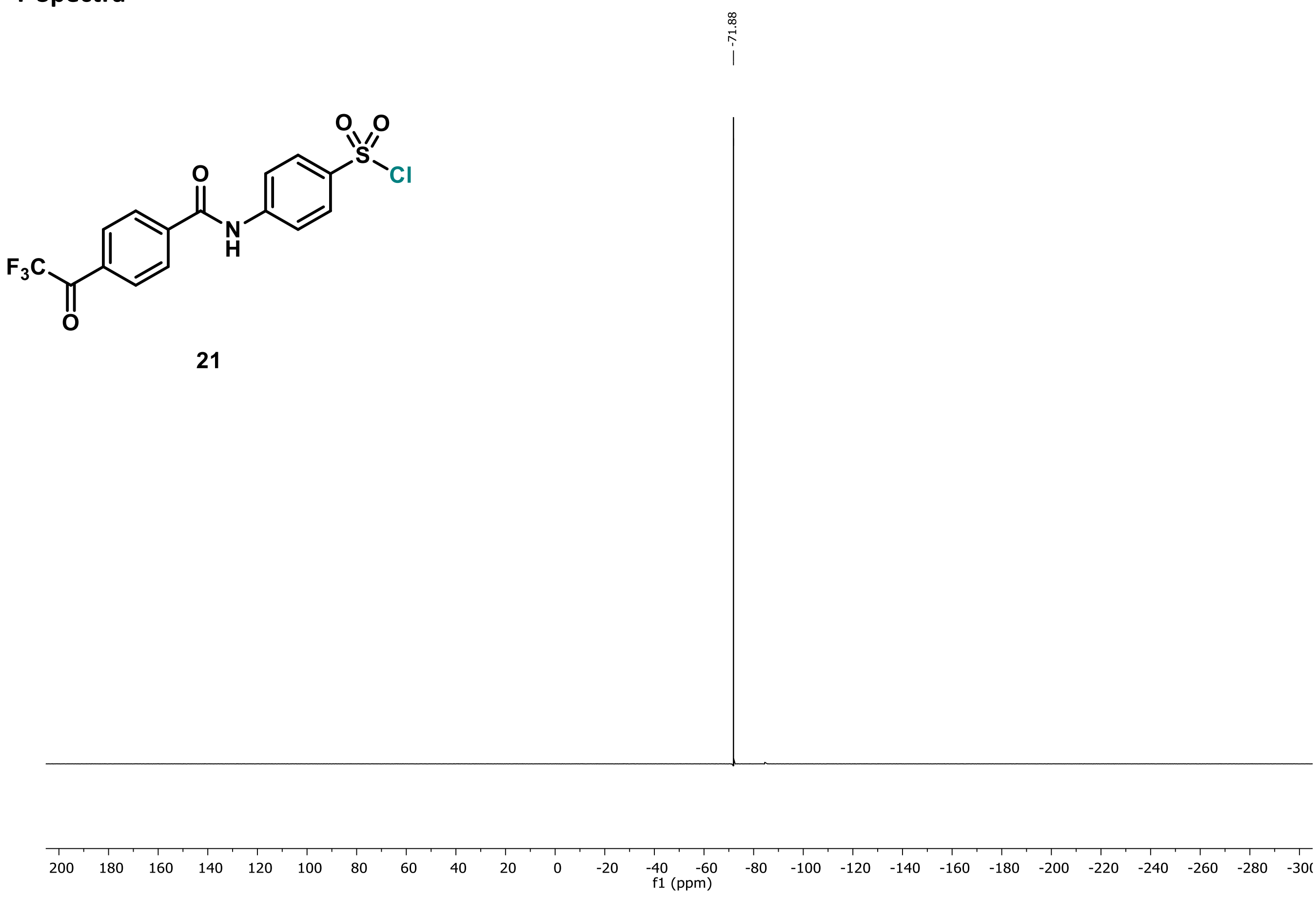

-71.88
O
O
S
Cl
O
N
H
F3C
O
21
200
180
160
140
120
100
80
60
40
20
0
-20
-40
-60
-80
-100
-120
-140
-160
-180
-200
-220
-240
-260
-280
f1 (ppm)

[1]H Spectra

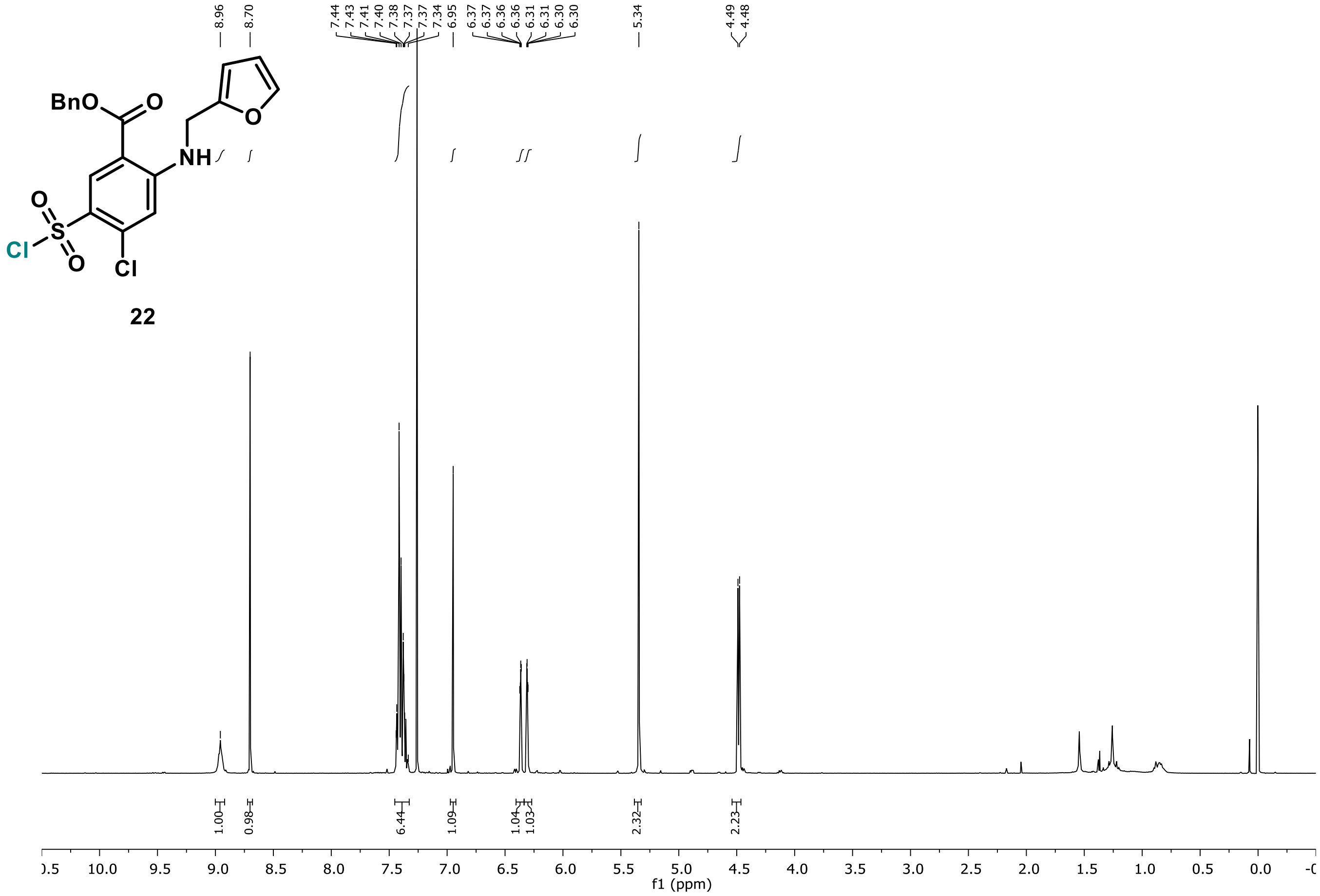

[13]C Spectra

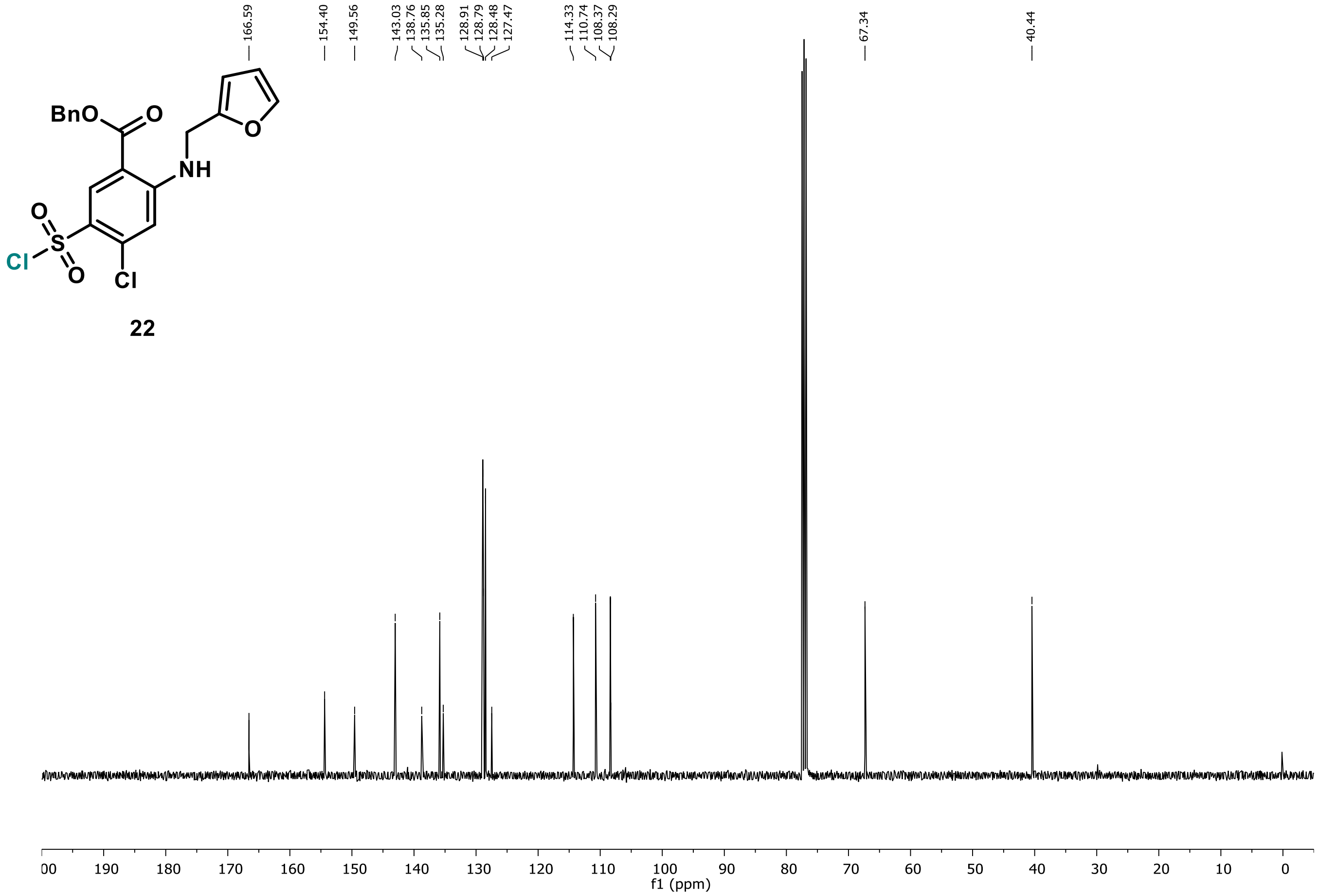

^{1}H Spectra

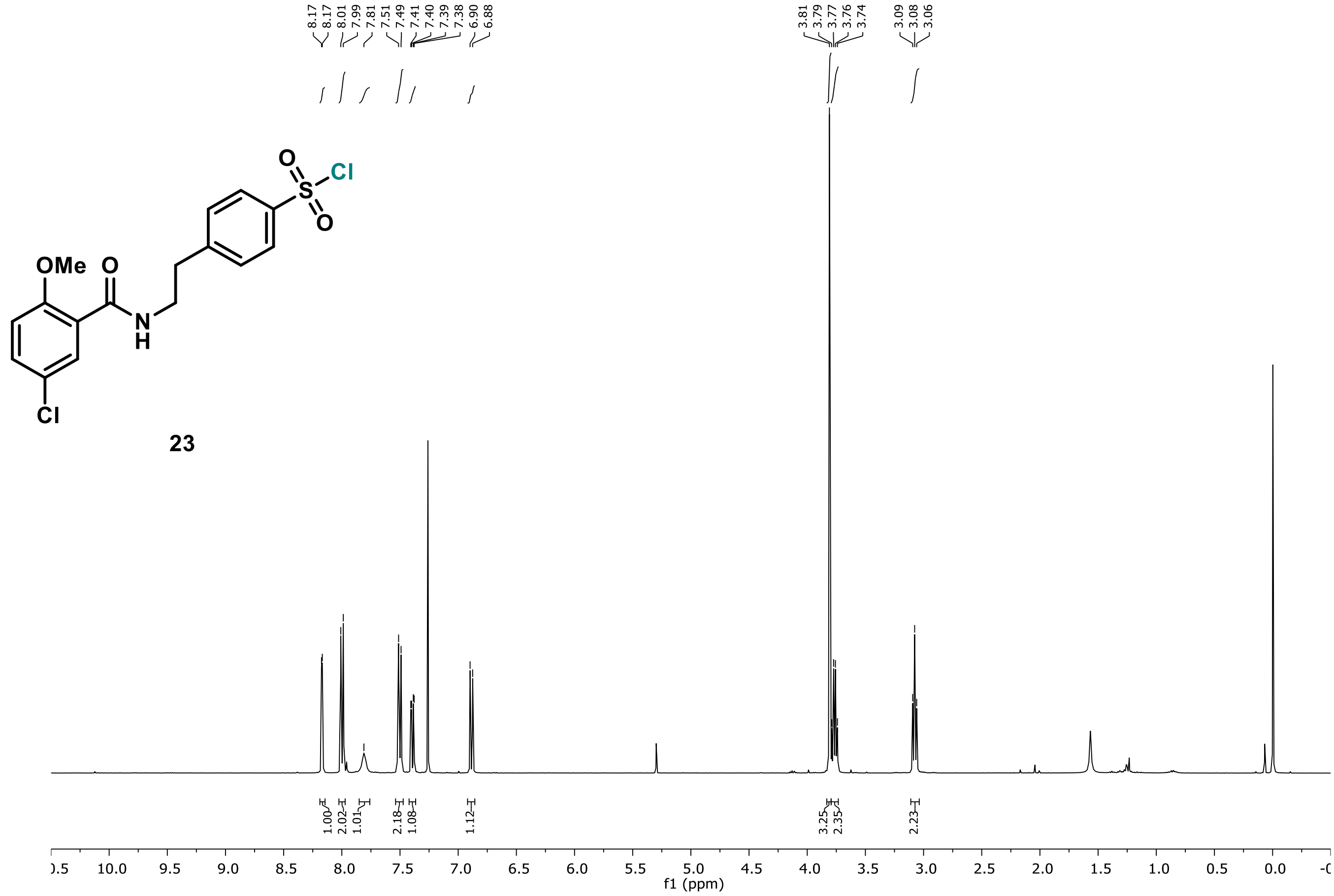

^{13}C Spectra

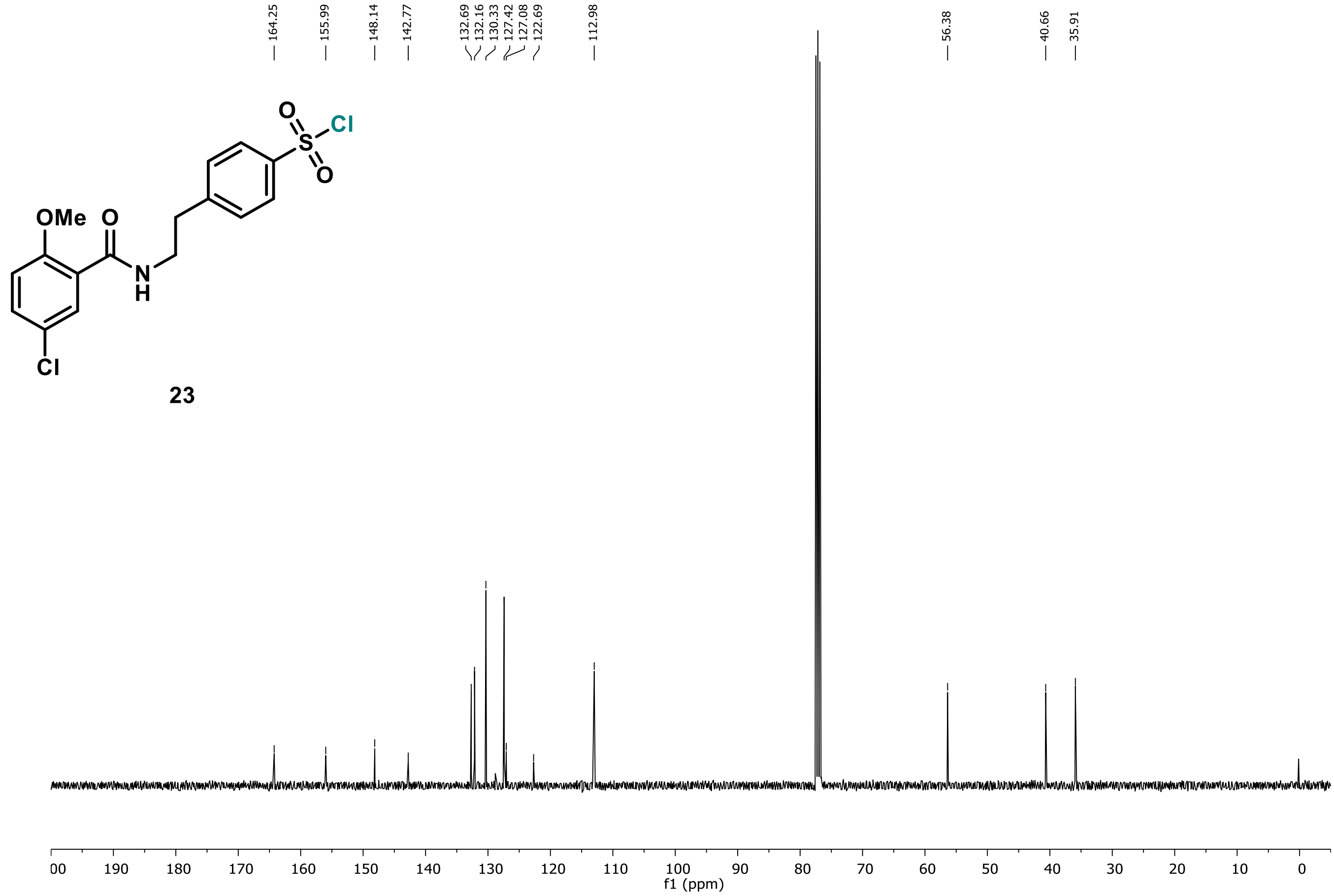

[1]H Spectra

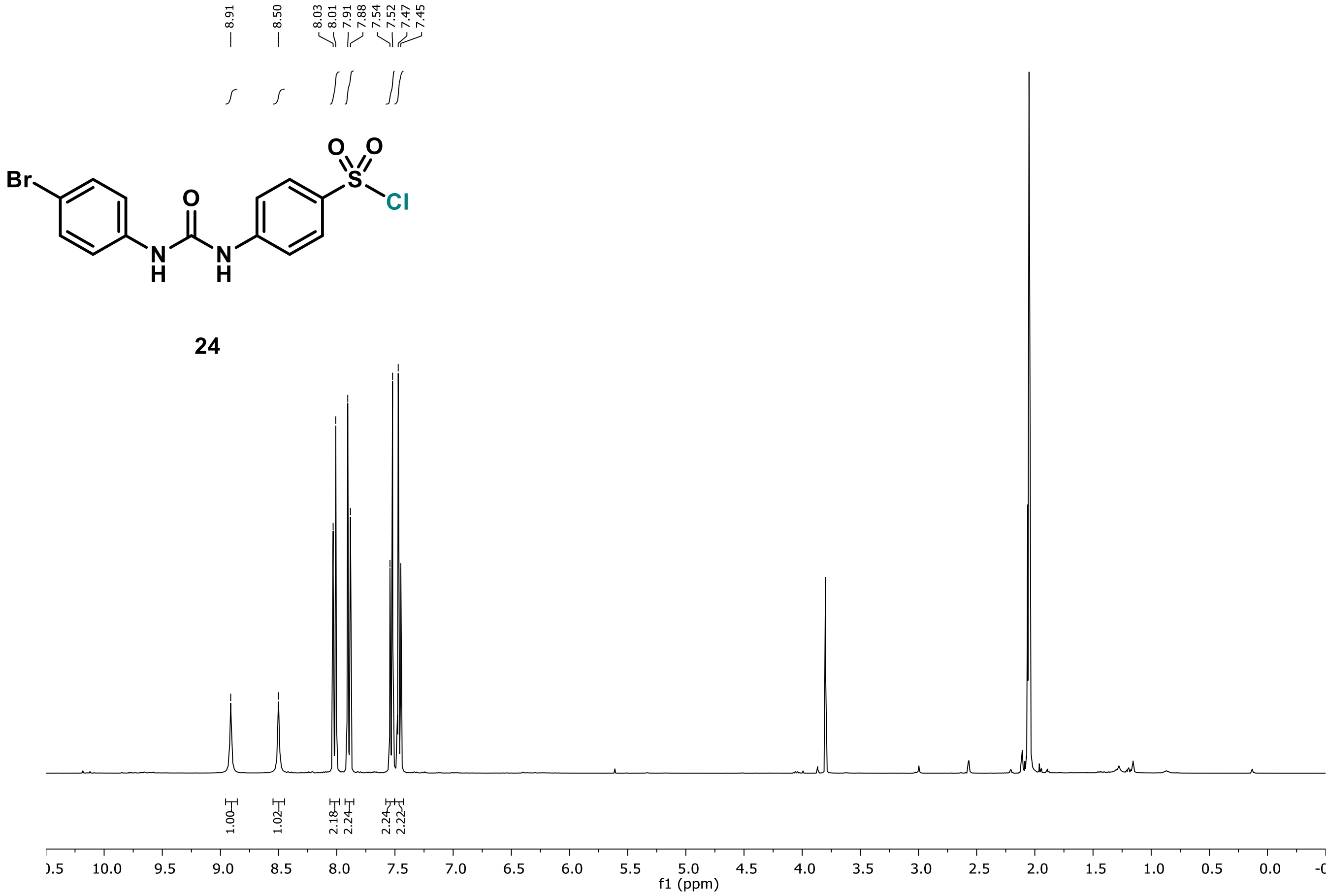

[13]C Spectra

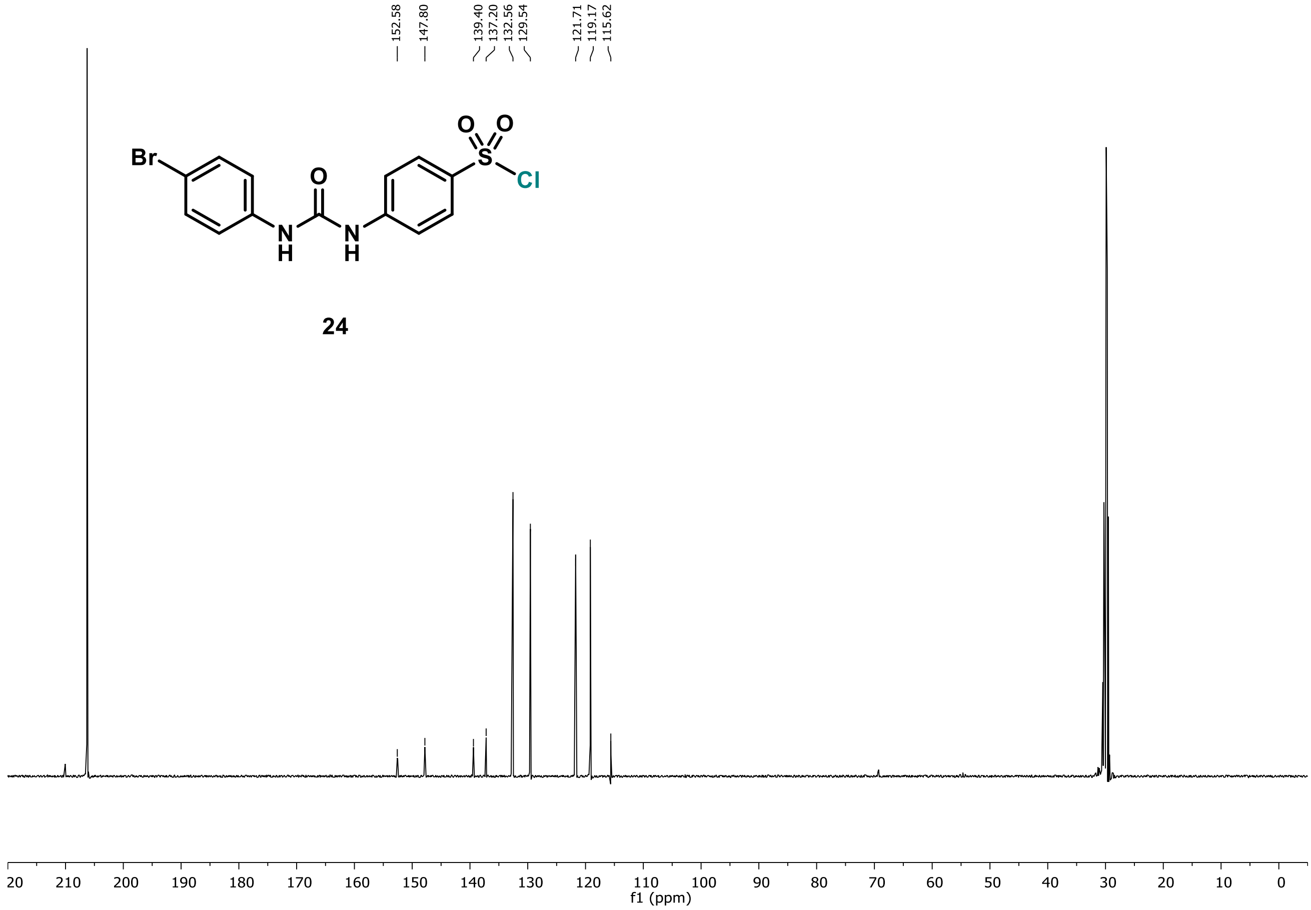

[1]H Spectra

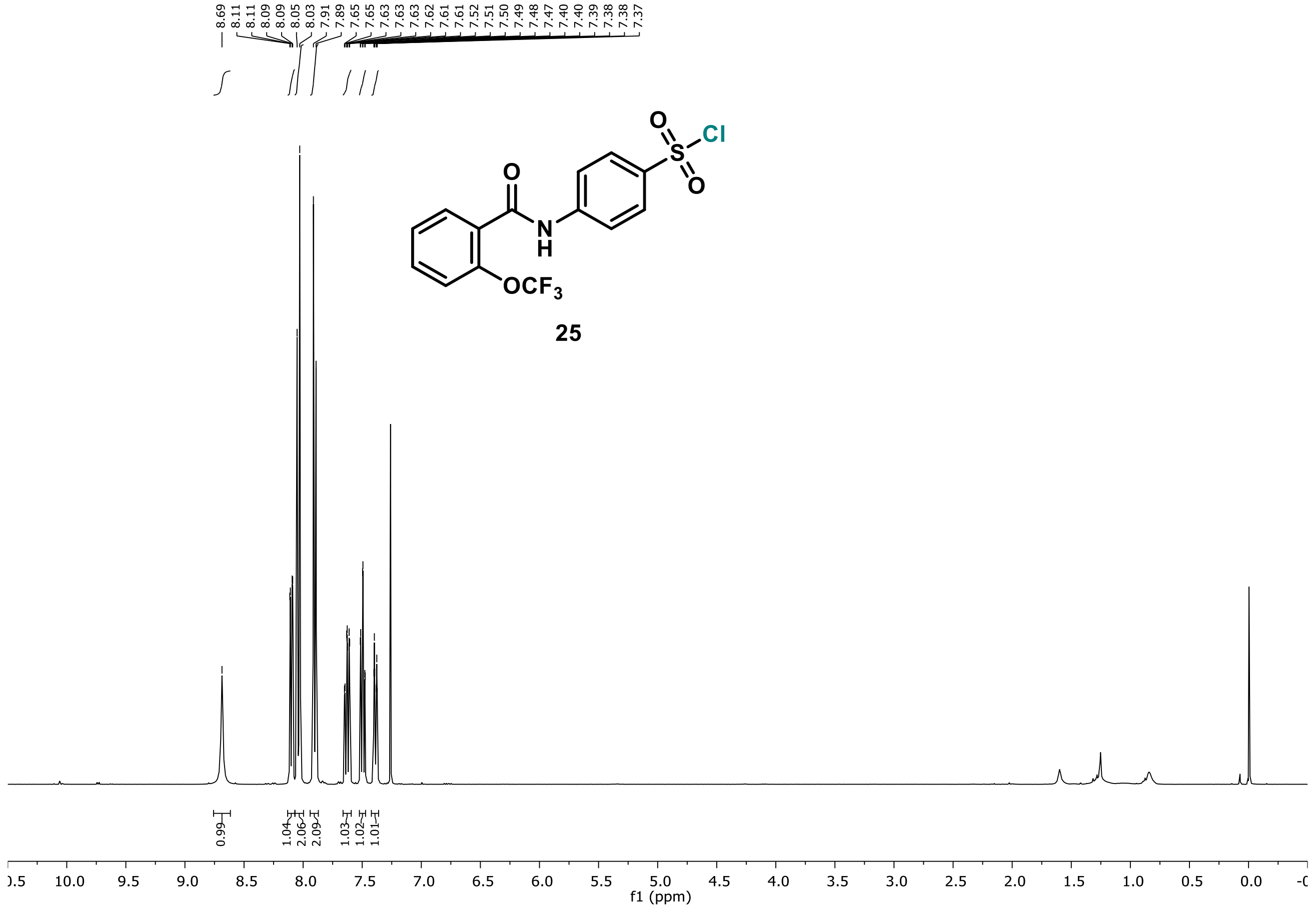

[13]C Spectra

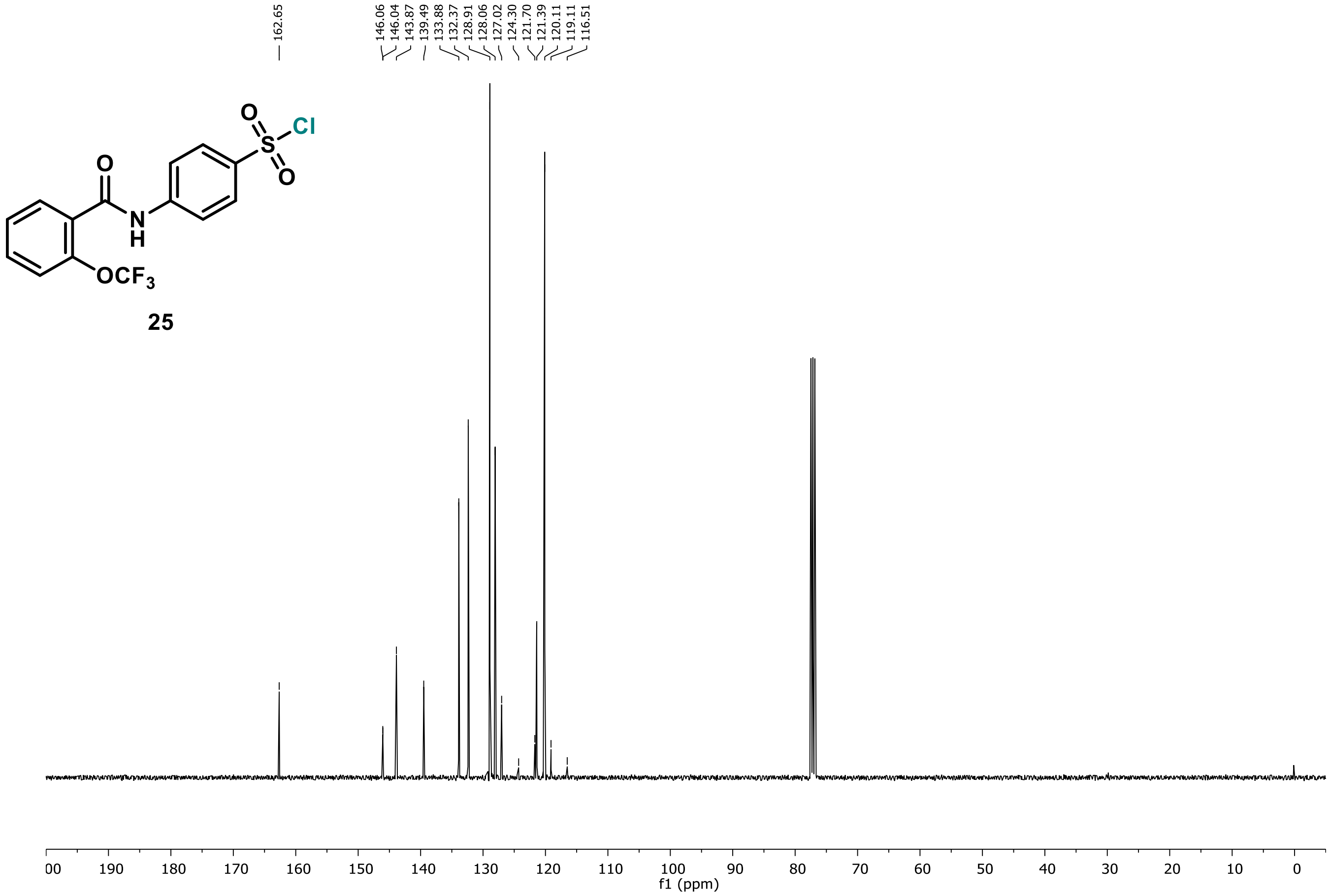

^{19}F Spectra

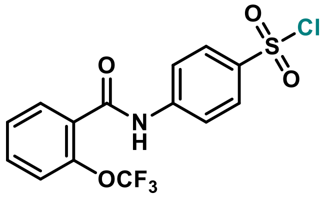

25

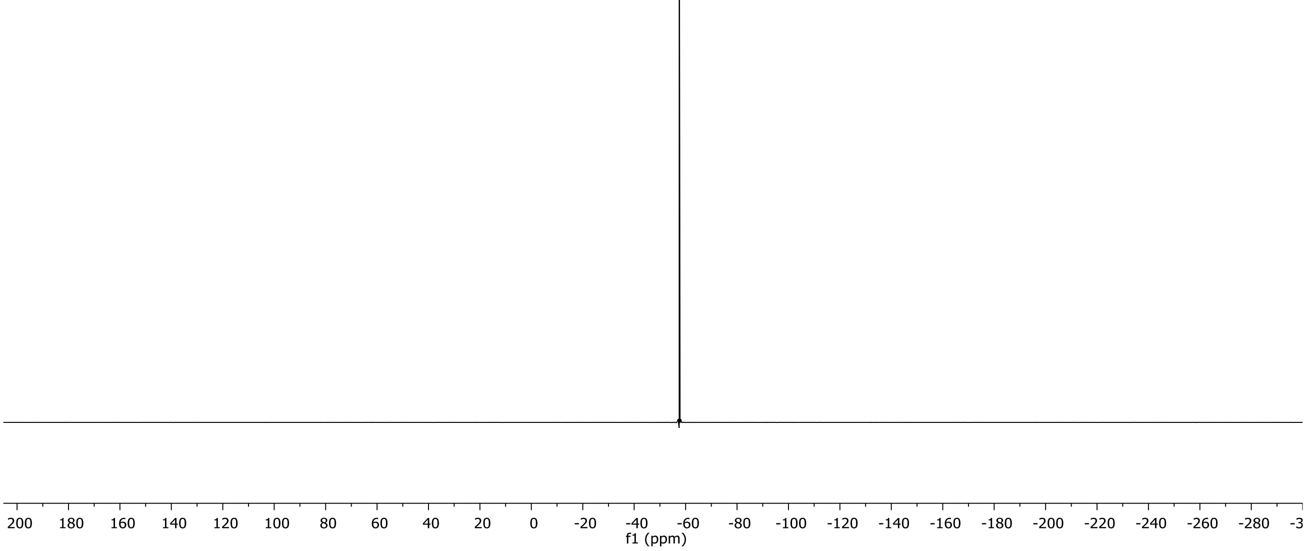

^{1}H Spectra

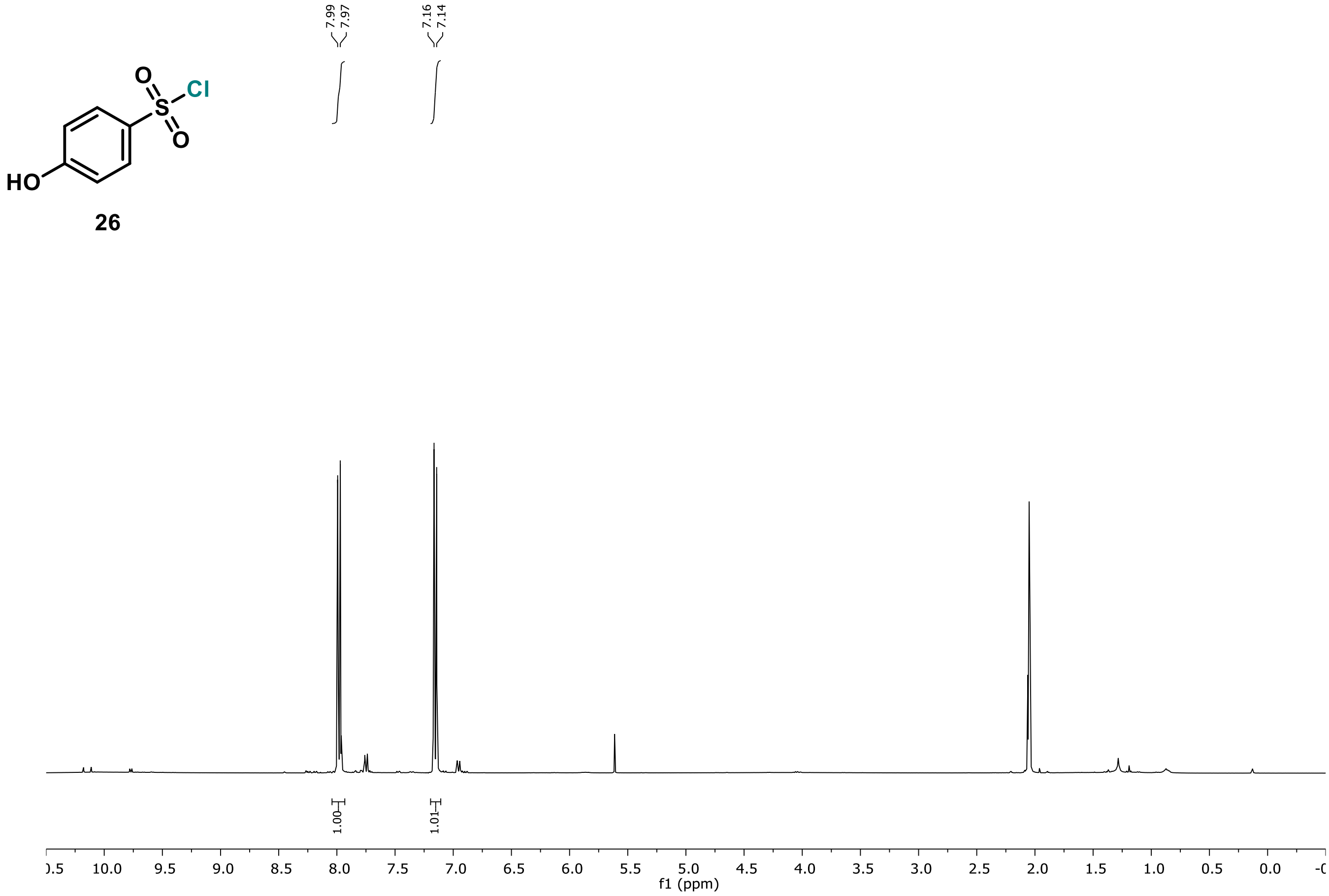

^{13}C Spectra

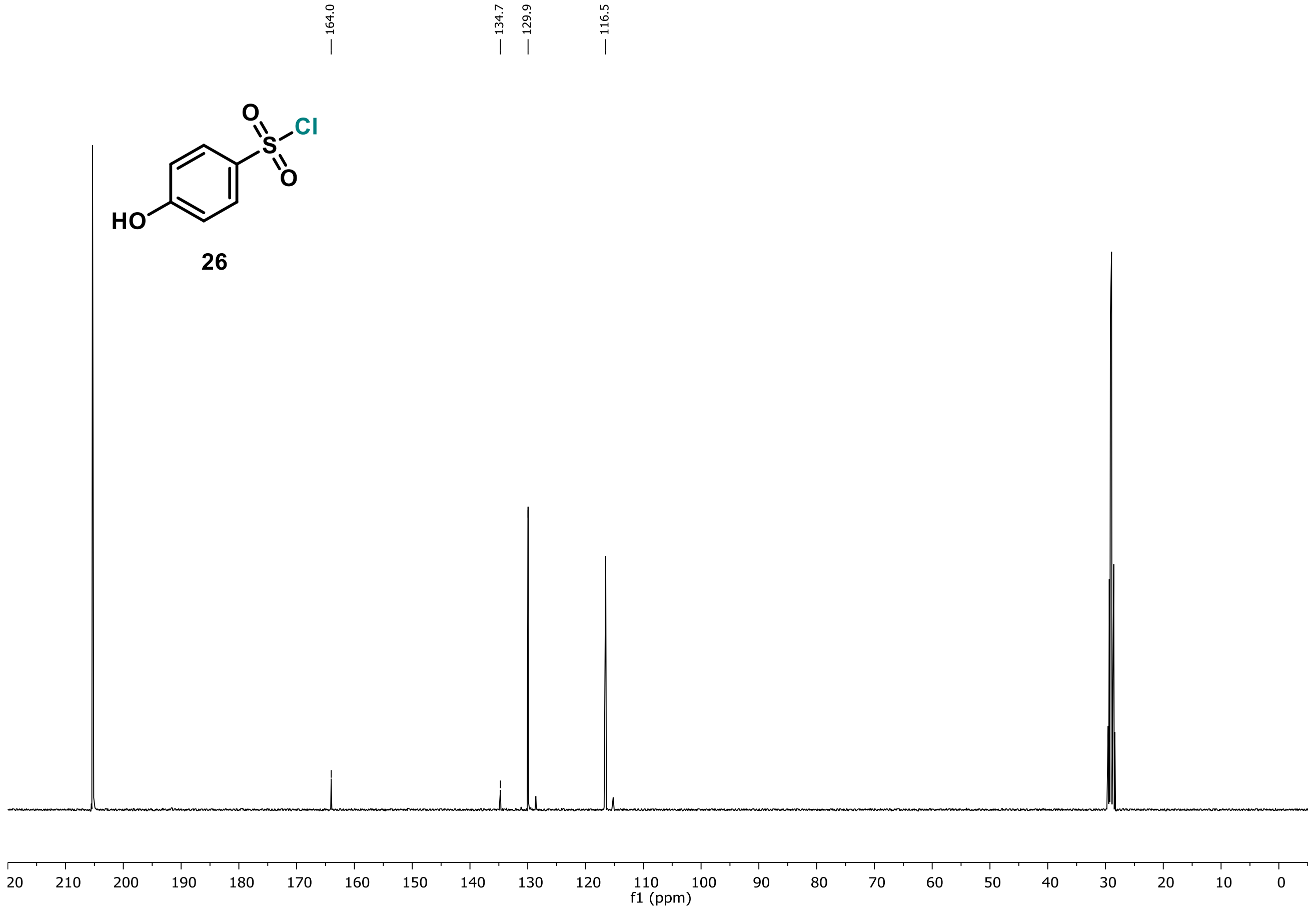

^{1}H Spectra

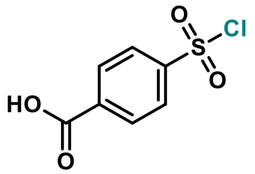

27

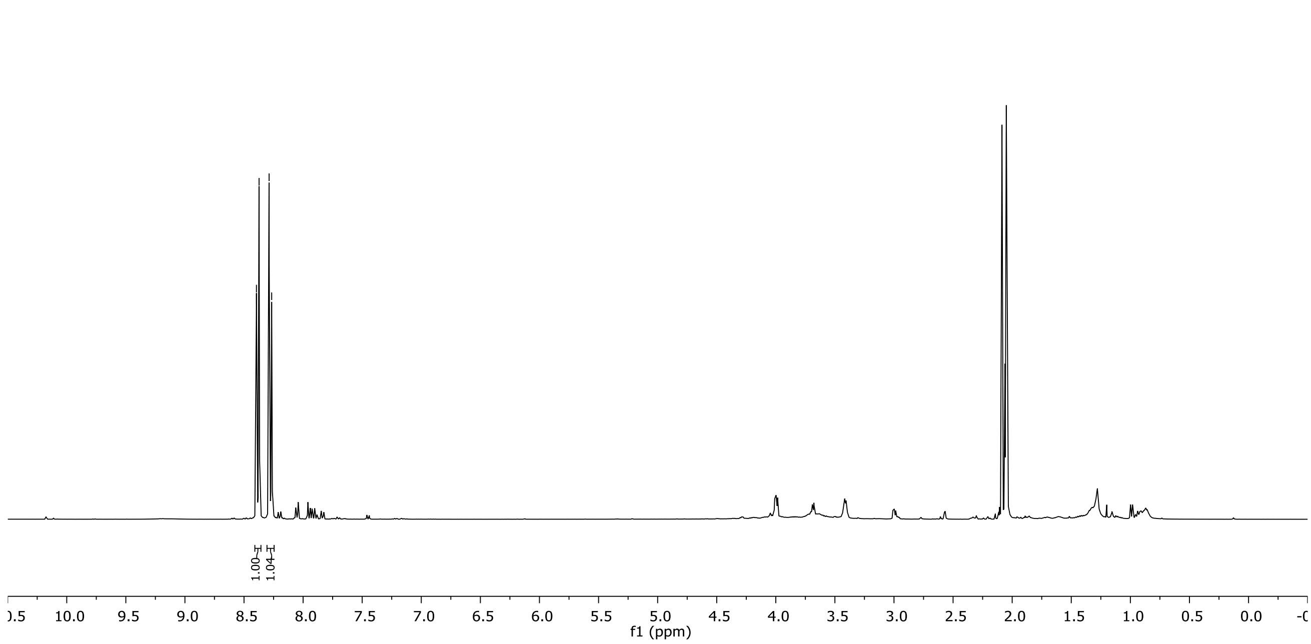

^{13}C Spectra

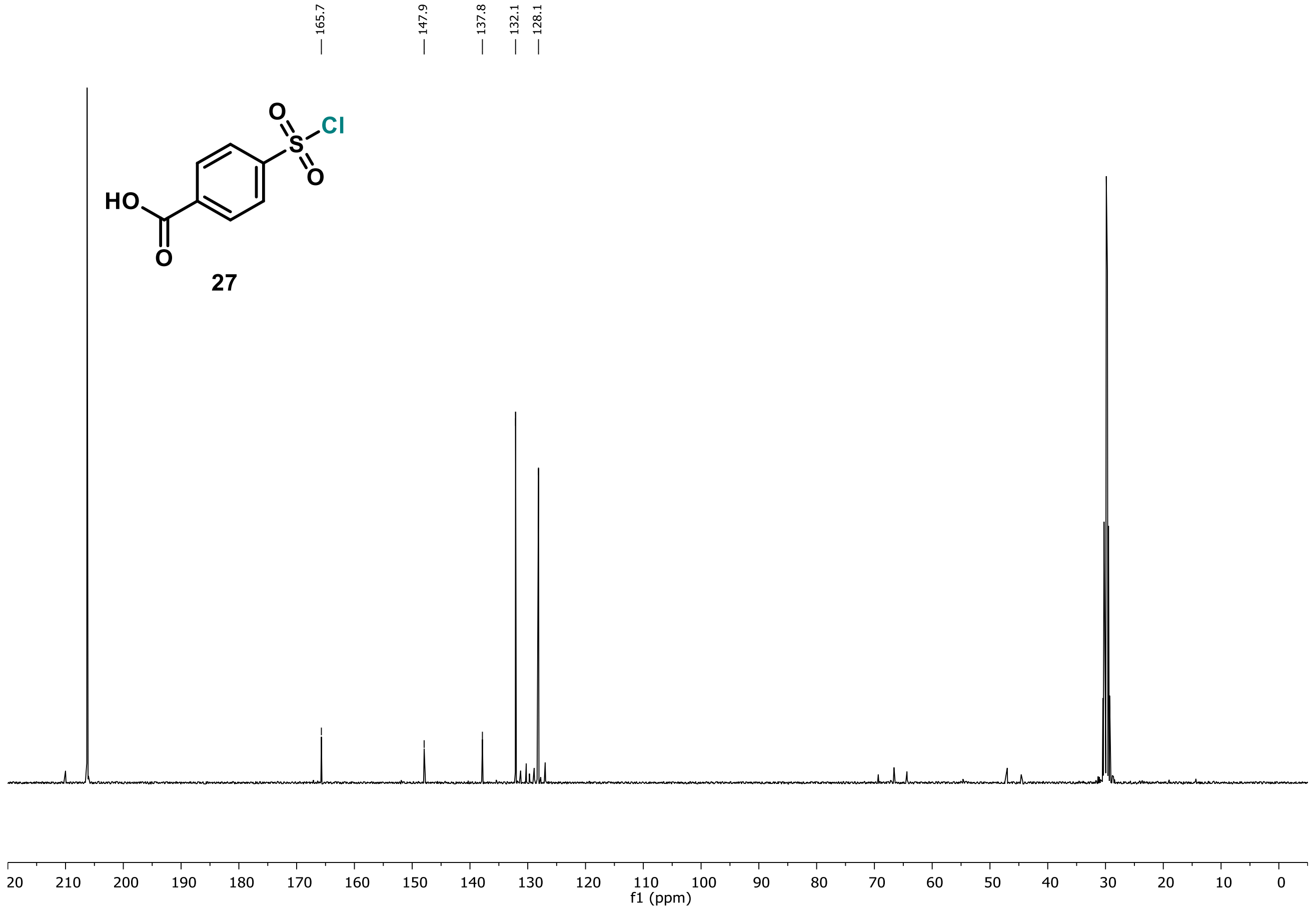

^{1}H Spectra

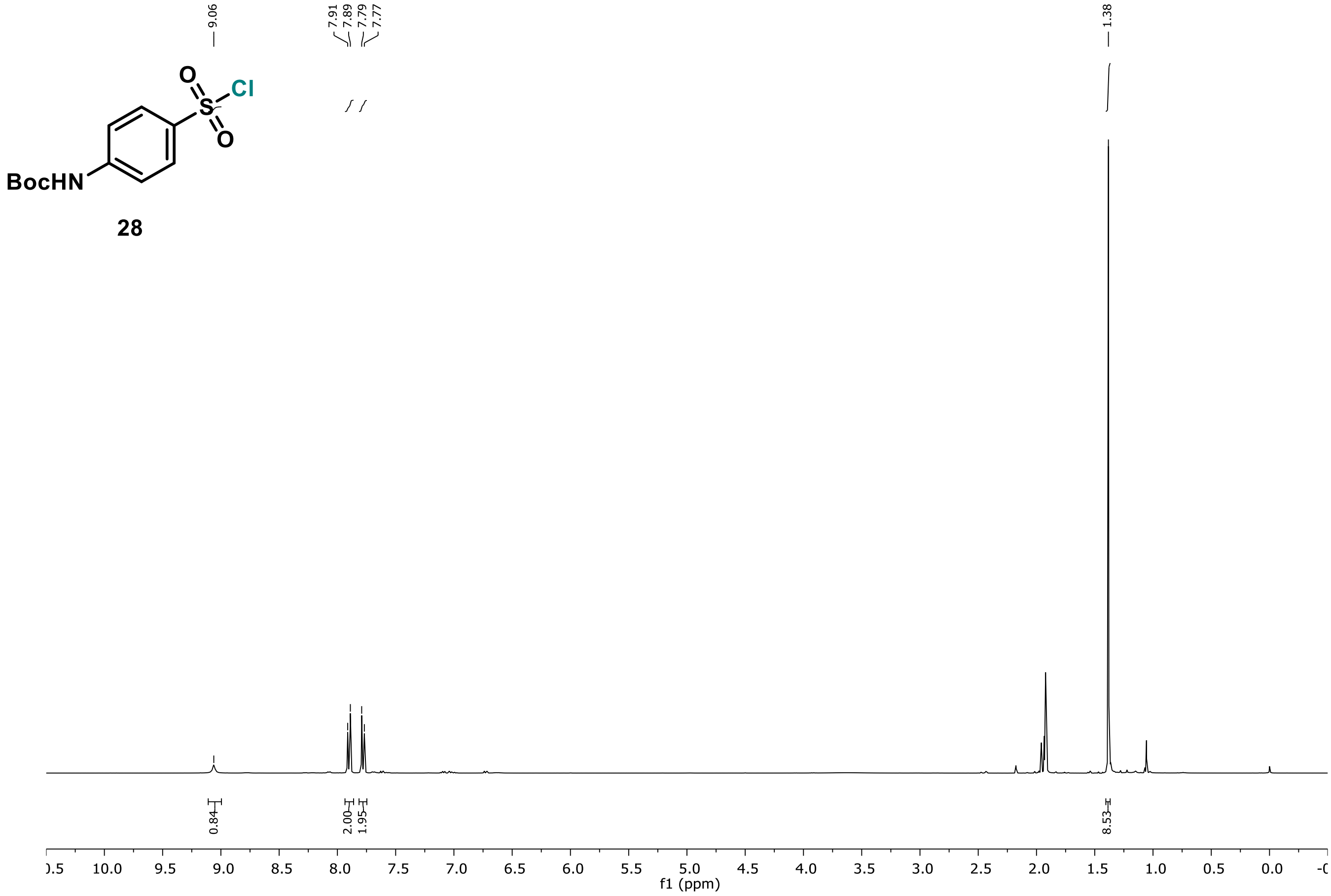

^{13}C Spectra

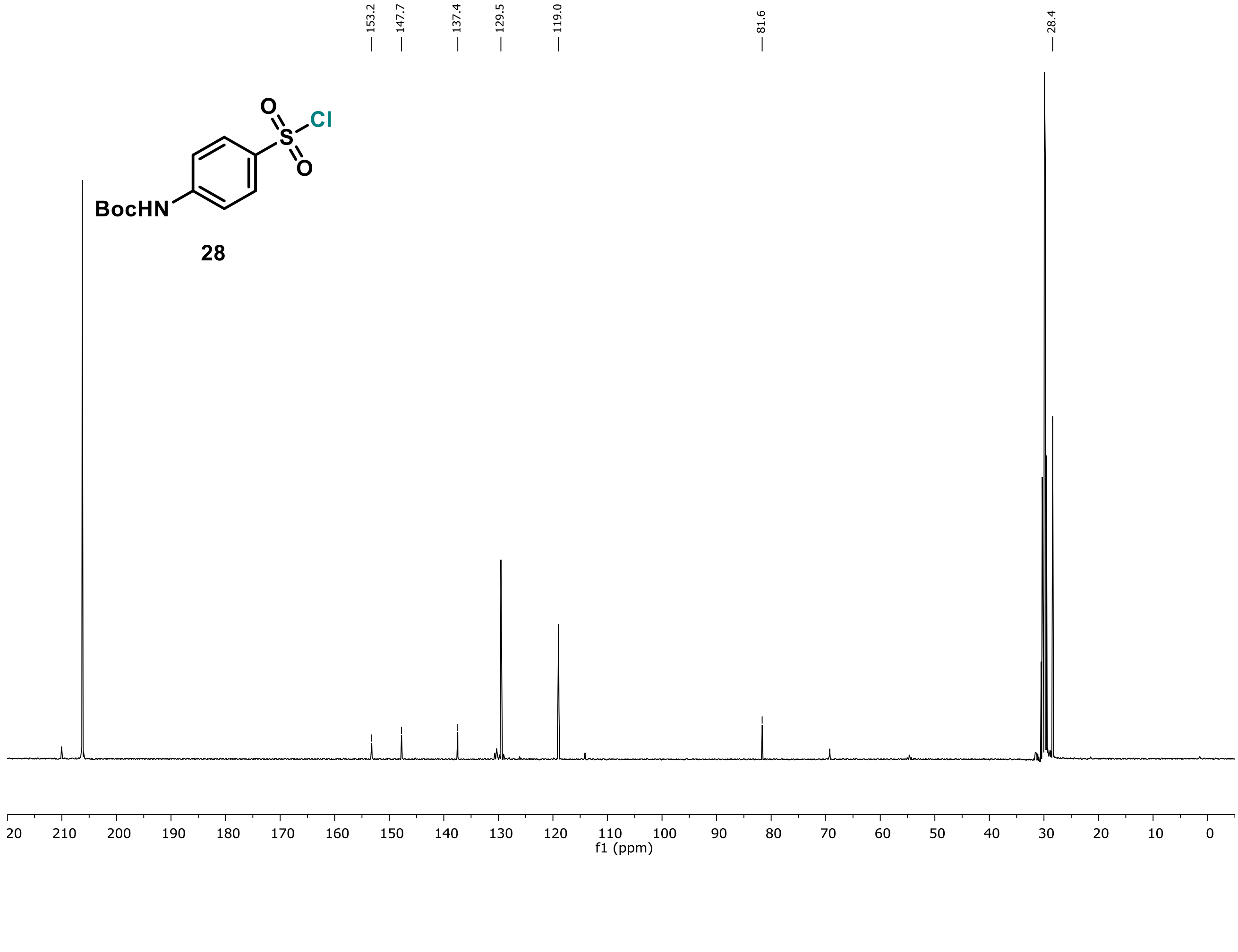

^{1}H Spectra

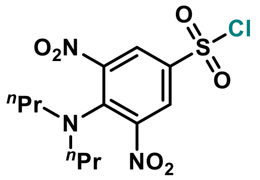

29

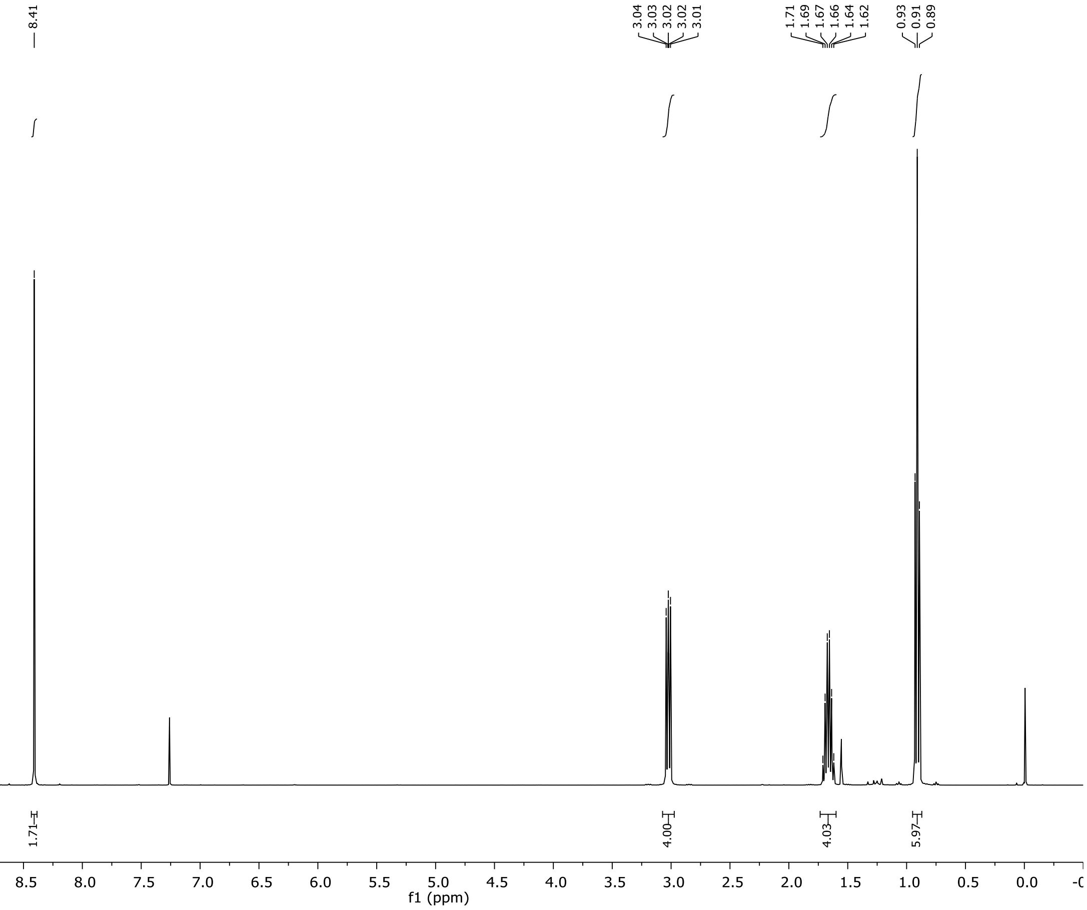

^{13}C Spectra

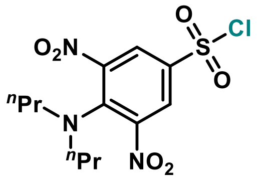

29

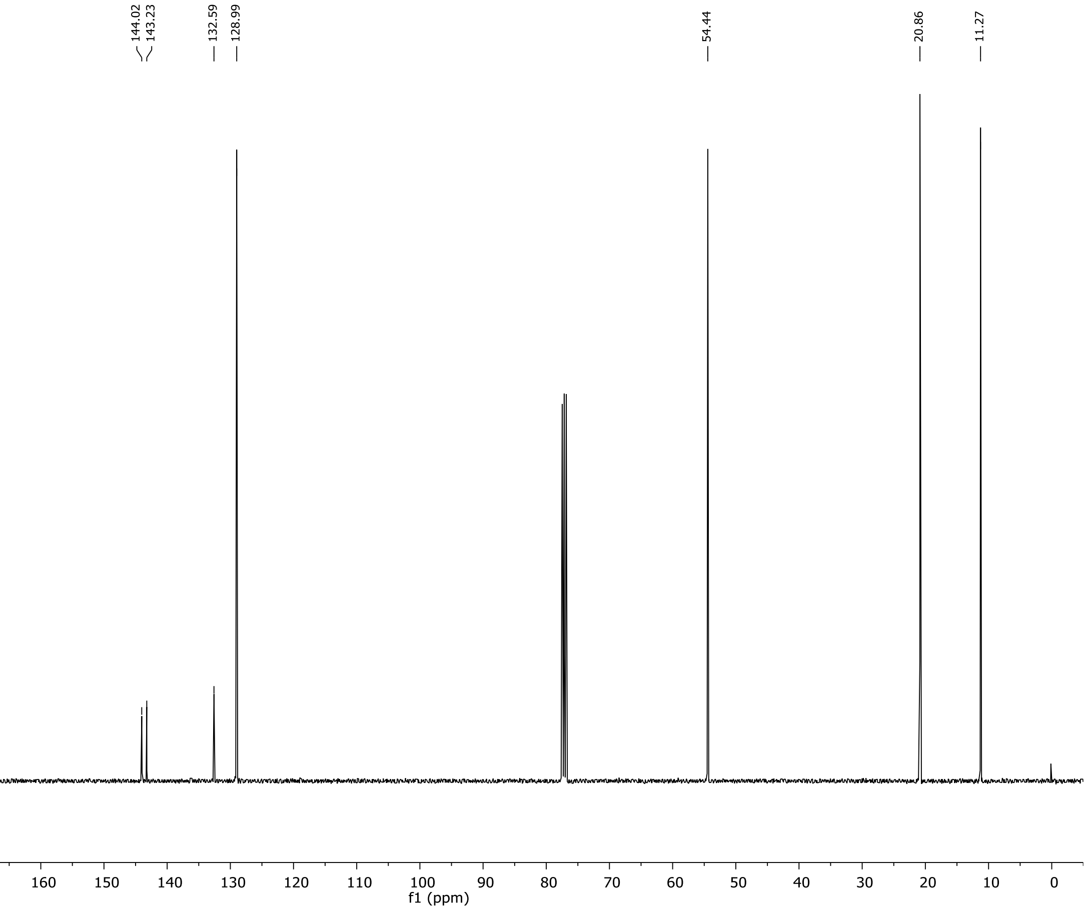

^{1}H Spectra

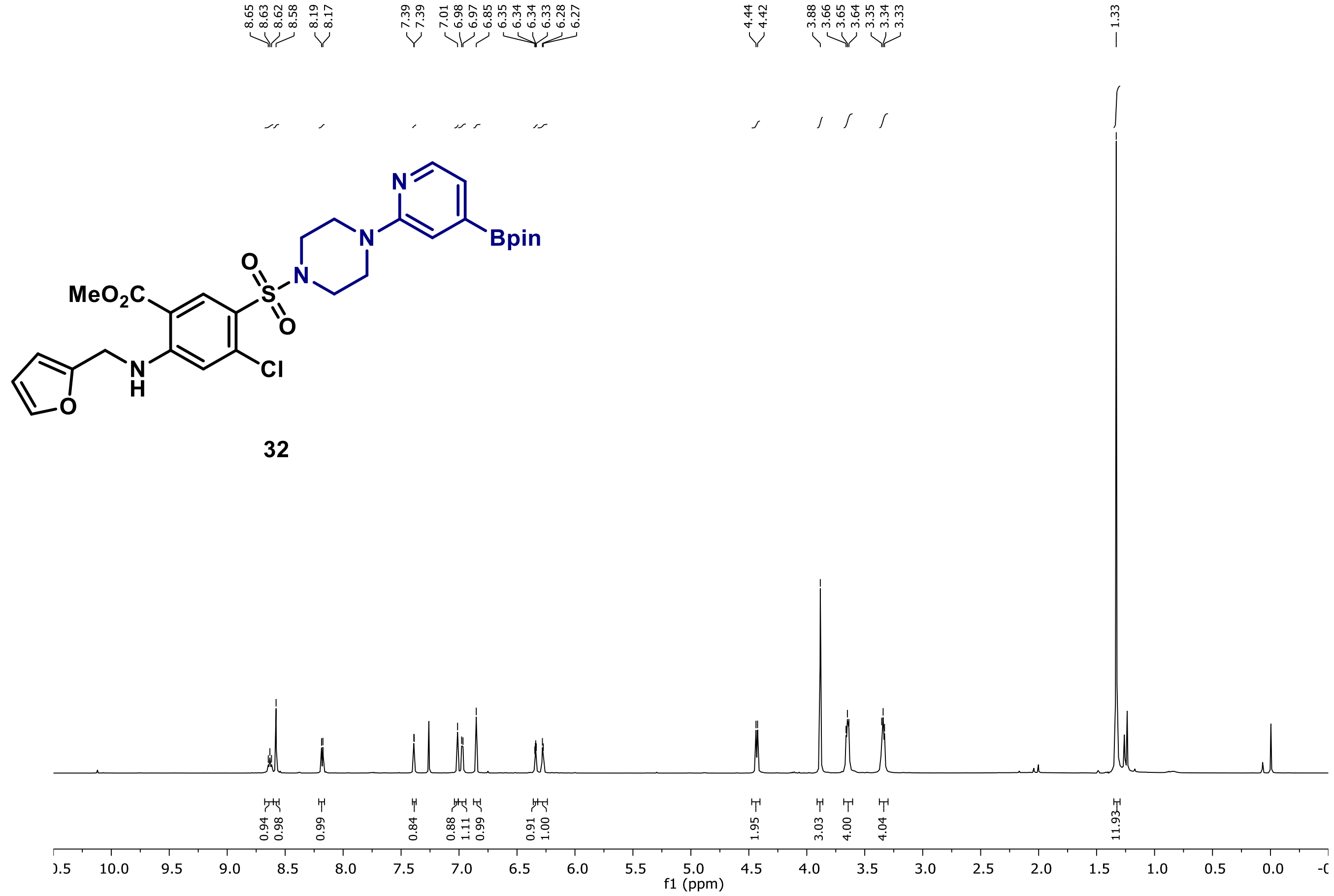

^{13}C Spectra

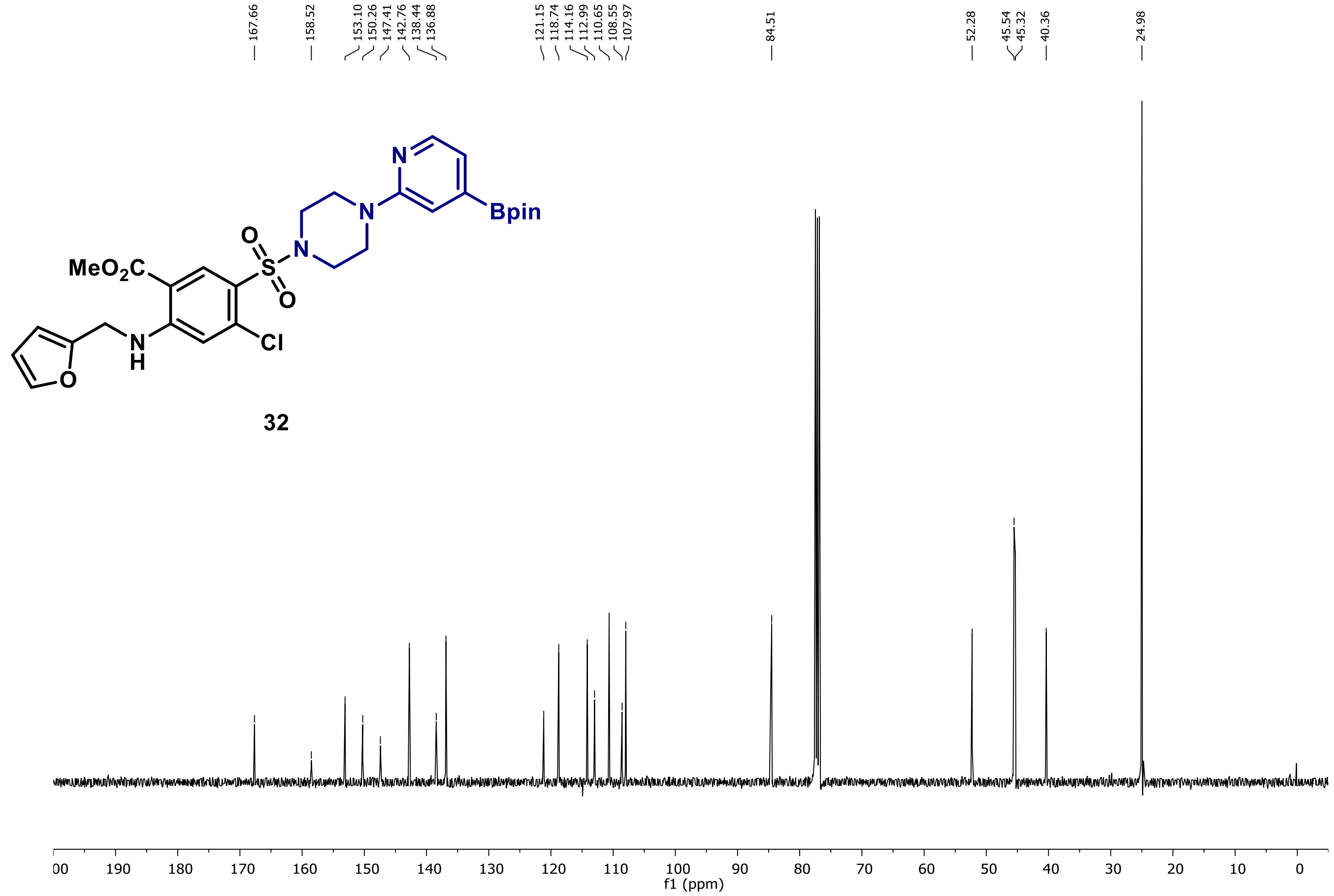

[1]H Spectra

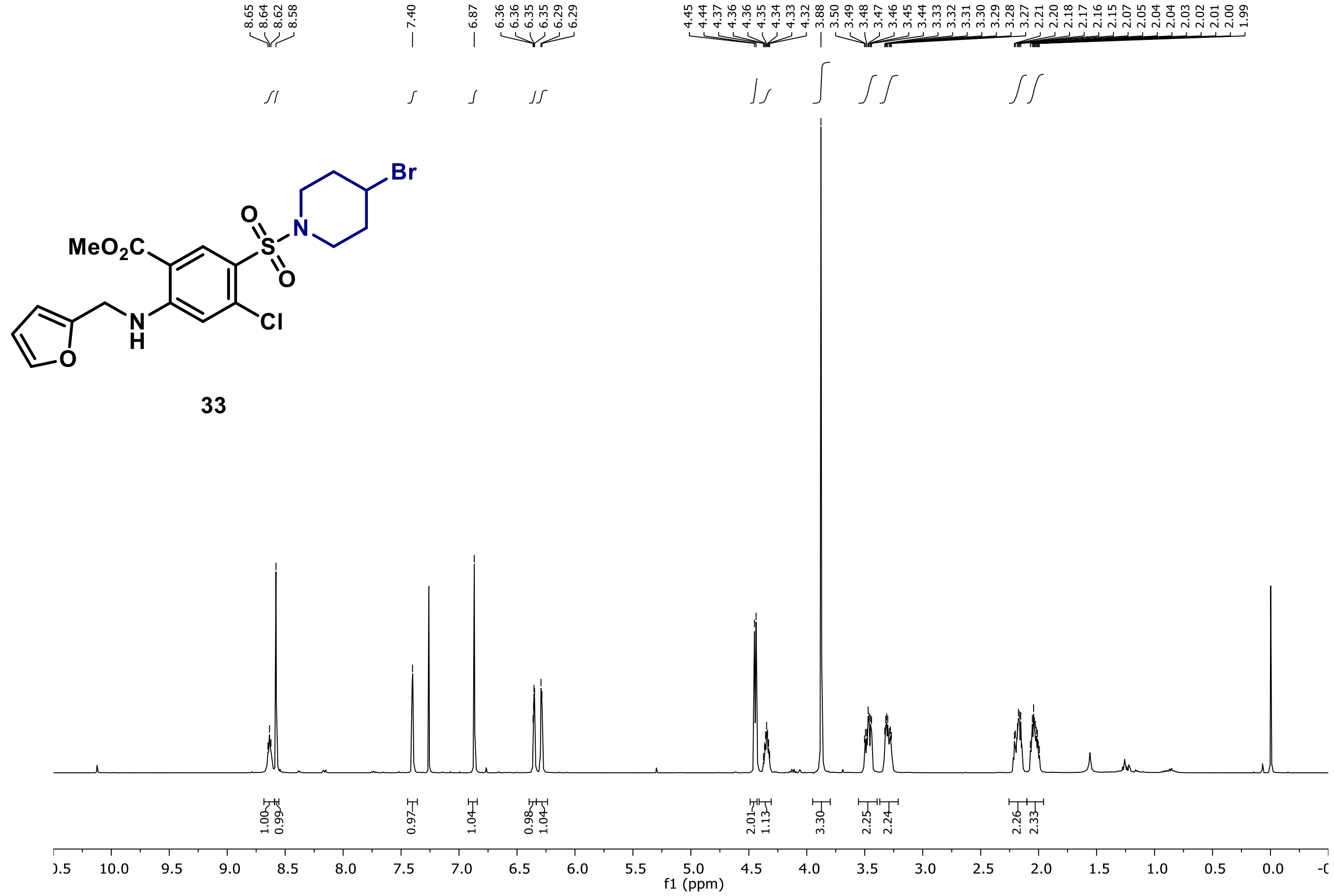

[13]C Spectra

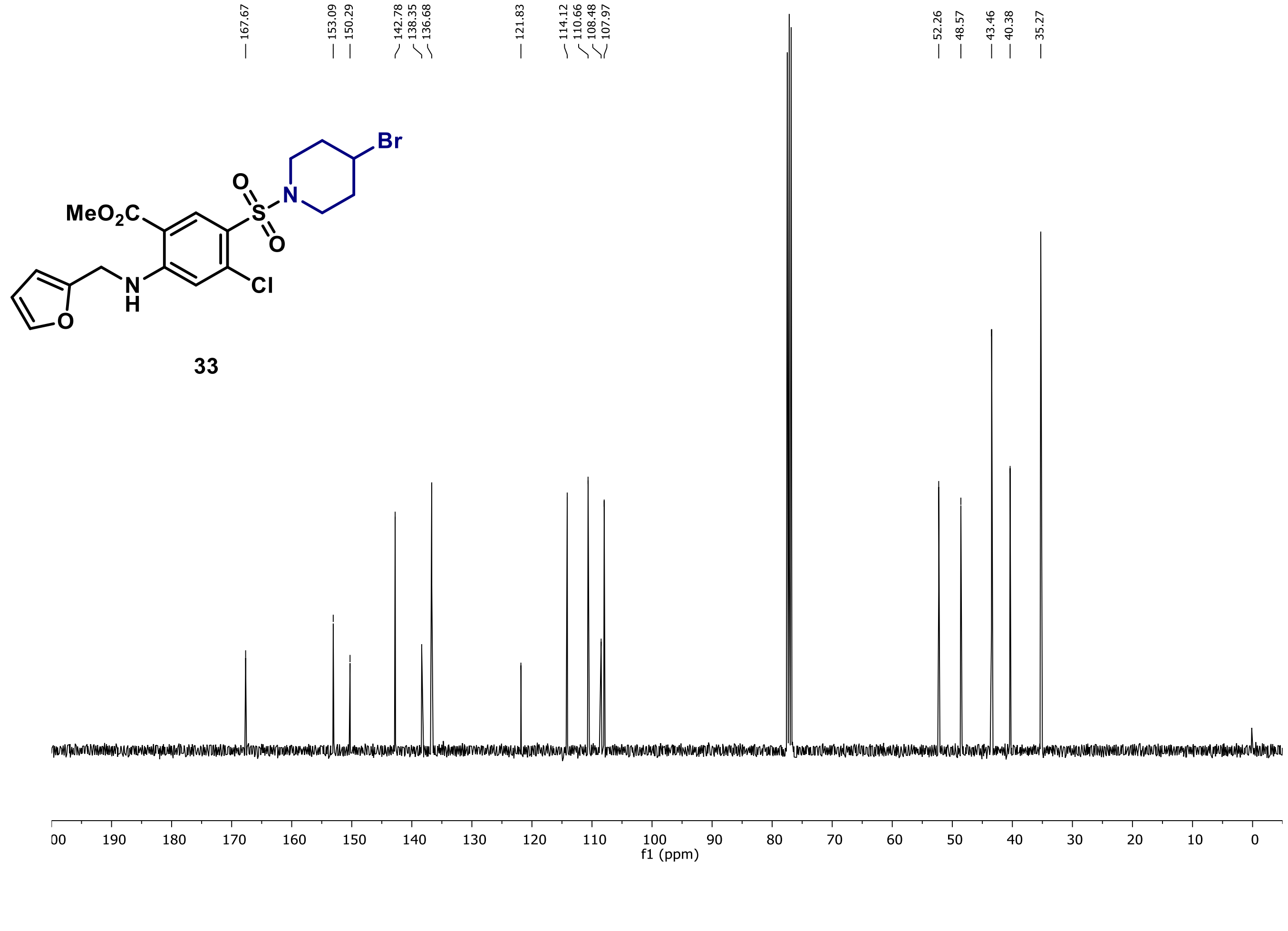

^{1}H Spectra

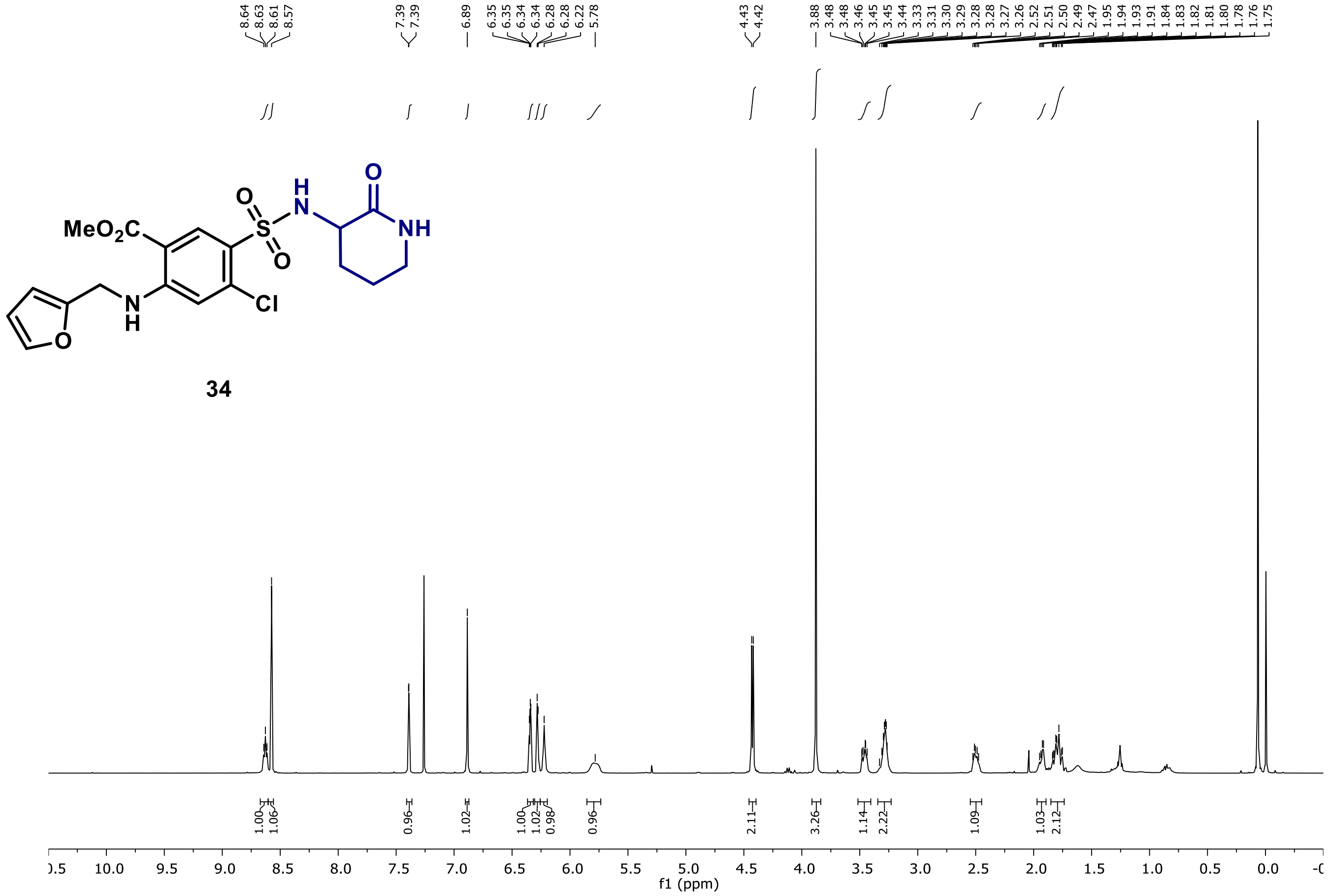

^{13}C Spectra

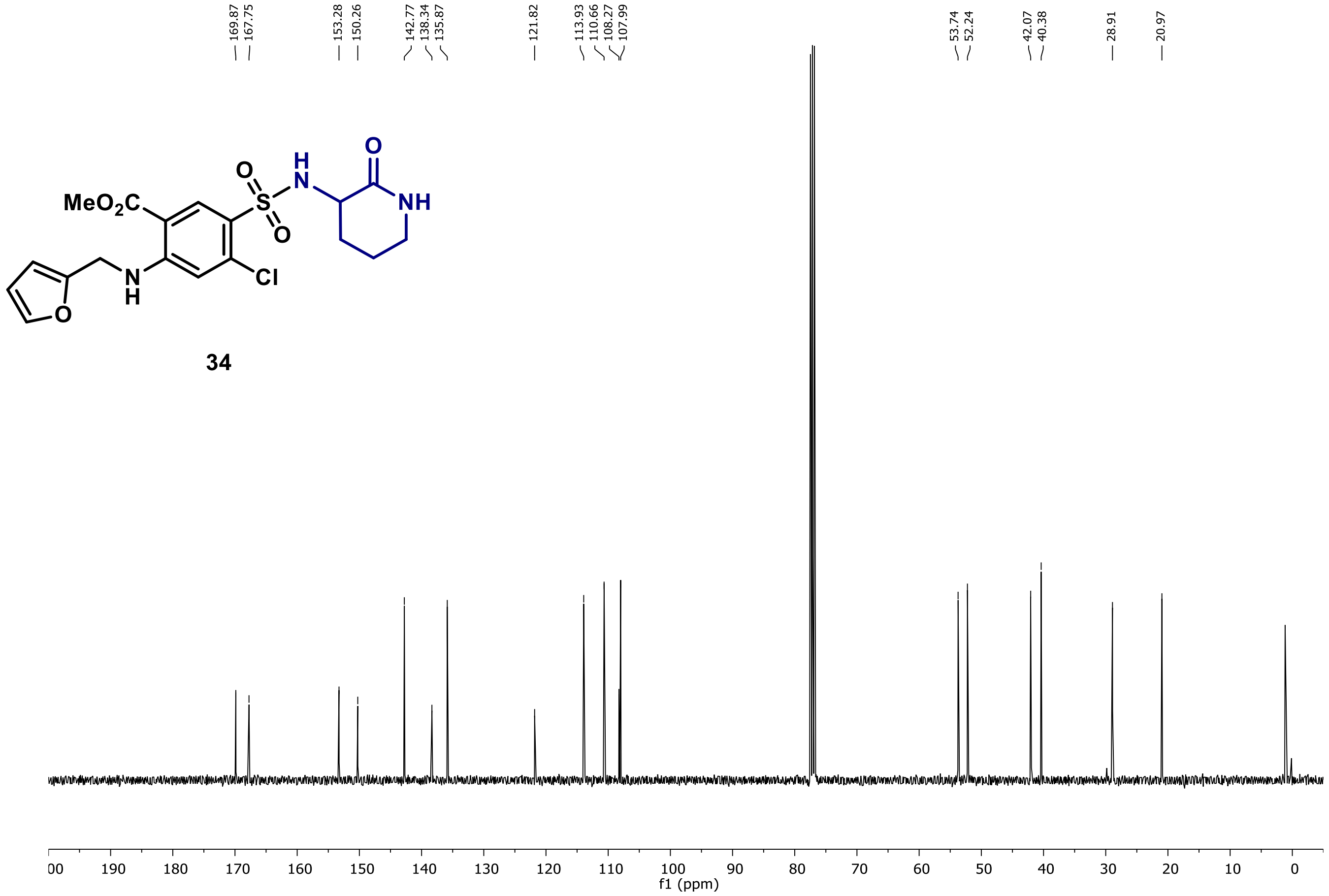

[1]H Spectra

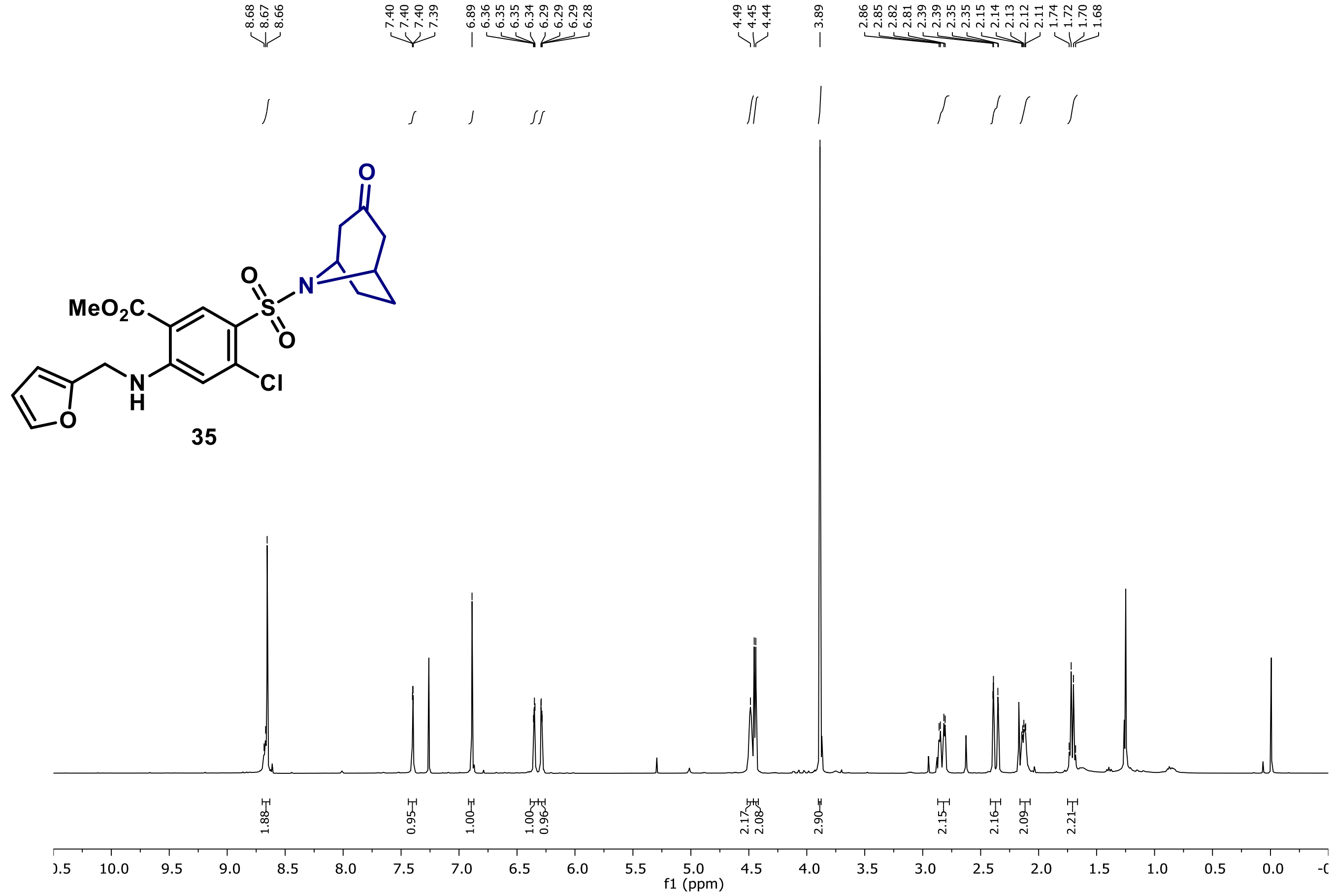

[13]C Spectra

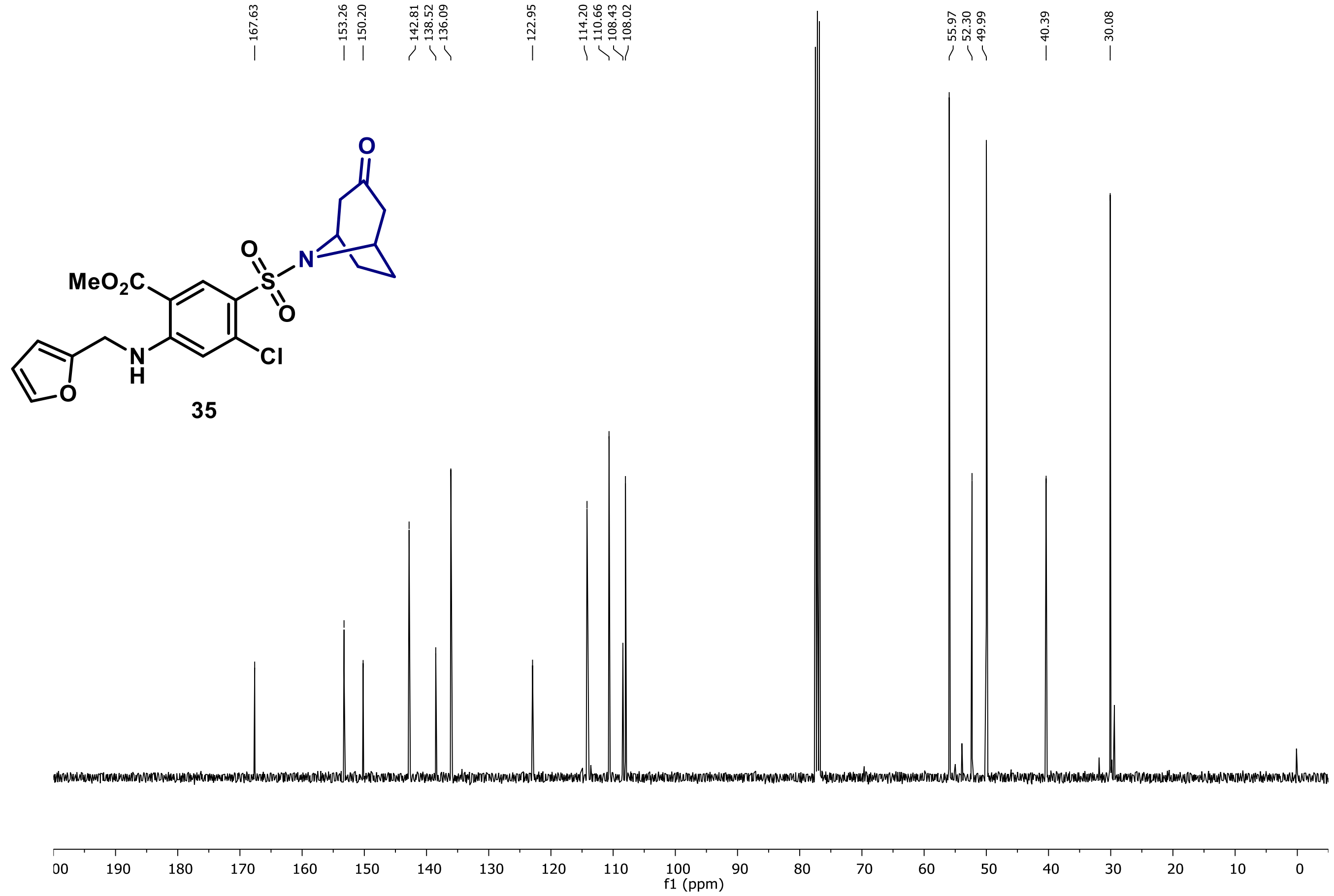

^{1}H Spectra

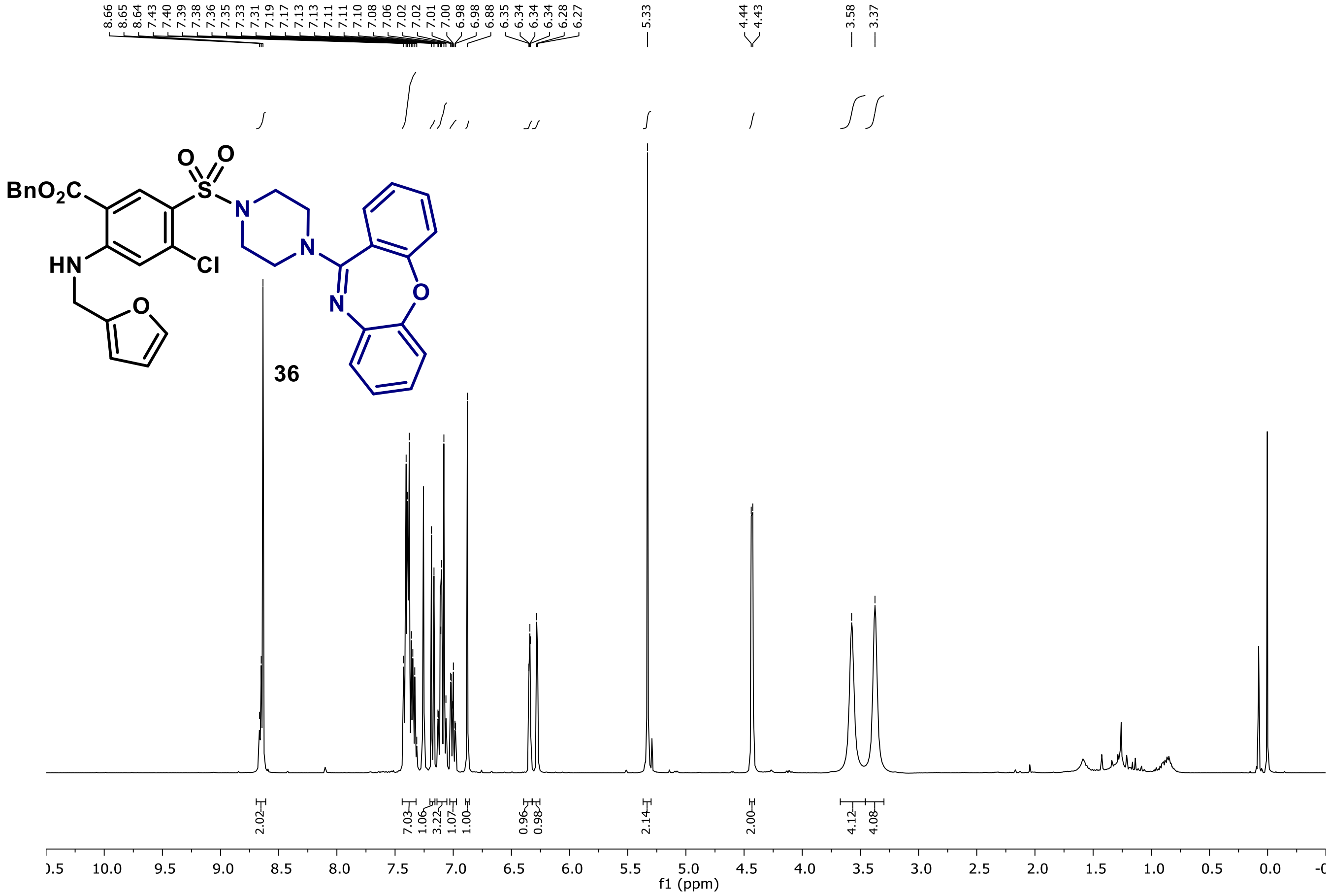

^{13}C Spectra

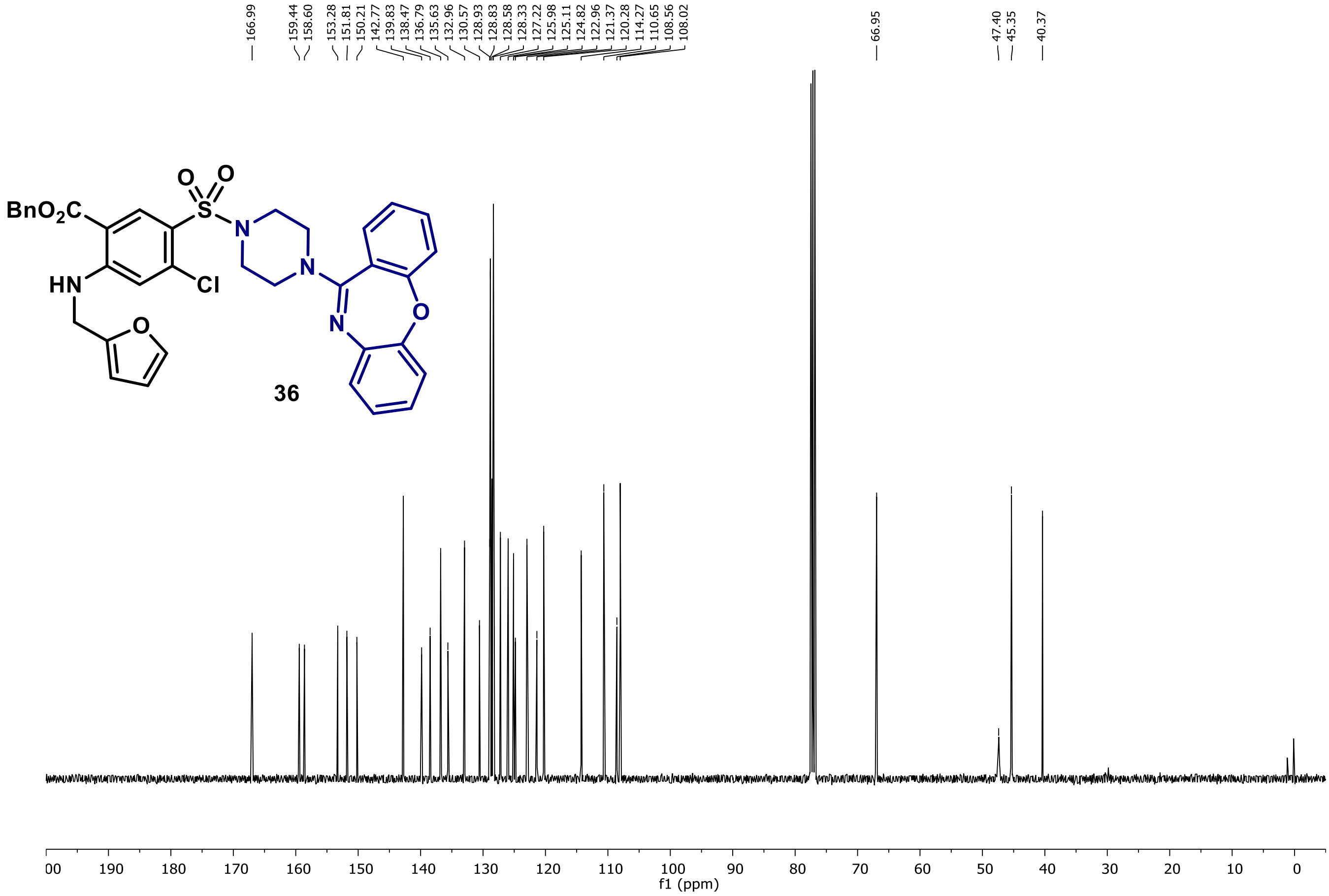

[1]H Spectra

37

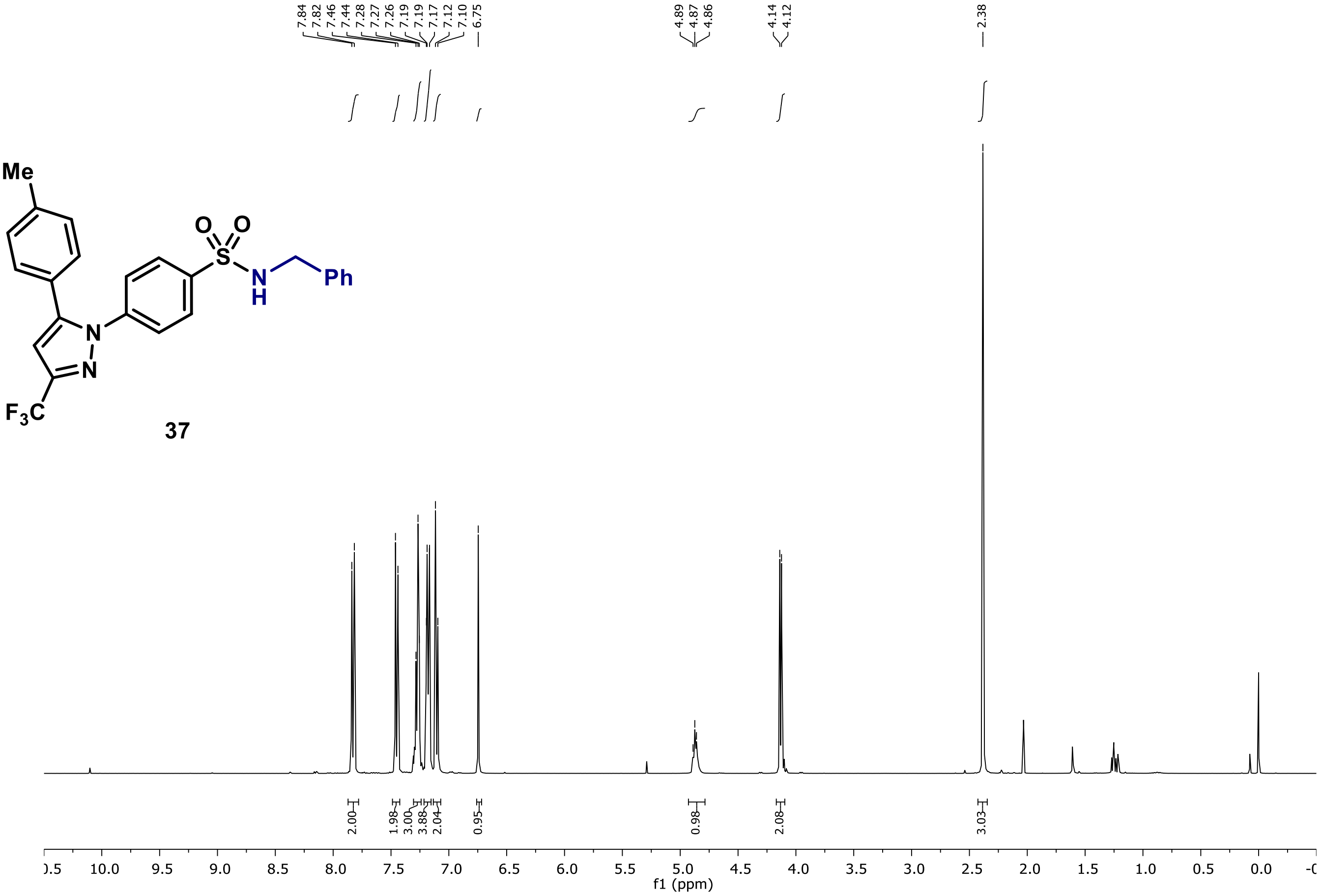

[13]C Spectra

37

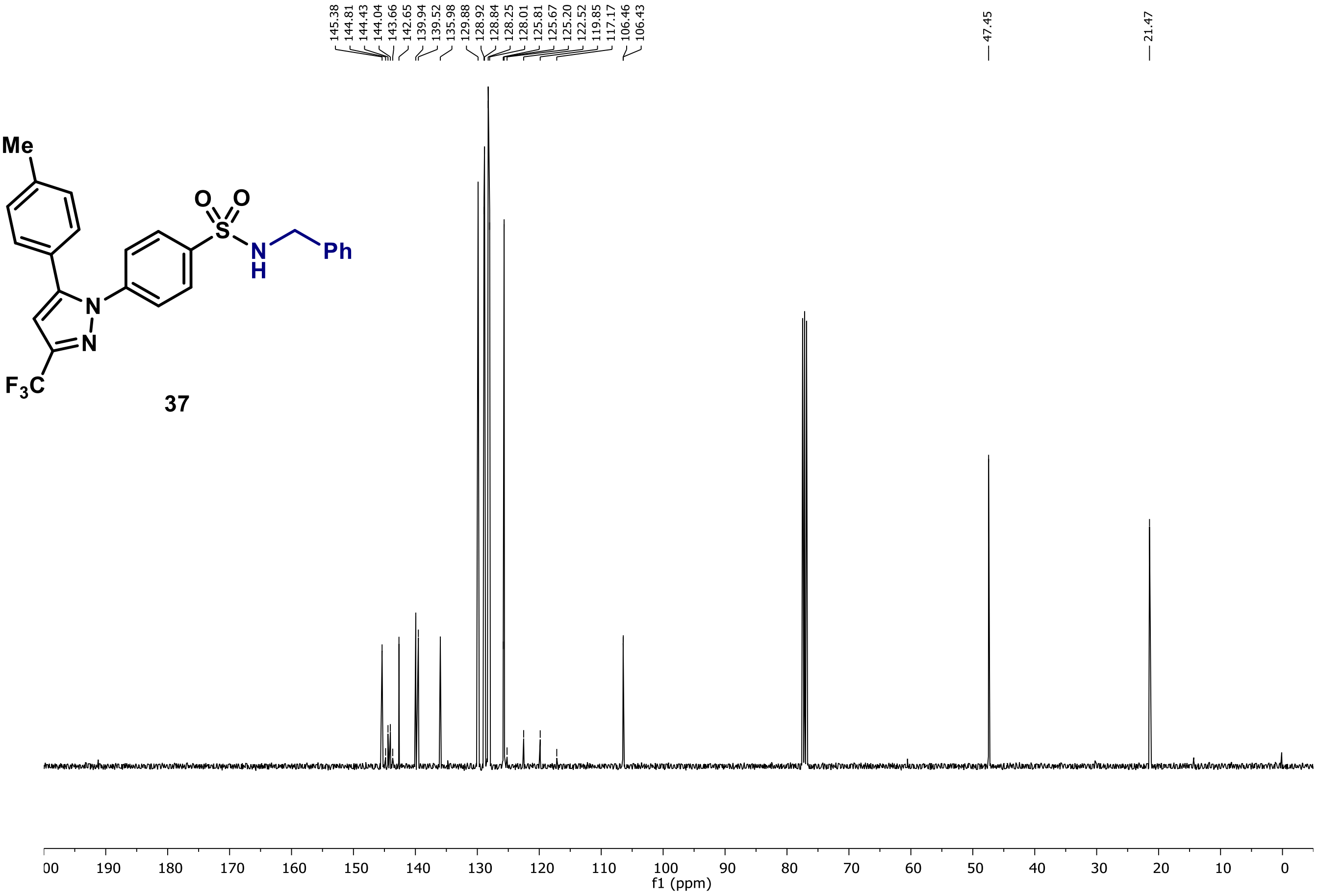

19F Spectra

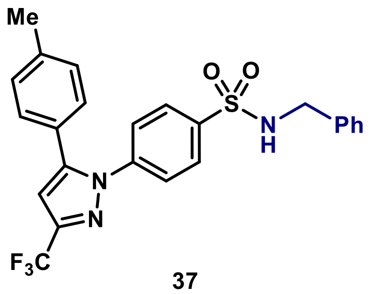

37

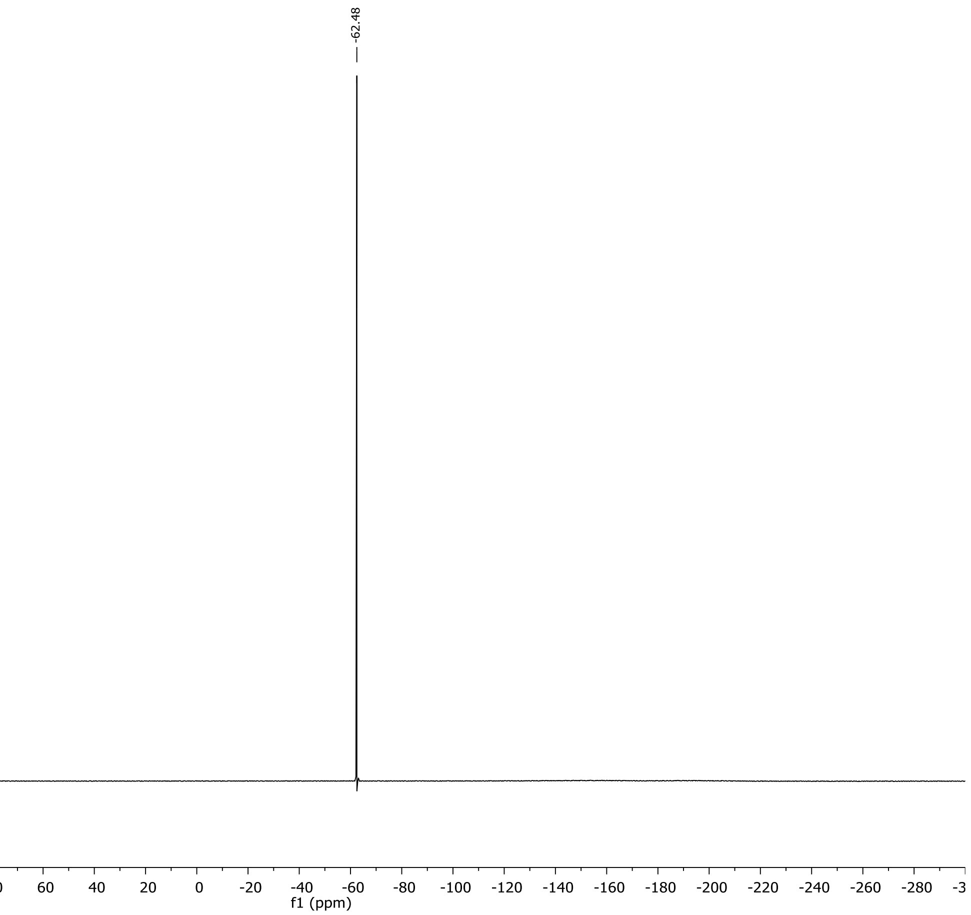

[1]H Spectra

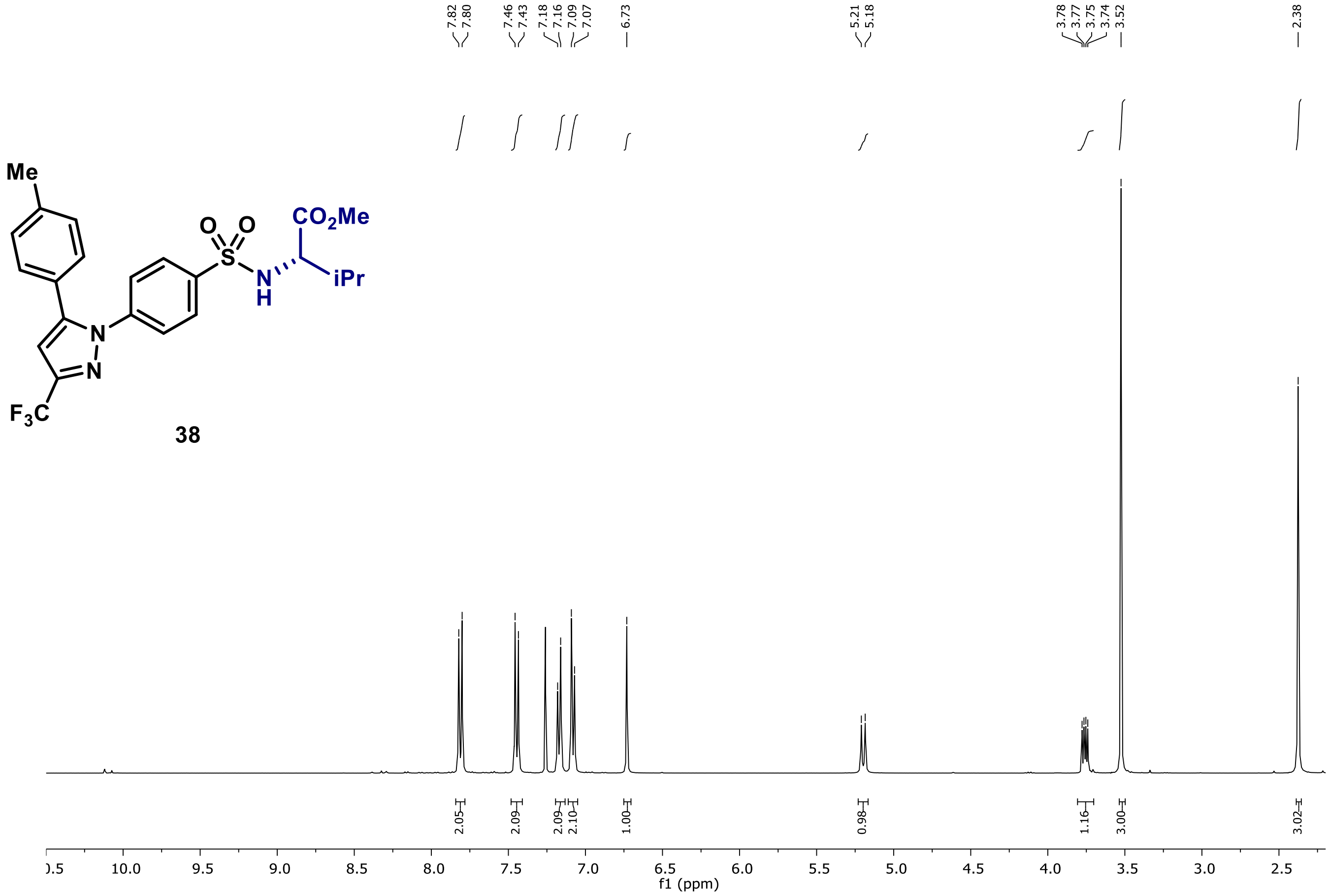

38

[13]C Spectra

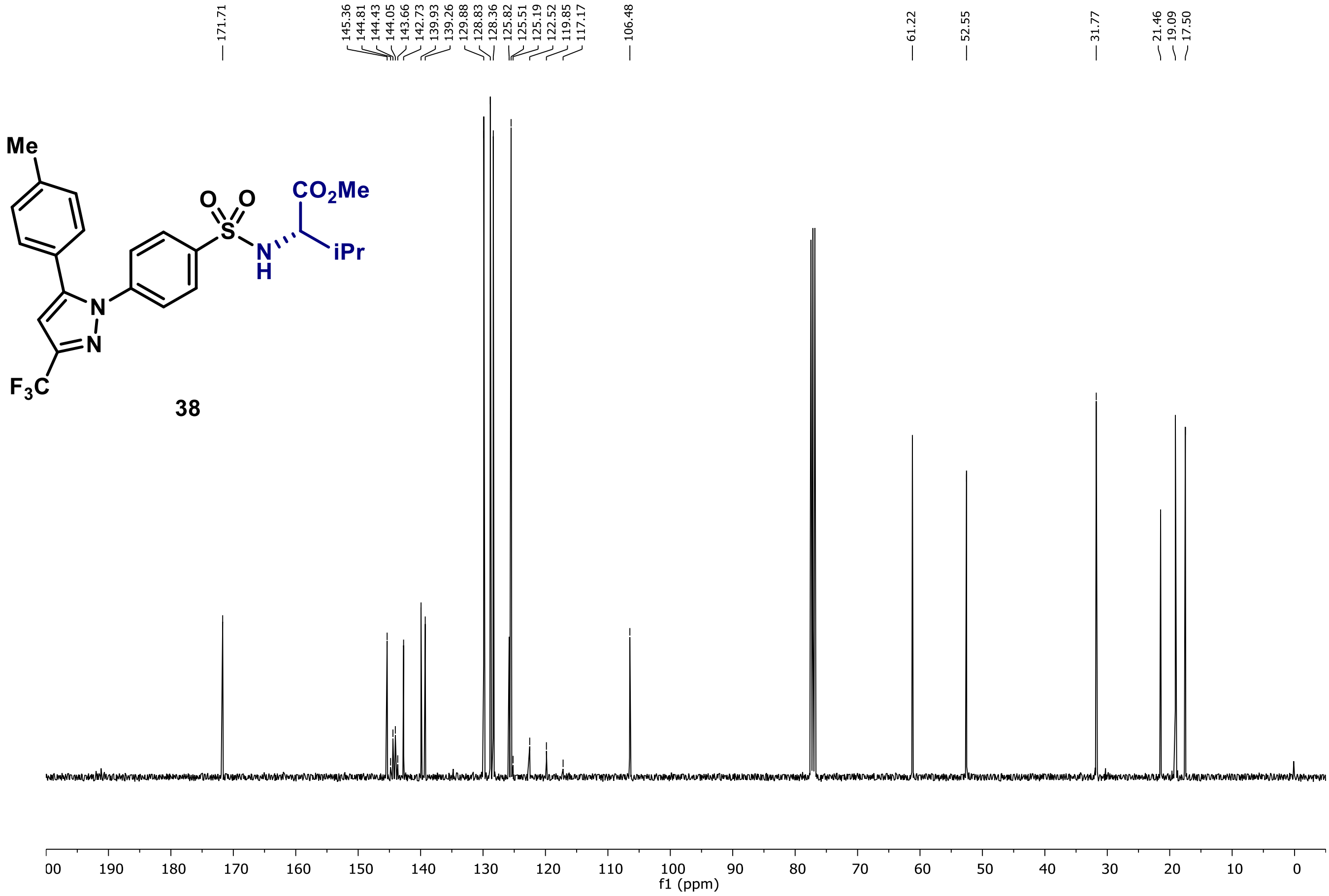

38

^{19}F Spectra

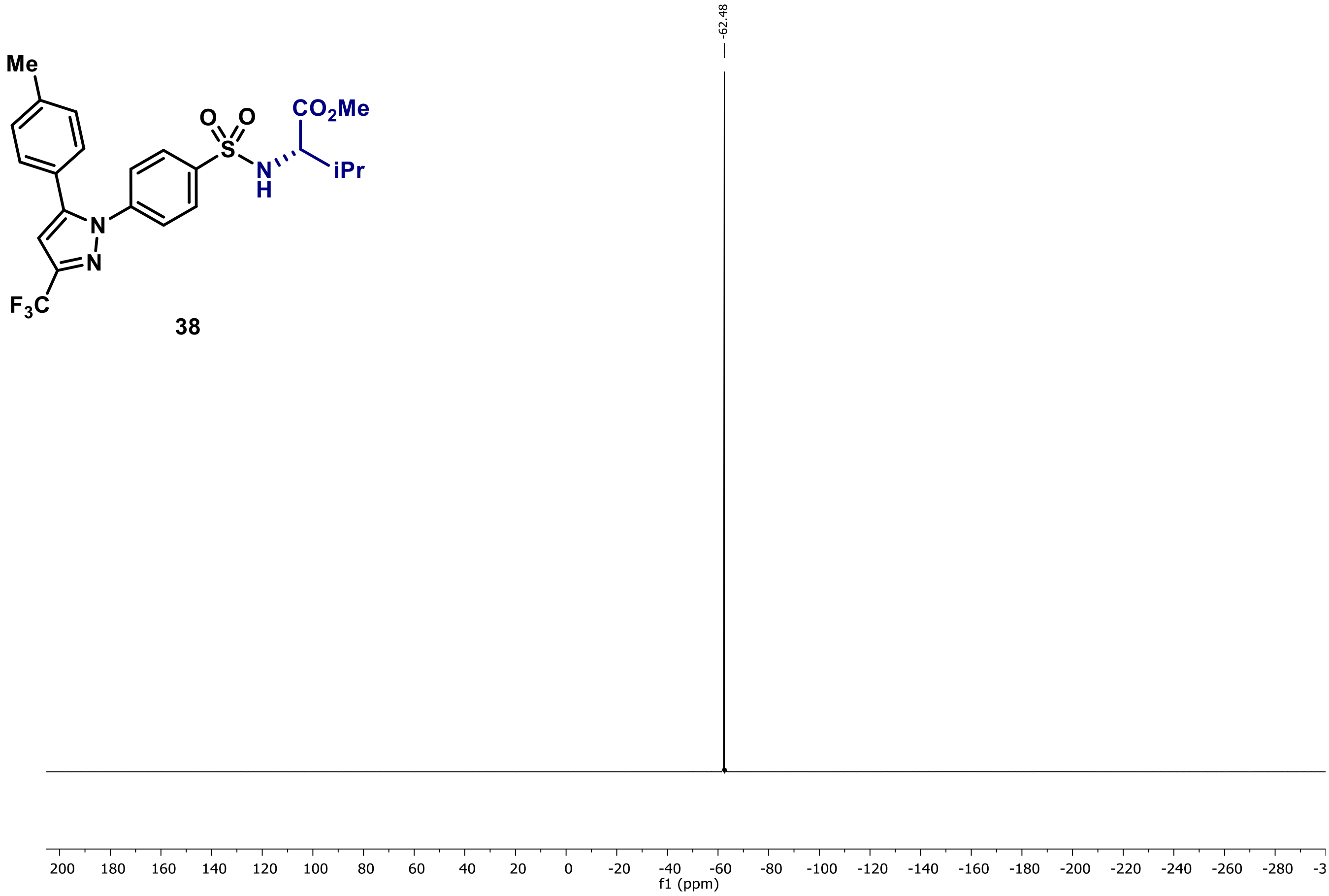
Me
O
O
S
N
H
CO2Me
iPr
N
N
F3C
38
-62.48
200
180
160
140
120
100
80
60
40
20
0
-20
-40
-60
-80
-100
-120
-140
-160
-180
-200
-220
-240
-260
-280
-3
f1 (ppm)

[1]H Spectra

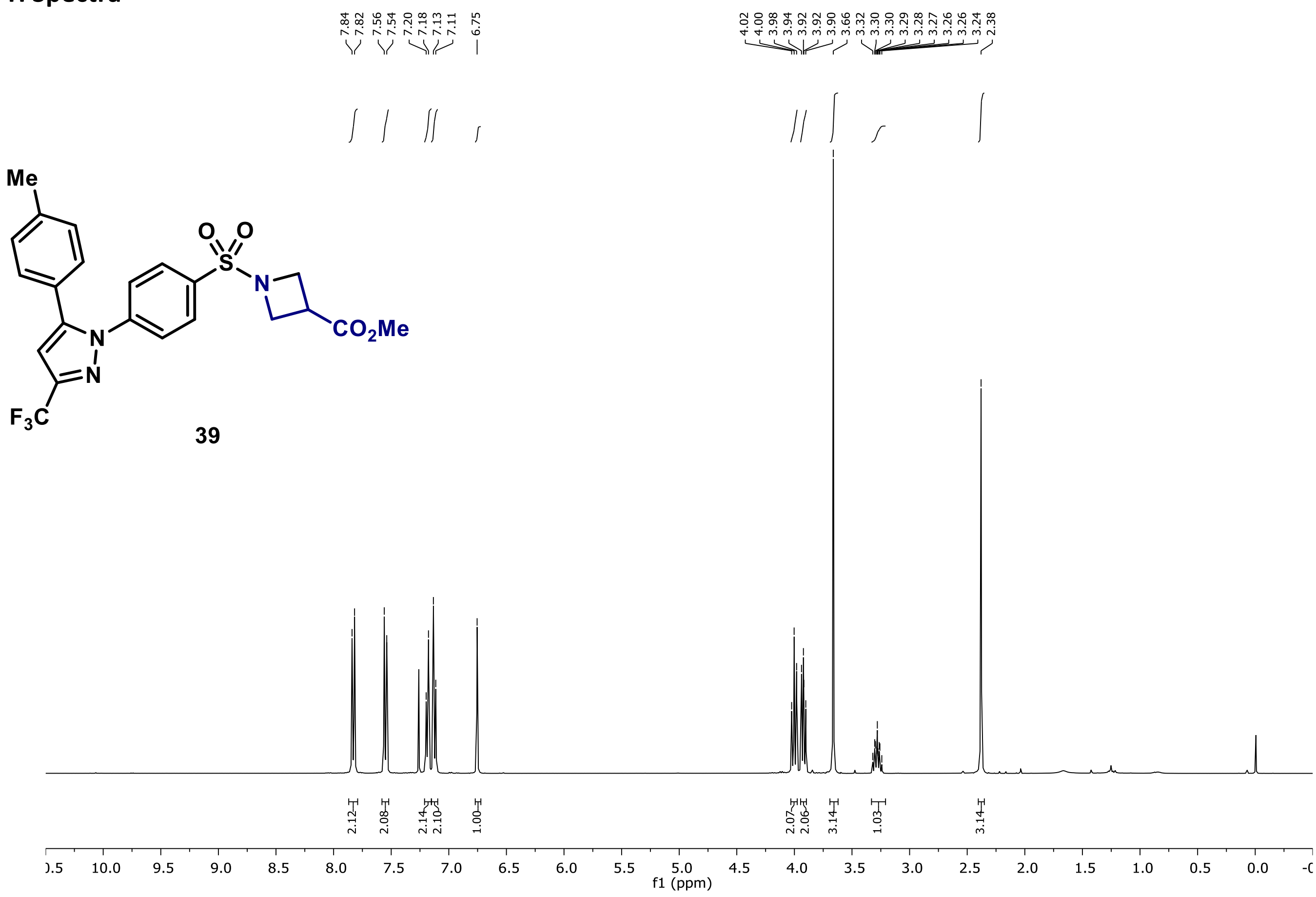
39

[13]C Spectra

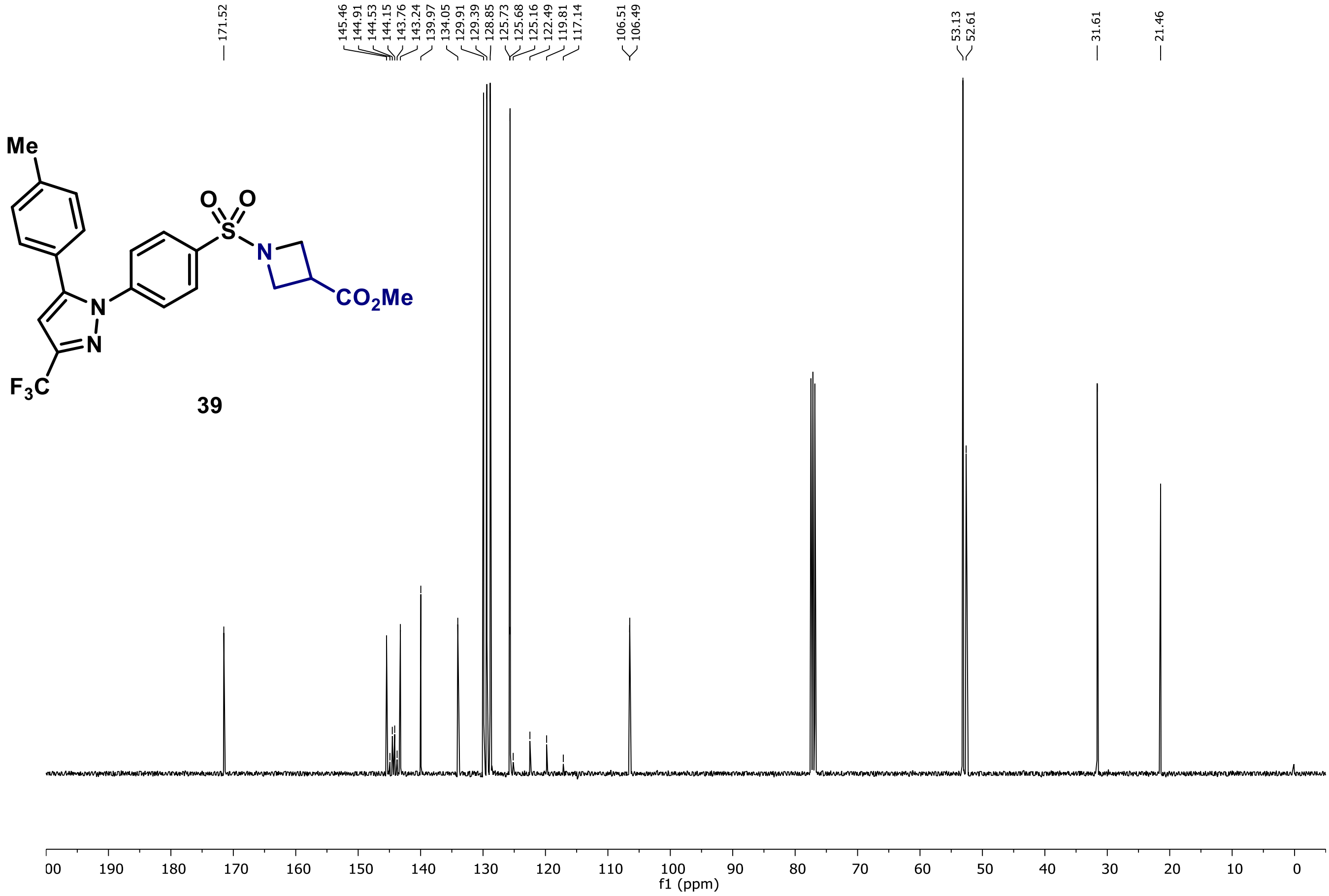
39

^{19}F Spectra

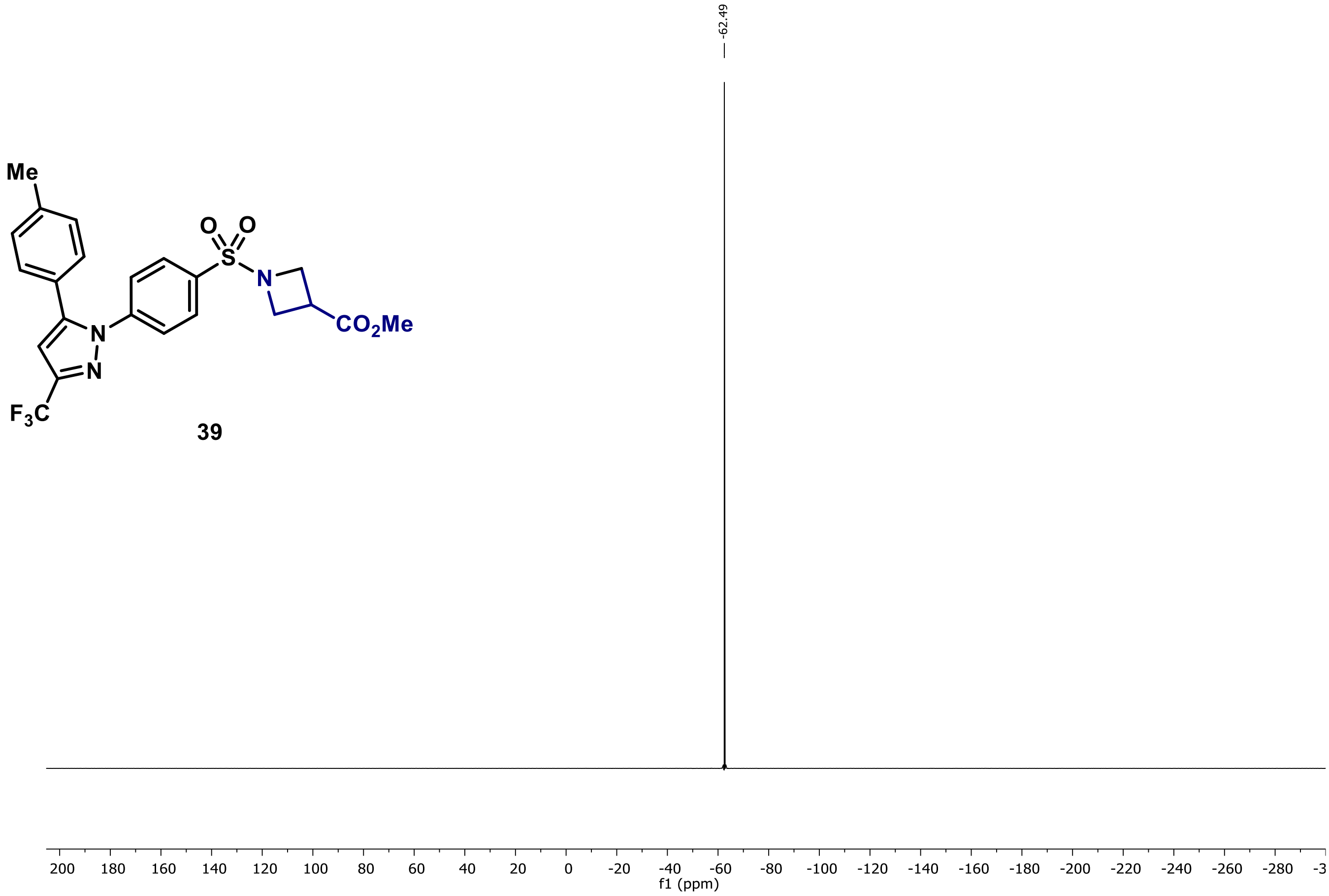

Me
O
O
S
N
CO2Me
N
N
F3C
39
-62.49
200 180 160 140 120 100 80 60 40 20 0 -20 -40 -60 -80 -100 -120 -140 -160 -180 -200 -220 -240 -260 -280 -3
f1 (ppm)

[1]H Spectra

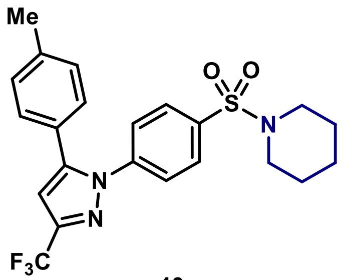

40

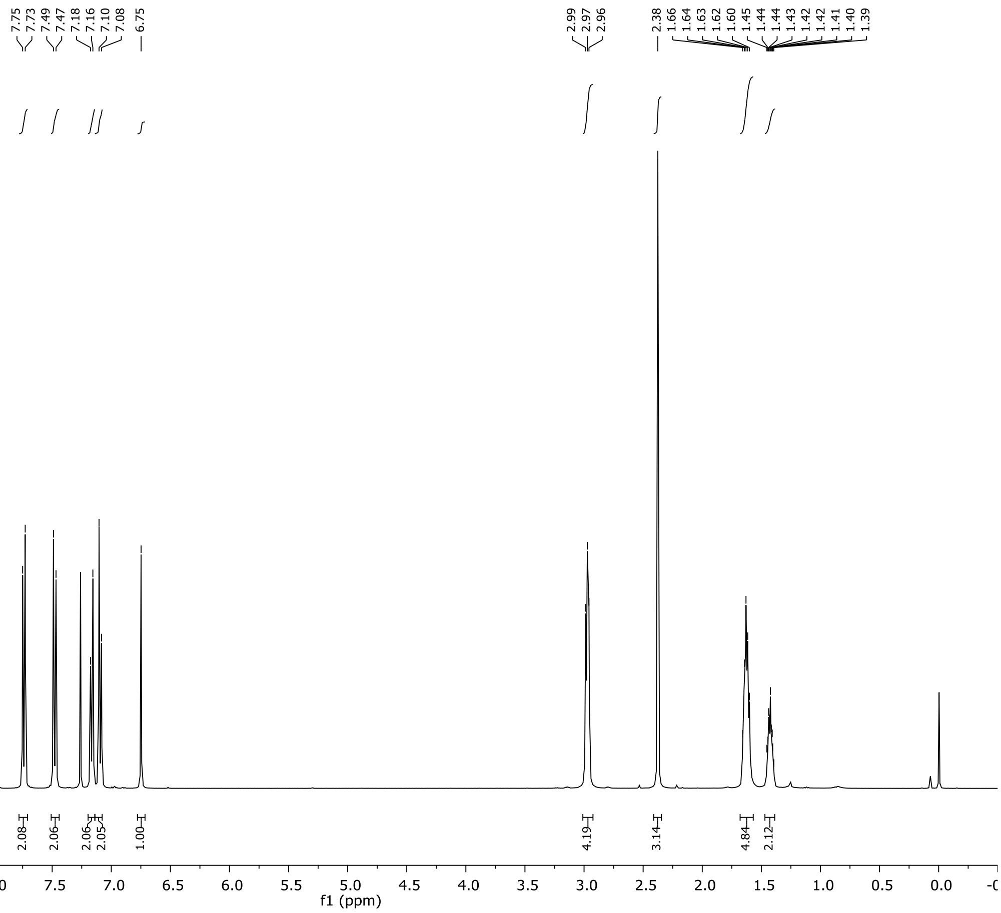

[13]C Spectra

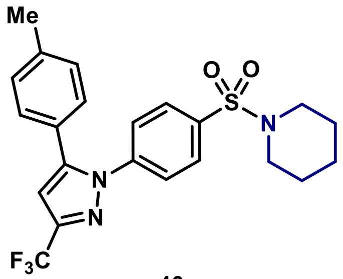

40

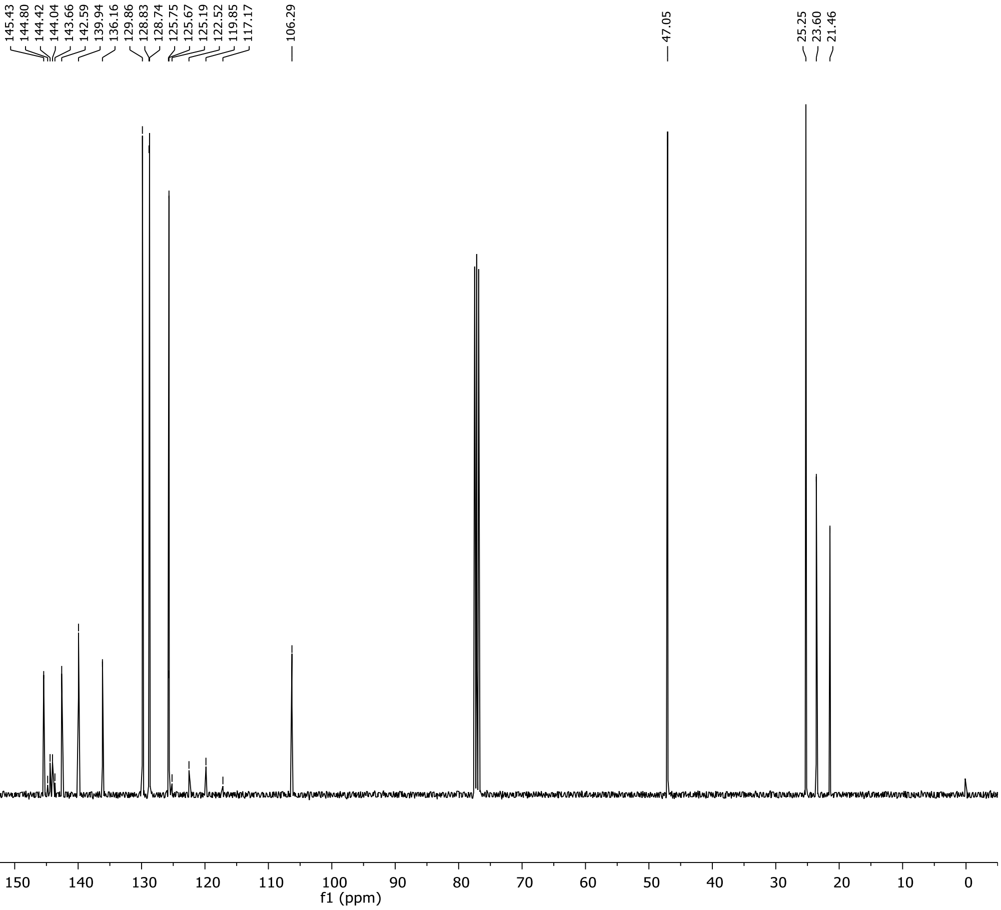

^{19}F Spectra

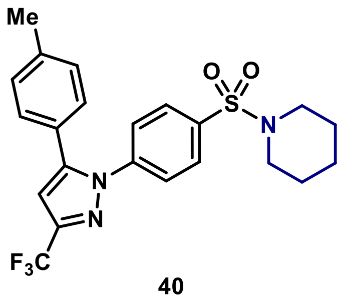

40

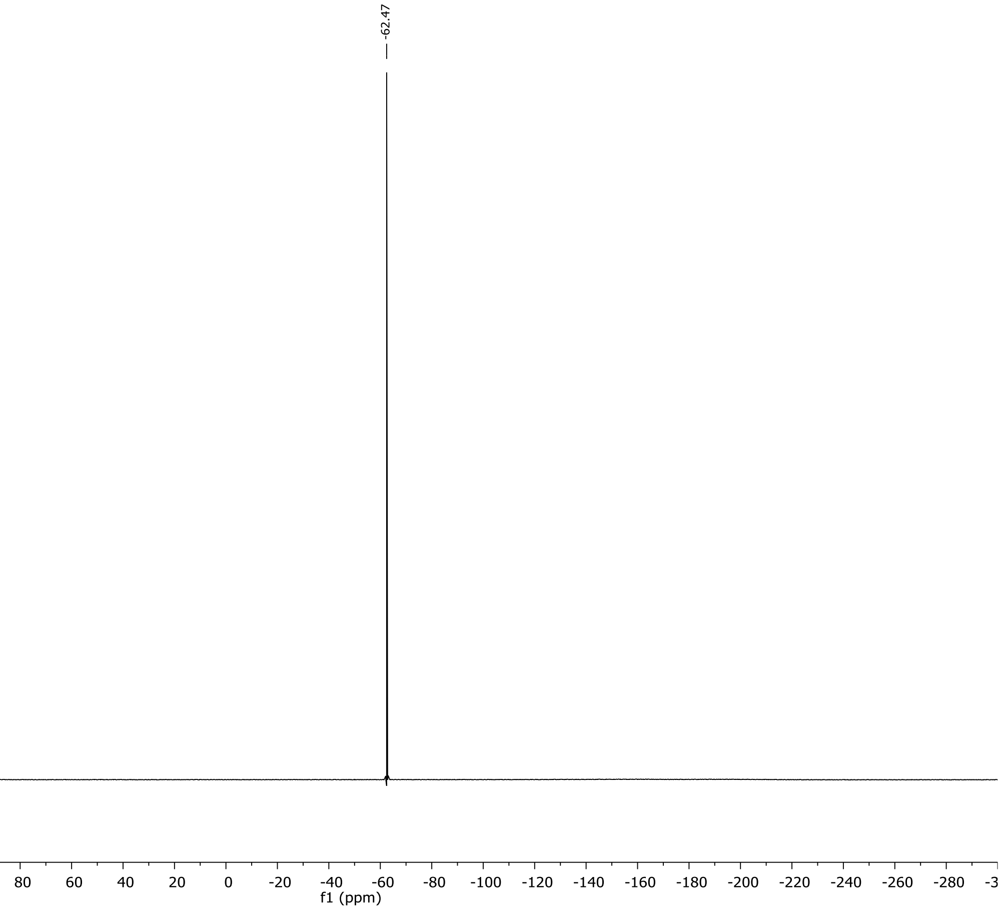

[1]H Spectra

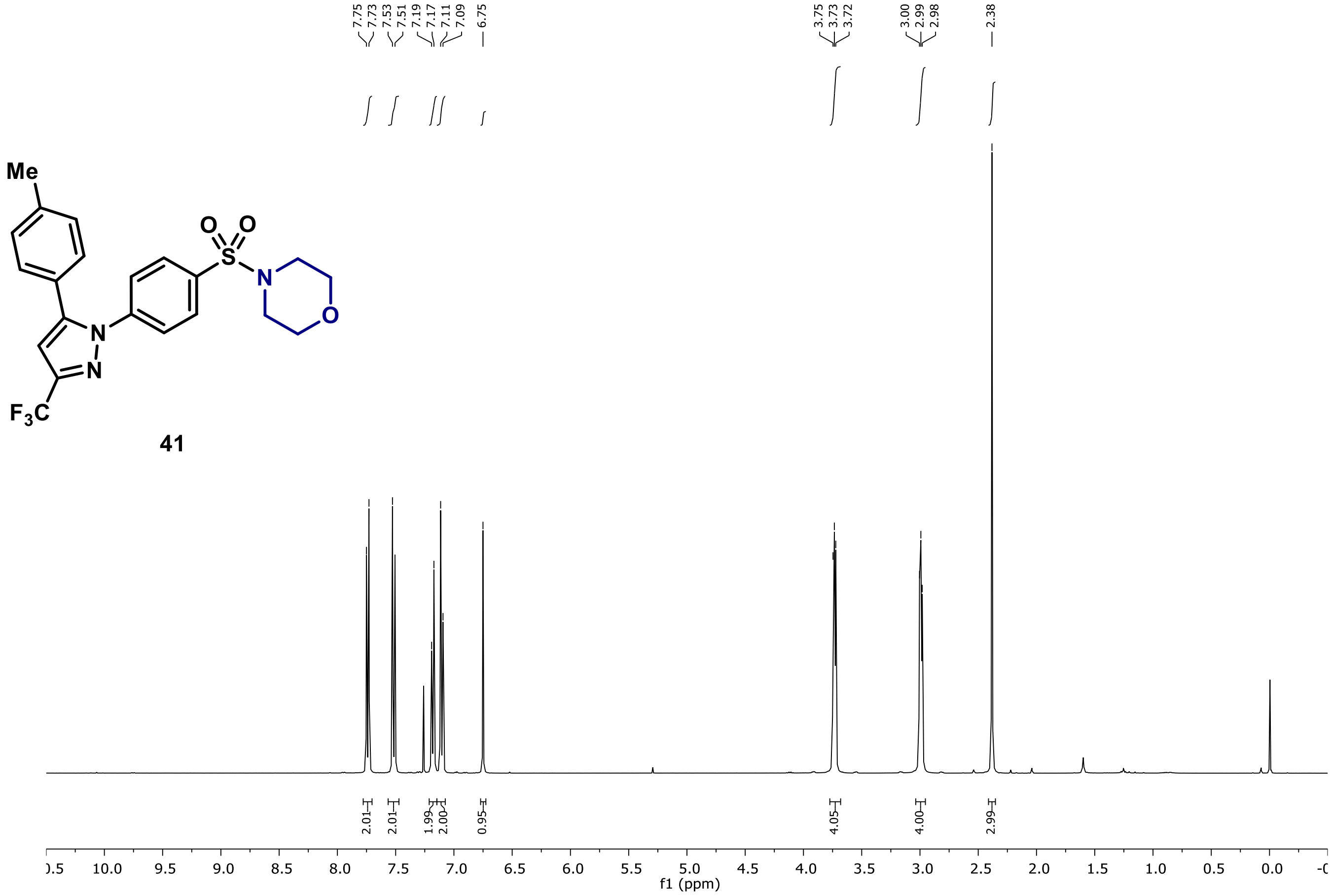

[13]C Spectra

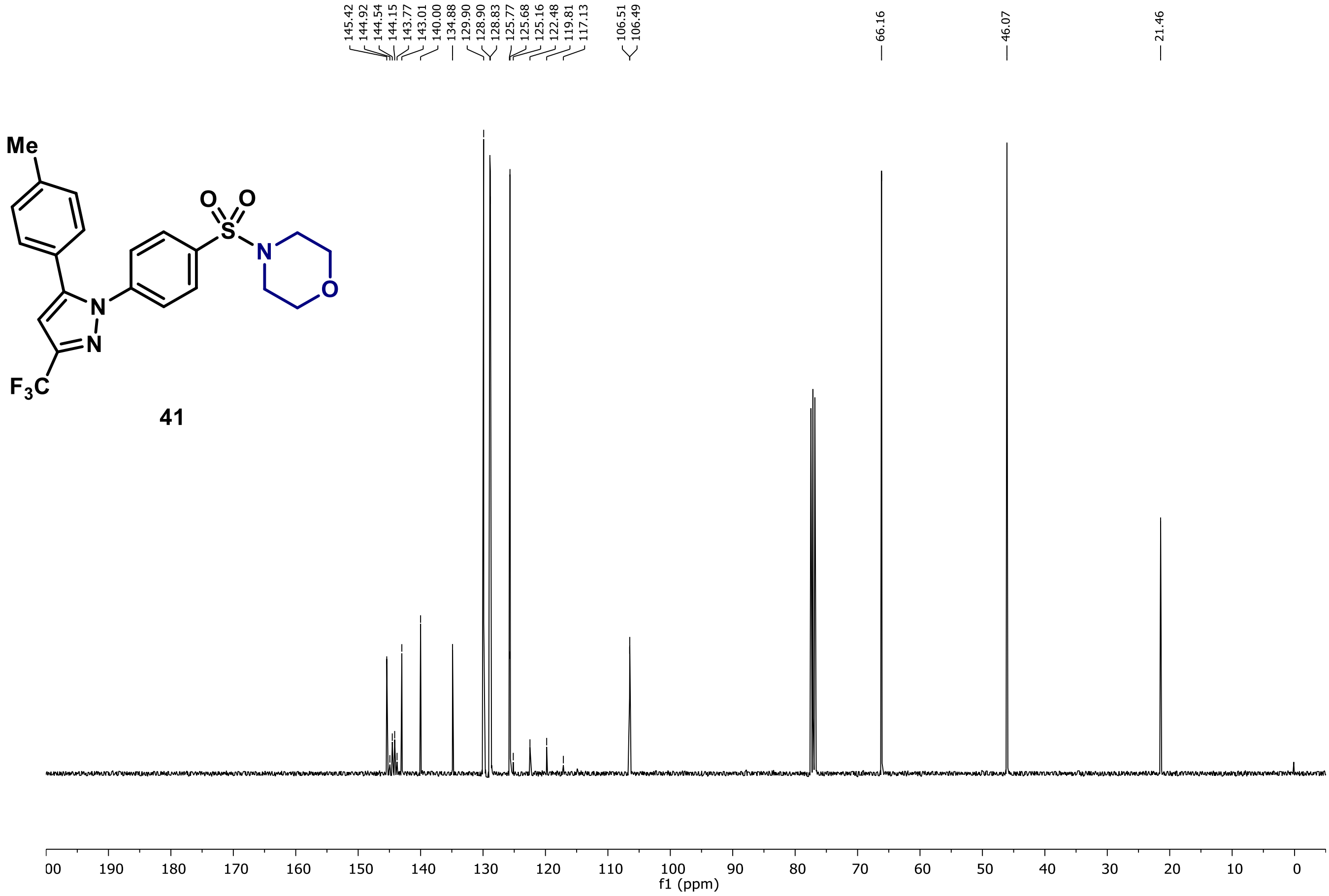

^{19}F Spectra

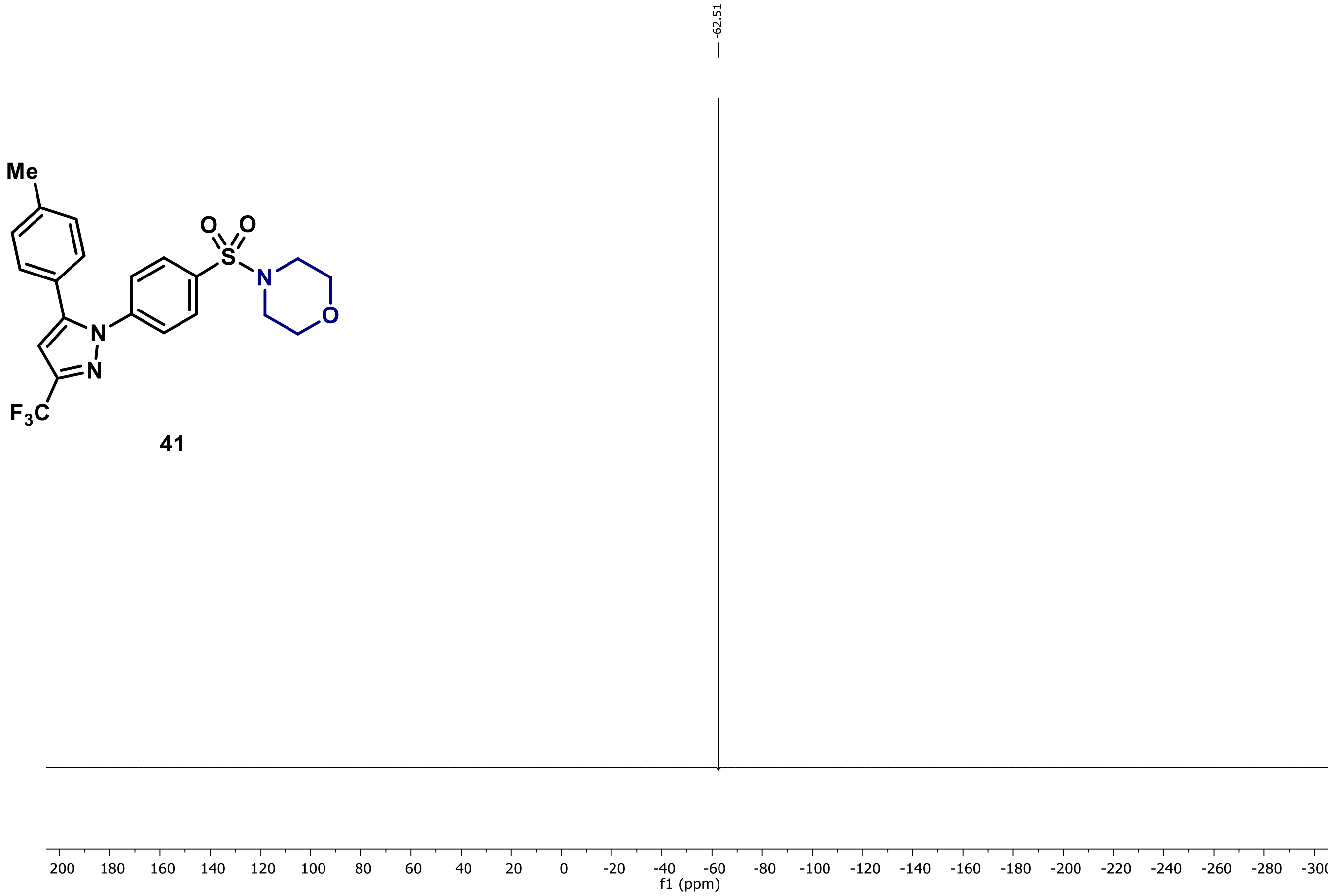

Me
O
O
S
N
O
N
N
F3C
41
-62.51
200 180 160 140 120 100 80 60 40 20 0 -20 -40 -60 -80 -100 -120 -140 -160 -180 -200 -220 -240 -260 -280
f1 (ppm)

[1]H Spectra

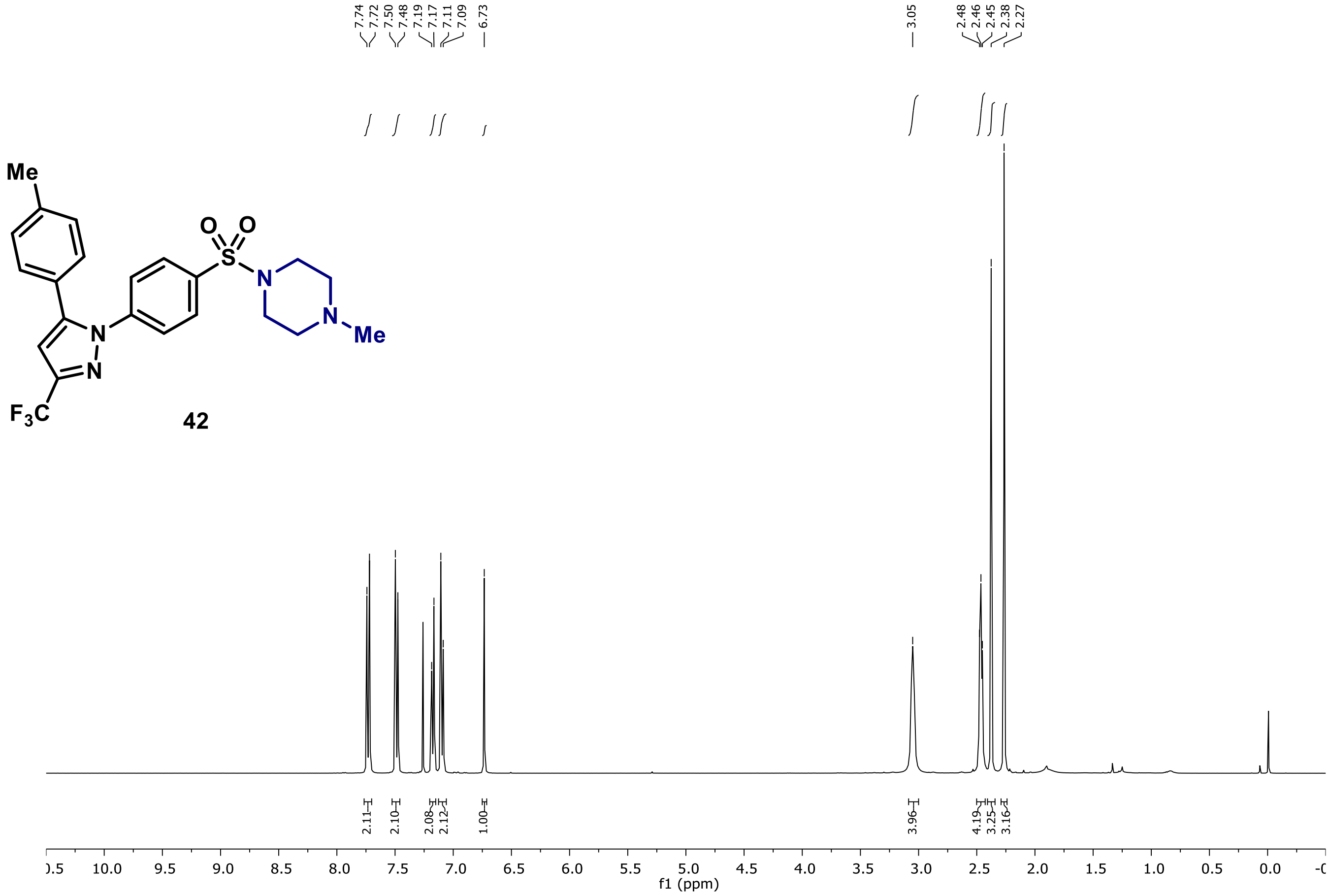

42

[13]C Spectra

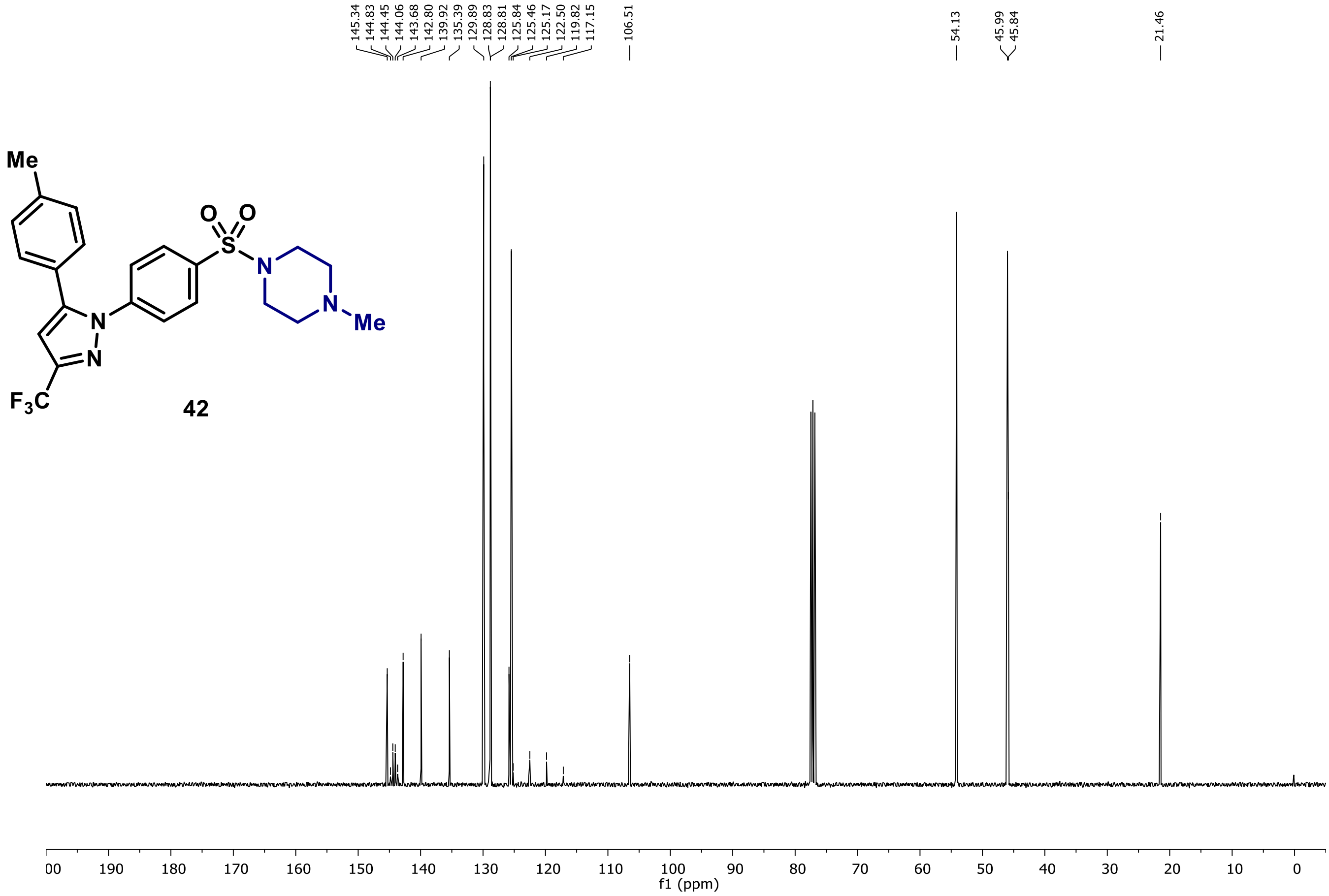

42

^{19}F Spectra

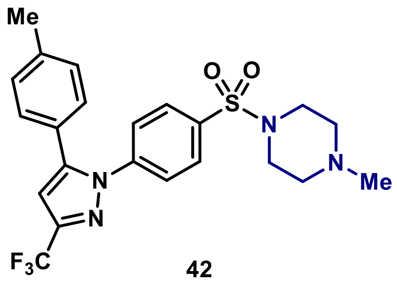

42

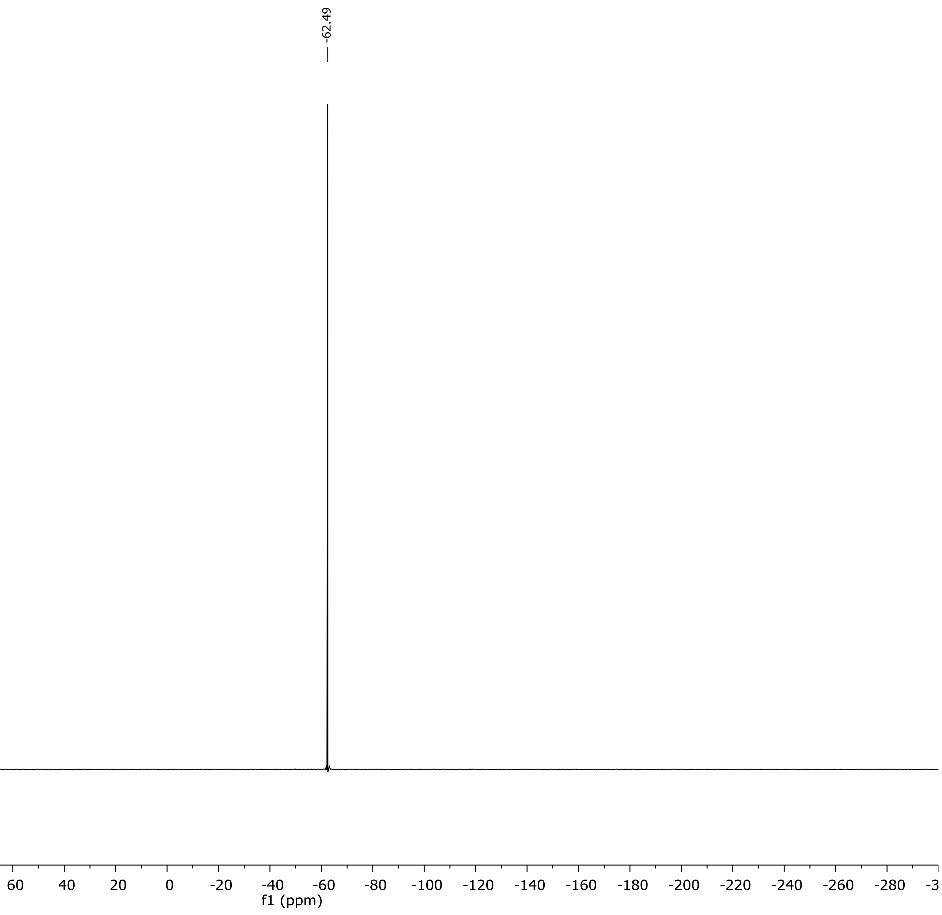

^{1}H Spectra

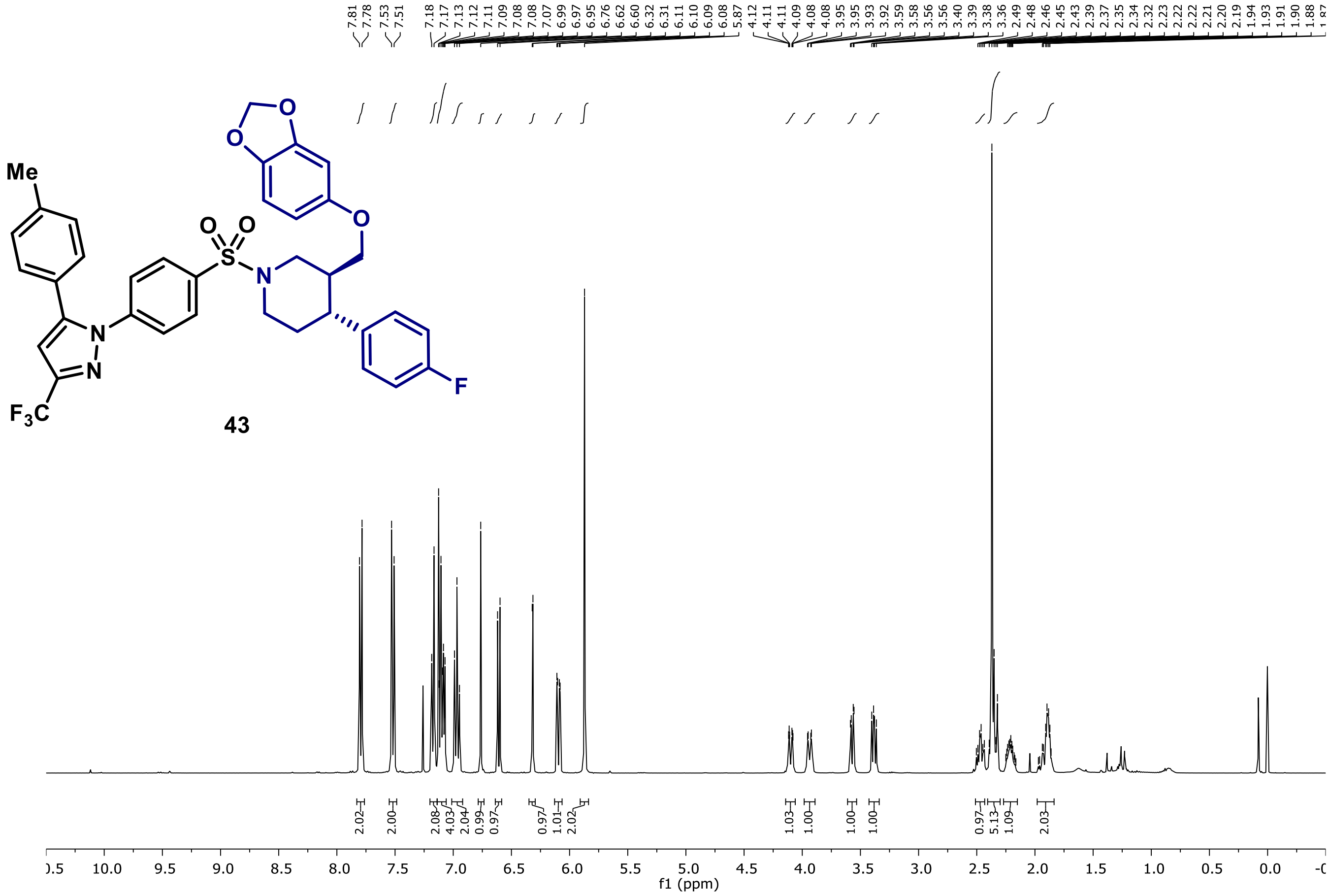

^{13}C Spectra

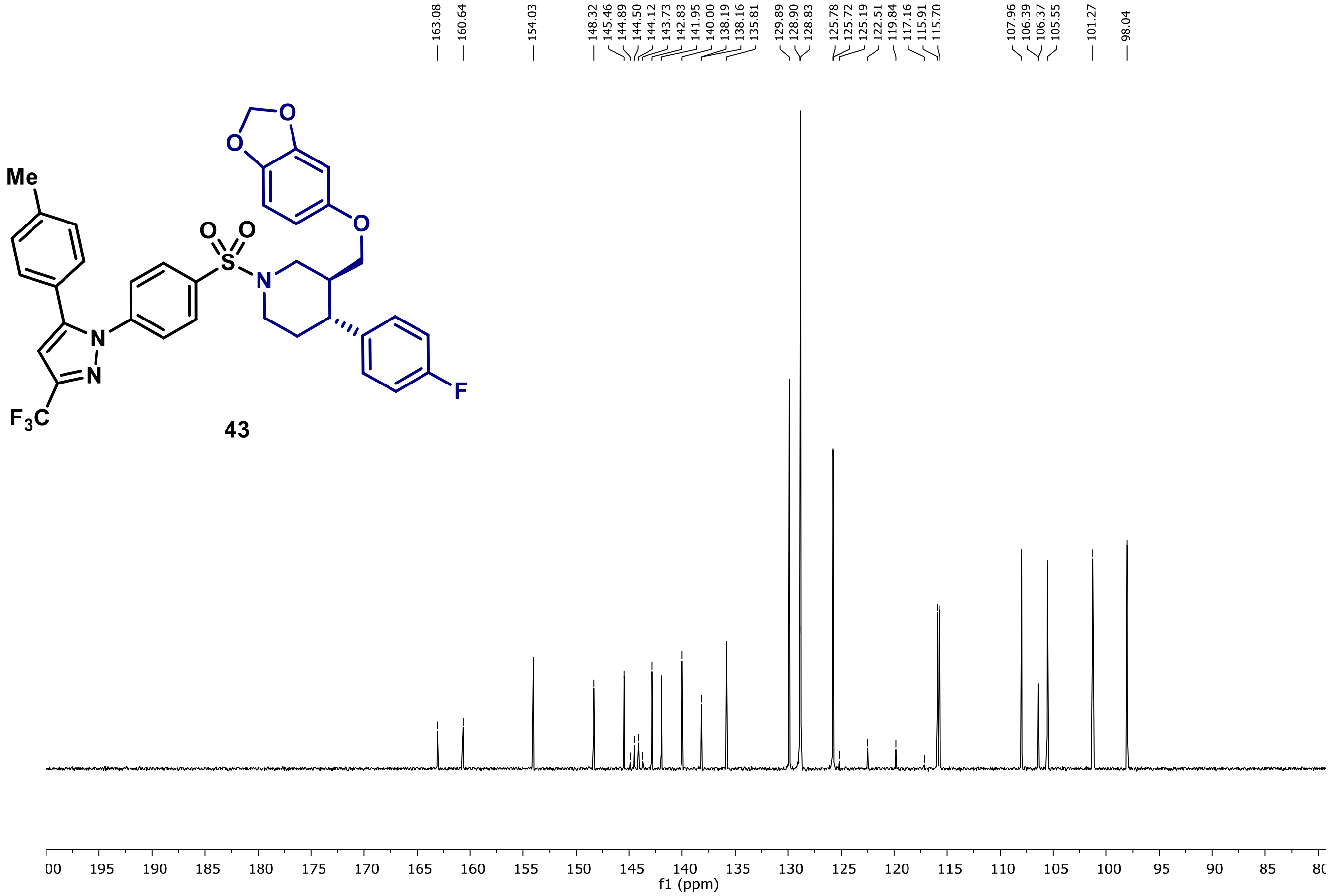

^{19}F Spectra

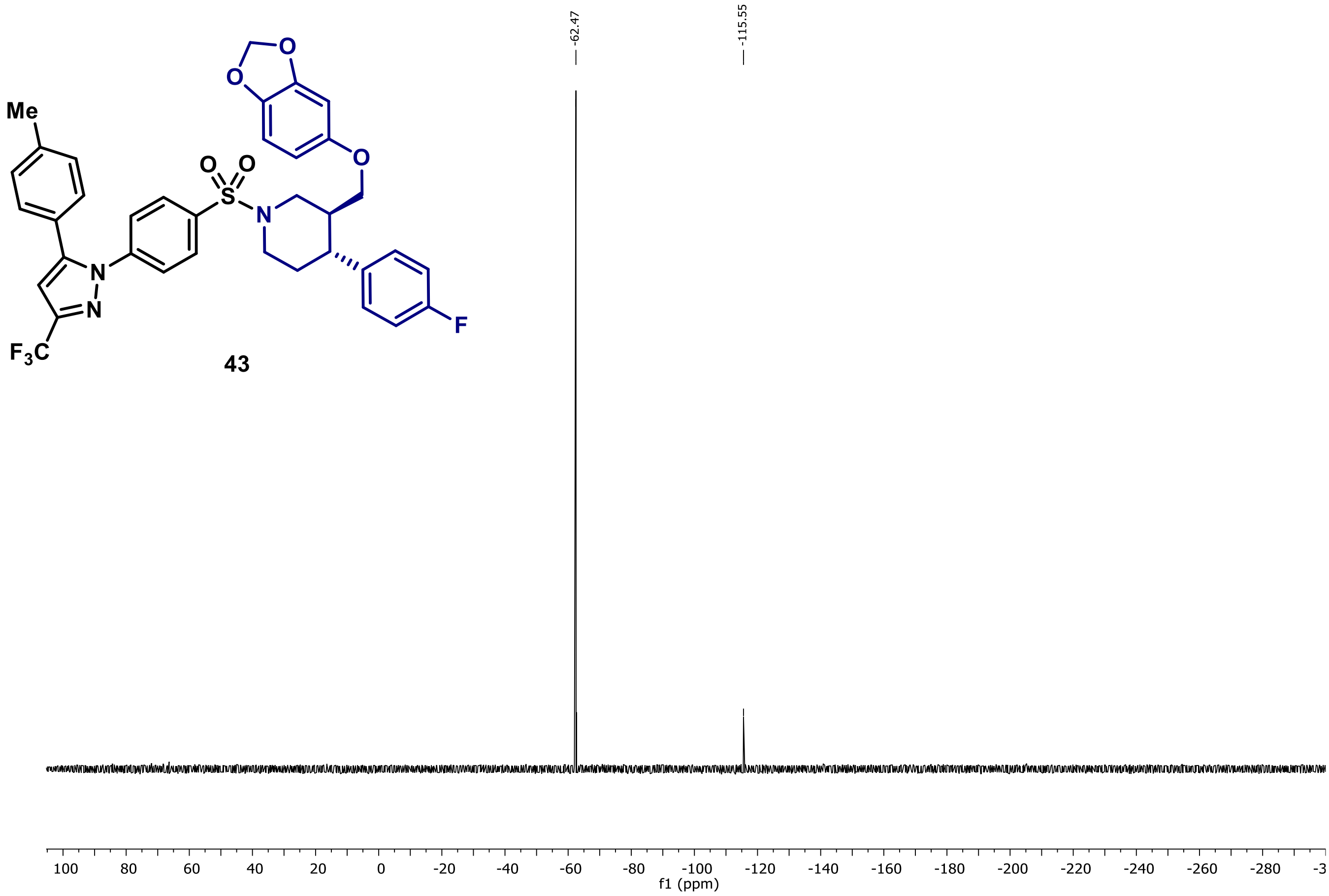

-62.47
-115.55
Me
O
O
O
O
O
S
N
N
N
F
F3C
43
100
80
60
40
20
0
-20
-40
-60
-80
-100
-120
-140
-160
-180
-200
-220
-240
-260
-280
-3
f1 (ppm)

^{1}H Spectra

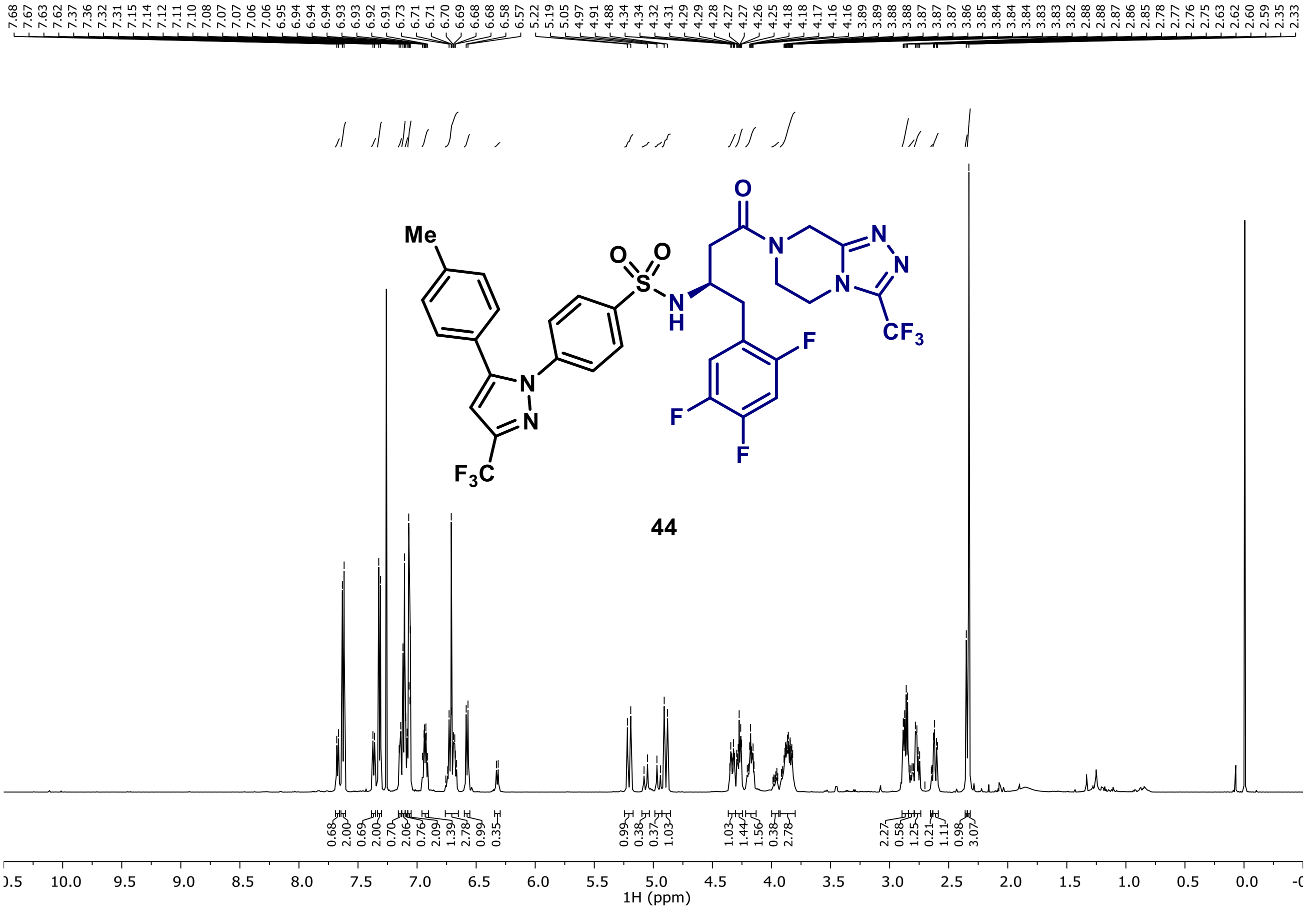

^{13}C Spectra

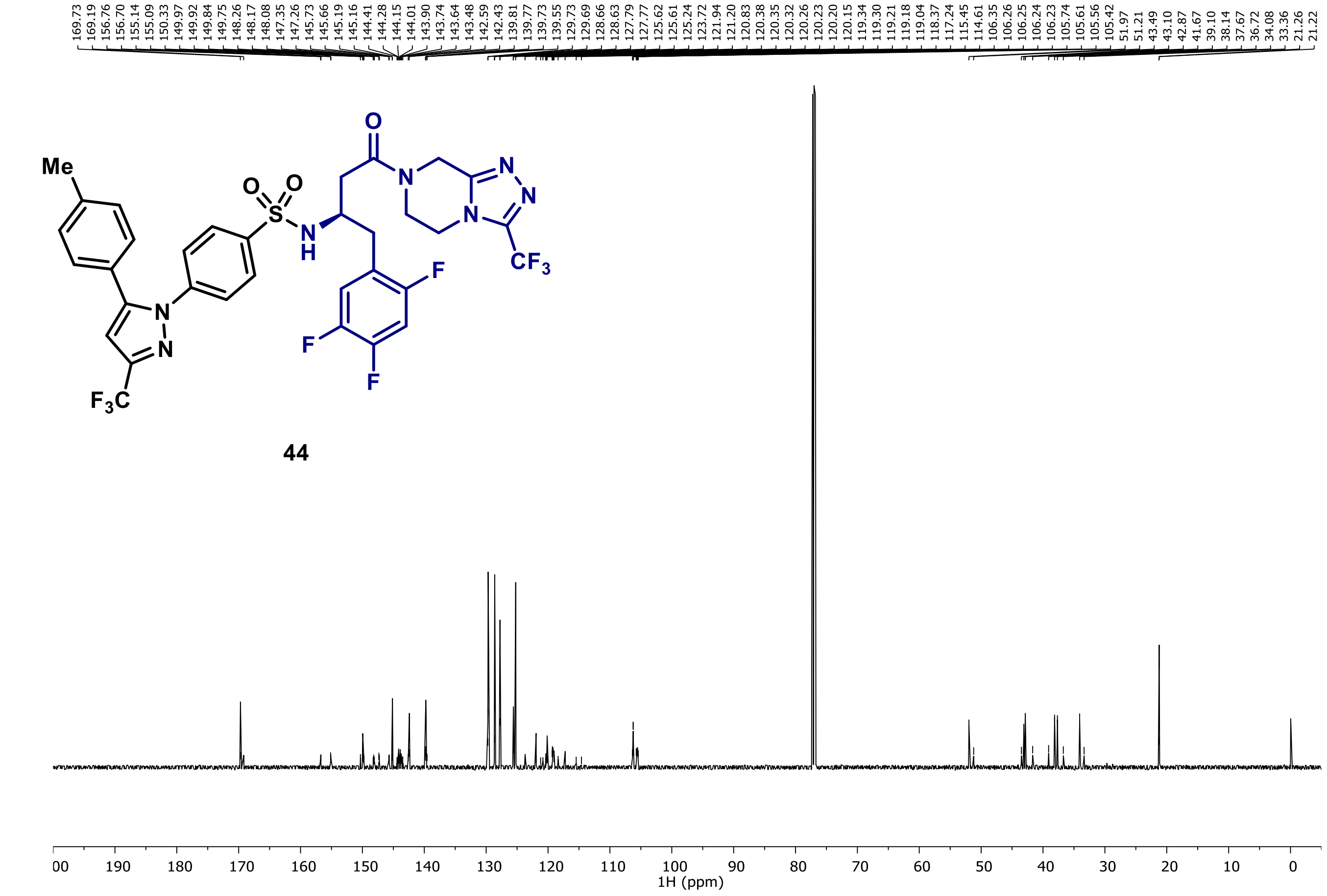

[19]F Spectra

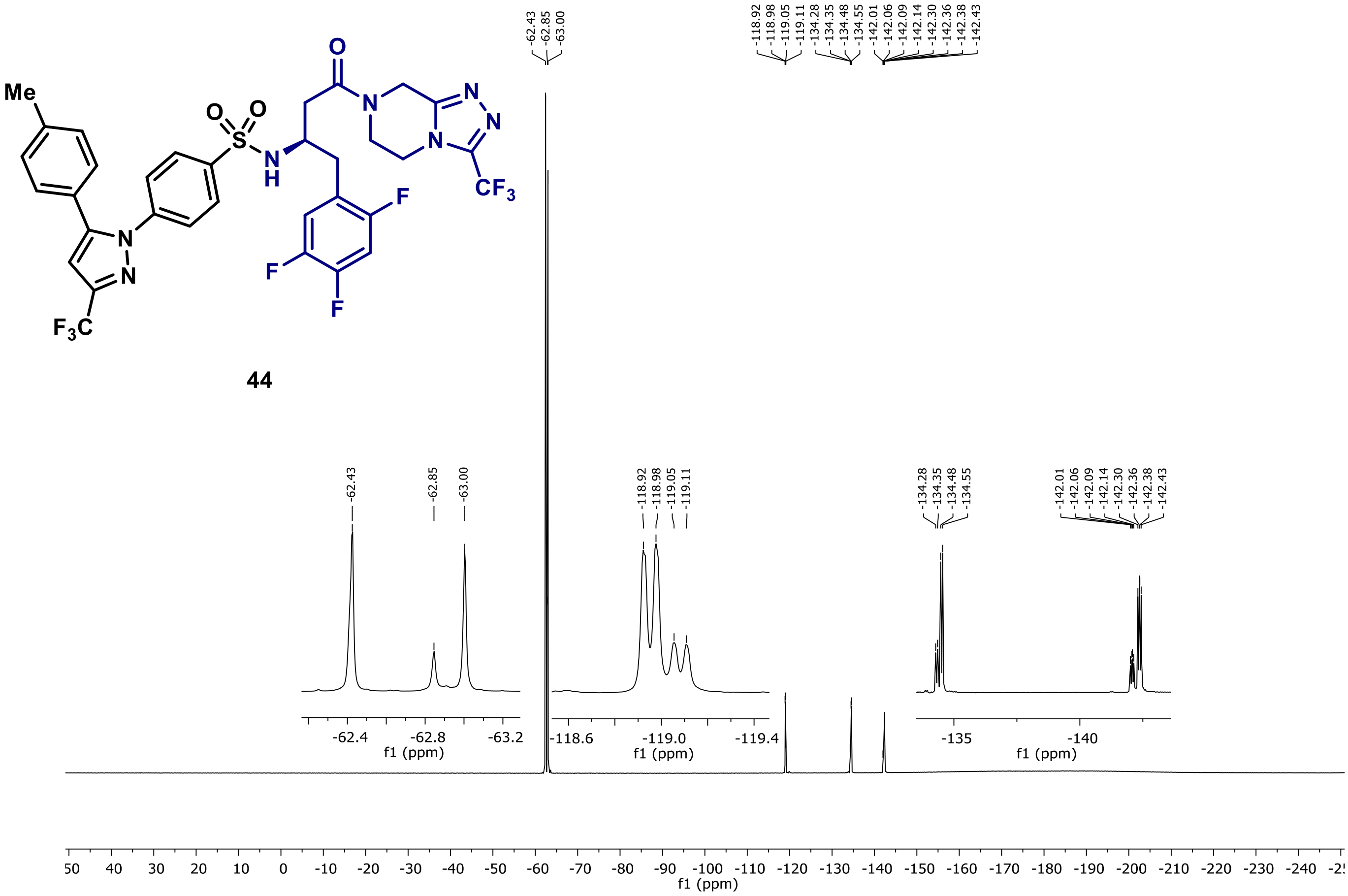

44

^{1}H Spectra

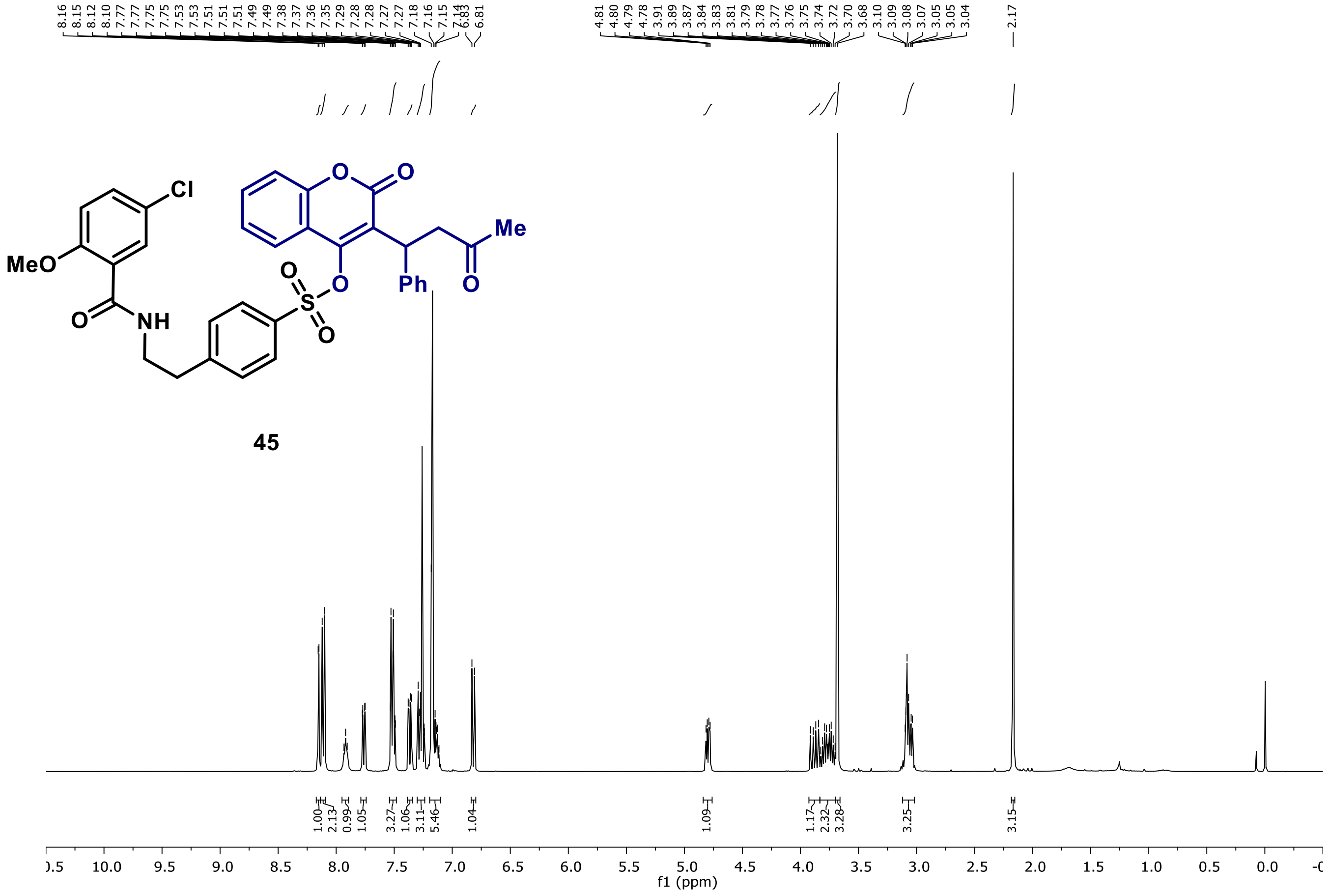

^{13}C Spectra

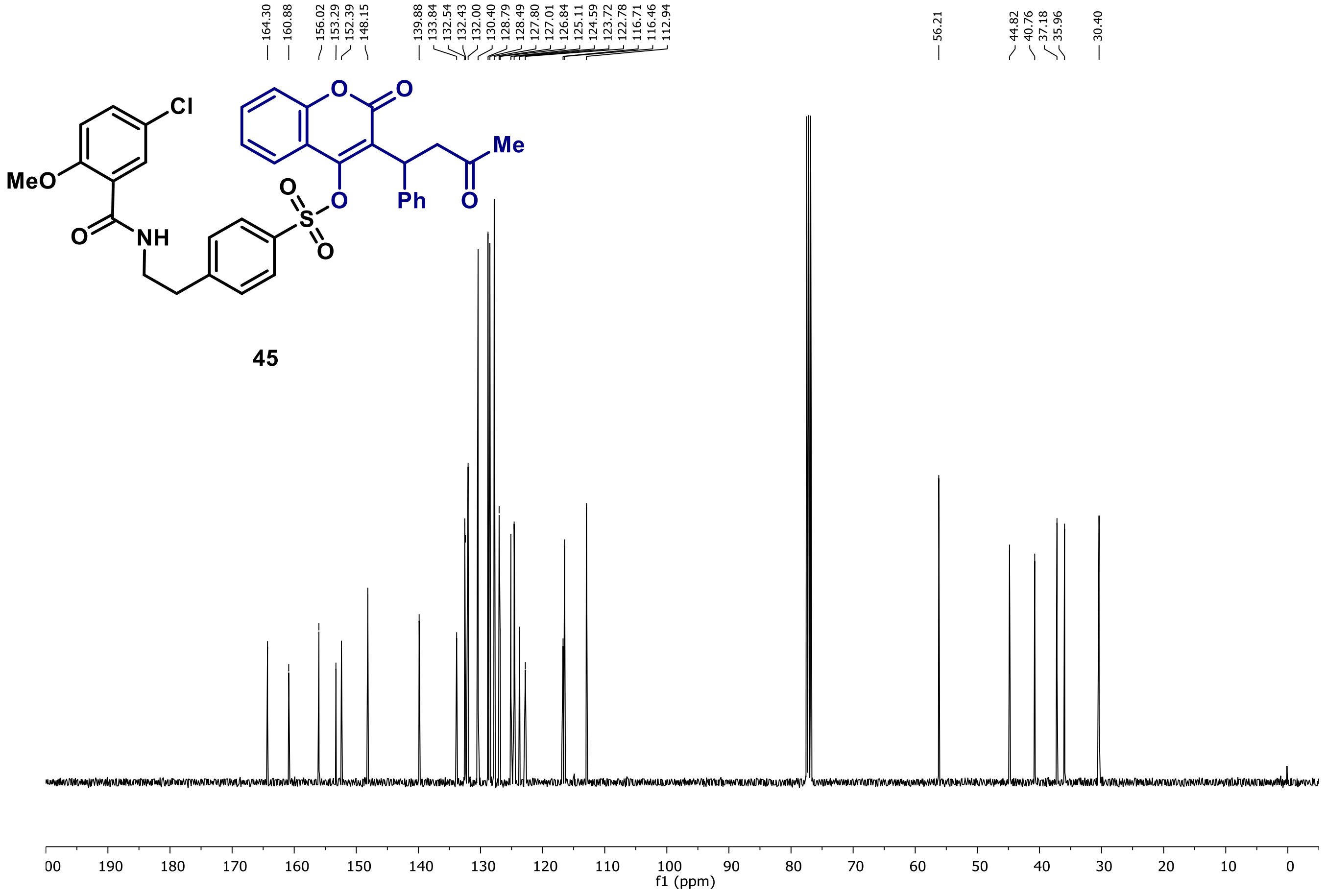

^{1}H Spectra

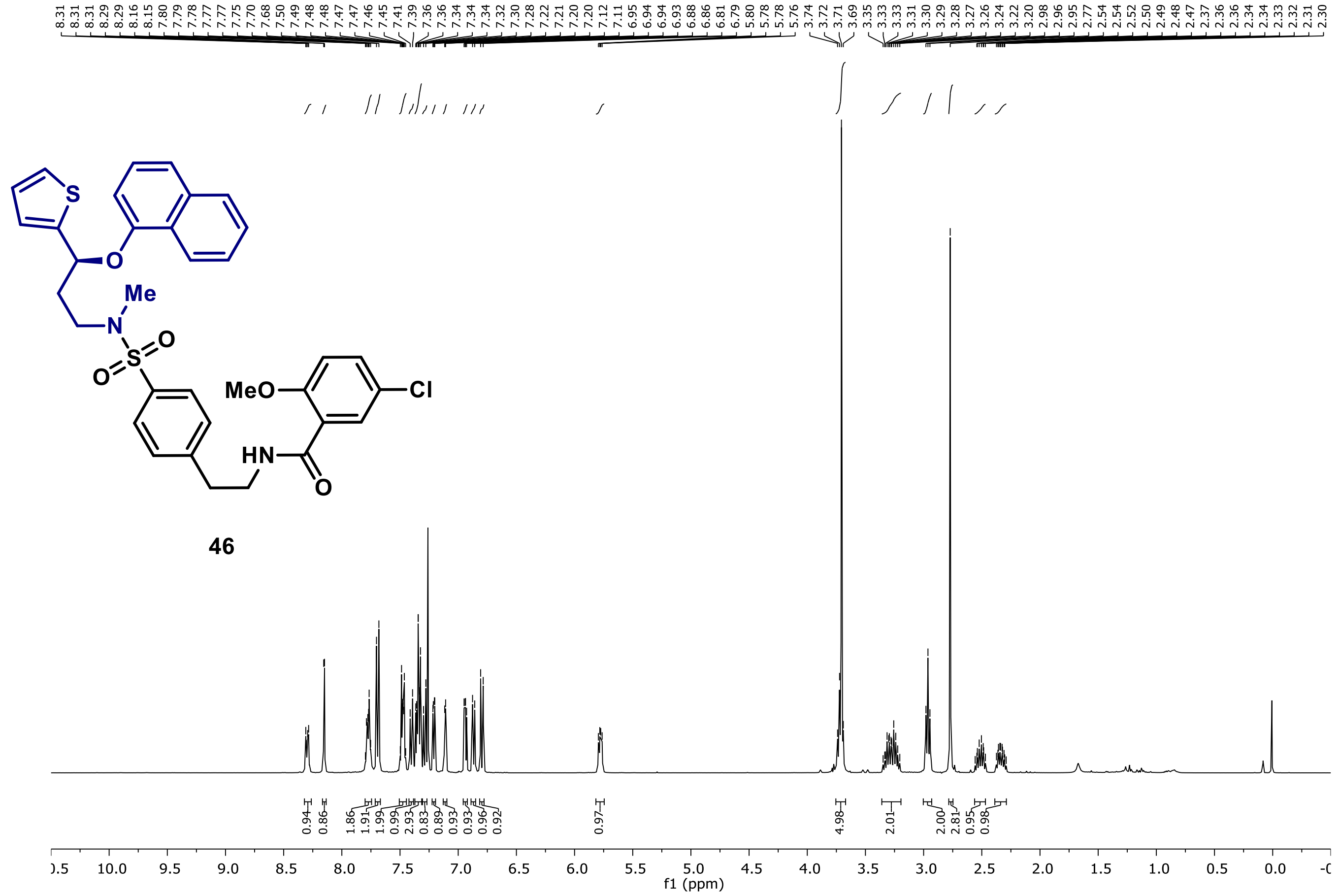

^{13}C Spectra

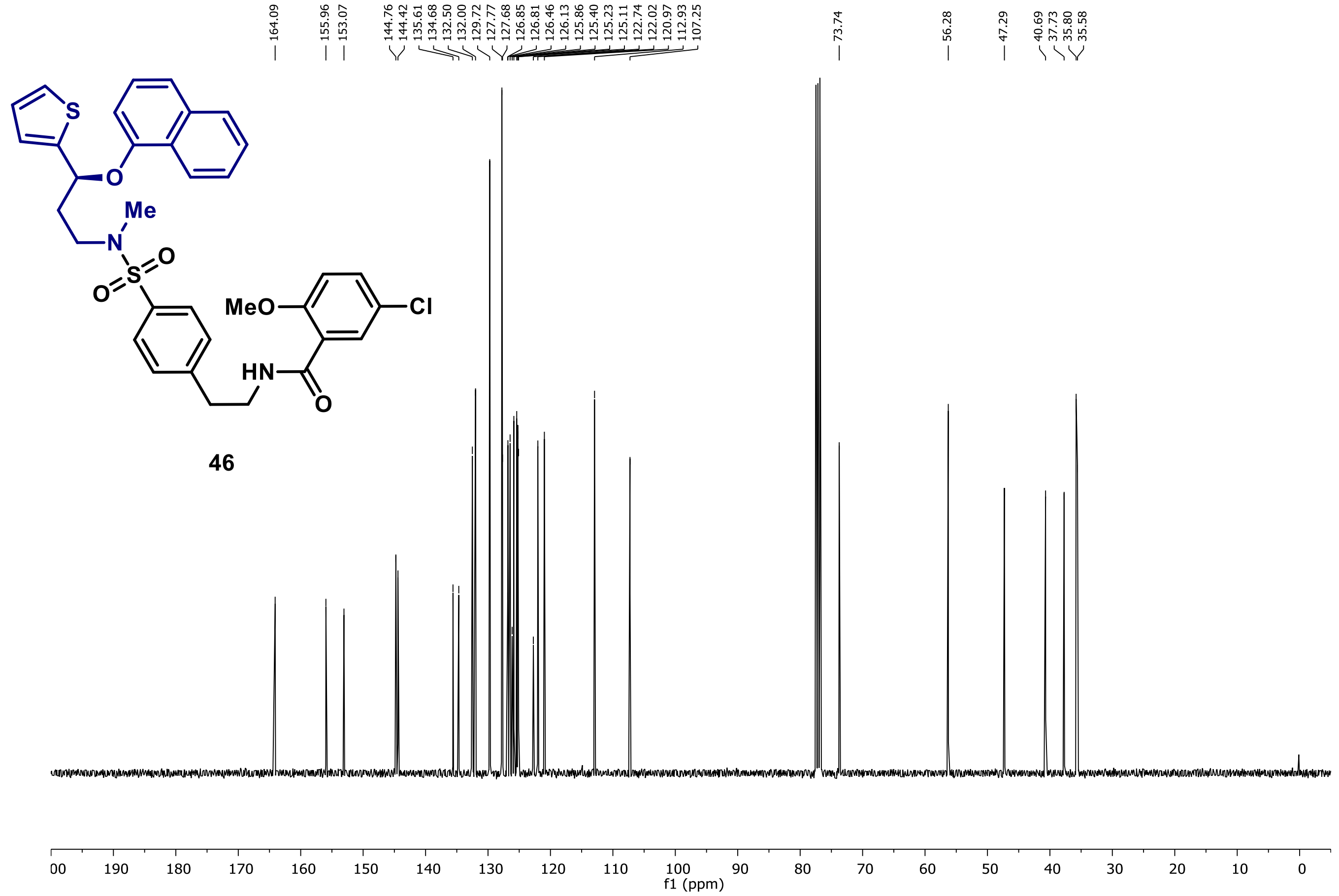

[1]H Spectra

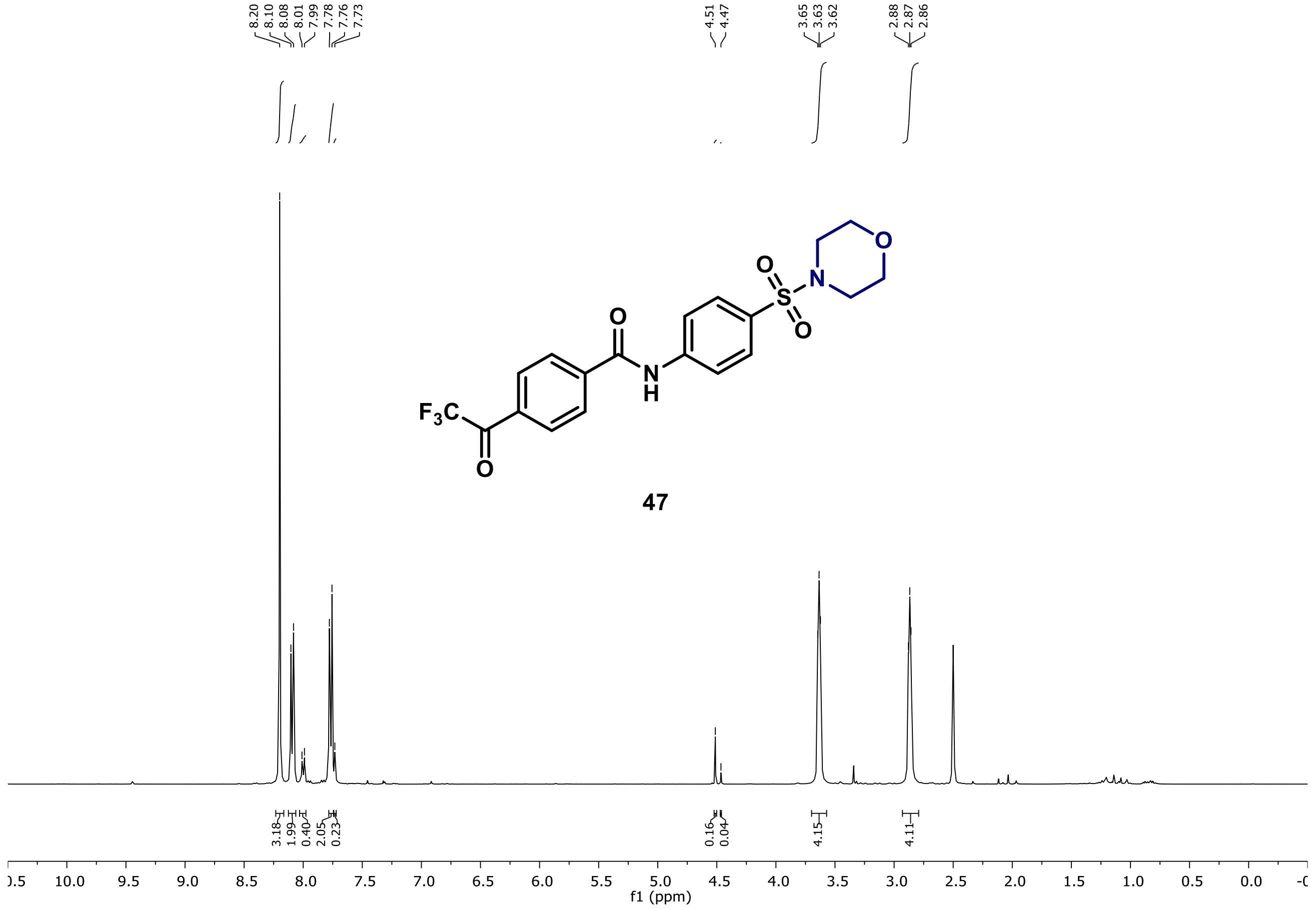

[13]C Spectra

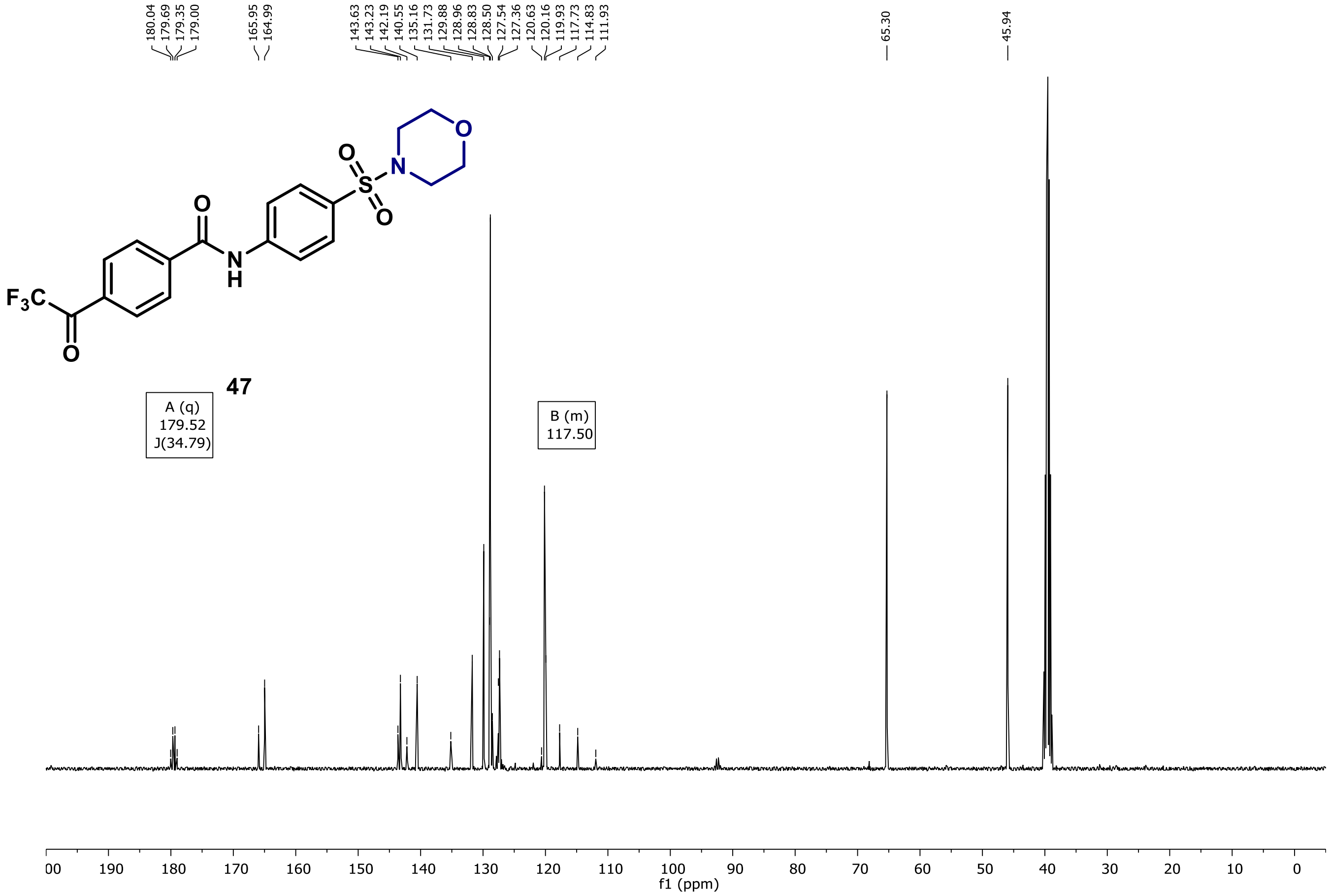

^{19}F Spectra

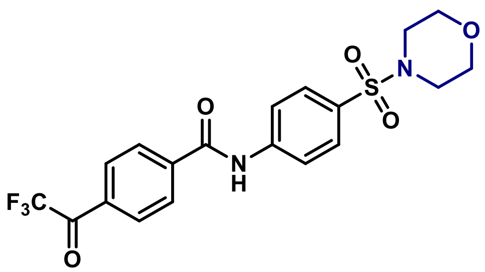

47

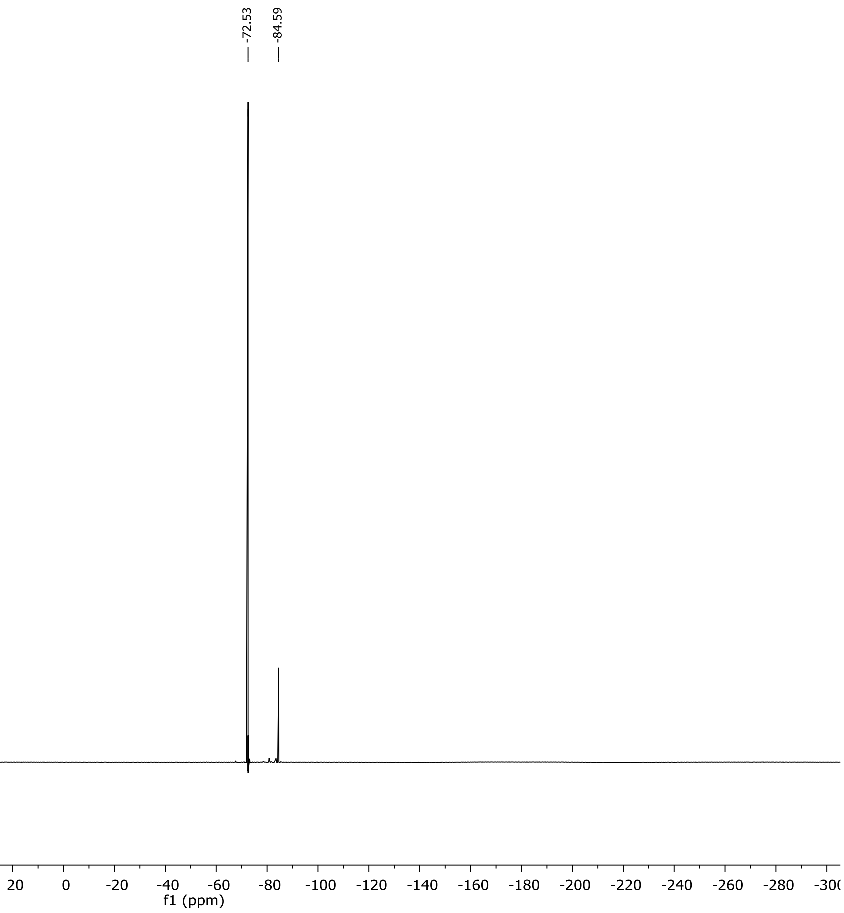

[1]H Spectra

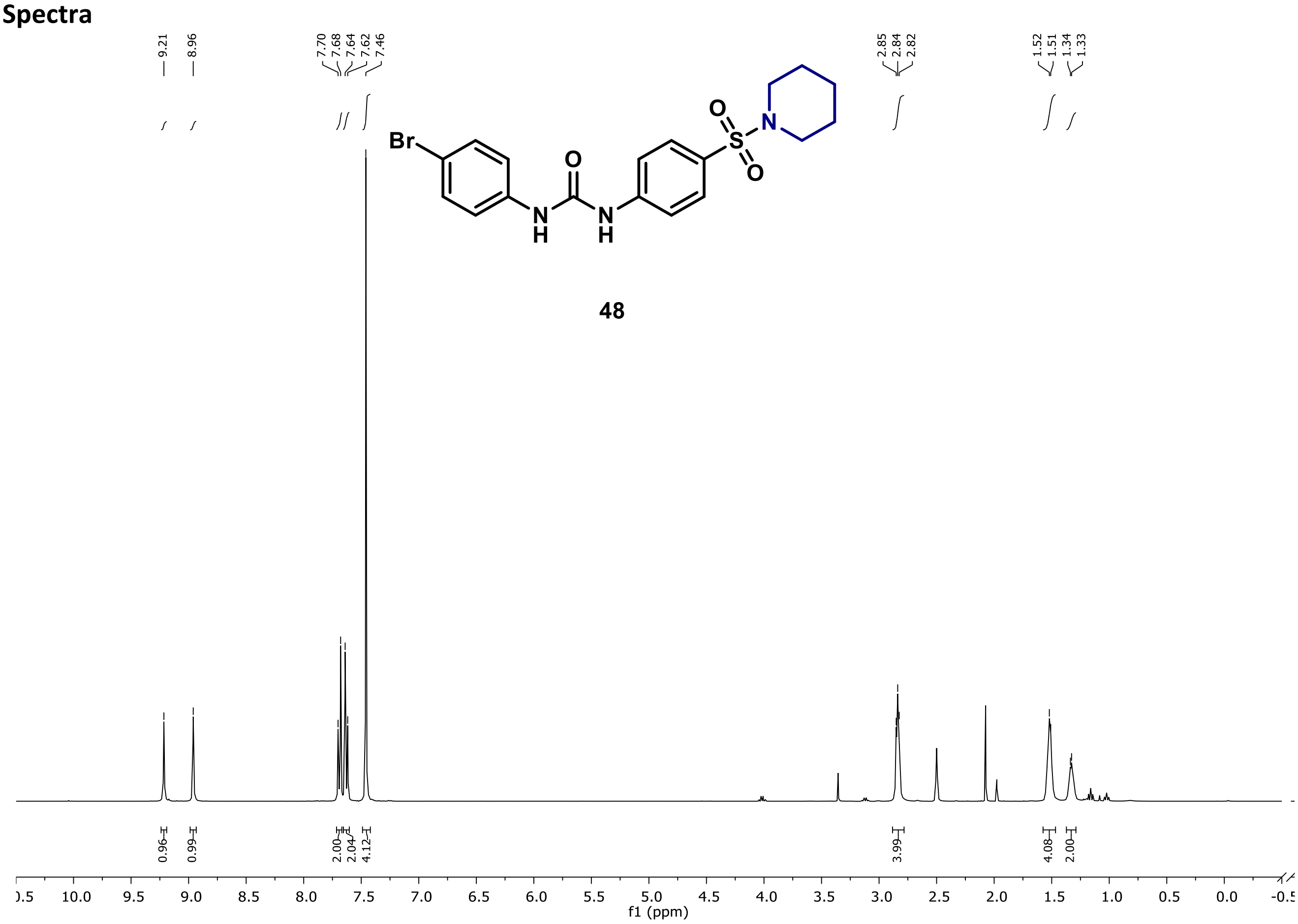

[13]C Spectra

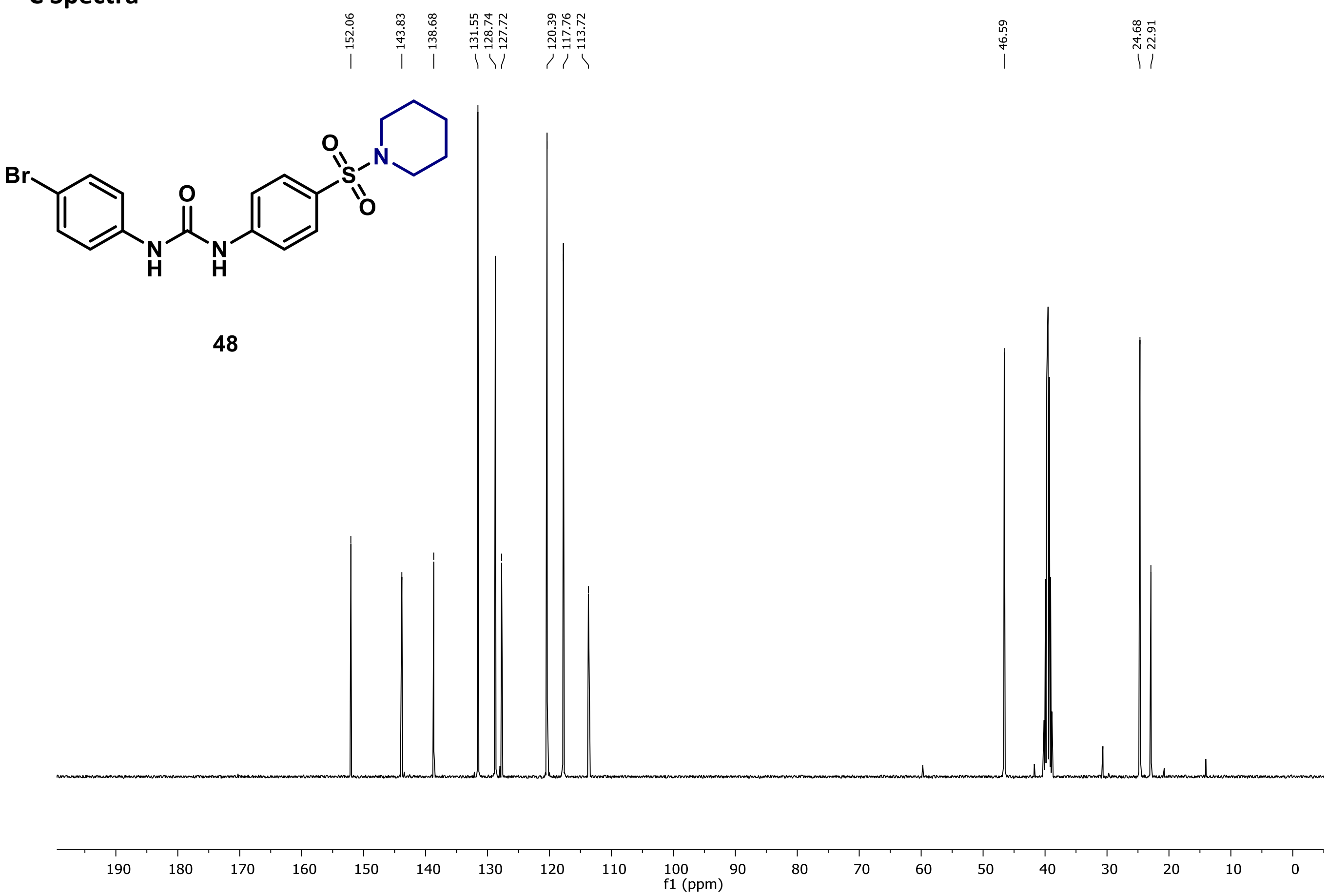

^{1}H Spectra

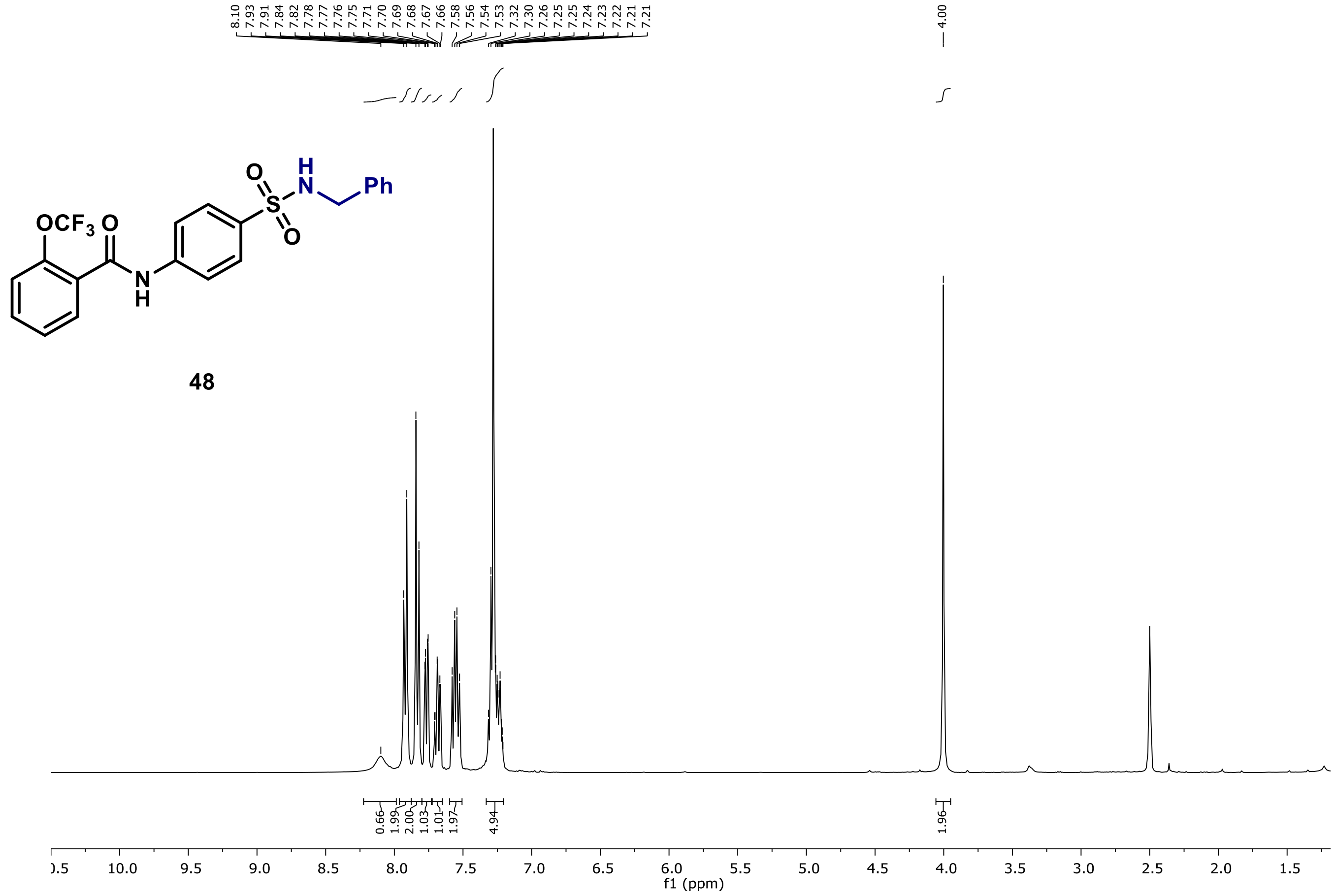

^{13}C Spectra

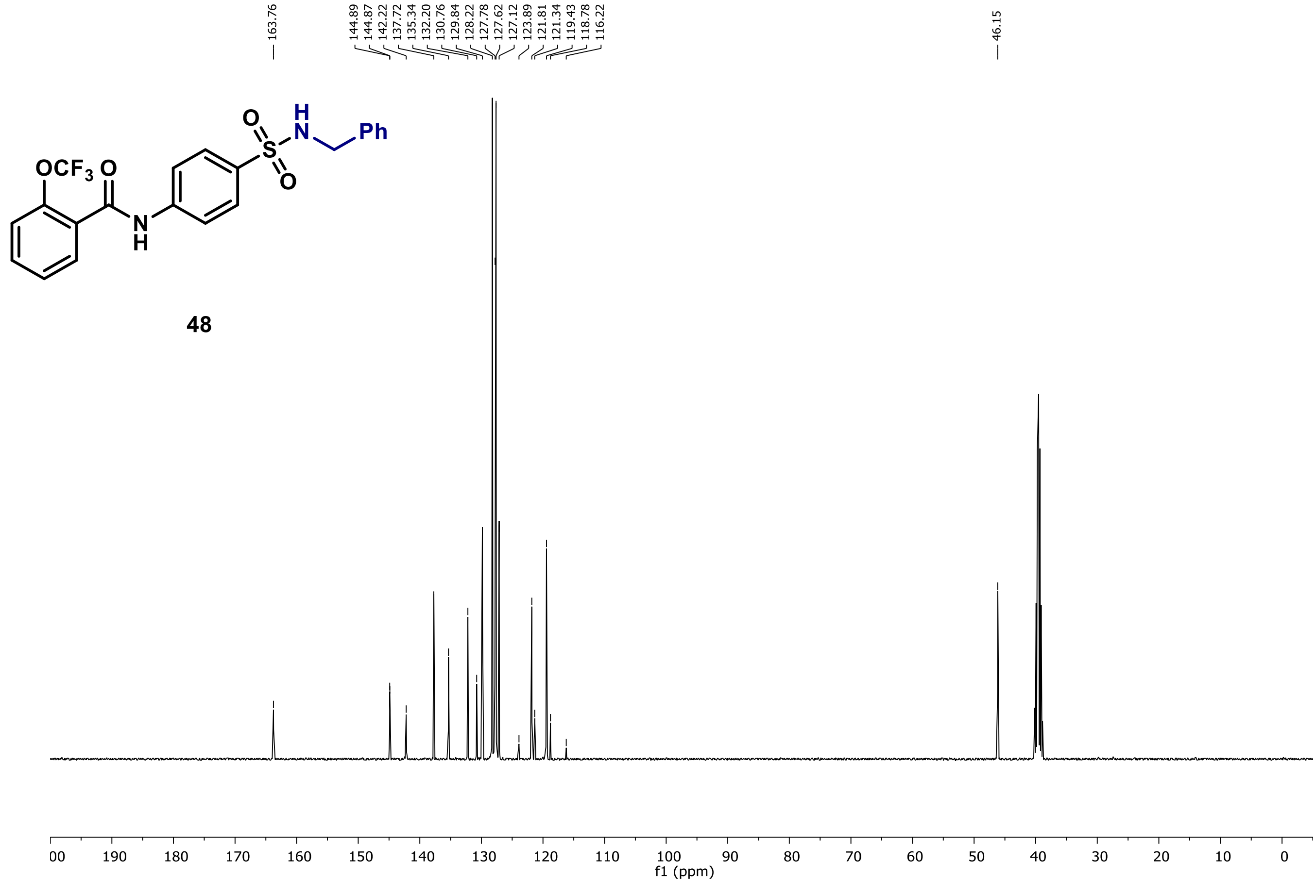

[19]F Spectra

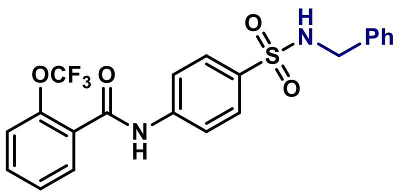

48

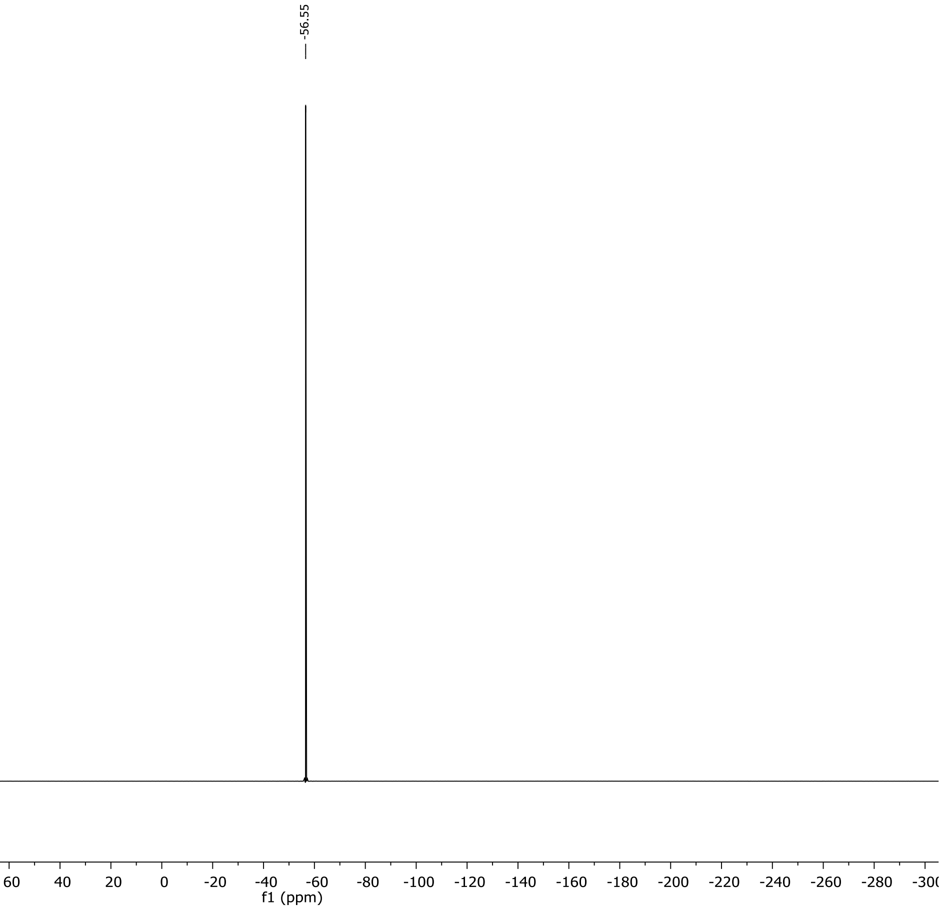

^{1}H Spectra

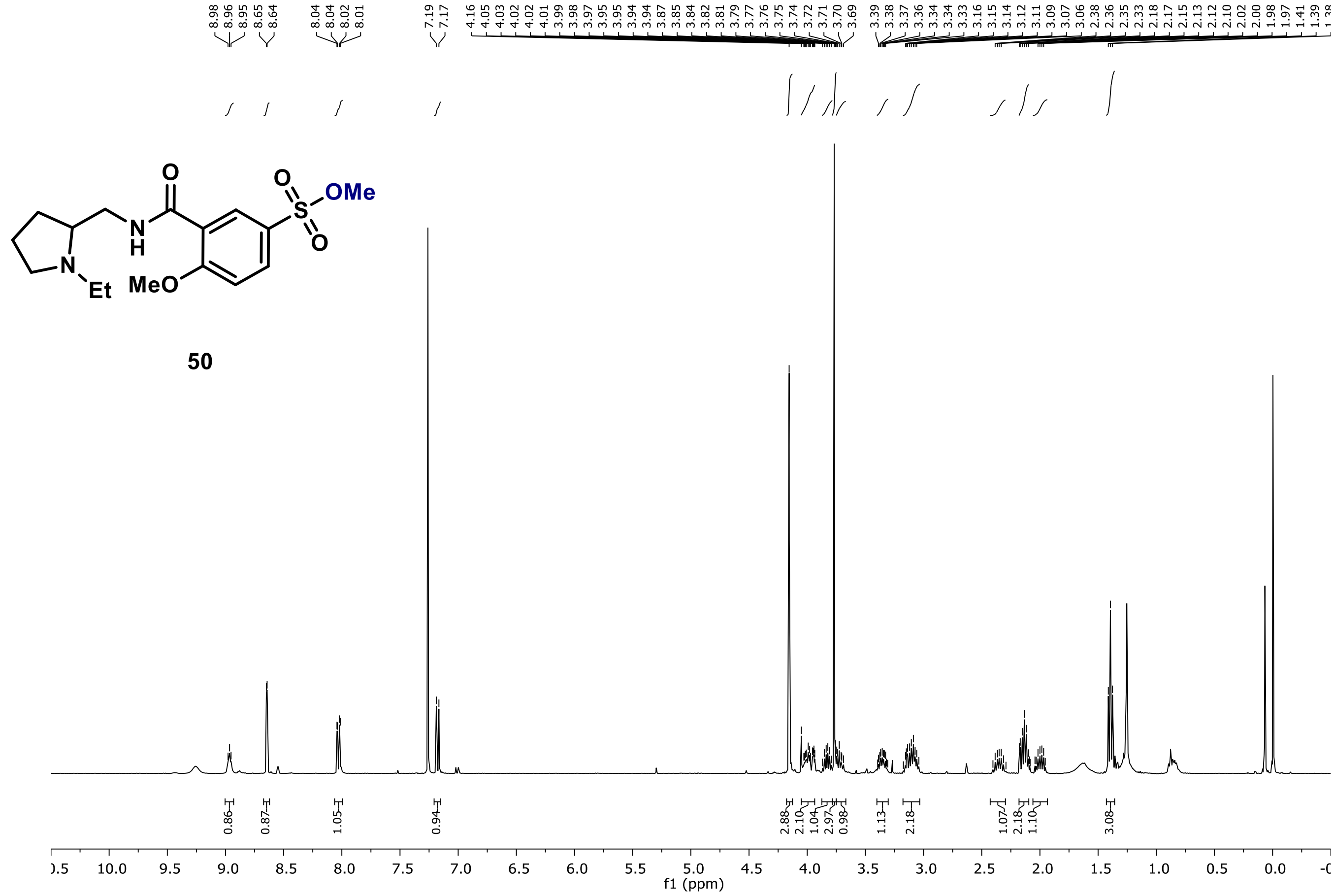

^{13}C Spectra

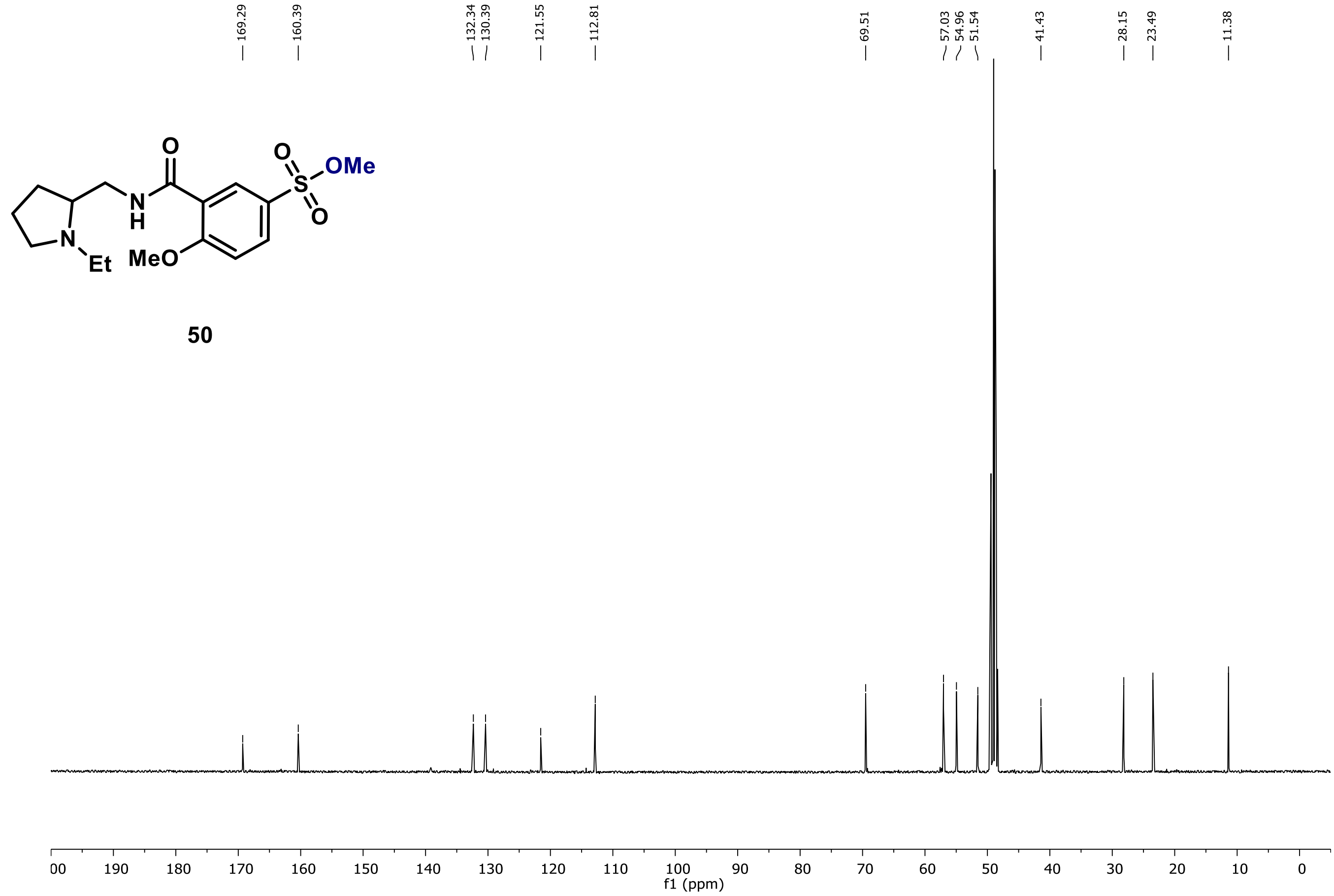

^{1}H Spectra

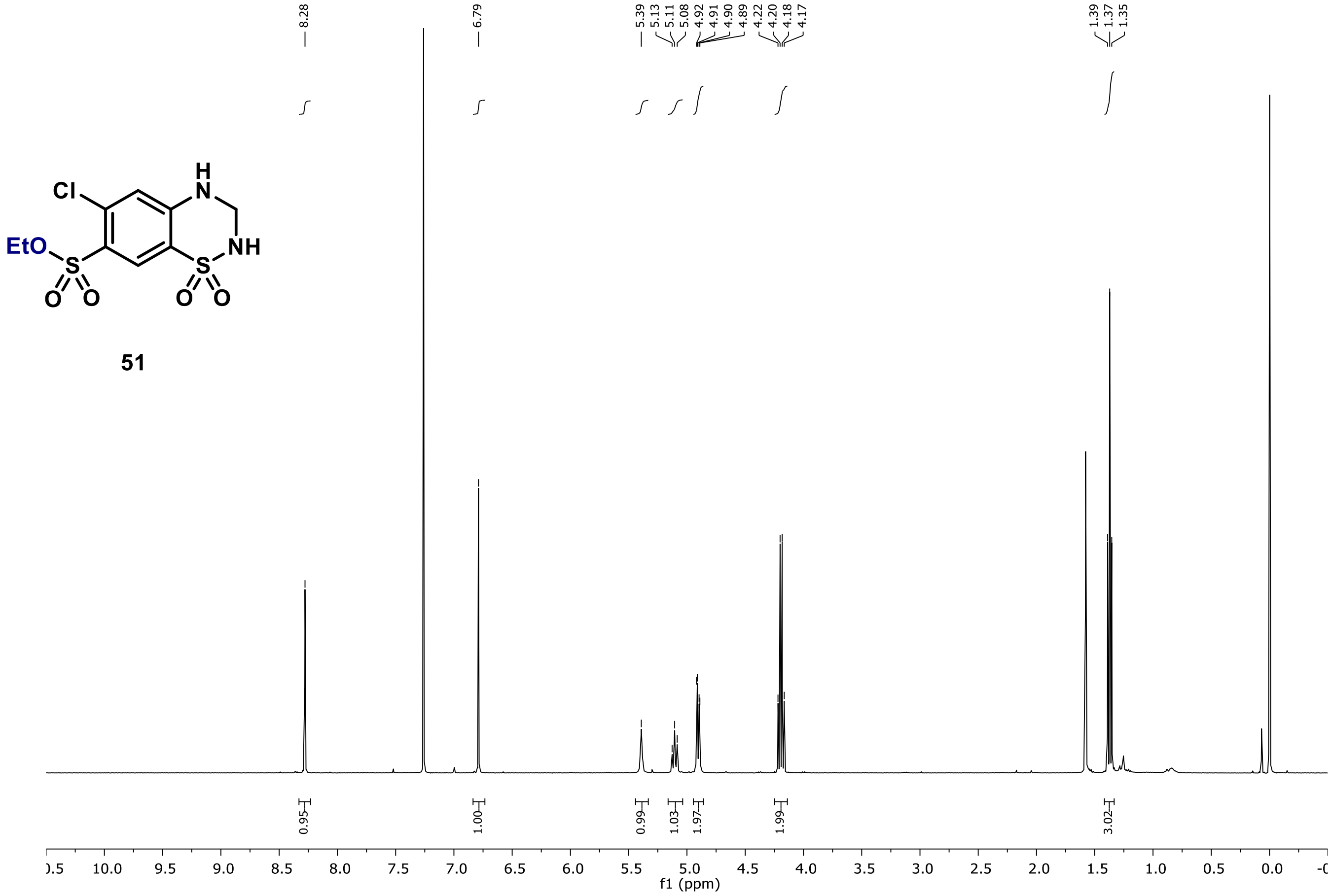

^{13}C Spectra

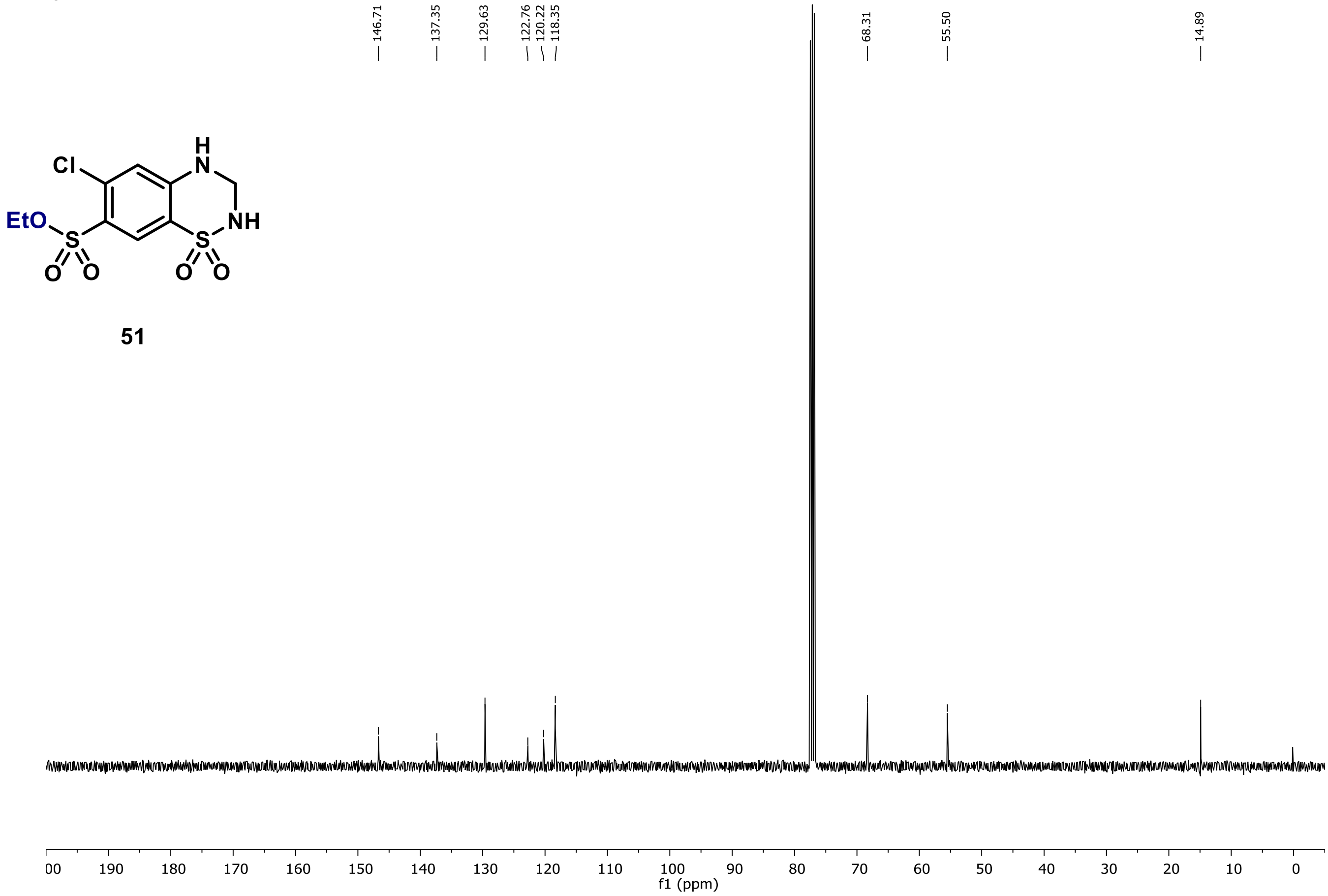

[1]H Spectra

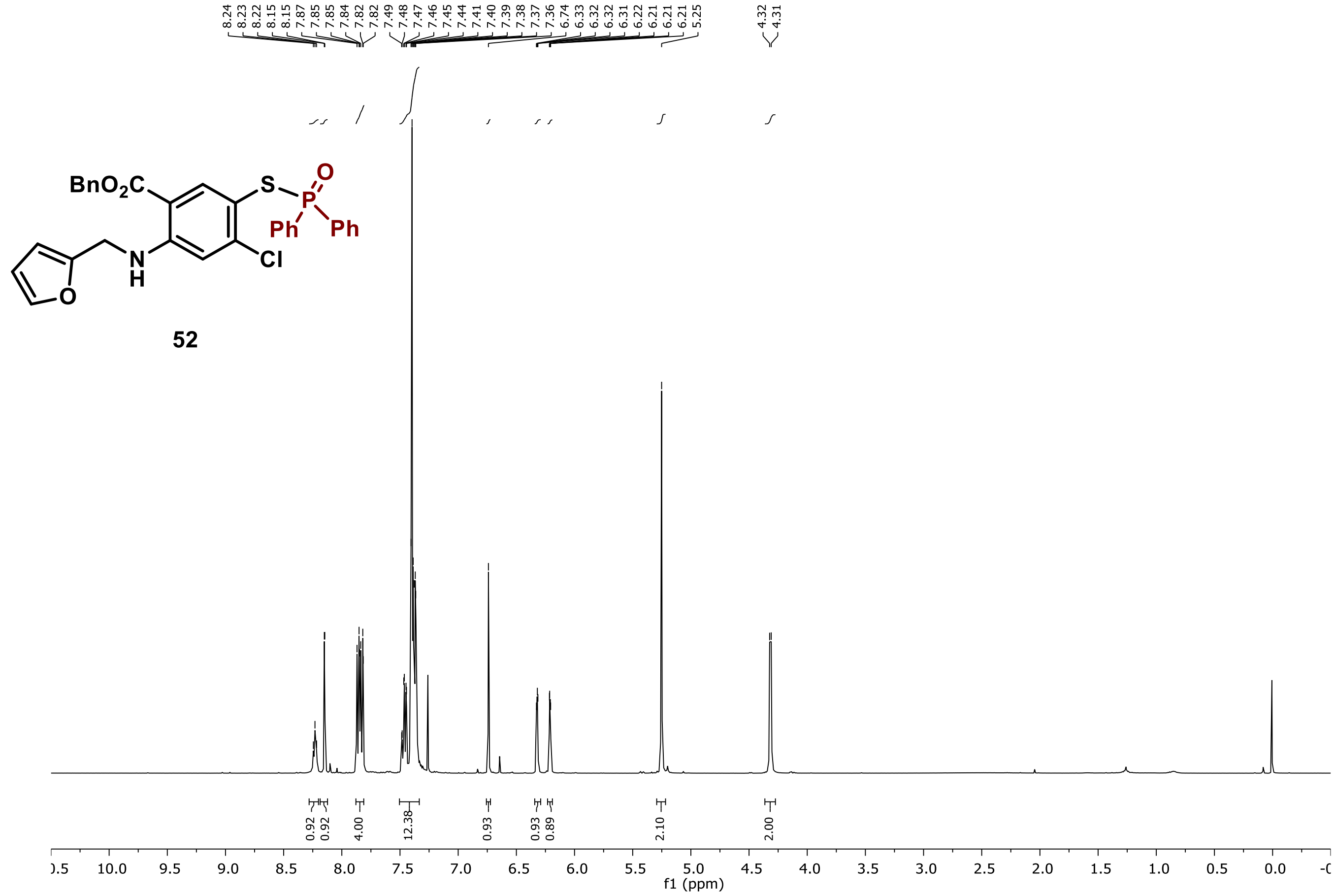

[13]C Spectra

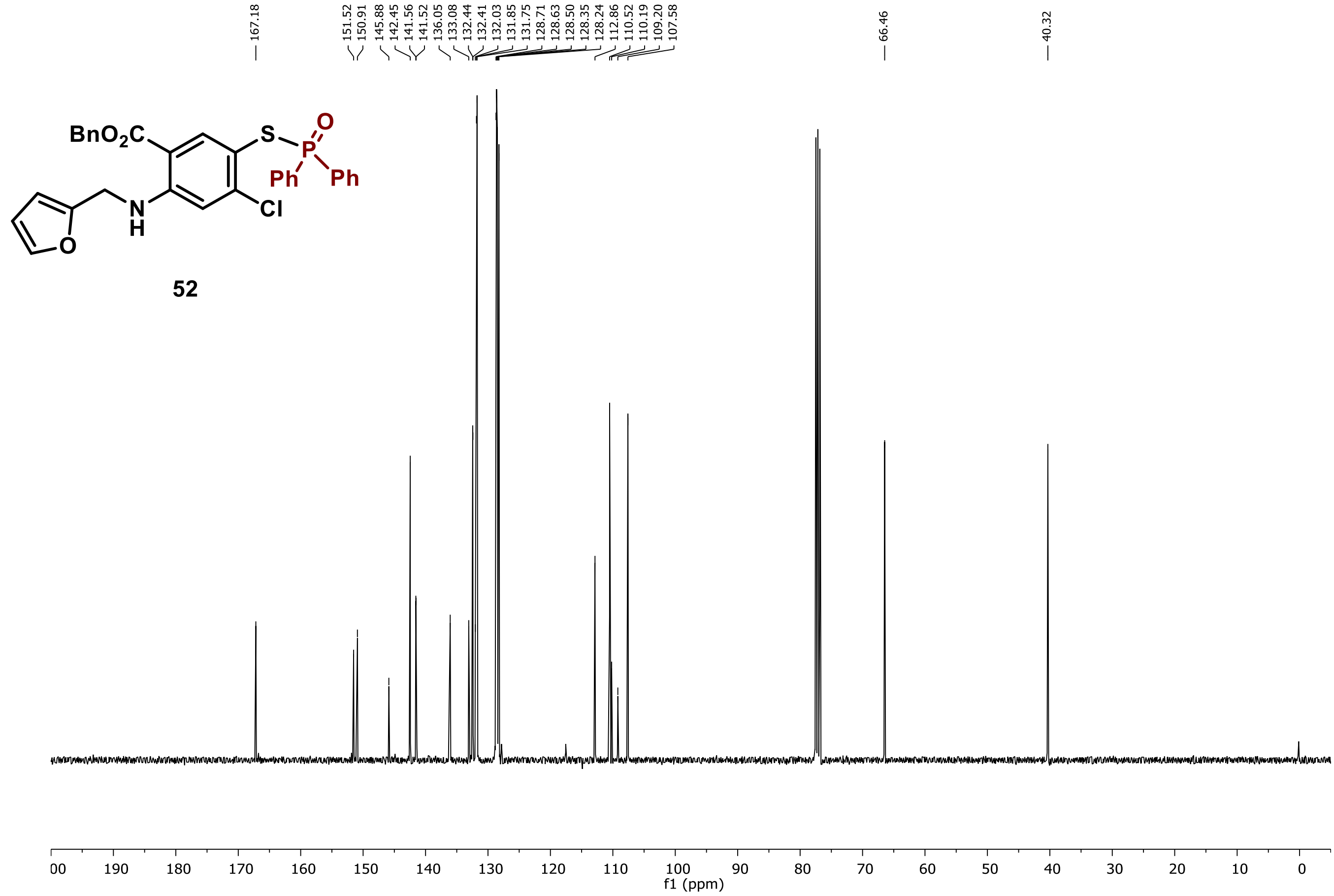

[1]H Spectra

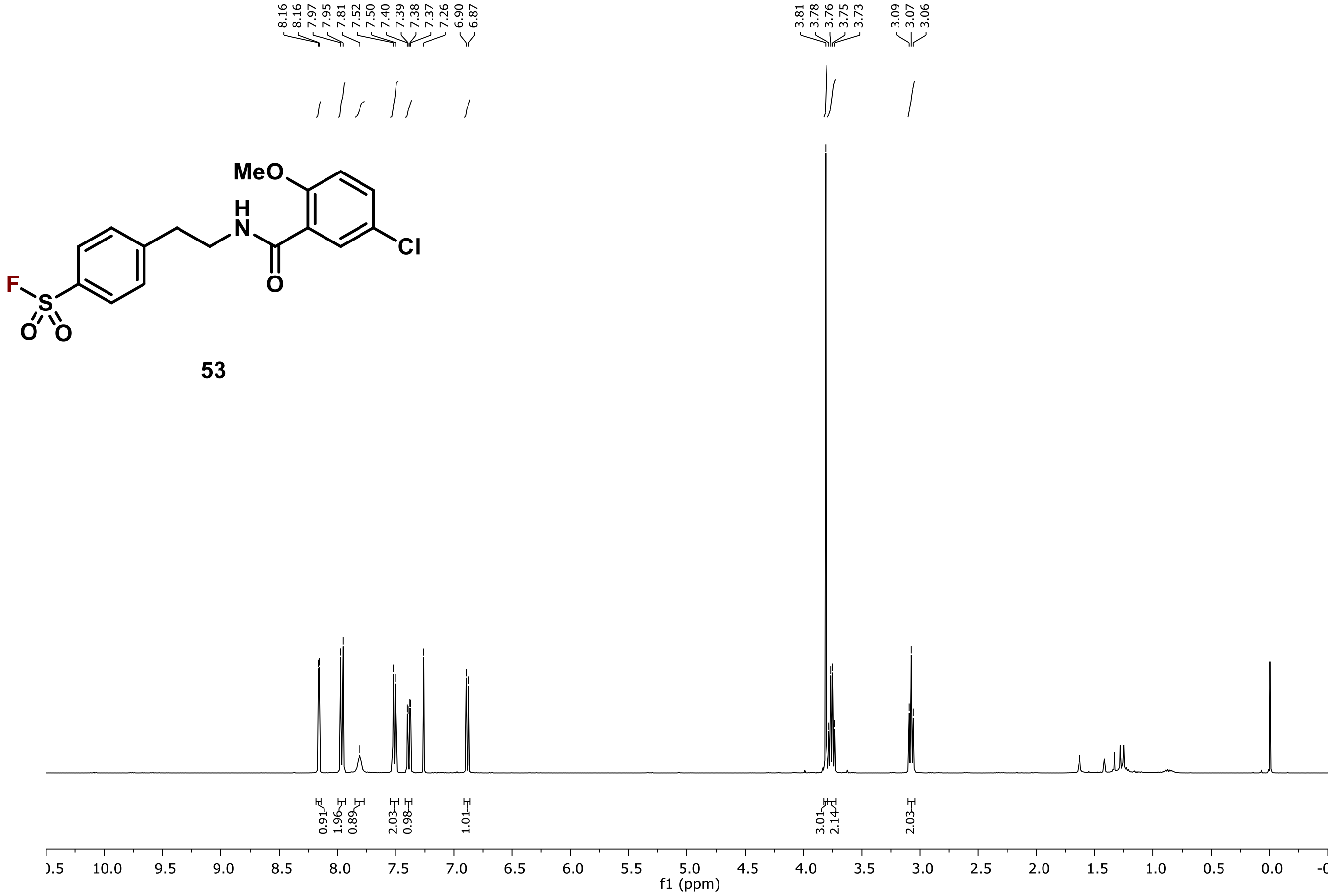

[13]C Spectra

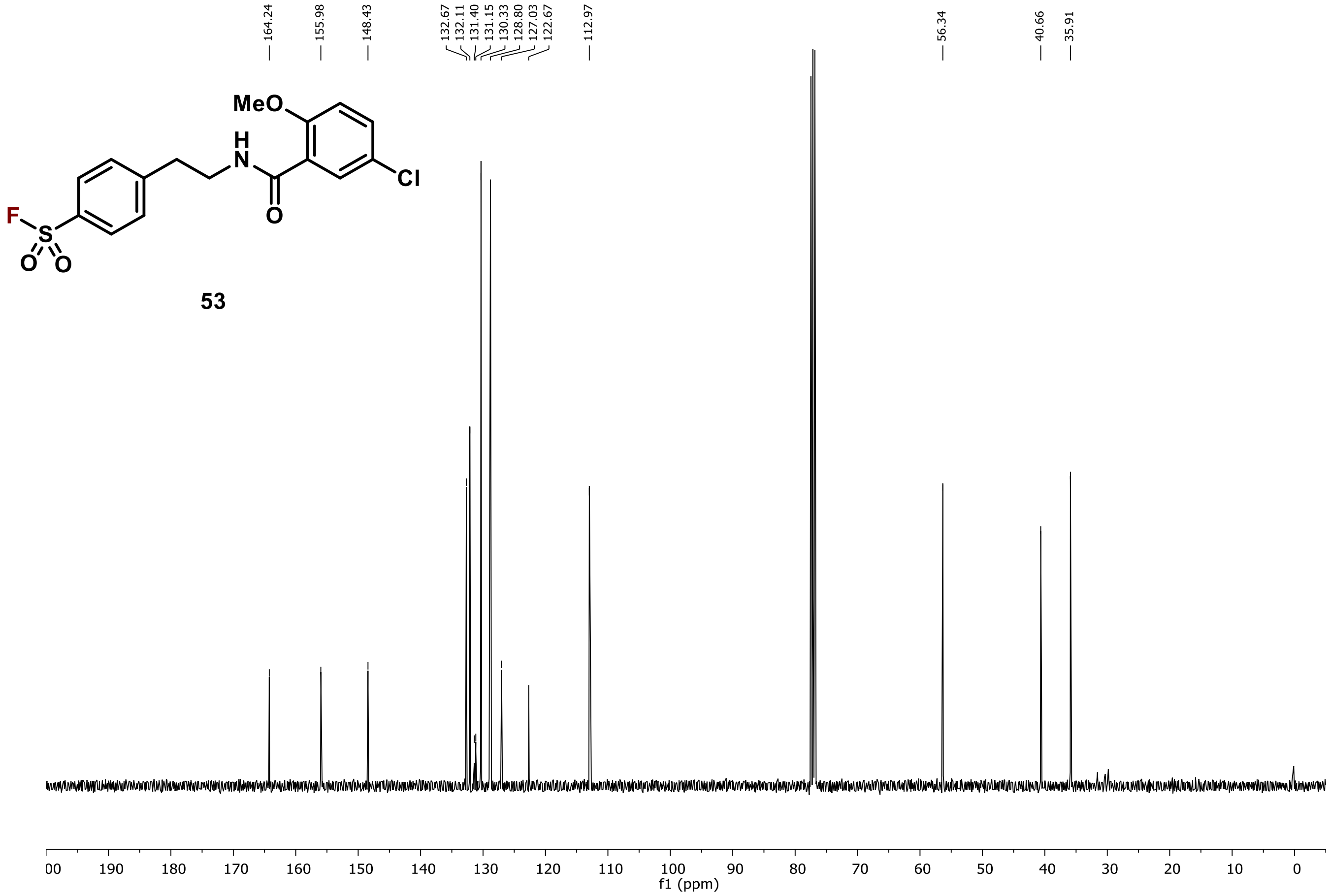

^{19}F Spectra

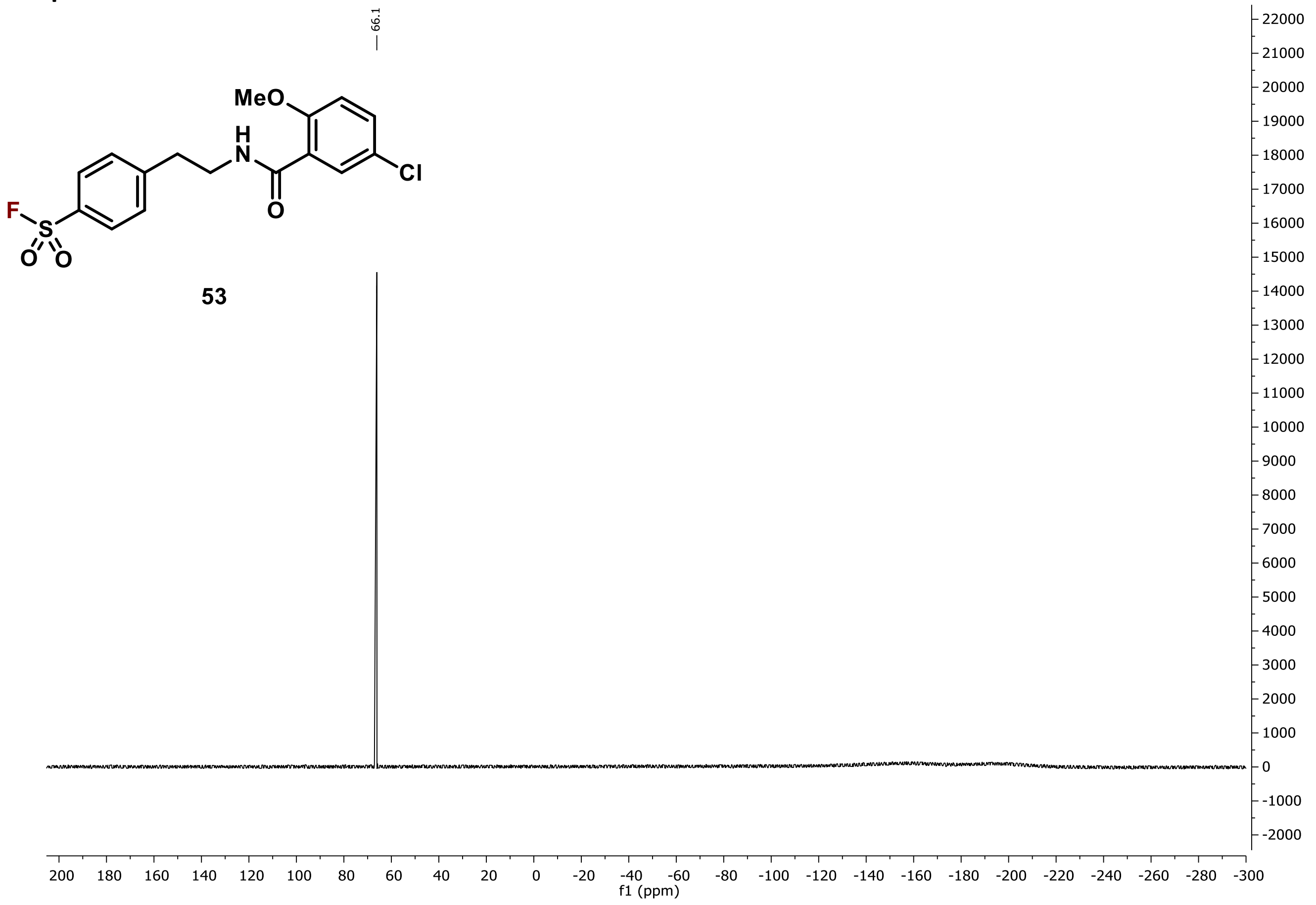

53
